# Phosphine-catalyzed [5+1] annulation of δ-sulfonamido-substituted enones with *N*-sulfonylimines: a facile synthesis of tetrahydropyridines†
†Electronic supplementary information (ESI) available. CCDC 1575011. For ESI and crystallographic data in CIF or other electronic format see DOI: 10.1039/c7sc04515h


**DOI:** 10.1039/c7sc04515h

**Published:** 2018-01-05

**Authors:** Leijie Zhou, Chunhao Yuan, Yuan Zeng, Honglei Liu, Chang Wang, Xing Gao, Qijun Wang, Cheng Zhang, Hongchao Guo

**Affiliations:** a Department of Applied Chemistry , China Agricultural University , Beijing 100193 , China . Email: hchguo@cau.edu.cn

## Abstract

Phosphine-catalyzed [5+1] annulation of δ-sulfonamido-substituted enones with *N*-sulfonylimines for the synthesis of 1,2,3,6-tetrahydropyridines is developed. The reaction proceeds smoothly under mild reaction conditions to give the annulation products in moderate to excellent yields. Mechanistic exploration of this new annulation shows that the δ-sulfonamido-substituted enone and the *N*-sulfonylimine serve as C5 and C1 synthons to furnish the annulation, respectively. Using chiral phosphine as the catalyst, an asymmetric variant of the model reaction gave the chiral product in up to 73% ee.

## Abstract 2

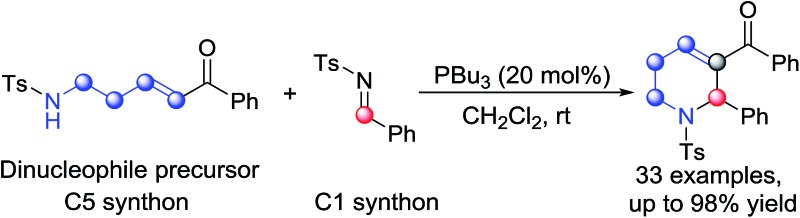
The first phosphine-catalyzed [5+1] annulation of enones with *N*-sulfonylimines works efficiently to give tetrahydropyridines.

## Introduction

Phosphine-catalyzed annulation reactions are powerful synthetic tools to construct carbo- and heterocycles.^1^ Since the pioneering work of Lu on phosphine catalysis,^2^ many types of phosphine-promoted annulation reaction such as [1+*n*],^3^ [2+*n*],^4^ [3+*n*],^5^ and [4+*n*]^6^ annulations have been developed. In these reactions, the reactive intermediates from nucleophilic addition of phosphine to activated allenes, Morita–Baylis–Hillman carbonates, activated alkynes, *etc.*, namely phosphorus ylides, serve as one-, two-, three-, or four-membered synthons when reacting with a variety of electrophilic coupling partners. Despite the fact that extremely diverse annulation reactions have been developed in the past two decades,^1^ the development of phosphine catalysis is reaching its limit since phosphine catalysis is stuck with a single activation mode. Therefore, exploration of new activation modes and synthons is very significant. Generally, phosphorus ylides work as equal to or less than four-membered synthons in phosphine catalysis. Examples with phosphorus ylides as greater than or equal to five-membered synthons, which could probably be used for synthesis of six-membered or medium-ring cyclic compounds, have not been reported.

**Scheme 1:**
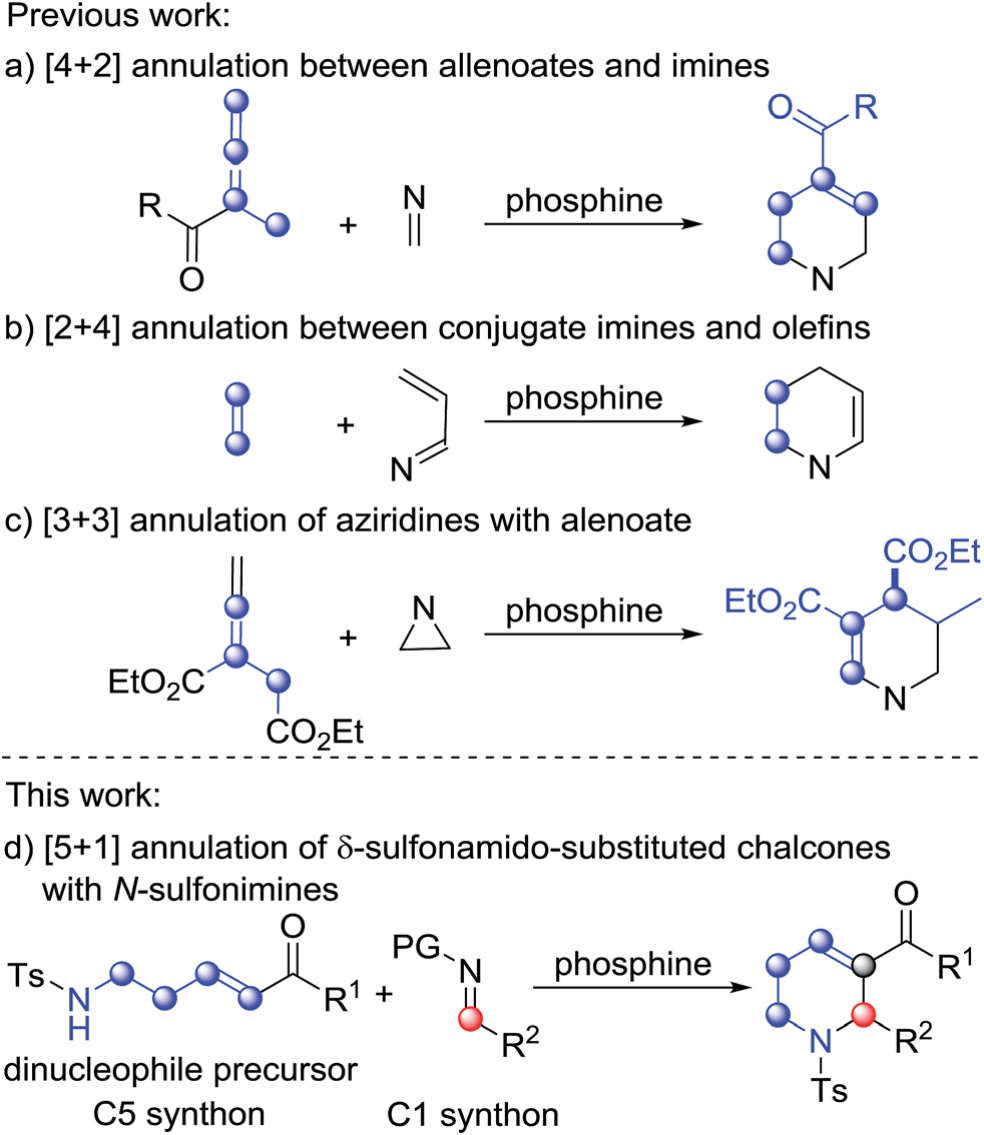
Typical reactions involving phosphine catalysis to construct tetrahydropyridines.

Functionalized tetrahydropyridines are important structural motifs of numerous biologically active natural products and synthetic pharmaceuticals, and their synthesis has attracted much attention.^7^ In the area of phosphine catalysis, several attractive strategies involving phosphine-catalyzed annulation reactions have been established for the synthesis of functionalized tetrahydropyridines. In 2003, Kwon described PBu_3_-catalyzed [4+2] annulation of imines with allenes as a facile pathway to access functionalized tetrahydropyridines (Scheme 1a).^8^ Two years later, through the use of a bulky *tert*-butyl-substituted binaphthyl-based chiral phosphine as the catalyst, Fu accomplished asymmetric versions of the above [4+2] reactions with excellent enantioselectivities.^9^ After the work of Kwon and Fu, Shi,10*a* Marinetti10*b* and Zhao10*c* made great contributions to the development of this classic [4+2] annulation reaction, and the reaction was also utilized by Kwon as a key step in the synthesis of natural products.^11^ In 2012, Loh and Zhong reported the phosphine-catalyzed asymmetric [2+4] annulation of olefins with conjugate imines, which provided an alternative approach to the synthesis of enantioenriched tetrahydropyridines (Scheme 1b).^12^ After the work, Chi,13*a* Shi,13*b* Wu13*c* and Zhang13*d* enriched this reaction by introducing intramolecular modes or other types of catalysts. The [3+3] annulation mode is another typical way to synthesize functionalized tetrahydropyridines. In 2009, Kwon developed the first phosphine-promoted [3+3] annulation of aziridines with allenoates to afford highly functionalized tetrahydropyridines under mild conditions,^14^ broadening the synthetic strategies of tetrahydropyridines by utilizing different types of building block (Scheme 1c). Herein, as the initial attempt of our exploration of new five-membered linear synthons for the [5+*n*] annulation reaction, we report a phosphine-catalyzed [5+1] annulation reaction of δ-sulfonamido-substituted enones with *N*-sulfonylimines (Scheme 1d). To the best of our knowledge, this is the first phosphine-catalyzed [5+1] annulation with a phosphorus ylide as a five-membered synthon.

## Results and discussion

At the outset of our experiment, the reaction between δ-sulfonamido-substituted enone **1a** and *N*-sulfonylimine **2a** was chosen as the model reaction, and various Lewis bases such as phosphines and amines were examined as the catalyst (Table 1). PPh_3_ (20 mol%) did not show any catalytic activity, and no annulation product was observed after the reaction mixture was stirred at rt for 72 h (Table 1, entry 1). Under otherwise identical conditions, the [5+1] annulation product **3aa** was obtained in 20% yield when MePPh_2_ was employed as the catalyst (entry 2). It seems that more nucleophilic phosphines were beneficial to the reaction. With the use of Me_2_PPh as the catalyst, the reaction worked at rt for 36 h to give the product **3aa** in 75% yield (entry 3). Compared with Me_2_PPh, more nucleophilic Bu_3_P displayed much better catalytic activity, greatly shortening the reaction time to 3 h to afford the product **3aa** in 95% yield (entry 4). Lowering the catalyst loading to 10 mol% still resulted in the product in 90% yield, albeit requiring a reaction time of 28 h (entry 5). However, when the catalyst loading was lowered to 5 mol%, the yield of **3aa** was greatly decreased to 15% (entry 6). With the use of organic amines such as Et_3_N, DMAP and DABCO instead of phosphines as the catalyst, no annulation product was observed even when the reaction time was prolonged to 72 h under otherwise identical conditions (entries 7–9). A stronger Lewis base DBU displayed certain catalytic activity, promoting the reaction to afford the annulation product **3aa** in 20% yield (entry 10).

**Table 1:** Screening of reaction conditions^*a*^

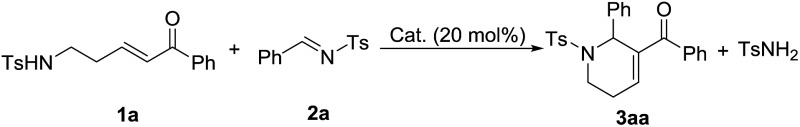			
Entry	Cat.	*t* (h)	Yield^*b*^ (%)
1	Ph_3_P	72	NR^*e*^
2	MePPh_2_	72	20
3	Me_2_PPh	36	75
4	Bu_3_P	3	95
5^*c*^	Bu_3_P	28	90
6^*d*^	Bu_3_P	72	15
7	Et_3_N	72	NR
8	DMAP	72	NR
9	DABCO	72	NR
10	DBU	72	20

^*a*^Unless otherwise stated, all reactions were carried out with **1a** (0.1 mmol), **2a** (0.15 mmol), and catalyst (0.02 mmol) in CH_2_Cl_2_ (1 mL) at rt.

^*b*^Isolated yield.

^*c*^10 mol% PBu_3_ was used.

^*d*^5 mol% PBu_3_ was used.

^*e*^No reaction.

After the optimal conditions were determined, various *N*-sulfonylimines with different substituents were carefully investigated (Table 2). The results indicated that imines with either electron-deficient or electron-rich substituents on the benzene ring are suitable substrates, and the corresponding 1,2,3,6-tetrahydropyridine derivatives were obtained with usually good to high yields. However, the position of the substituent on the benzene ring had a remarkable influence on the reaction. For example, the 2-Cl, 2-Br, and 2-Me substituted aryl imines led to lower yields of the products compared with their 3- or 4-substituted counterparts (entry 5 *vs.* entries 6–7, entry 8 *vs.* entries 9–10, entry 11 *vs.* entries 12–13). The 2-thiophenyl and 2-naphthyl imines were also compatible substrates under the optimal reaction conditions and the corresponding products were obtained in excellent yields (entries 16–17). The *N*-Boc-3-indole derived imine **2r** also underwent the reaction, providing the product **3ar** in 72% yield (entry 18). Unfortunately, the alkyl *N*-sulfonylimine **2s** did not perform the reaction and no desired product was observed (entry 19). The structure of the [5+1] annulation product was unambiguously determined through X-ray crystallographic analysis of the product **3aq**.^15^

**Table 2:** Substrate scope of *N*-sulfonylimines^*a*^

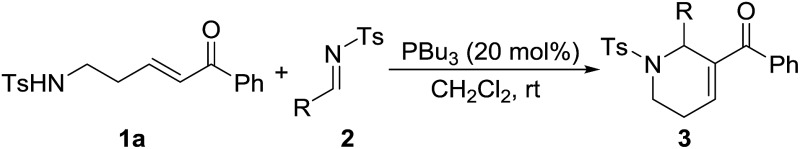				
Entry	R	*t* (h)	**3**	Yield^*b*^ (%)
1	Ph (**2a**)	3	**3aa**	95
2	2-FC_6_H_4_ (**2b**)	2	**3ab**	96
3	3-FC_6_H_4_ (**2c**)	6	**3ac**	85
4	4-FC_6_H_4_ (**2d**)	2	**3ad**	93
5	2-ClC_6_H_4_ (**2e**)	12	**3ae**	75
6	3-ClC_6_H_4_ (**2f**)	9	**3af**	82
7	4-ClC_6_H_4_ (**2g**)	1	**3ag**	85
8	2-BrC_6_H_4_ (**2h**)	16	**3ah**	68
9	3-BrC_6_H_4_ (**2i**)	6	**3ai**	81
10	4-BrC_6_H_4_ (**2j**)	2	**3aj**	84
11	2-MeC_6_H_4_ (**2k**)	28	**3ak**	41
12	3-MeC_6_H_4_ (**2l**)	48	**3al**	79
13	4-MeC_6_H_4_ (**2m**)	10	**3am**	95
14	3-MeOC_6_H_4_ (**2n**)	8	**3an**	90
15	4-MeOC_6_H_4_ (**2o**)	6	**3ao**	98
16	2-Thiophenyl (**2p**)	0.75	**3ap**	95
17	2-Naphthyl (**2q**)	2	**3aq**	98
18	*N*-Boc-3-indole (**2r**)	24	**3ar**	72
19	Et (**2s**)	72	**3as**	NR^*c*^

^*a*^Unless otherwise stated, all reactions were carried out with **1a** (0.2 mmol), **2** (0.3 mmol), and PBu_3_ (0.04 mmol) in CH_2_Cl_2_ (2 mL) at rt.

^*b*^Isolated yield.

^*c*^No reaction.

As shown in Table 3, a series of functionalized ketones with variations of the R group were examined under the optimal reaction conditions. The results showed that no matter what the electronic properties or the substitution positions of the substituents, such as F-, Cl-, Br-, Me-, MeO-, and –NO_2_ substituted enones at the benzene ring, the reactions proceeded smoothly to afford the desired 1,2,3,6-tetrahydropyridine derivatives with good to excellent yields (entries 1–12). However, the MeO and NO_2_ substituted substrates required longer times to finish the reaction (entries 10–12). In addition, the 2-thiophenyl and 2-naphthyl modified enones underwent the [5+1] annulation reaction to produce the corresponding products in excellent yields (entries 13–14). To our delight, aliphatic enone **1s** underwent this reaction to afford the desired product **3sa** in 73% yield (entry 15).

**Table 3:** Substrate scope of δ-sulfonamido-substituted ketones^*a*^

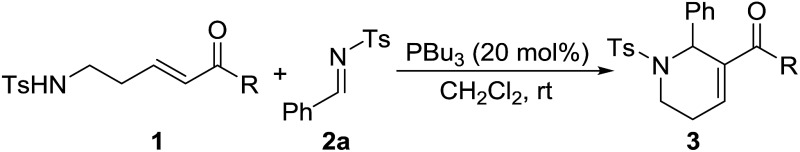				
Entry	R	*t* (h)	**3**	Yield^*b*^ (%)
1	2-FC_6_H_4_ (**1b**)	4	**3ba**	97
2	4-FC_6_H_4_ (**1c**)	3	**3ca**	90
3	2-ClC_6_H_4_ (**1d**)	2	**3da**	90
4	3-ClC_6_H_4_ (**1e**)	3	**3ea**	91
5	4-ClC_6_H_4_ (**1f**)	3	**3fa**	84
6	3-BrC_6_H_4_ (**1g**)	5	**3ga**	98
7	4-BrC_6_H_4_ (**1h**)	5	**3ha**	87
8	3,4-Cl_2_C_6_H_3_ (**1i**)	1.5	**3ia**	90
9	4-MeC_6_H_4_ (**1j**)	5	**3ja**	91
10	2-MeOC_6_H_4_ (**1k**)	12	**3ka**	98
11	3-MeOC_6_H_4_ (**1l**)	12	**3la**	98
12	4-NO_2_C_6_H_4_ (**1m**)	36	**3ma**	75
13	2-Thiophenyl (**1n**)	12	**3na**	93
14	2-Naphthyl (**1o**)	3	**3oa**	88
15	Me (**1s**)	16	**3sa**	73

^*a*^Unless otherwise stated, all reactions were carried out with **1** (0.2 mmol), **2a** (0.3 mmol), and PBu_3_ (0.04 mmol) in CH_2_Cl_2_ (2 mL) at rt.

^*b*^Isolated yield.

**Scheme 2:**
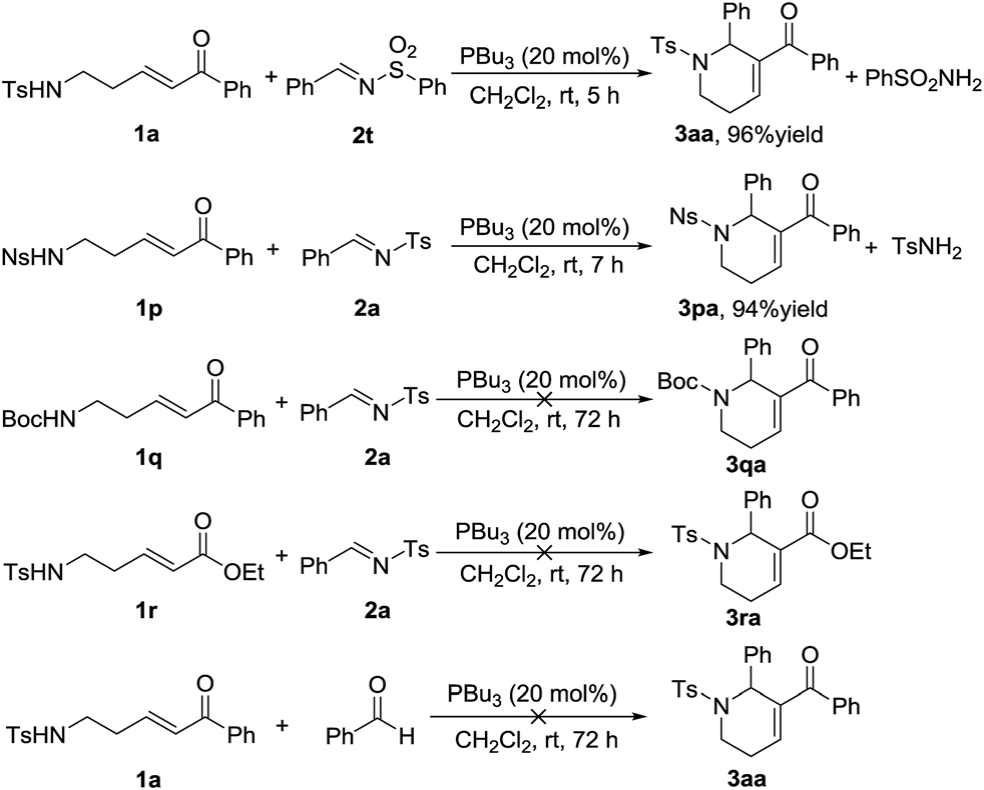
Control experiments.

Following the substrate scope evaluation, we attempted to figure out how the reaction works. As a result, several control experiments were carried out under the optimal reaction conditions (Scheme 2). In the presence of PBu_3_, treatment of enone **1a** with *N*-sulfonylimine **2t** afforded the annulation product **3aa** in 96% yield together with benzenesulfonamide. In contrast, treatment of enone **1p** with imine **2a** afforded the annulation product **3pa** in 94% yield together with *p*-toluenesulfonamide. By comparison, δ-Boc-amido-substituted enones did not undergo annulation in the presence of phosphine. Meanwhile, when the benzoyl group was replaced by an ester group, the reaction did not work either. Therefore, the acidity of the substituted amine group at the δ-position of the ketone probably has a remarkable impact on the reaction process, and so does the benzoyl group. Replacement of the imine substrate with benzaldehyde did not yield the desired annulation product (Scheme 2). These results demonstrated that the amino-group in the annulation product comes from the enone substrate.

**Scheme 3:**
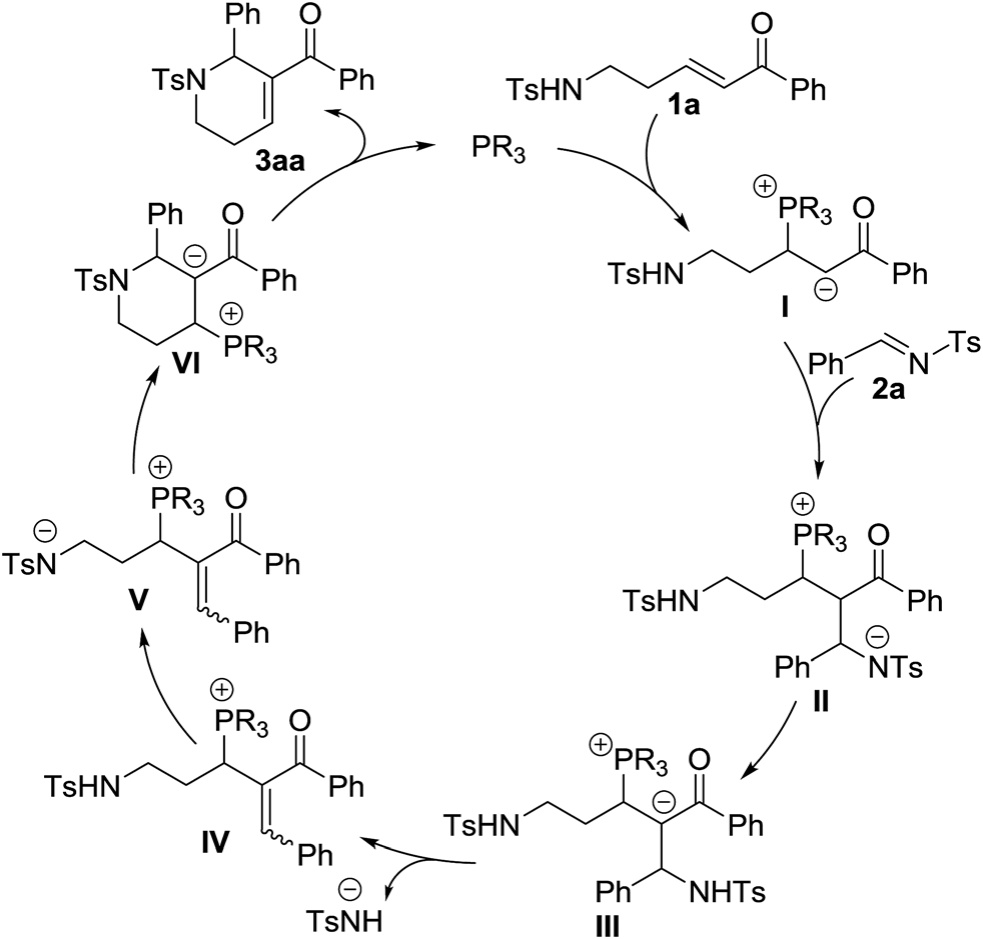
A plausible mechanism.

On the basis of the results obtained, a plausible mechanism was proposed (Scheme 3).^1^ Nucleophilic addition of the phosphine catalyst to enone **1a** produces a zwitterionic intermediate **I**, which then performs another nucleophilic addition to imine **2a** to afford the intermediate **II**. The intermediate **III** generated from the intermediate **II***via* an intramolecular proton transfer eliminates a 4-methylbenzenesulfonamide anion to produce the intermediate **IV**. Through the abstraction of a proton, the intermediate **IV** is transformed into the intermediate **V**. Subsequent intramolecular nucleophilic addition furnishes annulation to form the intermediate **VI**, which regenerates the phosphine catalyst to give the final annulation product **3aa**.

**Scheme 4:**
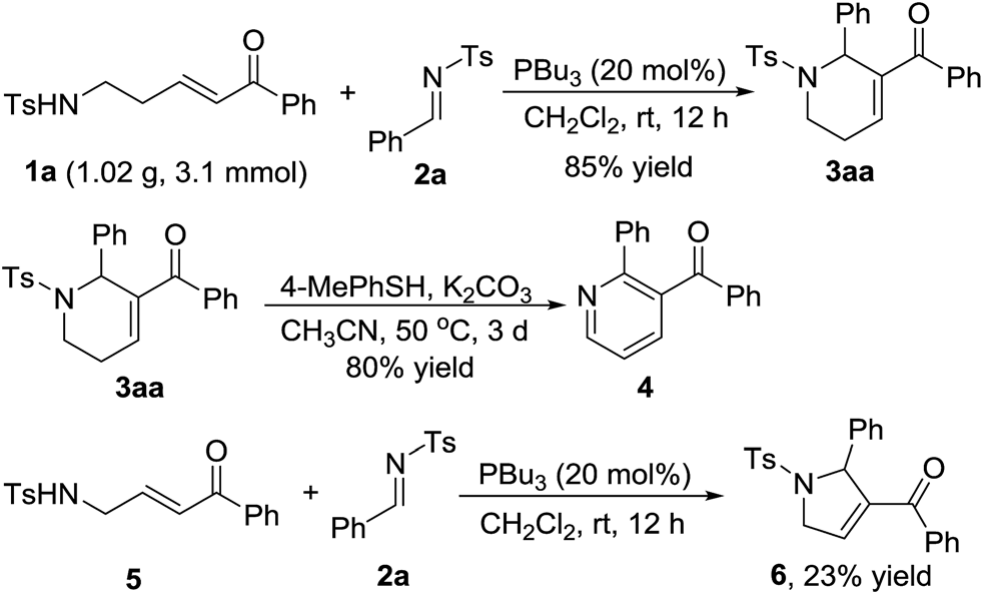
The reaction on the gram-scale and further transformations.

As indicated in Scheme 4, a gram-scale preparation of the product **3aa** was carried out. 1.02 g of enone **1a** (3.1 mmol) reacted with *N*-sulfonylimine **2a** (1.22 g, 4.7 mmol) under the optimal reaction conditions to give 1,2,3,6-tetrahydropyridine derivative **3aa** in 85% yield. Treatment of **3aa** with 4-methylbenzenethiol and K_2_CO_3_ in air provided a good yield of the pyridine derivative **4***via* dehydrogenation aromatization. Further exploration on the variety of the sulfonamido-substituted enone indicated that enone **5**, which is a homologue of enone **1a**, could work as a C4 synthon to perform the [4+1] annulation reaction to give 2,5-dihydro-1*H*-pyrrole derivative **6** in 23% yield (Scheme 4).

**Scheme 5:**
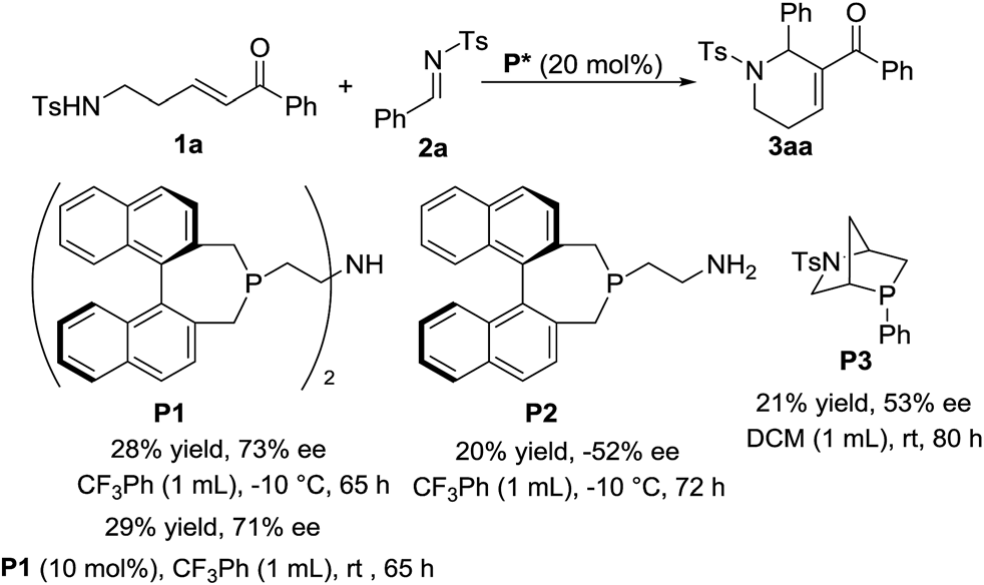
Investigation of the asymmetric [5+1] annulation.

As shown in Scheme 5, the asymmetric version of the reaction was also investigated. To our delight, with the use of chiral phosphine **P1** as the catalyst and CF_3_Ph as the solvent, the [5+1] annulation of enone **1a** with *N*-sulfonylimine **2a** worked at –10 °C for 65 h to give chiral product **3aa** in 28% yield with up to 73% ee. When we decreased the amount of **P1** to 10 mol%, the reaction worked at rt to give the product in 29% yield and 71% ee, which is similar to the result from the reaction using 20 mol% of the catalyst at low temperature. With the use of chiral phosphines **P2** or **P3** as the catalyst, moderate enantiomeric excesses were obtained. Unfortunately, a variety of attempts to improve enantioselectivity failed.

## Conclusions

In summary, we have developed a phosphine-catalyzed [5+1] annulation of δ-sulfonamido-substituted enones with *N*-sulfonimines to prepare tetrahydropyridines with good to excellent yields. The reaction has broad substrate scope for both enones and *N*-sulfonimines. A plausible mechanism was proposed according to the results of control experiments. In addition, the reaction on the gram-scale worked well and further transformation of the product provided the pyridine derivative. The asymmetric version of the model [5+1] annulation reaction was also investigated, and up to 73% ee was achieved.

## Conflicts of interest

There are no conflicts to declare.

## Supplementary Material

Supplementary information

Crystal structure data

## References

[cit1] Marinetti A., Voituriez A. (2010). Synlett.

[cit2] Zhang C., Lu X. (1995). J. Org. Chem..

[cit3] Szeto J., Sriramurthy V., Kwon O. (2011). Org. Lett..

[cit4] Meng X., Huang Y., Zhao H., Xie P., Ma J., Chen R. (2009). Org. Lett..

[cit5] Voituriez A., Panossian A., Fleury-Brégeot N., Retailleau P., Marinetti A. (2008). J. Am. Chem. Soc..

[cit6] Tran Y. S., Kwon O. (2007). J. Am. Chem. Soc..

[cit7] (c) Maison W., in Highlights in Bioorganic Chemistry: Pipecolic acid derivatives, Wiley-VCH, Weinheim, 2004, p. 18.

[cit8] Zhu X. F., Lan J., Kwon O. (2003). J. Am. Chem. Soc..

[cit9] Wurz R. P., Fu G. C. (2005). J. Am. Chem. Soc..

[cit10] Zhao G., Shi M. (2005). Org. Biomol. Chem..

[cit11] Tran Y. S., Kwon O. (2005). Org. Lett..

[cit12] Shi Z., Tong Q., Leong W. W. D., Zhong G. (2012). Chem.–Eur. J..

[cit13] Jin Z., Yang R., Du Y., Tiwari B., Ganguly R., Chi Y. R. (2012). Org. Lett..

[cit14] Guo H., Xu Q., Kwon O. (2009). J. Am. Chem. Soc..

[cit15] Crystallographic data for **3aq** have been deposited with the Cambridge Crystallographic Data Centre as deposition number CCDC ; 1575011.

