# Supplementary material for: Phosphine-catalyzed [5+1] annulation of δ-sulfonamido-substituted enones with N-sulfonylimines: a facile synthesis of tetrahydropyridines
Source: Chem Sci. 2018 Jan 5;9(7):1831–5. doi: 10.1039/c7sc04515h (PMC5892350; doi:10.1039/c7sc04515h)
Supplement: Supplementary file 1 [file SC-009-C7SC04515H-s001.pdf]

Electronic Supplementary Material (ESI) for Chemical Science.
This journal is © The Royal Society of Chemistry 2018

Electronic Supplementary Information

# Phosphine-Catalyzed [5+1] Annulation of δ-Sulfonamido-Substituted Enones with *N*-Sulfonylimines: A Facile Synthesis of Tetrahydropyridines

Leijie Zhou, Chunhao Yuan, Yuan Zeng, Honglei Liu, Chang Wang, Xing Gao, Qijun Wang, Cheng Zhang, and Hongchao Guo*

Department of Applied Chemistry, China Agricultural University, Beijing 100193, P. R. China

Fax: (+86) 10-6273-0784; E-mail: hchguo@cau.edu.cn

## Contents

General Information S2

Preparation of δ-Sulfonamido-Substituted enones **1** S2–S9

Preparation of *N*-Sulfonylimines **2** S9

General Procedure for Phosphine-Catalyzed [5+1] Annulation S9

Characterization Data for the Products **3** S10–S26

Scaled-up Synthesis and Transformation of the Product **3aa** S27

Exploration on the Phosphine-Catalyzed [4+1] Annulation S28–S29

Analytic and Characterization Data for the Chiral Product **3aa** S29

$^{1}$H and $^{13}$C NMR Spectra of all δ-Sulfonamido-Substituted enones **1** S30–S47

$^{1}$H and $^{13}$C NMR Spectra of All Products **3-6** S48–S83

HPLC Chromatograms of the Product **3aa** S84

X-Ray Crystallographic Data of **3aq** S85–S96

## General Information

All reactions were performed under $N_2$ atmospheres in oven-dried glassware with magnetic stirring. Unless otherwise stated, all reagents were purchased from commercial suppliers and used without further purification. All solvents were purified and dried according to standard methods prior to use. Organic solutions were concentrated under reduced pressure on a rotary evaporator or an oil pump. Reactions were monitored through thin layer chromatography (TLC) on silica gel–precoated glass plates. Chromatograms were visualized by fluorescence quenching with UV light at 254 nm. Flash column chromatography was performed using Qingdao Haiyang flash silica gel (200–300 mesh). Infrared spectra were recorded using a Bruker Optics TENSOR 27 instrument. $^1$H and $^{13}$C NMR spectra were recorded in $CDCl_3$ or DMSO-$d_6$ using a 300 MHz NMR instrument (referenced internally to $Me_4Si$). $^1$H NMR data are reported as follows: chemical shift, multiplicity (s = singlet; d = doublet; q = quartet; m = multiplet; br = broad), coupling constant (Hz), and integral. Data for $^{13}$C NMR spectra are reported in terms of chemical shift. Optical rotation was obtained on an Autopol VI automatic polarimeter. Accurate mass measurements were performed using an Agilent instrument with the ESI-MS technique. Melting points were determined on a Stuard SMP3 melting point apparatus. X-ray crystallographic data were collected using a MM007HF Saturn724+. HPLC analysis was performed on Agilent 1100 or 1200 series, UV detection monitored at 254 nm, using Chiralpak RC-OD column with hexane and *i*-PrOH as the eluent.

## General procedure for preparation of δ-Sulfonamido-substituted enones 1[1]

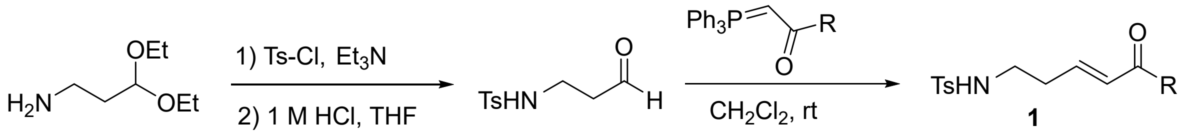


δ-Sulfonamido-substituted enones were prepared according to a reported procedure. To a solution of 1-amino-3,3-diethoxyaminopropane (7.36 g, 50.0 mmol) in $CH_2Cl_2$ (200 mL) was added $Et_3N$ (8.30 mL, 60.0 mmol). The solution was cooled to 0 °C and p-toluenesulfonyl chloride (10.5 g, 55.0 mmol, 1.0 equiv) in $CH_2Cl_2$ (100 mL) was added over 30 min. The resulting mixture was allowed to warm to room temperature and treated with saturated aqueous $NH_4Cl$ solution. The layers were separated, the organic layer was extracted with $CH_2Cl_2$, dried over sodium sulfate, and concentrated to a yellow

[1] B.-L. Zhao, Y. Lin, H.-H. Yan, D.-M. Du. *Org. Biomol. Chem.* **2015**, *13*, 11351–11361.

oil. The crude sulfonamide was dissolved in THF (100 mL), treated with 1 M HCl (50 mL), and stirred at room temperature about 3 h. Upon complete consumption of the acetal as judged by TLC analysis (petroleumether/EtOAc 1:1), EtOAc (100 mL) was added and the layers were separated. The organic layer was washed ($H_2O$, brine), dried over $Na_2SO_4$ and concentrated. Wittig reagent (5 mmol, 1 equiv) was added to a solution of the crude tosylamino propaldehyde (5 mmol, 1 equiv) in $CH_2Cl_2$ (30 mL) in round bottom flask. The solution was stirred at room temperature for 30 h. After concentration under reduced pressure, the residue was purified by flash chromatography on silica gel (petroleumether/EtOAc 3:1) to afford δ-Sulfonamido-substituted enones **1**.

**(*E*)-4-Methyl-*N*-(5-oxo-5-phenylpent-3-en-1-yl)benzenesulfonamide (1a)**

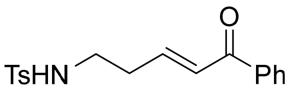


Prepared according to the general procedure as a white solid. $^1$H NMR (400 MHz, Chloroform-d) δ 8.01 – 7.83 (m, 2H), 7.85 – 7.67 (m, 2H), 7.66 – 7.54 (m, 1H), 7.51 – 7.46 (m, 2H), 7.30 (d, J = 8.2 Hz, 2H), 6.94 – 6.77 (m, 2H), 5.06 (t, J = 6.3 Hz, 1H), 3.18 (q, J = 6.6 Hz, 2H), 2.52 (q, J = 6.4 Hz, 2H), 2.41 (s, 3H); $^{13}$C NMR (101 MHz, CDCl3) δ 190.1, 144.3, 143.6, 137.4, 136.8, 132.9, 129.8, 128.6, 128.0, 127.1, 41.6, 32.9, 21.5; HRMS (ESI) calcd for $C_{18}H_{19}NO_3SH^+$ $[M+H]^+$ 330.1158, found 330.1156.

**(*E*)-*N*-(5-(2-Fluorophenyl)-5-oxopent-3-en-1-yl)-4-methylbenzenesulfonamide (1b)**

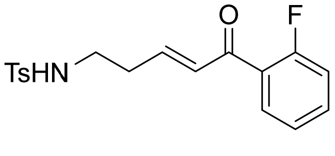


Prepared according to the general procedure as a white solid. $^1$H NMR (400 MHz, Chloroform-d) δ 7.79 – 7.73 (m, 2H), 7.72 – 7.70 (m, 1H), 7.54 – 7.48 (m, 1H), 7.37 – 7.26 (m, 2H), 7.24 – 7.22 (m, J = 7.6, 1H), 7.16 – 7.10 (m, 1H), 6.83 – 6.79 (m, 1H), 6.74 – 6.69 (m, 1H), 5.06 (t, J = 6.3 Hz, 1H), 3.15 (q, J = 6.7 Hz, 2H), 2.50 – 2.46 (m, 2H), 2.41 (s, 3H); $^{13}$C NMR (101 MHz, $CDCl_3$) δ 188.8 (d, J = 2.3 Hz), 161.1 (d, J = 253.5 Hz), 144.7, 143.5, 136.8, 134.1, 134.0, 131.6, 131.5, 130.9, 130.8, 129.8, 127.0, 126.5, 126.4, 124.5, 124.4, 116.5 (d, J = 23.1 Hz), 41.5, 32.7, 21.5; HRMS (ESI) calcd for $C_{18}H_{18}FNO_3SH^+$ $[M+H]^+$ 348.1064, found 348.1059.

**(*E*)-*N*-(5-(4-Fluorophenyl)-5-oxopent-3-en-1-yl)-4-methylbenzenesulfonamide (1c)**

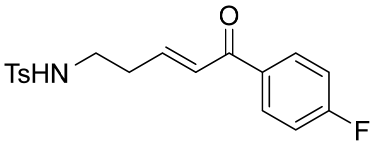


Prepared according to the general procedure as a white solid. $^{1}$H NMR (400 MHz, Chloroform-d) δ 8.10 – 7.86 (m, 2H), 7.83 – 7.66 (m, 2H), 7.34 – 7.25 (m, 2H), 7.20 – 7.06 (m, 2H), 6.94 – 6.82 (m, 2H), 5.22 (t, J = 6.3 Hz, 1H), 3.17 (q, J = 6.6 Hz, 2H), 2.53 – 2.50 (m, 2H), 2.41 (s, 3H). $^{13}$C NMR (101 MHz, $CDCl_3$) δ 188.5, 165.6 (d, J = 254.6 Hz), 144.6, 143.6, 136.7, 133.7, 133.7, 131.3, 131.2, 129.8, 127.6, 127.1, 127.0, 115.7 (d, J = 21.8 Hz), 41.6, 32.8, 21.5; HRMS (ESI) calcd for $C_{18}H_{18}FNO_3SH^+$ $[M+H]^+$ 348.1064, found 348.1060.

**(*E*)-*N*-(5-(2-Chlorophenyl)-5-oxopent-3-en-1-yl)-4-methylbenzenesulfonamide (1d)**

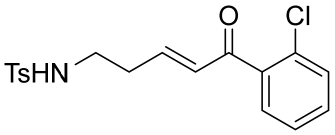


Prepared according to the general procedure as a white solid. $^{1}$H NMR (300 MHz, Chloroform-d) δ 7.77 – 7.70 (m, 2H), 7.41 – 7.37 (m, 2H), 7.35 – 7.26 (m, 4H), 6.69 – 6.35 (m, 2H), 5.17 (t, J = 6.3 Hz, 1H), 3.10 (q, J = 6.7 Hz, 2H), 2.48 – 2.44 (m, 2H), 2.41 (s, 3H); $^{13}$C NMR (75 MHz, $CDCl_3$) δ 193.1, 146.3, 143.2, 138.1, 136.4, 131.8, 131.1, 130.8, 129.9, 129.4, 129.3, 129.0, 126.8, 126.7, 126.4, 41.0, 32.4, 21.1; HRMS (ESI) calcd for $C_{18}H_{18}ClNO_3SH^+$ $[M+H]^+$ 364.0769, found 364.0763.

**(*E*)-*N*-(5-(3-Chlorophenyl)-5-oxopent-3-en-1-yl)-4-methylbenzenesulfonamide (1e)**

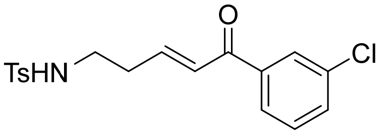


Prepared according to the general procedure as a white solid. $^{1}$H NMR (400 MHz, Chloroform-d) δ 7.85 (t, J = 1.9 Hz, 1H), 7.81 – 7.70 (m, 3H), 7.53 (ddd, J = 8.0, 2.1, 1.1 Hz, 1H), 7.40 (t, J = 7.8 Hz, 1H), 7.34 – 7.21 (m, 2H), 7.01 – 6.74 (m, 2H), 5.24 (t, J = 6.3 Hz, 1H), 3.17 (q, J = 6.6 Hz, 2H), 2.57 – 2.45 (m, 2H), 2.41 (s, 3H); $^{13}$C NMR (101 MHz, $CDCl_3$) δ 188.8, 145.4, 143.6, 139.0, 136.7, 134.8, 132.8, 129.9, 129.8, 128.5, 127.5, 127.1, 127.0, 126.7, 41.5, 32.9, 21.5; HRMS (ESI) calcd for $C_{18}H_{18}ClNO_3SH^+$ $[M+H]^+$ 364.0769, found 364.0765.

**(*E*)-*N*-(5-(4-Chlorophenyl)-5-oxopent-3-en-1-yl)-4-methylbenzenesulfonamide (1f)**

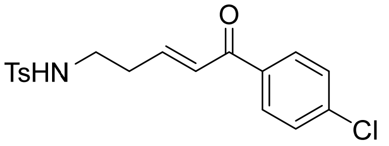


Prepared according to the general procedure as a white solid. $^1$H NMR (400 MHz, Chloroform-d) δ 7.94 – 7.79 (m, 2H), 7.81 – 7.70 (m, 2H), 7.50 – 7.37 (m, 2H), 7.39 – 7.20 (m, 2H), 6.94 – 6.86 (m, 2H), 5.08 (t, J = 6.3 Hz, 1H), 3.17 (q, J = 6.6 Hz, 2H), 2.58 – 2.47 (m, 2H), 2.42 (s, 3H). $^{13}$C NMR (101 MHz, $CDCl_3$) δ 188.8, 144.9, 143.6, 139.3, 136.7, 135.7, 130.0, 129.9, 129.8, 129.0, 128.9, 127.6, 127.1, 127.0, 41.5, 32.9, 21.5; HRMS (ESI) calcd for $C_{18}H_{18}ClNO_3SH^+$ $[M+H]^+$ 364.0769, found 364.0765.

**(*E*)-*N*-(5-(3-Bromophenyl)-5-oxopent-3-en-1-yl)-4-methylbenzenesulfonamide (1g)**

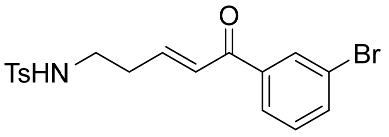


Prepared according to the general procedure as a white solid. $^1$H NMR (400 MHz, Chloroform-d) δ 8.02 (t, J = 1.8 Hz, 1H), 7.85 – 7.79 (m, 1H), 7.79 – 7.73 (m, 2H), 7.69 (ddd, J = 7.9, 2.0, 1.0 Hz, 1H), 7.39 – 7.24 (m, 3H), 6.97 – 6.79 (m, 2H), 5.09 (t, J = 6.3 Hz, 1H), 3.18 (q, J = 6.6 Hz, 2H), 2.58 – 2.48 (m, 2H), 2.42 (s, 3H); $^{13}$C NMR (101 MHz, $CDCl_3$) δ 188.6, 145.4, 143.6, 139.2, 136.7, 135.7, 131.5, 130.2, 129.8, 127.5, 127.1, 127.0, 122.9, 41.5, 32.9, 21.5; HRMS (ESI) calcd for $C_{18}H_{18}BrNO_3SH^+$ $[M+H]^+$ 408.0264, found 408.0262.

**(*E*)-*N*-(5-(4-Bromophenyl)-5-oxopent-3-en-1-yl)-4-methylbenzenesulfonamide (1h)**

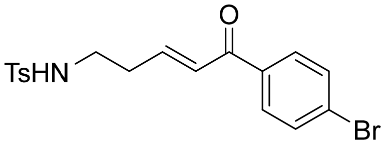


Prepared according to the general procedure as a white solid. $^1$H NMR (400 MHz, Chloroform-d) δ 7.85 – 7.69 (m, 4H), 7.69 – 7.54 (m, 2H), 7.40 – 7.20 (m, 2H), 6.92 – 6.81 (m, 2H), 5.18 (t, J = 6.3 Hz, 1H), 3.17 (q, J = 6.6 Hz, 2H), 2.51 (dtd, J = 6.7, 5.3, 1.9 Hz, 2H), 2.42 (s, 3H); $^{13}$C NMR (101 MHz, $CDCl_3$) δ 189.0, 145.0, 143.6, 136.7, 136.1, 131.9, 131.8, 130.1, 130.0, 129.8, 129.6, 128.0, 127.5, 127.1, 127.0, 41.5, 32.8, 21.5; HRMS (ESI) calcd for $C_{18}H_{18}BrNO_3SH^+$ $[M+H]^+$ 408.0264, found 408.0263.

**(*E*)-*N*-(5-(3,4-Dichlorophenyl)-5-oxopent-3-en-1-yl)-4-methylbenzenesulfonamide (1i)**

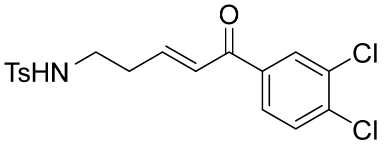


Prepared according to the general procedure as a pale yellow solid. $^1$H NMR (400 MHz, Chloroform-d) δ 7.97 (d, J = 2.0 Hz, 1H), 7.81 – 7.67 (m, 3H), 7.53 (d, J = 8.3 Hz, 1H), 7.37 – 7.23 (m, 2H), 7.00 – 6.76 (m, 2H), 5.19 (t, J = 6.3 Hz, 1H), 3.17 (q, J = 6.5 Hz, 2H), 2.55 – 2.51 (m, 2H), 2.42 (s, 3H); $^{13}$C NMR (101 MHz, $CDCl_3$) δ 187.6, 145.8, 143.6, 137.4, 136.9, 136.7, 133.2, 130.8, 130.7, 130.4, 129.8, 129.6, 127.6, 127.1, 127.0, 126.9, 41.5, 32.9, 21.5. HRMS (ESI) calcd for $C_{18}H_{18}Cl_2NO_3SH^+$ $[M+H]^+$ 398.0379, found 398.0377.

**(*E*)-4-Methyl-*N*-(5-oxo-5-(p-tolyl)pent-3-en-1-yl)benzenesulfonamide (1j)**

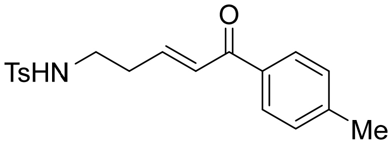


Prepared according to the general procedure as a white solid. $^1$H NMR (400 MHz, Chloroform-d) δ 7.81 – 7.72 (m, 2H), 7.52 – 7.43 (m, 2H), 7.37 (t, J = 7.9 Hz, 1H), 7.33 – 7.23 (m, 2H), 7.12 (ddd, J = 8.2, 2.7, 1.0 Hz, 1H), 6.92 – 6.82 (m, 2H), 5.12 (t, J = 6.3 Hz, 1H), 3.87 (s, 3H), 3.17 (q, J = 6.6 Hz, 2H), 2.57 – 2.46 (m, 2H), 2.41 (s, 3H); $^{13}$C NMR (101 MHz, $CDCl_3$) δ 189.8, 159.8, 144.4, 143.5, 138.8, 136.8, 129.8, 129.5, 128.0, 127.0, 121.2, 119.5, 112.8, 55.4, 41.6, 32.8, 21.5; HRMS (ESI) calcd for $C_{19}H_{21}NO_3SH^+$ $[M+H]^+$ 344.1315, found 344.1312.

**(*E*)-*N*-(5-(2-Methoxyphenyl)-5-oxopent-3-en-1-yl)-4-methylbenzenesulfonamide (1k)**

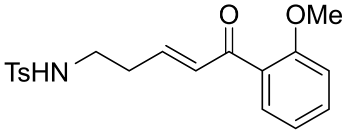


Prepared according to the general procedure as a white solid. $^1$H NMR (300 MHz, Chloroform-d) δ 7.75 – 7.68 (m, 2H), 7.53 – 7.38 (m, 2H), 7.27 – 7.20 (m, 2H), 7.02 – 6.89 (m, 2H), 6.77 – 6.55 (m, 2H), 5.33 (t, J = 6.2 Hz, 1H), 3.83 (s, 3H), 3.07 (q, J = 6.6 Hz, 2H), 2.43 – 2.33 (m, 5H); $^{13}$C NMR (75 MHz, $CDCl_3$) δ 192.4, 157.7, 143.1, 142.6, 136.5, 132.7, 132.3, 129.9, 129.3, 128.2, 126.7, 120.3, 111.5, 55.3, 41.2, 32.1, 21.1; HRMS (ESI) calcd for $C_{19}H_{21}NO_4SH^+$ $[M+H]^+$ 360.1264, found 360.1259.

**(*E*)-*N*-(5-(3-Methoxyphenyl)-5-oxopent-3-en-1-yl)-4-methylbenzenesulfonamide (1l)**

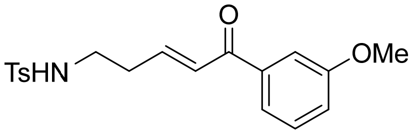


Prepared according to the general procedure as a white solid. $^{1}$H NMR (400 MHz, Chloroform-d) δ 7.84 – 7.80 (m, 2H), 7.78 – 7.73 (m, 2H), 7.30 – 7.25 (m, 4H), 6.96 – 6.77 (m, 2H), 5.15 (t, J = 6.3 Hz, 1H), 3.17 (q, J = 6.6 Hz, 2H), 2.51 (td, J = 6.8, 5.8 Hz, 2H), 2.42 (d, J = 5.6 Hz, 6H). $^{13}$C NMR (101 MHz, $CDCl_3$) δ 189.7, 143.9, 143.8, 143.5, 136.8, 134.8, 129.8, 129.3, 128.7, 128.0, 127.0, 41.7, 32.8, 21.6, 21.5; HRMS (ESI) calcd for $C_{19}H_{21}NO_4SH^+$ $[M+H]^+$ 360.1264, found 360.1259.

**(*E*)-4-Methyl-*N*-(5-(4-nitrophenyl)-5-oxopent-3-en-1-yl)benzenesulfonamide (1m)**

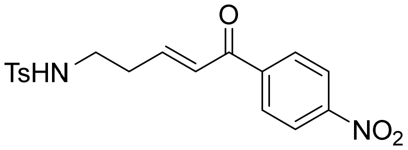


Prepared according to the general procedure as a white solid. $^{1}$H NMR (400 MHz, Chloroform-d) δ 8.38 – 8.25 (m, 2H), 8.09 – 8.03 (m, 2H), 7.81 – 7.70 (m, 2H), 7.34 – 7.29 (m, 2H), 7.04 – 6.80 (m, 2H), 5.18 (t, J = 6.3 Hz, 1H), 3.19 (q, J = 6.5 Hz, 2H), 2.56 (q, J = 6.3 Hz, 2H), 2.43 (s, 3H). $^{13}$C NMR (101 MHz, $CDCl_3$) δ 188.7, 150.0, 146.6, 143.7, 142.2, 136.6, 129.8, 129.7, 129.5, 127.7, 127.0, 123.7, 41.4, 32.9, 21.5; HRMS (ESI) calcd for $C_{18}H_{18}N_2O_5SH^+$ $[M+H]^+$ 375.1009, found 375.1006.

**(*E*)-4-Methyl-*N*-(5-oxo-5-(thiophen-2-yl)pent-3-en-1-yl)benzenesulfonamide (1n)**

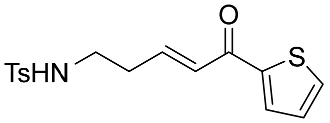


Prepared according to the general procedure as a pale yellow solid. $^{1}$H NMR (400 MHz, Chloroform-d) δ 7.76 (d, J = 1.8 Hz, 1H), 7.76 – 7.73 (m, 2H), 7.66 (dd, J = 4.9, 1.1 Hz, 1H), 7.32 – 7.24 (m, 2H), 7.14 (dd, J = 4.9, 3.8 Hz, 1H), 6.98 – 6.77 (m, 2H), 5.25 (t, J = 6.2 Hz, 1H), 3.16 (q, J = 6.6 Hz, 2H), 2.53 – 2.48 (m, 2H), 2.41 (s, 3H); $^{13}$C NMR (101 MHz, $CDCl_3$) δ 181.8, 144.7, 143.8, 143.5, 136.8, 134.2, 132.4, 129.7, 128.3, 127.5, 127.0, 41.6, 32.7, 21.5; HRMS (ESI) calcd for $C_{16}H_{17}NO_3S_2H^+$ $[M+H]^+$ 336.0723, found 336.0720.

**(*E*)-4-Methyl-*N*-(5-(naphthalen-2-yl)-5-oxopent-3-en-1-yl)benzenesulfonamide (1o)**

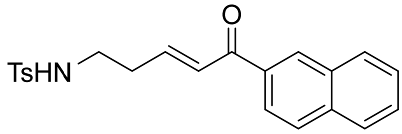


Prepared according to the general procedure as a white solid. $^1$H NMR (400 MHz, Chloroform-d) δ 8.43 (d, J = 1.6 Hz, 1H), 7.99 – 7.93(m, 2H), 7.91 – 7.84 (m, 2H), 7.79 – 7.74 (m, 2H), 7.68 – 7.56 (m, 2H), 7.28 – 7.23 (m, 2H), 7.12 – 7.06 (m, 1H), 6.96 – 6.92 (m, 1H), 5.30 (t, J = 6.2 Hz, 1H), 3.20 (q, J = 6.6 Hz, 2H), 2.58 – 2.54 (m, 2H), 2.37 (s, 3H); $^{13}$C NMR (101 MHz, $CDCl_3$) δ 189.9, 144.3, 143.5, 136.8, 135.5, 134.7, 132.4, 130.3, 129.8, 129.6, 128.5, 128.4, 128.0, 127.7, 127.0, 126.7, 124.3, 41.7, 32.9, 21.4; HRMS (ESI) calcd for $C_{22}H_{21}NO_3SH^+$ $[M+H]^+$ 380.1315, found 380.1314.

**(*E*)-4-Nitro-*N*-(5-oxo-5-phenylpent-3-en-1-yl)benzenesulfonamide (1p)**

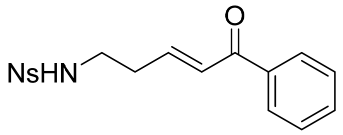


Prepared according to the general procedure as a white solid. $^1$H NMR (400 MHz, Chloroform-d) δ 8.67 (t, J = 2.0 Hz, 1H), 8.36 (ddd, J = 8.2, 2.3, 1.0 Hz, 1H), 8.19 (dt, J = 7.8, 1.3 Hz, 1H), 7.92 – 7.80 (m, 2H), 7.71 (t, J = 8.0 Hz, 1H), 7.60 – 7.52 (m, 1H), 7.44 (dd, J = 8.4, 7.0 Hz, 2H), 7.03 – 6.78 (m, 2H), 5.92 (t, J = 6.2 Hz, 1H), 3.26 (q, J = 6.5 Hz, 2H), 2.57 (q, J = 6.4 Hz, 2H). $^{13}$C NMR (101 MHz, $CDCl_3$) δ 190.3, 148.2, 144.4, 142.1, 137.1, 133.1, 132.5, 130.6, 128.6, 128.5, 128.1, 127.1, 122.1, 41.8, 32.9; HRMS (ESI) calcd for $C_{17}H_{16}N_2O_5SH^+$ $[M+H]^+$ 361.0853, found 361.0851.

***tert*-Butyl (*E*)-(5-oxo-5-phenylpent-3-en-1-yl)carbamate (1q)**

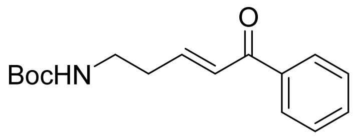


Prepared according to the general procedure as a colorless oil. $^1$H NMR (400 MHz, Chloroform-d) δ 7.91 (d, J = 9.0 Hz, 2H), 7.54 (s, 1H), 7.46 (s, 2H), 6.96 (d, J = 10.7 Hz, 2H), 4.71 (s, 1H), 3.32 (d, J = 5.6 Hz, 2H), 2.51 (d, J = 8.3 Hz, 2H), 1.41 (s, 9H); $^{13}$C NMR (101 MHz, Chloroform-d) δ 190.5, 155.9, 145.8, 137.7, 132.9, 128.6, 127.7, 79.5, 39.1, 33.4, 28.4; HRMS (ESI) calcd for $C_{16}H_{21}NO_3SH^+$ $[M+H]^+$ 298.1414, found 298.1412.

**Ethyl (*E*)-5-((4-methylphenyl)sulfonamido)pent-2-enoate (1r)**

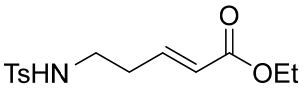

Prepared according to a literature procedure.

**(*E*)-4-Methyl-N-(5-oxohex-3-en-1-yl)benzenesulfonamide (1s)**

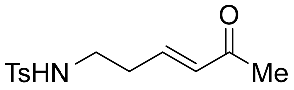

Prepared according to the general procedure as a pale yellow solid. $^1$H NMR (300 MHz, Chloroform-d) δ 7.88 – 7.61 (m, 2H), 7.30 (d, J = 8.0 Hz, 2H), 6.65 (dt, J = 16.0, 6.9 Hz, 1H), 6.02 (dt, J = 16.0, 1.5 Hz, 1H), 5.35 (t, J = 6.3 Hz, 1H), 3.09 (q, J = 6.6 Hz, 2H), 2.45 – 2.35 (m, 5H), 2.19 (s, 3H). $^{13}$C NMR (75 MHz, $CDCl_3$) δ 198.0, 143.2, 143.0, 136.4, 132.8, 129.4, 126.7, 41.2, 32.1, 26.6, 21.1; HRMS (ESI) calcd for $C_{13}H_{17}NO_3SH^+$ $[M+H]^+$ 268.1002, found 268.0998.

**General procedure for preparation of *N*-Sulfonylimine 2**[2]

*N*-Sulfonylimines were prepared according to the literature.

**General Procedure for Achiral Phosphine-Catalyzed [5+1] Annulation**

Under a nitrogen atmosphere, to a stirred solution of δ-sulfonamido-substituted enones **1** (0.2 mmol, 1.0 equiv) and *N*-sulfonylimine **2** (0.3 mmol, 1.5 equiv) in DCM (2 mL) was added catalyst $PBu_3$ (0.04 mmol, 20 mol%) via a syringe. Then the reaction solution was vigorously stirred at room temperature and monitored by TLC. After the reaction was complete, the mixture was directly purified by column chromatography on silica gel (petroleum ether/EtOAc as the eluent) to furnish the corresponding product.

**General Procedure for Chiral Phosphine-Catalyzed Asymmetric [5+1] Annulation**

Under a nitrogen atmosphere, to a mixture of δ-sulfonamido-substituted enone **1** (0.1 mmol, 1.0 equiv), *N*-sulfonylimine 2 (0.15 mmol, 1.5 equiv) and catalyst $P^*$ (0.02 mmol, 20 mol%) was added $CF_3Ph$ (1 mL) via a syringe. Then the reaction solution was vigorously stirred at -10°C and monitored by TLC. After the reaction was complete, the mixture was directly purified by column chromatography on silica gel (Petroleum Ether/EtOAc as the eluent) to furnish the corresponding product.

[2] M. K. Ghorai, S. Das, K. Das, A. Kumar. *Org. Biomol. Chem.* **2015**, *13*, 9042–9049.

**Characterization Data for the [5+1] Annulation Products 3**

**Phenyl (2-phenyl-1-tosyl-1,2,5,6-tetrahydropyridin-3-yl)methanone (3aa)**

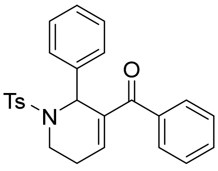

**3aa**

Prepared according to the general procedure as described above in 95% yield. It was purified by flash chromatography (15% EtOAc/PE) to afford a white solid. mp = 161 – 163 °C; $^1$H NMR (300 MHz, $CDCl_3$) δ 7.77 – 7.67 (m, 2H), 7.64 – 7.57 (m, 2H), 7.55 – 7.48 (m, 1H), 7.47 – 7.34 (m, 4H), 7.33 – 7.25 (m, 3H), 7.24 – 7.18 (m, 2H), 6.64 – 6.52 (m, 1H), 6.29 (s, 1H), 3.94 – 3.86 (m, 1H), 3.31 – 3.04 (m, 1H), 2.38 (s, 3H), 2.25 – 2.18 (m, 2H); $^{13}$C NMR (75 MHz, $CDCl_3$) δ 192.5, 143.0, 141.3, 138.2, 137.5, 137.3, 135.1, 130.3, 129.2, 128.3, 128.2, 127.8, 127.6, 126.8, 54.2, 36.9, 24.0, 21.1; IR (film) $\nu_{max}$ 3061, 2927, 1645, 1597, 1493, 1447, 1342, 1275, 1212, 1161, 1091, 962, 891, 815, 718, 699, 671, 653, 574, 548 cm$^{-1}$; HRMS (ESI) calcd for $C_{25}H_{23}NO_3SH^+$ $[M+H]^+$ 418.1471, found 418.1470.

**(2-(2-Fluorophenyl)-1-tosyl-1,2,5,6-tetrahydropyridin-3-yl)(phenyl)methanone (3ab)**

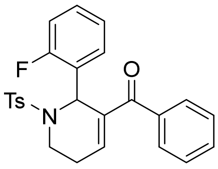

**3ab**

Prepared according to the general procedure as described above in 96% yield. It was purified by flash chromatography (15% EtOAc/PE) to afford a white solid. mp = 66 – 68 °C; $^1$H NMR (300 MHz, $CDCl_3$) 7.73 – 7.64 (m, 2H), 7.62 – 7.55 (m, 2H), 7.54 – 7.46 (m, 1H), 7.44 – 7.34 (m, 3H), 7.25 – 7.15 (m, 3H), 7.09 – 7.04 (m, 1H),7.00 – 6.93 (m, 1H), 6.68 – 6.68 (m, 1H), 6.41 (s, 1H), 3.89 (dd, $J$ = 14.7, 6.5 Hz, 1H), 3.35 – 3.25 (m, 1H), 2.49 – 2.23 (m, 5H); $^{13}$C NMR (75 MHz, $CDCl_3$) δ 193.7 , 160.6 (d, $J$ = 249.2 Hz), 143.0, 141.0, 137.1 (d, $J$ = 3.6 Hz), 137.0, 131.7, 130.6 (d, $J$ = 3.6 Hz), 129.7, 129.5, 129.1, 128.9, 127.9, 126.9, 125.4 (d, $J$ = 13.4 Hz), 123.6 (d, $J$ = 3.5 Hz), 115.9 (d, $J$ = 22.5 Hz), 50.3, 37.5 (d, $J$ = 2.6 Hz), 24.2, 21.1; IR (film) $\nu_{max}$ 2924, 1645, 1597, 1511, 1447, 1342, 1275, 1212, 1160, 1091, 963, 893, 815, 713, 668, 652, 581, 550 cm$^{-1}$; HRMS (ESI) calcd for $C_{25}H_{22}FNO_3SH^+$ $[M+H]^+$ 436.1377, found 436.1378.

**(2-(3-Fluorophenyl)-1-tosyl-1,2,5,6-tetrahydropyridin-3-yl)(phenyl)methanone (3ac)**

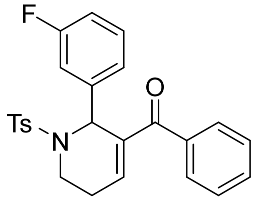

**3ac**

Prepared according to the general procedure as described above in 85% yield. It was purified by flash chromatography (15% EtOAc/PE) to afford a white solid. mp = 180 – 182 °C; $^{1}$H NMR (300 MHz, $CDCl_3$) δ 7.79 – 7.68 (m, 2H), 7.61 – 7.48 (m, 3H), 7.44 – 7.40 (m, 2H), 7.33 – 7.20 (m, 3H), 7.19 – 7.12 (m, 1H), 7.09 – 7.05 (m, 1H), 6.97 – 6.93 (m, 1H), 6.65 – 6.62 (m, 1H), 6.25 (s, 1H), 4.02 – 3.80 (m, 1H), 3.16 – 3.10 (m, 1H), 2.38 (s, 3H), 2.32 – 2.15 (m, 2H); $^{13}$C NMR (75 MHz, $CDCl_3$) δ 193.7, 162.4 (d, *J* = 246.4 Hz) , 143.2, 142.0, 137.4, 136.8 (d, *J* = 10.6 Hz) , 131.9, 129.6 (d, *J* = 8.2 Hz), 129.3, 128.8, 128.0, 126.8, 123.6 (d, *J* = 2.8 Hz),114.9 (d, *J* = 22.2 Hz) , 114.5 (d, *J* = 20.9 Hz), 53.8 (d, *J* = 2.0 Hz) , 37.0, 23.8, 21.1; IR (film) $\nu_{max}$ 1644, 1591, 1486, 1446, 1343, 1274, 1161, 1091, 980, 885, 816, 765, 713, 670, 655, 555 cm$^{-1}$; HRMS (ESI) calcd for $C_{25}H_{22}FNO_3SH^+$ $[M+H]^+$ 436.1377, found 436.1378.

**(2-(4-Fluorophenyl)-1-tosyl-1,2,5,6-tetrahydropyridin-3-yl)(phenyl)methanone (3ad)**

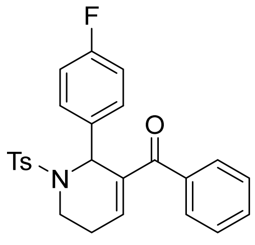

**3ad**

Prepared according to the general procedure as described above in 93% yield. It was purified by flash chromatography (15% EtOAc/PE) to afford a white solid. mp = 176 – 178 °C; $^{1}$H NMR (300 MHz, $CDCl_3$) δ 7.89 – 7.67 (m, 2H), 7.62 – 7.47 (m, 3H), 7.41 – 7.35 (m, 4H), 7.22 (d, *J* = 8.2 Hz, 2H), 7.03 – 6.87 (m, 2H), 6.67 – 6.48 (m, 1H), 6.25 (s, 1H), 4.06 – 3.71 (m, 1H), 3.15 – 3.09 (m, 1H), 2.38 (s, 3H), 2.24 – 2.18 (m, 2H); $^{13}$C NMR (75 MHz, $CDCl_3$) δ 193.7, 162.0 (d, *J* = 247.0 Hz), 143.1, 141.8, 137.5, 137.1, 136.7, 134.4 (d, *J* = 3.3 Hz), 131.9, 129.6 (d, *J* = 8.3 Hz), 129.3, 128.8, 128.0, 126.7, 115.0 (d, *J* = 21.7 Hz) , 53.6, 36.8, 23.8, 21.1; IR (film) $\nu_{max}$ 2927, 1644, 1598, 1507, 1447, 1342, 1275, 1224, 1160, 1091, 963, 893, 815, 773, 751, 713, 669, 652, 580, 550 cm$^{-1}$; HRMS (ESI) calcd for $C_{25}H_{22}FNO_3SH^+$ $[M+H]^+$ 436.1377, found 436.1378.

**(2-(2-Chlorophenyl)-1-tosyl-1,2,5,6-tetrahydropyridin-3-yl)(phenyl)methanone (3ae)**

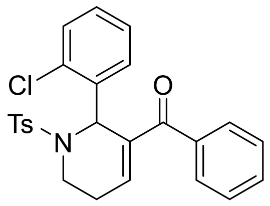

**3ae**

Prepared according to the general procedure as described above in 75% yield. It was purified by flash chromatography (15% EtOAc/PE) to afford a white solid. mp = 172 – 174 °C; $^1$H NMR (300 MHz, $CDCl_3$) δ 7.84 – 7.70 (m, 2H), 7.64 – 7.46 (m, 4H), 7.44 – 7.34 (m, 2H), 7.29 (dd, J = 7.7, 1.9 Hz, 1H), 7.26 – 7.18 (m, 3H), 7.12 – 7.10 (m, J = 7.6, 1.9 Hz, 1H), 6.76 (t, J = 4.0 Hz, 1H), 6.59 (s, 1H), 3.76 (dd, J = 14.9, 7.1 Hz, 1H), 3.38 – 3.33 (m, 1H), 2.73 – 2.49 (m, 1H), 2.39 (s, 3H), 2.37 – 2.22 (m, 1H); $^{13}$C NMR (75 MHz, $CDCl_3$) δ 193.7, 143.1, 141.5, 137.4, 137.0, 136.9, 135.6, 134.6, 131.6, 130.5, 129.4, 129.1, 129.0, 128.8, 127.9, 127.3, 125.9, 52.0, 37.3, 24.0, 21.2; IR (film) $\nu_{max}$ 2924, 1645, 1597, 1446, 1346, 1273, 1162, 1090, 1039, 959, 886, 816, 733, 713, 698, 670, 652, 550 cm$^{-1}$; HRMS (ESI) calcd for $C_{25}H_{22}ClNO_3SH^+$ $[M+H]^+$ 452.1082, found 452.1082.

**(2-(3-Chlorophenyl)-1-tosyl-1,2,5,6-tetrahydropyridin-3-yl)(phenyl)methanone (3af)**

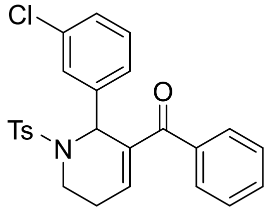

**3af**

Prepared according to the general procedure as described above in 82% yield. It was purified by flash chromatography (15% EtOAc/PE) to afford a white solid. mp = 172 – 174 °C; $^1$H NMR (300 MHz, $CDCl_3$) δ 7.76 – 7.67 (m, 2H), 7.55 – 7.42 (m, 3H), 7.41 – 7.29 (m, 5H), 7.29 – 7.21 (m, 3H), 6.72 – 6.55 (m, 1H), 6.23 (s, 1H), 3.97 – 3.80 (m, 1H), 3.17 – 3.11 (m, 1H), 2.40 (s, 3H), 2.26 – 2.22 (m, 2H); $^{13}$CNMR (75 MHz, $CDCl_3$) δ 193.7, 143.2, 142.2, 140.6, 137.4, 136.7, 134.0, 131.9, 129.4, 129.3, 128.8, 128.0, 127.8, 126.8, 126.1, 53.8, 37.0, 23.9, 21.1; IR (film) $\nu_{max}$ 2926, 1644, 1596, 1447, 1343, 1274, 1161, 1091, 967, 794, 746, 697, 670, 654, 553 cm$^{-1}$; HRMS (ESI) calcd for $C_{25}H_{22}ClNO_3SH^+$ $[M+H]^+$ 452.1082, found 452.1082.

**(2-(4-Chlorophenyl)-1-tosyl-1,2,5,6-tetrahydropyridin-3-yl)(phenyl)methanone (3ag)**

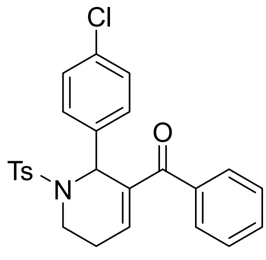

**3ag**

Prepared according to the general procedure as described above in 85% yield. It was purified by flash chromatography (15% EtOAc/PE) to afford a white solid. mp = 136 – 138 °C; $^1$H NMR (300 MHz, $CDCl_3$) δ 7.84 – 7.62 (m, 2H), 7.62 – 7.51 (m, 2H), 7.43 – 7.36 (m, 2H), 7.36 – 7.25 (m, 5H), 7.25 – 7.17 (m, 2H), 6.61 – 6.57 (m, 1H), 6.25 (s, 1H), 3.92– 3.90 (m, 1H), 3.17 – 3.14 (m, 1H), 2.38 (s, 3H), 2.34 – 2.14 (m, 2H); $^{13}$C NMR (75 MHz, $CDCl_3$) δ 193.7, 143.2, 141.9, 137.4, 137.1, 136.9, 136.7, 133.5, 131.9, 129.3, 129.3, 128.8, 128.3, 128.0, 126.7, 53.7, 36.9, 23.8, 21.1; IR (film) $\nu_{max}$ 2927, 1644, 1597, 1489, 1447, 1343, 1274, 1212, 1161, 1090, 1016, 963, 892, 815, 751, 711, 651, 576, 548 cm$^{-1}$; HRMS (ESI) calcd for $C_{25}H_{22}ClNO_3SH^+$ $[M+H]^+$ 452.1082, found 452.1082.

**(2-(2-Bromophenyl)-1-tosyl-1,2,5,6-tetrahydropyridin-3-yl)(phenyl)methanone (3ah)**

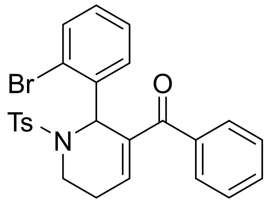

**3ah**

Prepared according to the general procedure as described above in 68% yield. It was purified by flash chromatography (15% EtOAc/PE) to afford a white solid. mp = 184 – 186 °C; $^1$H NMR (300 MHz, $CDCl_3$) δ 7.84 – 7.70 (m, 2H), 7.64 – 7.46 (m, 4H), 7.44 – 7.34 (m, 2H), 7.32 – 7.26 (m, 1H), 7.26 – 7.18 (m, 3H), 7.13 – 7.09 (m, 1H), 6.76 (t, $J$ = 4.0 Hz, 1H), 6.59 (s, 1H), 3.78 – 3.74 (m, 1H), 3.40 – 3.31 (m, 1H), 2.73 – 2.49 (m, 1H), 2.39 (s, 4H); $^{13}$CNMR (75 MHz, $CDCl_3$) δ 193.7, 143.1, 141.1, 137.7, 137.3, 137.0, 136.8, 134.0, 131.6, 129.4, 129.2, 129.1, 128.8, 127.9, 127.4, 126.5, 125.0, 54.3, 37.4, 24.0, 21.2; IR (film) $\nu_{max}$ 3060, 2960, 1644, 1597, 1467, 1446, 1346, 1273, 1160, 1090, 1026, 958, 712, 699, 652, 550 cm$^{-1}$; HRMS (ESI) calcd for $C_{25}H_{22}BrNO_3SH^+$ $[M+H]^+$ 496.0577, found 496.0576.

**(2-(3-Bromophenyl)-1-tosyl-1,2,5,6-tetrahydropyridin-3-yl)(phenyl)methanone (3ai)**

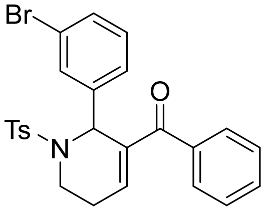

**3ai**

Prepared according to the general procedure as described above in 81% yield. It was purified by flash chromatography (15% EtOAc/PE) to afford a white solid. mp = 157 – 160 °C; $^1$H NMR (300 MHz, $CDCl_3$) δ 7.75 – 7.67 (m, 2H), 7.61 – 7.49 (m, 3H), 7.47 – 7.34 (m, 4H), 7.29 – 7.17 (m, 4H), 6.65 (t, *J* = 3.9 Hz, 1H), 6.20 (s, 1H), 4.08 – 3.81 (m, 1H), 3.14 – 3.10 (m, 1H), 2.39 (s, 3H), 2.33 – 2.19 (m, 2H); $^{13}$C NMR (75 MHz, CDCl3) δ 193.6, 143.2, 142.1, 140.8, 137.3, 136.7, 131.9, 130.9, 130.7, 129.7, 129.3, 128.8, 128.0, 126.8, 126.6, 122.3, 53.7, 37.0, 23.9, 21.2; IR (film) $\nu_{max}$ 2925, 1645, 1597, 1447, 1343, 1274, 1161, 1091, 966, 795, 728, 696, 666, 653, 552 cm$^{-1}$; HRMS (ESI) calcd for $C_{25}H_{22}BrNO_3SH^+$ $[M+H]^+$ 496.0577, found 496.0576.

**(2-(4-Bromophenyl)-1-tosyl-1,2,5,6-tetrahydropyridin-3-yl)(phenyl)methanone (3aj)**

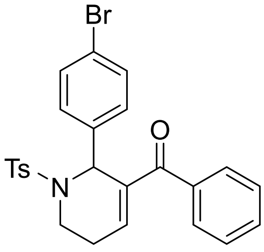

**3aj**

Prepared according to the general procedure as described above in 84% yield. It was purified by flash chromatography (15% EtOAc/PE) to afford a white solid. mp = 142 – 144 °C; $^1$H NMR (300 MHz, $CDCl_3$) δ 7.75 – 7.65 (m, 2H), 7.60 – 7.48 (m, 3H), 7.46 – 7.37 (m, 4H), 7.27 –7.21 (m, 4H), 6.63 (t, *J* = 3.9 Hz, 1H), 6.20 (s, 1H), 4.11 – 3.82 (m, 1H), 3.17 – 3.12 (m, 1H), 2.39 (s, 3H), 2.28 – 2.07 (m, 2H); $^{13}$C NMR (75 MHz, $CDCl_3$) δ 193.7, 143.2, 141.9, 137.6, 137.4, 136.8, 136.7, 131.9, 131.3, 129.6, 129.3, 128.8, 128.0, 126.7, 121.7, 53.8, 37.0, 23.8, 21.2; IR (film) $\nu_{max}$ 2926, 1644, 1597, 1486, 1447, 1342, 1274, 1161, 1091, 1071, 1011, 963, 892, 815, 710, 674, 651, 575, 547 cm$^{-1}$; HRMS (ESI) calcd for $C_{25}H_{22}BrNO_3SH^+$ $[M+H]^+$ 496.0577, found 496.0577.

**Phenyl(2-(*o*-tolyl)-1-tosyl-1,2,5,6-tetrahydropyridin-3-yl)methanone (3ak)**

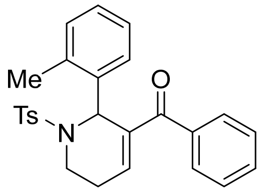


**3ak**

Prepared according to the general procedure as described above in 41% yield. It was purified by flash chromatography (15% EtOAc/PE) to afford a white solid. mp = 143 – 145 °C; $^1$H NMR (300 MHz, $CDCl_3$) δ 7.80 – 7.68 (m, 2H), 7.56 – 7.48 (m, 3H), 7.46 – 7.38 (m, 2H), 7.29 – 7.08 (m, 6H), 6.57 (t, *J* = 3.9 Hz, 1H), 6.52 (s, 1H), 3.88 (dd, *J* = 15.2, 6.5 Hz, 1H), 3.50 – 3.28 (m, 1H), 2.75 (s, 3H), 2.40 (s, 3H), 2.36 – 2.17 (m, 2H); $^{13}$C NMR (75 MHz, $CDCl_3$) δ 193.8, 143.1, 140.6, 138.3, 137.9, 137.1, 137.1, 135.7, 131.6, 131.3, 129.1, 128.7, 127.9, 127.8, 127.3, 127.1, 124.9, 51.3, 36.9, 23.3, 21.1, 19.6; IR (film) $\nu_{max}$ 2924, 1643, 1597, 1447, 1342, 1274, 1212, 1160, 1090, 958, 886, 815, 742, 712, 670, 653, 579, 551 cm$^{-1}$; HRMS (ESI) calcd for $C_{26}H_{25}NO_3SH^+$ $[M+H]^+$ 432.1628, found 432.1624.

**Phenyl(2-(*m*-tolyl)-1-tosyl-1,2,5,6-tetrahydropyridin-3-yl)methanone (3al)**

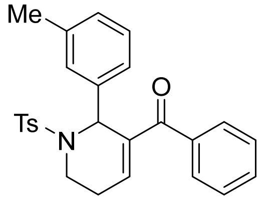


**3al**

Prepared according to the general procedure as described above in 79% yield. It was purified by flash chromatography (15% EtOAc/PE) to afford a white solid. mp = 146 – 149 °C; $^1$H NMR (300 MHz, $CDCl_3$) δ 7.77 – 7.70 (m, 2H), 7.63 – 7.56 (m, 2H), 7.56 – 7.48 (m, 1H), 7.46 – 7.37 (m, 2H), 7.25 – 7.01 (m, 6H), 6.59 (t, *J* = 4.0 Hz, 1H), 6.24 (s, 1H), 3.92 (dd, *J* = 14.9, 6.0 Hz, 1H), 3.28 – 3.09 (m, 1H), 2.38 (s, 3H), 2.30 (s, 3H), 2.28 – 2.10 (m, 2H); $^{13}$C NMR (75 MHz, $CDCl_3$) δ 165.1, 148.8, 144.9, 141.9, 138.9, 135.6, 134.8, 134.0, 133.3, 130.0, 129.5, 129.2, 128.7, 127.7, 125.9, 125.6, 125.3, 124.6, 61.5, 61.1, 57.3, 38.4, 21.5, 13.9; IR (film) $\nu_{max}$ 2922, 1645, 1597, 1447, 1368, 1342, 1275, 1161, 1091, 1019, 801, 761, 744, 713, 671, 654, 552 cm$^{-1}$; HRMS (ESI) calcd for $C_{26}H_{25}NO_3SH^+$ $[M+H]^+$ 432.1628, found 432.1626.

**Phenyl(2-(*p*-tolyl)-1-tosyl-1,2,5,6-tetrahydropyridin-3-yl)methanone (3am)**

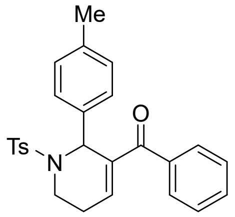


**3am**

Prepared according to the general procedure as described above in 95% yield. It was purified by flash chromatography (15% EtOAc/PE) to afford a white solid. mp = 170 – 173 °C; $^{1}H$ NMR (300 MHz, $CDCl_3$) δ 7.82 – 7.66 (m, 2H), 7.64 – 7.56 (m, 2H), 7.56 – 7.47 (m, 1H), 7.46 – 7.35 (m, 2H), 7.27 (s, 1H), 7.25 (d, *J* = 2.1 Hz, 1H), 7.23 – 7.18 (m, 2H), 7.10 (d, *J* = 7.9 Hz, 2H), 6.58 (t, *J* = 3.9 Hz, 1H), 6.25 (s, 1H), 4.06 – 3.72 (m, 1H), 3.27 – 3.10 (m, 1H), 2.38 (s, 3H), 2.30 (s, 3H), 2.26 – 2.13 (m, 2H); $^{13}C$ NMR (75 MHz, $CDCl_3$) δ 193.8, 142.9, 141.3, 137.7, 137.4, 137.3, 136.9, 135.4, 131.7, 129.2, 128.9, 128.8, 127.9, 127.8, 126.8, 54.0, 36.9, 23.9, 21.1, 20.7; IR (film) $\nu_{max}$ 2926, 1644, 1597, 1489, 1447, 1345, 1274, 1162, 1091, 961, 891, 815, 763, 713, 670, 652, 578, 552 $cm^{-1}$; HRMS (ESI) calcd for $C_{26}H_{25}NO_3SH^+$ $[M+H]^+$ 432.1628, found 432.1626.

**(2-(3-Methoxyphenyl)-1-tosyl-1,2,5,6-tetrahydropyridin-3-yl)(phenyl)methanone (3an)**

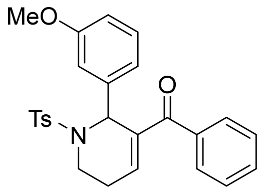


**3an**

Prepared according to the general procedure as described above in 90% yield. It was purified by flash chromatography (15% EtOAc/PE) to afford a white solid. mp = 106 – 108 °C; $^{1}H$ NMR (300 MHz, $CDCl_3$) δ $^{1}H$ NMR (300 MHz, $CDCl_3$) δ 7.78 – 7.68 (m, 2H), 7.65 – 7.57 (m, 2H), 7.56 – 7.46 (m, 1H), 7.46 – 7.35 (m, 2H), 7.25 – 7.13 (m, 3H), 7.01 – 6.89 (m, 2H), 6.87 – 6.71 (m, 1H), 6.59 (t, *J* = 3.9 Hz, 1H), 6.26 (s, 1H), 3.98 – 3.82 (m, 1H), 3.75 (s, 3H), 3.29 – 3.08 (m, 1H), 2.37 (s, 3H), 2.29 – 2.12 (m, 2H); $^{13}C$ NMR (75 MHz, $CDCl_3$) δ193.7, 159.4, 143.0, 141.5, 140.0, 137.6, 137.2, 136.8, 131.8, 129.2, 129.0, 128.9, 128.0, 126.8, 120.2, 113.7, 113.2, 54.8, 54.1, 37.0, 23.9, 21.1; IR (film) $\nu_{max}$ 2933, 1644, 1598, 1488, 1447, 1342, 1275, 1161, 1091, 1048, 970, 884, 815, 762, 713, 670, 658, 557 $cm^{-1}$; HRMS (ESI) calcd for $C_{26}H_{25}NO_4SH^+$ $[M+H]^+$ 448.1577, found 448.1576.

**(2-(4-Methoxyphenyl)-1-tosyl-1,2,5,6-tetrahydropyridin-3-yl)(phenyl)methanone (3ao)**

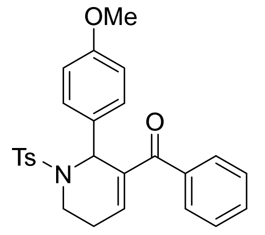


**3ao**

Prepared according to the general procedure as described above in 98% yield. It was purified by flash chromatography (15% EtOAc/PE) to afford a white solid. mp = 112 – 115 °C; $^1$H NMR (300 MHz, $CDCl_3$) δ 7.71 (d, *J* = 8.3 Hz, 2H), 7.63 – 7.55 (m, 2H), 7.55 – 7.47 (m, 1H), 7.46 – 7.34 (m, 2H), 7.30 – 7.26 (m, 2H), 7.24 – 7.16 (m, 2H), 6.85 – 6.77 (m, 2H), 6.65 – 6.43 (m, 1H), 6.22 (s, 1H), 3.97 – 3.81 (m, 1H), 3.75 (s, 3H), 3.26 – 3.03 (m, 1H), 2.37 (s, 3H), 2.28 – 2.12 (m, 2H); $^{13}$C NMR (75 MHz, $CDCl_3$) δ 193.8, 158.9, 143.0, 141.2, 137.6, 137.5, 136.9, 131.8, 130.5, 129.3, 129.2, 129.1, 128.9, 127.9, 126.8, 126.0, 113.5, 54.9, 53.8, 36.8, 23.9, 21.1; IR (film) $\nu_{max}$ 2932, 2360, 1643, 1597, 1511, 1446, 1340, 1305, 1276, 1250, 1160, 1090, 1031, 984, 962, 892, 815, 752, 713, 668, 652, 552 cm$^{-1}$; HRMS (ESI) calcd for $C_{26}H_{25}NO_4SH^+$ $[M+H]^+$ 448.1577, found 448.1576.

**Phenyl(2-(thiophen-2-yl)-1-tosyl-1,2,5,6-tetrahydropyridin-3-yl)methanone (3ap)**

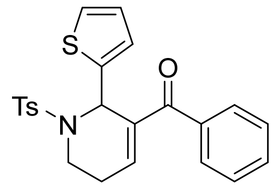


**3ap**

Prepared according to the general procedure as described above in 95% yield. It was purified by flash chromatography (15% EtOAc/PE) to afford a white solid. mp = 116 – 118 °C; $^1$H NMR (300 MHz, $CDCl_3$) δ 7.74 – 7.60 (m, 4H), 7.58 – 7.48 (m, 1H), 7.48 – 4.39 (m, 2H), 7.25 – 7.11 (m, 3H), 7.01 – 6.95 (m, 1H), 6.91 – 6.87 (m, 1H), 6.54 (d, *J* = 3.1 Hz, 2H), 4.03 – 3.86 (m, 1H), 3.45 – 3.26 (m, 1H), 2.37 (s, 3H), 2.32 – 2.19 (m, 2H); $^{13}$C NMR (75 MHz, $CDCl_3$) δ 193.5, 143.0, 141.7, 140.3, 138.0, 137.3, 136.8, 131.9, 129.2, 129.0, 128.0, 126.9, 126.8, 126.5, 125.2, 50.2, 37.1, 24.2, 21.2; IR (film) $\nu_{max}$ 2926, 1645, 1597, 1447, 1342, 1271, 1212, 1161, 1091, 991, 957, 884, 815, 708, 655, 562 cm$^{-1}$; HRMS (ESI) calcd for $C_{23}H_{21}NO_3S_2H^+$ $[M+H]^+$ 424.1036, found 424.1034.

**(2-(Naphthalen-2-yl)-1-tosyl-1,2,5,6-tetrahydropyridin-3-yl)(phenyl)methanone (3aq)**

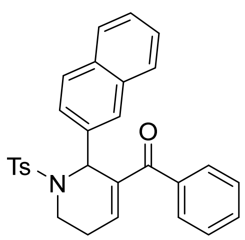


**3aq**

Prepared according to the general procedure as described above in 98% yield. It was purified by flash chromatography (15% EtOAc/PE) to afford a white solid. mp = 165 – 167 °C; $^1$H NMR (300 MHz, $CDCl_3$) δ $^1$H NMR (300 MHz, $CDCl_3$) δ 7.87 – 7.72 (m, 5H), 7.69 – 7.59 (m, 4H), 7.58 – 7.50 (m, 1H), 7.50 – 7.39 (m, 4H), 7.24 – 7.14 (m, 2H), 6.68 (t, *J* = 3.9 Hz, 1H), 6.44 (s, 1H), 4.02 – 3.83 (m, 1H), 3.25 – 3.13 (m, 1H), 2.37 (s, 3H), 2.32 – 2.18 (m, 2H); $^{13}$C NMR (75 MHz, $CDCl_3$) δ 193.8, 143.0, 141.7, 137.6, 137.3, 136.9, 135.9, 132.6, 132.6, 131.8, 129.2, 128.9, 128.2, 128.0, 127.7, 127.3, 126.9, 126.7, 126.2, 125.9, 125.8, 54.4, 37.0, 23.9, 21.3; IR (film) $\nu_{max}$ 3061, 1643, 1597, 1493, 1447, 1342, 1276, 1212, 1161, 1091, 962, 891, 816, 718, 699, 671, 653, 574, 548 cm$^{-1}$; HRMS (ESI) calcd for $C_{29}H_{25}NO_3SH^+$ $[M+H]^+$ 468.1628, found 468.1628.

***tert*-Butyl 3-(3-benzoyl-1-tosyl-1,2,5,6-tetrahydropyridin-2-yl)-1*H*-indole-1-carboxylate (3ar)**

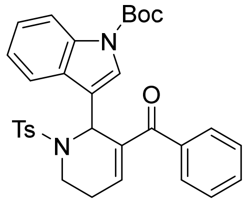


**3ar**

Prepared according to the general procedure as described above in 72% yield. It was purified by flash chromatography (15% EtOAc/PE) to afford a white solid. mp = 170 – 172 °C; $^1$H NMR (300 MHz, $CDCl_3$) δ 8.23 – 8.14 (m, 1H), 8.06 (d, *J* = 7.6 Hz, 1H), 7.85 – 7.77 (m, 2H), 7.66 – 7.60 (m, 2H), 7.60 – 7.51 (m, 1H), 7.51 – 7.42 (m, 2H), 7.41 – 7.29 (m, 3H), 7.23 (d, *J* = 8.0 Hz, 2H), 6.61 (s, 1H), 6.54 (t, *J* = 3.9 Hz, 1H), 3.94 (m, 1H), 3.40 – 3.25 (m, 1H), 2.40 (s, 3H), 2.23 (d, *J* = 5.4 Hz, 2H), 1.67 (s, 9H); $^{13}$C NMR (75 MHz, $CDCl_3$) δ 193.6, 149.3, 143.1, 141.2, 137.4, 137.1, 136.9, 135.3, 131.8, 129.2, 128.8, 128.0, 127.0, 124.8, 124.6, 122.7, 120.7, 118.9, 114.5, 83.7, 47.6, 37.1, 27.8, 23.3, 21.1; IR (film) $\nu_{max}$ 2978, 1735, 1646, 1453, 1368, 1268, 1224, 1161, 1083, 979, 739, 712, 671, 564 cm$^{-1}$; HRMS (ESI) calcd for $C_{32}H_{32}N_2O_5SH^+$ $[M+H]^+$ 557.2105, found 557.2104.

**(2-Fluorophenyl)(2-phenyl-1-tosyl-1,2,5,6-tetrahydropyridin-3-yl)methanone (3ba)**

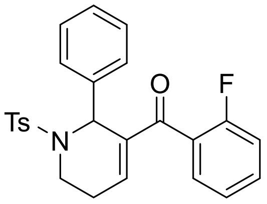

**3ba**

Prepared according to the general procedure as described above in 97% yield. It was purified by flash chromatography (15% EtOAc/PE) to afford a white solid. mp = 146 – 148 °C; $^{1}$H NMR (300 MHz, $CDCl_3$) δ 7.80 – 7.62 (m, 2H), 7.50 – 7.36 (m, 3H), 7.36 – 7.27 (m, 3H), 7.27 – 7.15 (m, 4H), 7.13 – 7.07 (m, 1H), 6.70 – 6.56 (m, 1H), 6.26 (s, 1H), 3.89 – 3.85 (m, 1H), 3.14 – 3.05 (m, 1H), 2.39 (s, 3H), 2.33 – 2.07 (m, 2H); $^{13}$C NMR (75 MHz, $CDCl_3$) δ 190.8, 158.9 (d, *J* = 250.5 Hz), 144.1 (d, *J* = 2.0 Hz), 138.3, 138.1, 137.5, 132.2(d, *J* = 8.3 Hz), 129.7 (d, *J* = 3.3 Hz), 129.2, 128.1, 127.9, 127.6, 126.8, 126.1 (d, *J* = 15.9 Hz), 123.9 (d, *J* = 3.5 Hz), 115.7 (d, *J* = 21.8 Hz), 53.9, 36.7, 24.2, 21.1; IR (film) $\nu_{max}$ 2926, 1653, 1611, 1484, 1451, 1343, 1290, 1215, 1162, 1090, 963, 751, 724, 705, 661, 569, 549 cm$^{-1}$; HRMS (ESI) calcd for $C_{25}H_{22}FNO_3SH^+$ $[M+H]^+$ 436.1377, found 436.1375.

**(4-Fluorophenyl)(2-phenyl-1-tosyl-1,2,5,6-tetrahydropyridin-3-yl)methanone (3ca)**

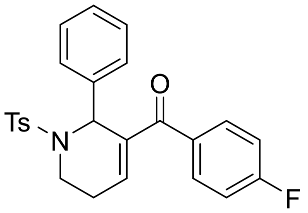

**3ca**

Prepared according to the general procedure as described above in 90% yield. It was purified by flash chromatography (16% EtOAc/PE) to afford a white solid. mp = 165 – 167 °C; $^{1}$H NMR (300 MHz, $CDCl_3$) δ 7.77 – 7.69 (m, 2H), 7.70 – 7.60 (m, 2H), 7.38 – 7.18 (m, 7H), 7.17 – 7.02 (m, 2H), 6.58 (t, *J* = 4.0 Hz, 1H), 6.27 (s, 1H), 4.01 – 3.78 (m, 1H), 3.22 – 3.14 (m, 1H), 2.38 (s, 3H), 2.29 – 2.18 (m, 2H); $^{13}$C NMR (75 MHz, $CDCl_3$) δ 192.3, 164.8 (d, *J* = 254.0 Hz), 143.0, 140.8, 138.3, 137.4 (d, *J* = 13.2 Hz), 132.9 (d, *J* = 3.1 Hz), 131.5, 131.4, 129.2, 128.1, 127.8, 127.6, 126.8, 115.1(d, *J* = 21.6 Hz), 54.3, 36.9, 23.9, 21.1; IR (film) $\nu_{max}$ 2927, 1646, 1598, 1504, 1453, 1342, 1275, 1161, 1091, 963, 891, 820, 751, 724, 705, 658, 568, 548 cm$^{-1}$; HRMS (ESI) calcd for $C_{25}H_{22}FNO_3SH^+$ $[M+H]^+$ 436.1377, found 436.1376.

**(2-Chlorophenyl)(2-phenyl-1-tosyl-1,2,5,6-tetrahydropyridin-3-yl)methanone (3da)**

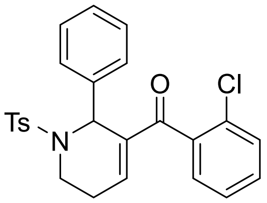

**3da**

Prepared according to the general procedure as described above in 90% yield. It was purified by flash chromatography (15% EtOAc/PE) to afford a white solid. mp = 155 – 157 °C; $^{1}$H NMR (300 MHz, $CDCl_3$) δ 7.80 – 7.66 (m, 2H), 7.50 – 7.18 (m, 10H), 7.11 – 6.95 (m, 1H), 6.55 (t, $J$ = 3.9 Hz, 1H), 6.20 (s, 1H), 4.00 – 3.70 (m, 1H), 3.14 – 3.03 (m, 1H), 2.41 (s, 3H), 2.36 – 2.02 (m, 2H); $^{13}$CNMR (75 MHz, $CDCl_3$) δ 192.7, 145.7, 143.0, 138.1, 137.8, 137.6, 137.5, 130.5, 130.4, 129.6, 129.3, 128.2, 128.0, 127.9, 127.6, 126.8, 126.1, 53.7, 36.6, 24.2, 21.1; IR (film) $\nu_{max}$ 2927, 1659, 1493, 1452, 1376, 1343, 1287, 1161, 1092, 963, 893, 765, 724, 700, 659, 645, 566, 547 cm$^{-1}$; HRMS (ESI) calcd for $C_{25}H_{22}ClNO_3SH^+$ $[M+H]^+$ 452.1082, found 452.1082.

**(3-Chlorophenyl)(2-phenyl-1-tosyl-1,2,5,6-tetrahydropyridin-3-yl)methanone (3ea)**

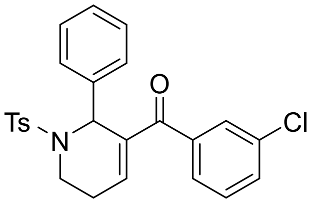

**3ea**

Prepared according to the general procedure as described above in 91% yield. It was purified by flash chromatography (15% EtOAc/PE) to afford a white solid. mp = 157 – 159 °C; $^{1}$H NMR (300 MHz, $CDCl_3$) δ 7.76 – 7.67 (m, 2H), 7.55 – 7.42 (m, 3H), 7.41 – 7.29 (m, 5H), 7.29 – 7.21 (m, 3H), 6.72 – 6.55 (m, 1H), 6.23 (s, 1H), 3.97 – 3.80 (m, 1H), 3.24 – 3.11 (m, 1H), 2.40 (s, 3H), 2.37 – 2.19 (m, 2H); $^{13}$C NMR (75 MHz, $CDCl_3$) δ 192.3, 143.1, 142.4, 138.6, 138.2, 137.5, 137.2, 134.1, 131.6, 129.4, 129.3, 128.6, 128.2, 127.8, 127.6, 126.8, 126.8, 54.2, 36.8, 23.9, 21.2; IR (film) $\nu_{max}$ 2927, 1653, 1493, 1452, 1417, 1343, 1275, 1211, 1161, 1090, 966, 893, 814, 731, 704, 661, 547 cm$^{-1}$; HRMS (ESI) calcd for $C_{25}H_{22}ClNO_3SH^+$ $[M+H]^+$ 452.1082, found 452.1081.

**(4-Chlorophenyl)(2-phenyl-1-tosyl-1,2,5,6-tetrahydropyridin-3-yl)methanone (3fa)**

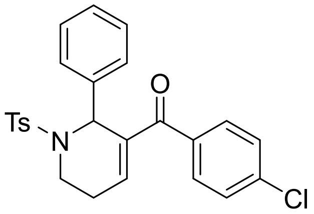

**3fa**

Prepared according to the general procedure as described above in 84% yield. It was purified by flash chromatography (15% EtOAc/PE) to afford a white solid. mp = 169 – 172 °C; $^1$H NMR (300 MHz, $CDCl_3$) δ 7.84 – 7.62 (m, 2H), 7.62 – 7.51 (m, 2H), 7.43 – 7.36 (m, 2H), 7.36 – 7.25 (m, 5H), 7.25 – 7.17 (m, 2H), 6.59 (t, *J* = 3.9 Hz, 1H), 6.25 (s, 1H), 3.90 (dd, *J* = 14.8, 6.0 Hz, 1H), 3.24 – 3.13 (m, 1H), 2.38 (s, 3H), 2.34 – 2.14 (m, 2H); $^{13}$CNMR (75 MHz, $CDCl_3$) δ 192.5, 143.0, 141.3, 138.2, 137.5, 137.3, 135.1, 130.3, 129.2, 128.3, 128.2, 127.8, 127.6, 126.8, 54.2, 36.8, 23.9, 21.1; IR (film) $\nu_{max}$ 2923, 1646, 1587, 1494, 1452, 1338, 1274, 1211, 1161, 1091, 1014, 962, 891, 816, 747, 700, 656, 548 cm$^{-1}$; HRMS (ESI) calcd for $C_{25}H_{22}ClNO_3SH^+$ $[M+H]^+$ 452.1082, found 452.1082.

**(3-Bromophenyl)(2-phenyl-1-tosyl-1,2,5,6-tetrahydropyridin-3-yl)methanone (3ga)**

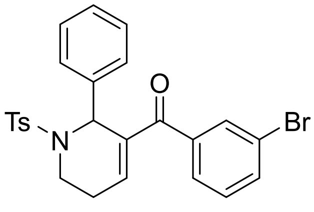

**3ga**

Prepared according to the general procedure as described above in 98% yield. It was purified by flash chromatography (15% EtOAc/PE) to afford a white solid. mp = 155 – 156 °C; $^1$H NMR (300 MHz, $CDCl_3$) δ 7.77 – 7.69 (m, 2H), 7.68 – 7.60 (m, 2H), 7.52 – 7.46 (m, 1H), 7.37 – 7.19 (m, 8H), 6.62 (t, *J* = 4.0 Hz, 1H), 6.23 (s, 1H), 4.02 – 3.77 (m, 1H), 3.24 – 3.06 (m, 1H), 2.40 (s, 3H), 2.36 – 2,10 (m, 2H); $^{13}$CNMR (75 MHz, $CDCl_3$) δ 192.2, 143.1, 142.4, 138.8, 138.2, 137.5, 137.2, 134.5, 131.5, 129.6, 129.3, 128.2, 127.8, 127.6, 127.3, 126.8, 122.1, 54.2, 36.8, 23.9, 21.2; IR (film) $\nu_{max}$ 2926, 1649, 1493, 1452, 1415, 1343, 1274, 1210, 1161, 1091, 965, 893, 802, 727, 702, 658, 547 cm$^{-1}$; HRMS (ESI) calcd for $C_{25}H_{22}BrNO_3SH^+$ $[M+H]^+$ 496.0577, found 496.0579.

**(4-Bromophenyl)(2-phenyl-1-tosyl-1,2,5,6-tetrahydropyridin-3-yl)methanone (3ha)**

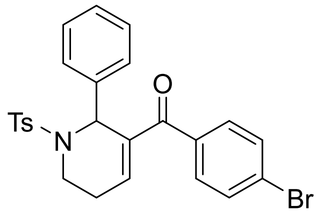

**3ha**

Prepared according to the general procedure as described above in 87% yield. It was purified by flash chromatography (15% EtOAc/PE) to afford a white solid. mp = 156 – 158 °C; $^1$H NMR (300 MHz, $CDCl_3$) δ 7.80 – 7.65 (m, 2H), 7.61 – 7.50 (m, 2H), 7.51 – 7.41 (m, 2H), 7.39 – 7.16 (m, 7H), 6.59 (t, *J* = 3.9 Hz, 1H), 6.25 (s, 1H), 4.00 – 3.75 (m, 1H), 3.23 – 3.10 (m, 1H), 2.38 (s, 3H), 2.31 – 2.12 (m, 2H); $^{13}$CNMR (75 MHz, $CDCl_3$) δ 192.7, 143.0, 141.5, 138.2, 137.5, 137.3, 135.6, 131.3, 130.4, 129.2, 128.2, 127.8, 127.6, 126.8, 126.7, 54.2, 36.9, 23.9, 21.2; IR (film) $\nu_{max}$ 2926, 1649, 1585, 1493, 1452, 1342, 1275, 1161, 1091, 1070, 1011, 962, 890, 815, 746, 727, 704, 655, 575, 548 cm$^{-1}$; HRMS (ESI) calcd for $C_{25}H_{22}BrNO_3SH^+$ $[M+H]^+$ 496.0577, found 496.0576.

**(3,4-Dichlorophenyl)(2-phenyl-1-tosyl-1,2,5,6-tetrahydropyridin-3-yl)methanone (3ia)**

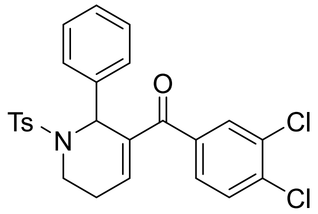

**3ia**

Prepared according to the general procedure as described above in 90% yield. It was purified by flash chromatography (15% EtOAc/PE) to afford a white solid. mp = 173 – 175 °C; $^1$H NMR (300 MHz, $CDCl_3$) δ 7.76 – 7.68 (m, 2H), 7.61 (d, *J* = 1.9 Hz, 1H), 7.50 (d, *J* = 8.2 Hz, 1H), 7.46 – 7.40 (m, 1H), 7.34 – 7.26 (m, 5H), 7.23 (d, *J* = 8.1 Hz, 2H), 6.62 (t, *J* = 3.9 Hz, 1H), 6.21 (s, 1H), 3.90 (dd, *J* = 14.9, 6.2 Hz, 1H), 3.20 – 3.08 (m, 1H), 2.40 (s, 3H), 2.35 – 2.13 (m, 2H); $^{13}$C NMR (75 MHz, $CDCl_3$) δ 191.3, 143.2, 142.2, 138.1, 137.4, 137.1, 136.5, 136.3, 132.6, 130.6, 130.2, 129.3, 128.2, 127.9, 127.8, 127.7, 126.8, 54.2, 36.8, 24.0, 21.2; IR (film) $\nu_{max}$ 2926, 1652, 1583, 1494, 1453, 1384, 1343, 1281, 1244, 1211, 1162, 1091, 1031, 967, 894, 816, 741, 720, 704, 656, 572, 550 cm$^{-1}$; HRMS (ESI) calcd for $C_{25}H_{21}Cl_2NO_3SH^+$ $[M+H]^+$ 484.0546, found 484.0554.

**(2-Phenyl-1-tosyl-1,2,5,6-tetrahydropyridin-3-yl)(p-tolyl)methanone (3ja)**

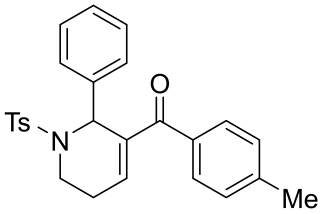


**3ja**

Prepared according to the general procedure as described above in 91% yield. It was purified by flash chromatography (5% EtOAc/PE) to afford a white solid. mp = 168 – 169 °C; $^1$H NMR (300 MHz, $CDCl_3$) δ 7.78 – 7.66 (m, 2H), 7.60 – 7.50 (m, 2H), 7.42 – 7.33 (m, 2H), 7.33 – 7.15 (m, 7H), 6.57 (t, *J* = 3.9 Hz, 1H), 6.30 (s, 1H), 4.05 – 3.80 (m, 1H), 3.21 – 3.10 (m, 1H), 2.39 (d, *J* = 6.4 Hz, 6H), 2.29 – 2.13 (m, 2H); $^{13}$C NMR (75 MHz, $CDCl_3$) δ 193.4, 142.9, 142.6, 140.5, 138.5, 137.6, 137.4, 134.1, 129.2, 129.1, 128.6, 128.1, 127.91 127.5, 126.8, 54.3, 37.0, 23.8, 21.2, 21.1; IR (film) $\nu_{max}$ 2925, 1642, 1606, 1493, 1452, 1342, 1276, 1211, 1161, 1091, 962, 890, 815, 744, 724, 705, 657, 569, 548 cm$^{-1}$; HRMS (ESI) calcd for $C_{26}H_{25}NO_3SH^+$ $[M+H]^+$ 432.1628, found 432.1626.

**(2-Methoxyphenyl)(2-phenyl-1-tosyl-1,2,5,6-tetrahydropyridin-3-yl)methanone (3ka)**

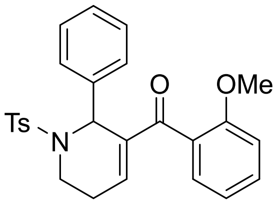


**3ka**

Prepared according to the general procedure as described above in 98% yield. It was purified by flash chromatography (15% EtOAc/PE) to afford a white solid. mp = 164 – 168 °C; $^1$H NMR (300 MHz, $CDCl_3$) δ 7.82 – 7.61 (m, 2H), 7.48 – 7.41 (m, 2H), 7.41 – 7.27 (m, 4H), 7.25 – 7.18 (m, 2H), 7.07 – 6.88 (m, 3H), 6.53 (t, *J* = 4.0 Hz, 1H), 6.25 (s, 1H), 3.79 (s, 4H), 3.12 – 3.01 (m, 1H), 2.41 (s, 3H), 2.35 – 1.96 (m, 2H); $^{13}$C NMR (75 MHz, $CDCl_3$) δ 194.1, 156.3, 143.1, 142.8, 138.6, 138.5, 137.7, 130.9, 129.1, 128.4, 128.2, 128.1, 127.8, 127.4, 126.8, 120.1, 111.0, 55.3, 53.8, 36.8, 24.0, 21.1; IR (film) $\nu_{max}$ 2926, 1653, 1598, 1488, 1454, 1373, 1342, 1293, 1245, 1161, 1090, 1068, 1020, 963, 755, 724, 698, 664, 570, 548 cm$^{-1}$; HRMS (ESI) calcd for $C_{26}H_{25}NO_4SH^+$ $[M+H]^+$ 448.1577, found 448.1577.

**(3-Methoxyphenyl)(2-phenyl-1-tosyl-1,2,5,6-tetrahydropyridin-3-yl)methanone (3la)**

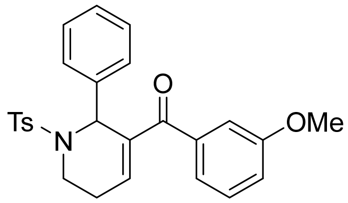


**3la**

Prepared according to the general procedure as described above in 98% yield. It was purified by flash chromatography (15% EtOAc/PE) to afford a white solid. mp = 62 – 64 °C; $^1$H NMR (300 MHz, $CDCl_3$) δ 7.76 – 7.67 (m, 2H), 7.40 – 7.33 (m, 2H), 7.33 – 7.25 (m, 4H), 7.25 – 7.18 (m, 2H), 7.18 – 7.10 (m, 2H), 7.09 – 7.03 (m, 1H), 6.63 (t, *J* = 3.9 Hz, 1H), 6.27 (s, 1H), 3.96 – 3.85 (m, 1H), 3.83 (s, 3H), 3.17 – 3.10 (m, 1H), 2.38 (s, 3H), 2.33 – 2.09 (m, 2H); $^{13}$C NMR (75 MHz, $CDCl_3$) δ 193.5, 159.2, 143.0, 141.5, 138.4, 138.2, 137.6, 137.3, 129.2, 128.9, 128.1, 127.9, 127.5, 126.8, 121.4, 117.6, 114.0, 55.1, 54.3, 36.9, 23.9, 21.1; IR (film) $\nu_{max}$ 2929, 1644, 1596, 1579, 1485, 1451, 1431, 1342, 1277, 1160, 1091, 1043, 977, 895, 801, 743, 723, 705, 656, 548 cm$^{-1}$; HRMS (ESI) calcd for $C_{26}H_{25}NO_4SH^+$ $[M+H]^+$ 448.1577, found 448.1578.

**(4-Nitrophenyl)(2-phenyl-1-tosyl-1,2,5,6-tetrahydropyridin-3-yl)methanone (3ma)**

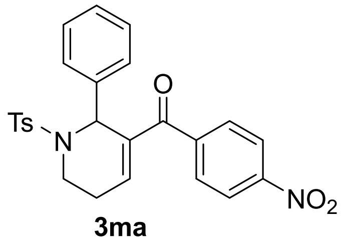


**3ma**

Prepared according to the general procedure as described above in 75% yield. It was purified by flash chromatography (15% EtOAc/PE) to afford a white solid. mp = 134 – 136 °C; $^1$H NMR (300 MHz, $CDCl_3$) δ 8.30 – 8.22 (m, 2H), 7.76 – 7.64 (m, 4H), 7.31 (d, *J* = 3.5 Hz, 5H), 7.23 (d, *J* = 8.0 Hz, 2H), 6.65 (t, *J* = 4.0 Hz, 1H), 6.22 (s, 1H), 3.90 (dd, *J* = 14.9, 6.6 Hz, 1H), 3.21 –3.09 (m, 1H), 2.40 (s, 3H), 2.38 – 2.18 (m, 2H); $^{13}$C NMR (75 MHz, $CDCl_3$) δ 192.0, 149.3, 143.5, 143.2, 142.3, 137.9, 137.5, 137.3, 129.5, 129.3, 129.2, 128.3, 127.8, 127.7, 126.9, 126.1, 123.2, 54.0, 36.7, 24.3, 21.2; IR (film) $\nu_{max}$ 2928, 1653, 1602, 1524, 1494, 1453, 1348, 1275, 1161, 1091, 963, 853, 722, 704, 656, 549 cm$^{-1}$; HRMS (ESI) calcd for $C_{25}H_{22}N_2O_5SH^+$ $[M+H]^+$ 461.1177, found 461.1183.

**(2-Phenyl-1-tosyl-1,2,5,6-tetrahydropyridin-3-yl)(thiophen-2-yl)methanone (3na)**

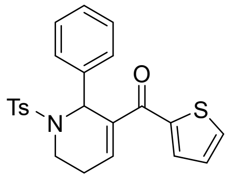

**3na**

Prepared according to the general procedure as described above in 93% yield. It was purified by flash chromatography (15% EtOAc/PE) to afford a white solid. mp = 130 – 132 °C; $^1$H NMR (300 MHz, $CDCl_3$) δ 7.78 – 7.71 (m, 2H), 7.68 – 7.54 (m, 2H), 7.41 – 7.34 (m, 2H), 7.29 – 7.18 (m, 5H), 7.11 (dd, $J$ = 5.0, 3.8 Hz, 1H), 6.77 (t, $J$ = 4.0 Hz, 1H), 6.30 (s, 1H), 3.96 – 3.82 (m, 1H), 3.25 – 3.03 (m, 1H), 2.37 (s, 3H), 2.27 – 2.09 (m, 2H); $^{13}$C NMR (75 MHz, $CDCl_3$) δ 185.1, 142.9, 142.1, 138.2, 138.1, 137.7, 137.6, 133.5, 132.9, 129.3, 128.1, 127.9, 127.6, 127.5, 126.8, 54.6, 36.9, 23.6, 21.1; IR (film) νmax 2926, 1621, 1513, 1493, 1452, 1413, 1341, 1281, 1211, 1160, 1090, 982, 959, 856, 815, 718, 704, 655, 578, 550 cm$^{-1}$; HRMS (ESI) calcd for $C_{23}H_{21}NO_3S_2H^+$ $[M+H]^+$ 424.1036, found 424.1032.

**Naphthalen-2-yl(2-phenyl-1-tosyl-1,2,5,6-tetrahydropyridin-3-yl)methanone (3oa)**

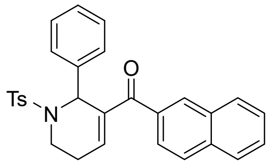

**3oa**

Prepared according to the general procedure as described above in 88% yield. It was purified by flash chromatography (15% EtOAc/PE) to afford a white solid. mp = 160 – 162 °C; $^1$H NMR (300 MHz, $CDCl_3$) δ 8.11 – 8.07 (m, 1H), 7.97 – 7.84 (m, 3H), 7.79 – 7.68 (m, 3H), 7.61 – 7.55 (m, 2H), 7.47 – 7.39 (m, 2H), 7.34 – 7.30 (m, 2H), 7.29 – 7.18 (m, 3H), 6.68 (t, $J$ = 3.9 Hz, 1H), 6.36 (s, 1H), 3.94 (dd, $J$ = 14.9, 5.9 Hz, 1H), 3.26 – 3.19 (m, 1H), 2.39 (s, 3H), 2.33 – 2.12 (m, 2H); $^{13}$C NMR (75 MHz, $CDCl_3$) δ 193.7, 142.9, 141.4, 138.5, 137.6, 137.5, 134.7, 134.1, 131.8, 130.0, 129.2, 128.8, 128.2, 128.0, 127.9, 127.8, 127.6, 127.5, 126.9, 126.5, 125.0, 54.4, 37.0, 23.9, 21.2; IR (film) $\nu_{max}$ 3059, 2926, 1653, 1641, 1597, 1453, 1371, 1342, 1284, 1161, 1090, 757, 722, 657 cm$^{-1}$; HRMS (ESI) calcd for $C_{29}H_{25}NO_3SH^+$ $[M+H]^+$ 468.1628, found 468.1628.

**(1-((4-Nitrophenyl)sulfonyl)-2-phenyl-1,2,5,6-tetrahydropyridin-3-yl)(phenyl)methanone (3pa)**

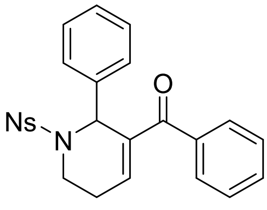


**3pa**

Prepared according to the general procedure as described above in 94% yield. It was purified by flash chromatography (15% EtOAc/PE) to afford a white solid. mp = 159 – 161°C; $^1$H NMR (300 MHz, $CDCl_3$) δ 8.31 – 8.10 (m, 2H), 8.12 – 7.89 (m, 2H), 7.69 – 7.59 (m, 2H), 7.59 – 7.49 (m, 1H), 7.48 – 7.34 (m, 4H), 7.31 – 7.25 (m, 3H), 6.60 (t, *J* = 4.0 Hz, 1H), 6.36 (s, 1H), 4.08 – 3.86 (m, 1H), 3.31 –3.23 (m, 1H), 2.38 – 2.15 (m, 2H); $^{13}$C NMR (75 MHz, $CDCl_3$) δ 193.4, 149.5, 146.2, 140.0, 137.5, 137.4, 136.4, 132.2, 128.9, 128.3, 128.1, 128.0, 127.9, 123.7, 54.7, 37.4, 24.2; IR (film) $\nu_{max}$ 3103, 3063, 2931, 1645, 1530, 1371, 1349, 1309, 1276, 1165, 1090, 991, 961, 856, 749, 740, 726, 714, 699, 606 cm$^{-1}$; HRMS (ESI) calcd for $C_{24}H_{20}N_2O_5SH^+$ $[M+H]^+$ 447.1026, found 447.1026.

**1-(2-Phenyl-1-tosyl-1,2,5,6-tetrahydropyridin-3-yl)ethan-1-one (3sa)**

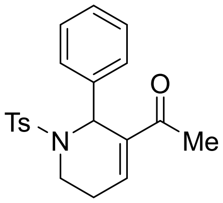


**3sa**

Prepared according to the general procedure as described above in about 73% yield. It was purified by flash chromatography (15% EtOAc/PE) to afford a white solid (contains some $TsNH_2$ which can't be separated completely, the ratio between **3sa** and $TsNH_2$ is 1:1.4, determined by $^1$H NMR, isolated yield was calculated based on the ratio); $^1$H NMR (400 MHz, Chloroform-d) δ 7.70 – 7.64 (m, 2H), 7.30 – 7.28 (m, 2H), 7.28 – 7.25 (m, 2H), 7.24 (d, J = 0.8 Hz, 1H), 7.22 (d, J = 2.5 Hz, 2H), 6.94 (dd, J = 4.6, 3.3 Hz, 1H), 6.04 (s, 1H), 3.85 – 3.79 (m, 1H), 3.05 – 2.97 (m, 1H), 2.44 – 2.41 (m, 2H) 2.41 (s, 3H), 2.23 (s, 3H); $^{13}$C NMR (101 MHz, $CDCl_3$) δ 196.2, 143.5, 143.3, 139.5, 139.1, 138.7, 138.5, 137.7, 129.6, 129.5, 128.3, 128.1, 127.8, 127.1, 126.4, 54.1, 36.8, 25.2, 24.2, 21.5; HRMS (ESI) calcd for $C_{20}H_{21}NO_3SH^+$ $[M+H]^+$ 356.1315, found 356.1312.

## The reaction on the gram-scale and further transformation of the product

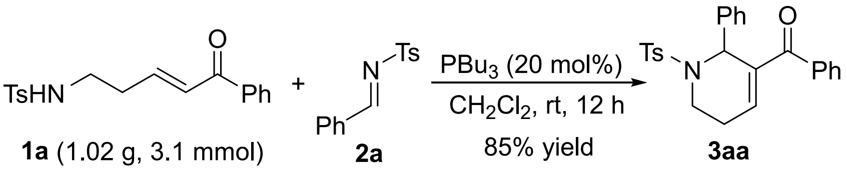


Under a nitrogen atmosphere, to a stirred solution of δ-sulfonamido-substituted enones **1** (3.1 mmol, 1.0 equiv) and *N*-sulfonylimine **2** (4.65 mmol, 1.5 equiv) in DCM (30 mL) was added catalyst $PBu_3$ (0.62 mmol, 20 mol%) via a syringe. Then the reaction solution was vigorously stirred at room temperature and monitored by TLC. After the reaction was complete, the mixture was directly purified by column chromatography on silica gel (petroleum ether/EtOAc as the eluent) to furnish the corresponding product.

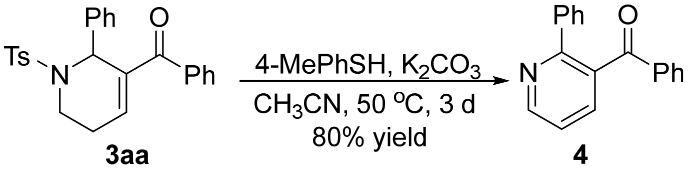


The cycloaddition **3aa** (83.5 mg, 0.2 mmol) was dissolved in 5 mL $CH_3CN$, then 4-MePhSH (29.8 mg, 0.24 mmol) and $K_2CO_3$ (82.8mg, 0.6 mmol) was added, the mixture was stirred at 50 °C for 72 hours. Once starting material was consumed (monitored by TLC), the mixture was concentrated to dryness. The residue was purified through flash column chromatography (EtOAc/PE) to afford the corresponding product **4** as a white solid (41.6 mg), 80% yield.

### Phenyl(2-phenylpyridin-3-yl)methanone (4)

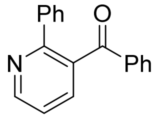


**4**

Prepared according to the general procedure as described above in 80% yield. It was purified by flash chromatography (15% EtOAc/PE) to afford a white solid. mp = 106 – 108 °C; $^1H$ NMR (300 MHz, $CDCl_3$) δ 8.33 (d, $J$ = 2.6 Hz, 1H), 7.67 – 7.56 (m, 2H), 7.49 – 7.39 (m, 1H), 7.39 – 7.29 (m, 3H), 7.29 – 7.24 (m, 2H), 7.18 – 7.04 (m, 4H); $^{13}C$ NMR (75 MHz, $CDCl_3$) δ 196.4, 152.4, 148.1, 138.5, 137.7, 135.9, 134.9, 133.2, 129.6, 128.6, 128.1, 128.0, 127.9, 123.8; IR (film) $\nu_{max}$ 3727, 2925, 1653, 1449, 1321, 749, 696 $cm^{-1}$; HRMS (ESI) calcd for $C_{18}H_{13}NOH^+$ $[M+H]^+$ 260.1070, found 260.1068.

**General Procedure for the Phosphine-Catalyzed [4+1] Annulation**

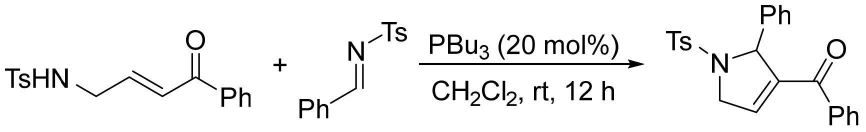


**5** **2a** **6**

Under a nitrogen atmosphere, to a stirred solution of γ-sulfonamido-substituted enone **5** (0.2 mmol, 1.0 equiv) and *N*-sulfonylimine **2a** (3.0 mmol, 1.5 equiv) in DCM (2 mL) was added the catalyst $PBu_3$ (0.04 mmol, 20 mol%) via a syringe. Then the reaction solution was vigorously stirred at room temperature and monitored by TLC. After the reaction was stirred for 12 h, the mixture was directly purified by column chromatography on silica gel (petroleum ether/EtOAc as the eluent) to furnish the corresponding product **6** in 23% yield.

**Phenyl(2-phenyl-1-tosyl-2,5-dihydro-1H-pyrrol-3-yl)methanone (6)**

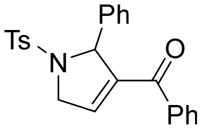


**6**

Prepared according to the general procedure as described above in 23% yield. It was purified by flash chromatography (15% EtOAc/PE) to afford a white solid (contains tracement of unknown impurities). $^1H$ NMR (300 MHz, Chloroform-d) δ 7.67 – 7.60 (m, 2H), 7.60 – 7.46 (m, 4H), 7.46 – 7.34 (m, 4H), 7.27 – 7.20 (m, 4H), 6.42 (t, $J$ = 2.0 Hz, 1H), 6.03 – 6.00 (m, 1H), 4.61 – 4.58 (m , 2H), 2.40 (s, 3H); $^{13}C$ NMR (101 MHz, $CDCl_3$) δ 190.0, 143.4, 142.7, 139.4, 137.2, 135.1, 132.8, 129.9, 129.8, 129.6, 128.8, 128.4, 128.3, 127.5, 127.3, 69.7, 55.7, 21.5; HRMS (ESI) calcd for $C_{24}H_{21}NO_3SH^+$ $[M+H]^+$ 404.1315, found 404.1313.

**Phosphine-Catalyzed Annulation of (*E*)-4-hydroxy-1-phenylbut-2-en-1-one**

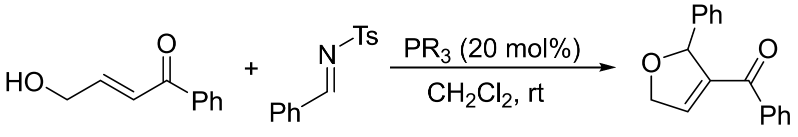


A hydroxyl-substituted enone, (*E*)-4-hydroxy-1-phenylbut-2-en-1-one (**7**), has also been examined under the standard reaction conditions. Unfortunately, no desired [4+1] annulation product **8** was observed. Further exploration through screening different phosphines such as $PPh_3$, $Ph_2PMe$, $PhPMe_2$ and $Me_3P$ had been carried out, but no product was observed on the TLC.

**(-)-Phenyl(2-phenyl-1-tosyl-1,2,5,6-tetrahydropyridin-3-yl)methanone (3aa)**

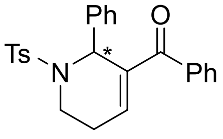


Prepared according to the general procedure as described above in 28% yield. It was purified by flash chromatography (20% EtOAc/PE) to afford a white solid. mp = 161 – 163 °C; $[\alpha]^{20}_{D}$= – 139.4 (*c* 0.34, $CH_2Cl_2$); mp = 161 – 163 °C; $^1$H NMR (300 MHz, $CDCl_3$) δ 7.77 – 7.67 (m, 2H), 7.64 – 7.57 (m, 2H), 7.55 – 7.48 (m, 1H), 7.47 – 7.34 (m, 4H), 7.33 – 7.25 (m, 3H), 7.24 – 7.18 (m, 2H), 6.64 – 6.52 (m, 1H), 6.29 (s, 1H), 3.94 – 3.86 (m, 1H), 3.31 – 3.04 (m, 1H), 2.38 (s, 3H), 2.25 – 2.18 (m, 2H); $^{13}$CNMR (75 MHz, $CDCl_3$) δ 192.5, 143.0, 141.3, 138.2, 137.5, 137.3, 135.1, 130.3, 129.2, 128.3, 128.2, 127.8, 127.6, 126.8, 54.2, 36.9, 24.0, 21.1; IR (film) $\nu_{max}$ 3061, 2927, 1645, 1597, 1493, 1447, 1342, 1275, 1212, 1161, 1091, 962, 891, 815, 718, 699, 671, 653, 574, 548 cm$^{-1}$; HRMS (ESI) calcd for $C_{25}H_{23}NO_3SH^+$ $[M+H]^+$ 418.1471, found 418.1470; HPLC analysis: 73% ee (RC-OD, isopropanol/hexane = 8:92, 1.0 mL/min, UV: 254 nm), $t_R$ = 20.364 min (minor), 22.279 min (major).

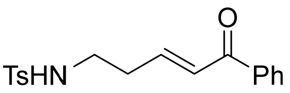

O
TsHN
Ph


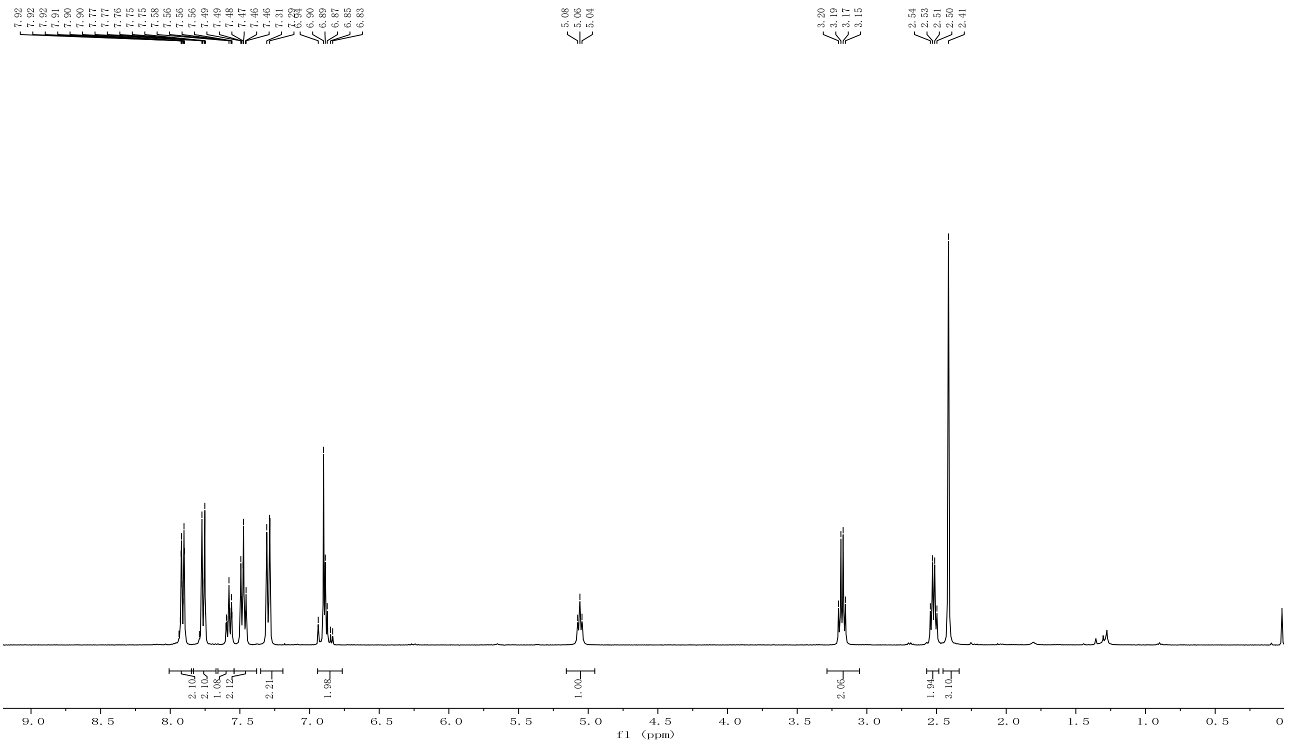

7.92 7.92 7.92 7.91 7.90 7.90 7.77 7.77 7.76 7.75 7.75 7.58 7.56 7.56 7.56 7.49 7.49 7.48 7.47 7.46 7.46 7.31 7.29 6.91 6.90 6.89 6.87 6.85 6.83
5.08 5.06 5.04
3.20 3.19 3.17 3.15
2.54 2.53 2.51 2.50 2.41
2.10 2.10 1.08 2.12 2.21 1.98 1.00 2.06 1.94 3.10
9.0 8.5 8.0 7.5 7.0 6.5 6.0 5.5 5.0 4.5 4.0 3.5 3.0 2.5 2.0 1.5 1.0 0.5 0
f1 (ppm)


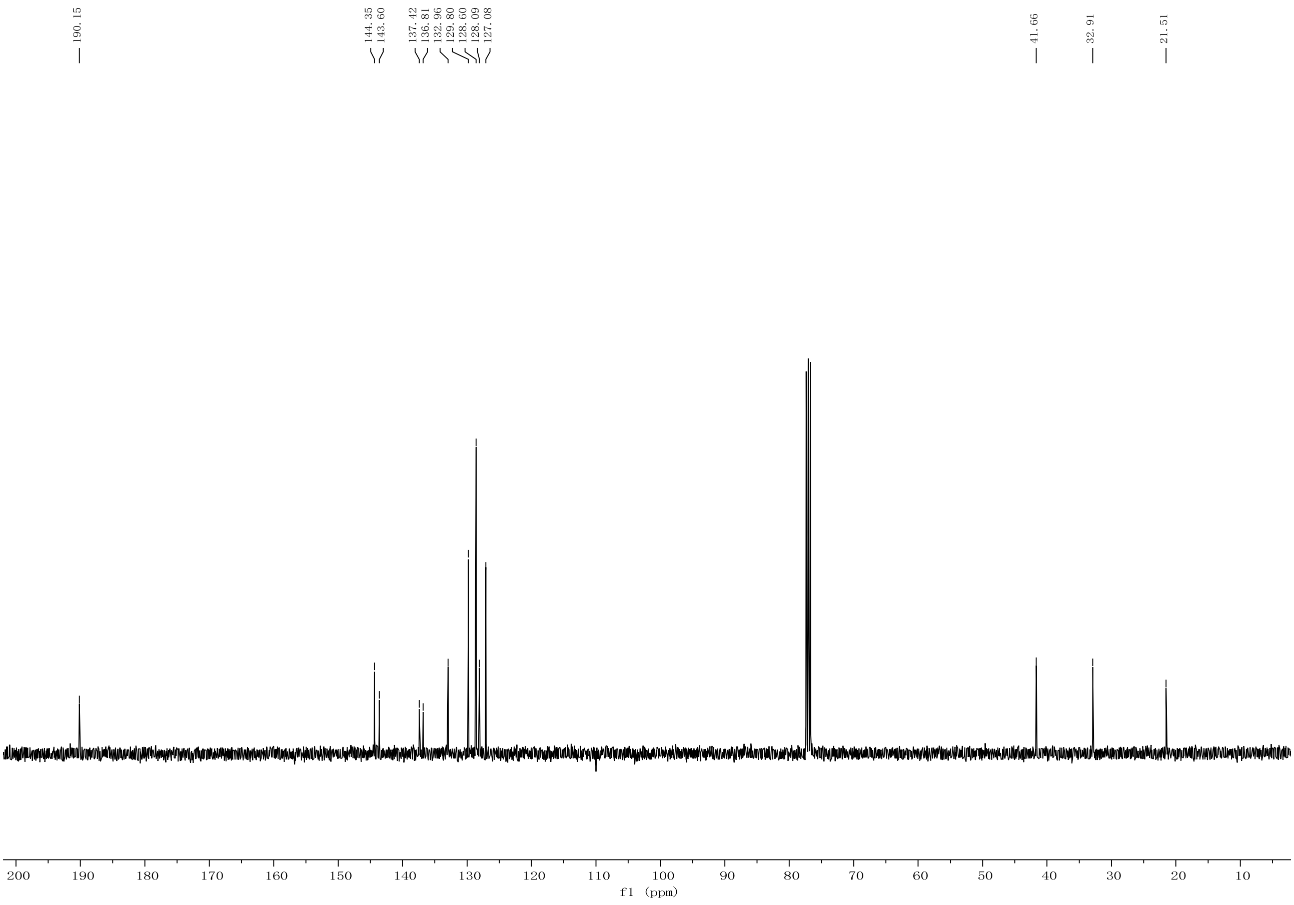

190.15
144.35 143.60 137.42 136.81 132.96 129.80 128.60 128.09 127.08
41.66 32.91 21.51
200 190 180 170 160 150 140 130 120 110 100 90 80 70 60 50 40 30 20 10
f1 (ppm)

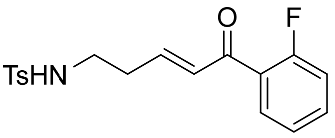

TsHN
O
F


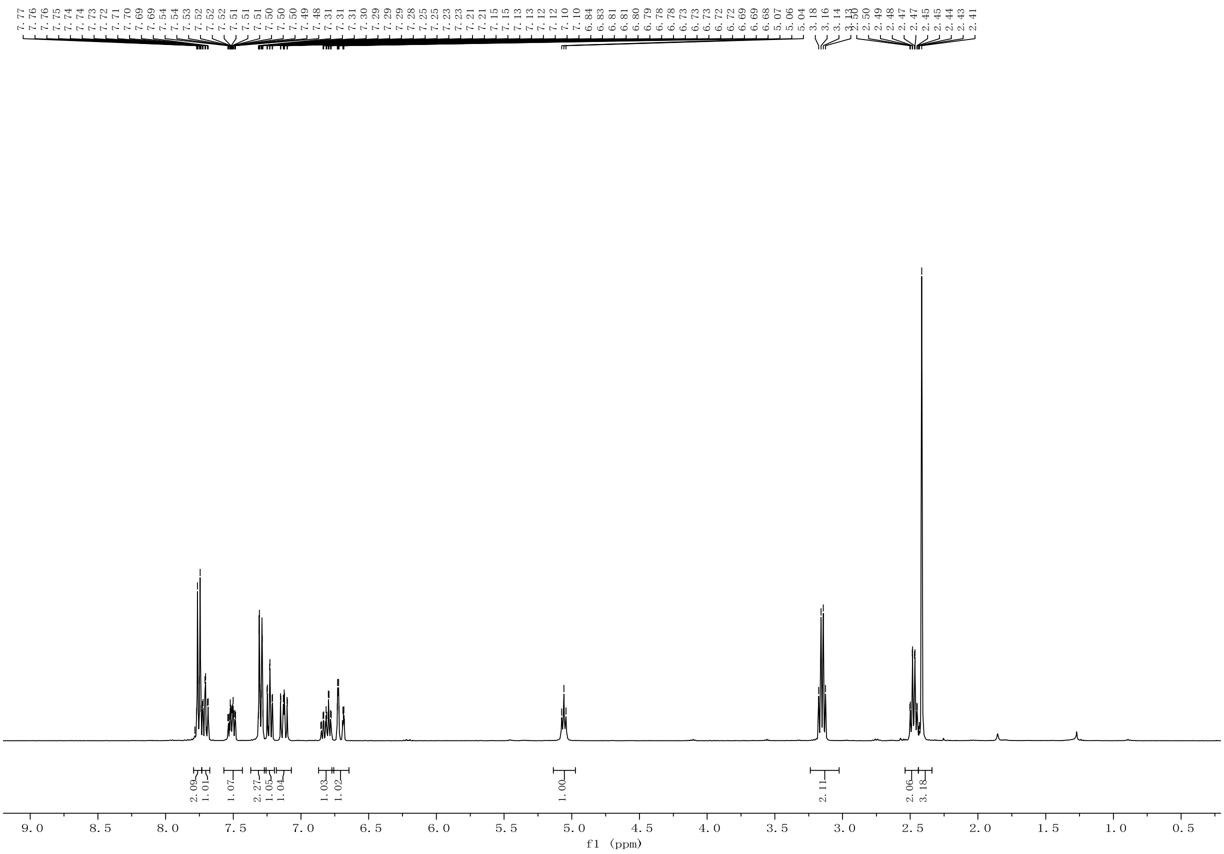

7.77 7.76 7.75 7.74 7.74 7.73 7.72 7.71 7.70 7.69 7.69 7.54 7.54 7.53 7.52 7.52 7.52 7.51 7.51 7.51 7.50 7.50 7.50 7.49 7.48 7.31 7.31 7.31 7.30 7.29 7.29 7.29 7.28 7.25 7.25 7.23 7.23 7.21 7.21 7.15 7.15 7.13 7.13 7.13 7.12 7.12 7.10 7.10 6.84 6.83 6.81 6.80 6.79 6.78 6.73 6.73 6.73 6.72 6.72 6.69 6.69 6.68 5.07 5.06 5.04 3.18 3.16 3.14 3.13 2.50 2.50 2.49 2.48 2.48 2.47 2.47 2.45 2.44 2.43 2.41
2.09 1.01 1.07 2.27 1.05 1.04 1.03 1.02 1.00 2.11 2.06 3.18
9.0 8.5 8.0 7.5 7.0 6.5 6.0 5.5 5.0 4.5 4.0 3.5 3.0 2.5 2.0 1.5 1.0 0.5
f1 (ppm)


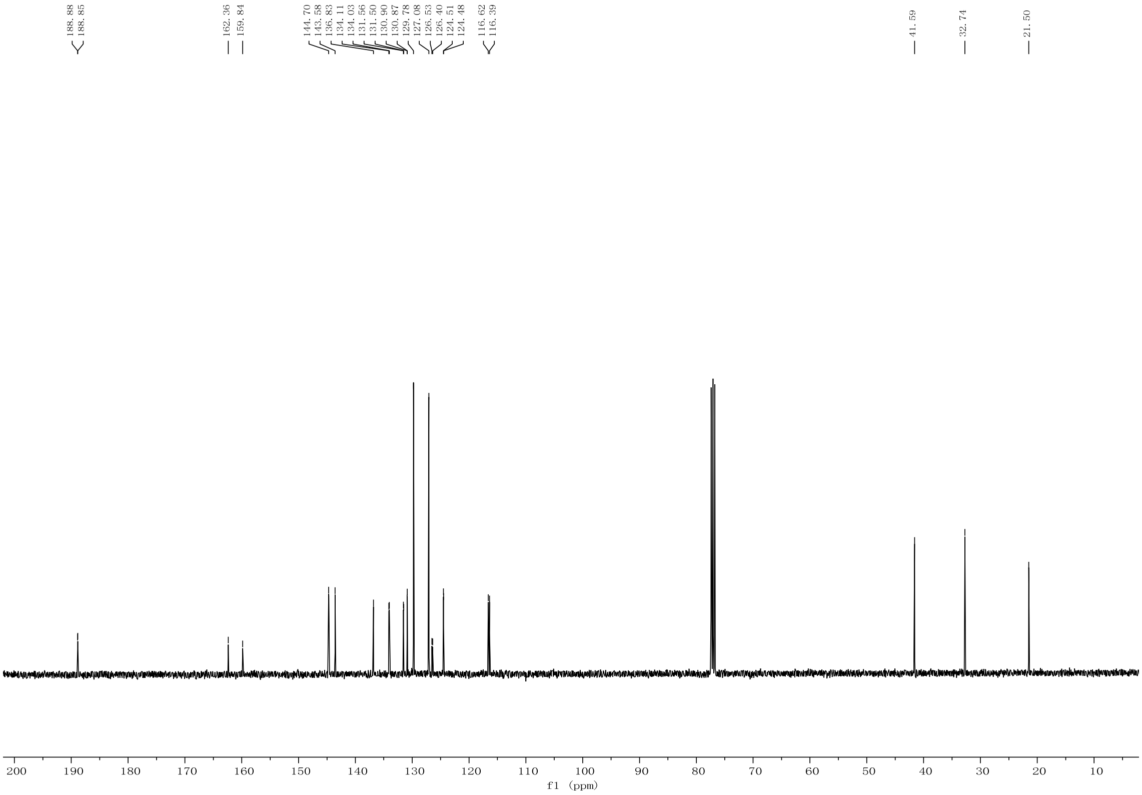

188.88 188.85 162.36 159.84 144.70 143.58 136.83 134.11 134.03 131.56 131.50 130.90 130.87 129.78 127.08 126.53 126.40 124.51 124.48 116.62 116.39 41.59 32.74 21.50
200 190 180 170 160 150 140 130 120 110 100 90 80 70 60 50 40 30 20 10
f1 (ppm)

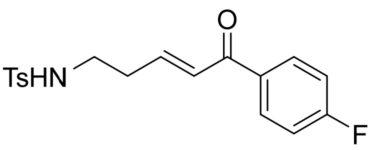
O
TsHN
F

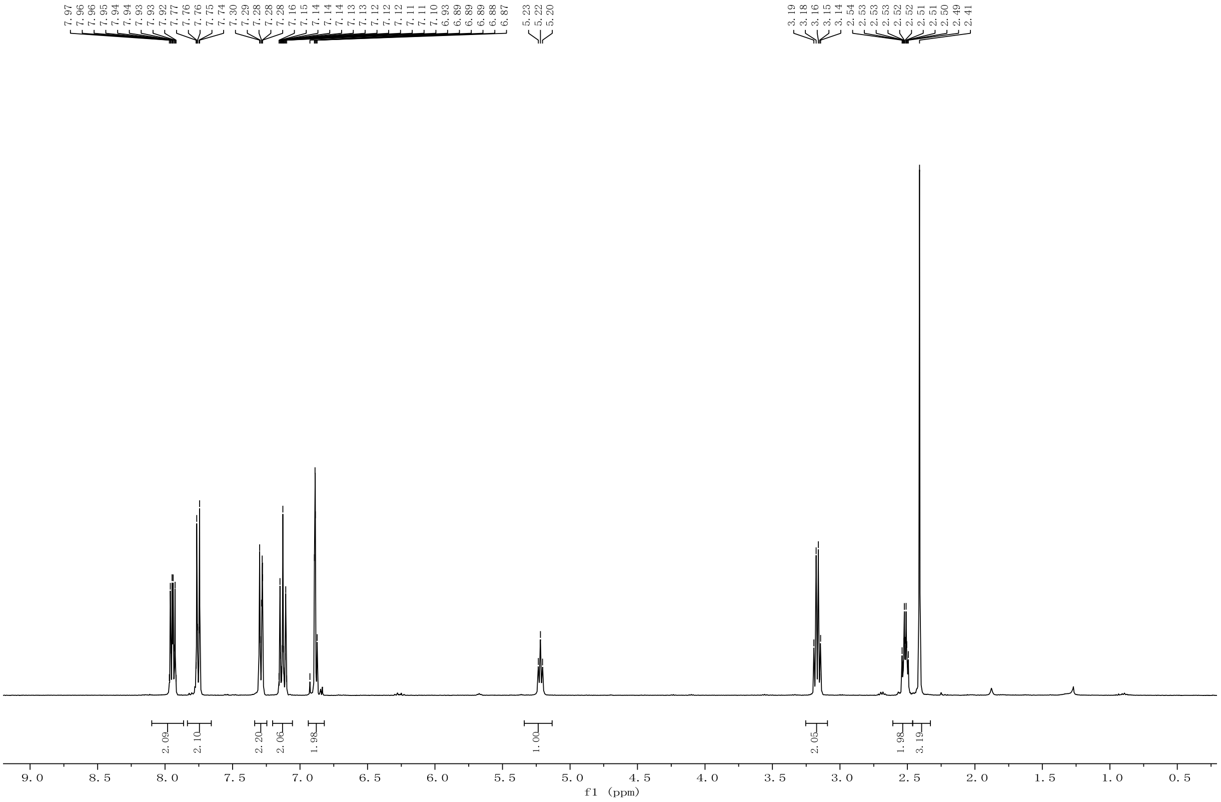
7.97 7.96 7.96 7.95 7.94 7.94 7.93 7.93 7.92 7.77 7.76 7.75 7.74 7.30 7.29 7.28 7.28 7.28 7.16 7.15 7.14 7.14 7.14 7.13 7.13 7.12 7.12 7.12 7.11 7.11 7.10 6.93 6.89 6.89 6.89 6.88 6.87 5.23 5.22 5.20
3.19 3.18 3.16 3.15 3.14 2.54 2.53 2.53 2.53 2.52 2.52 2.51 2.51 2.50 2.49 2.41
2.09 2.10 2.20 2.06 1.98 1.00 2.05 1.98 3.19
9.0 8.5 8.0 7.5 7.0 6.5 6.0 5.5 5.0 4.5 4.0 3.5 3.0 2.5 2.0 1.5 1.0 0.5
f1 (ppm)

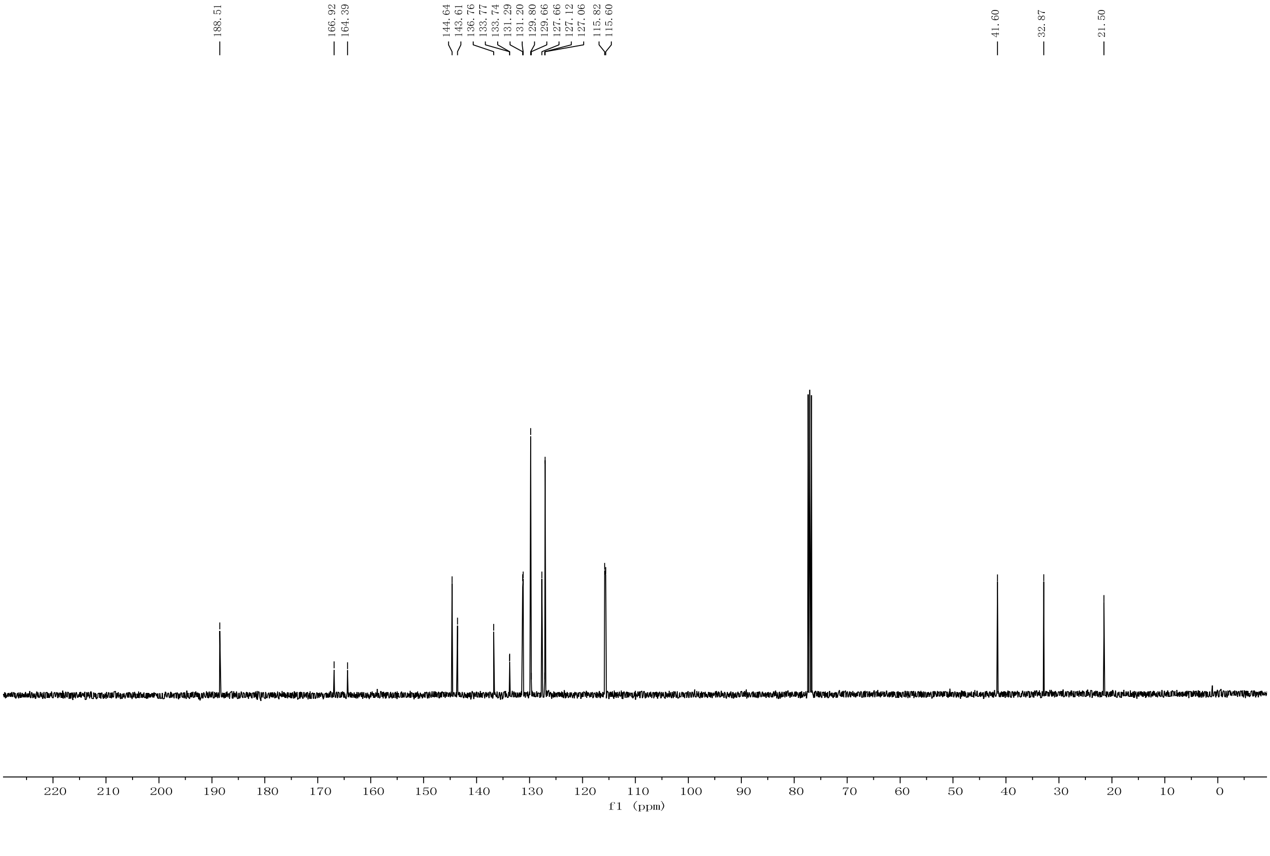
188.51
166.92 164.39
144.64 143.61 136.76 133.77 133.74 131.29 131.20 129.80 129.66 127.66 127.12 127.06 115.82 115.60
41.60
32.87
21.50
220 210 200 190 180 170 160 150 140 130 120 110 100 90 80 70 60 50 40 30 20 10 0
f1 (ppm)

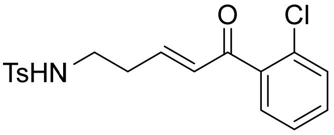
O
Cl
TsHN

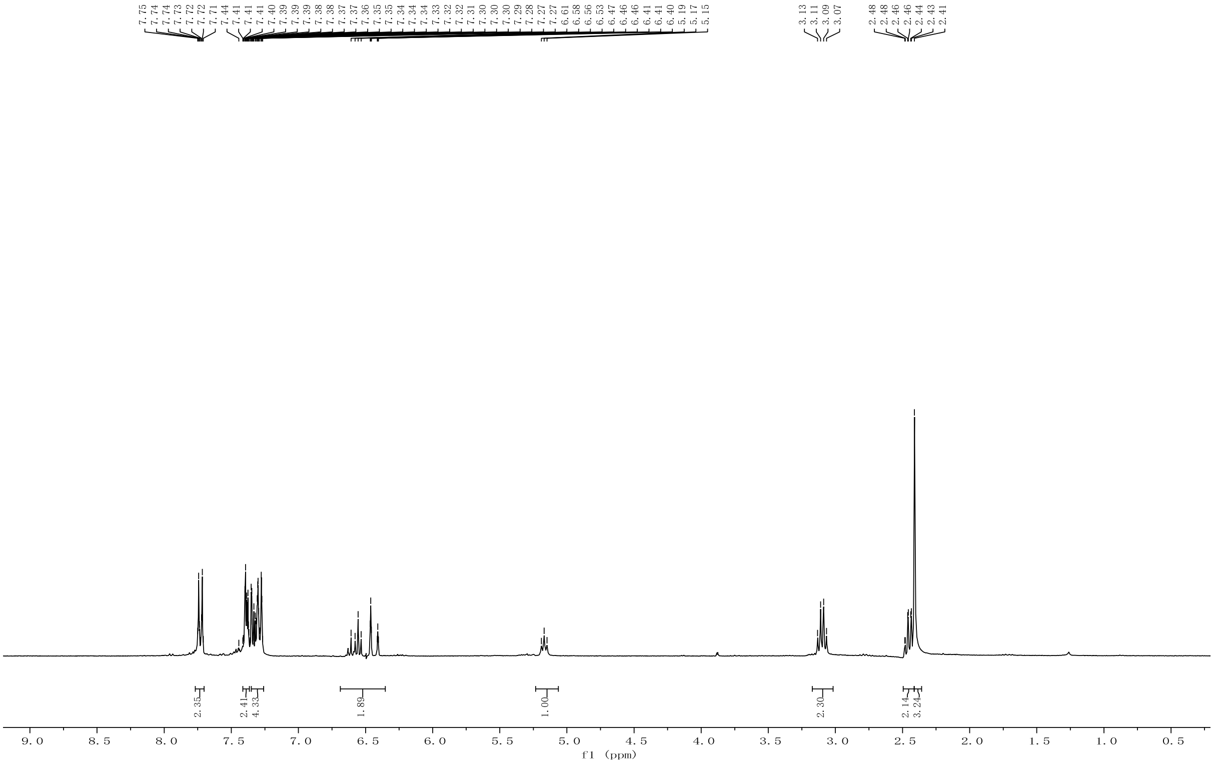
7.75 7.74 7.74 7.73 7.72 7.72 7.71 7.44 7.41 7.41 7.41 7.40 7.39 7.39 7.39 7.38 7.38 7.37 7.37 7.36 7.35 7.35 7.34 7.34 7.34 7.33 7.32 7.32 7.31 7.30 7.30 7.30 7.29 7.28 7.27 7.27 6.61 6.58 6.56 6.53 6.47 6.46 6.46 6.41 6.41 6.40 5.19 5.17 5.15
3.13 3.11 3.09 3.07
2.48 2.48 2.46 2.46 2.44 2.43 2.41
2.35
2.41
4.33
1.89
1.00
2.30
2.14
3.24
9.0 8.5 8.0 7.5 7.0 6.5 6.0 5.5 5.0 4.5 4.0 3.5 3.0 2.5 2.0 1.5 1.0 0.5
f1 (ppm)

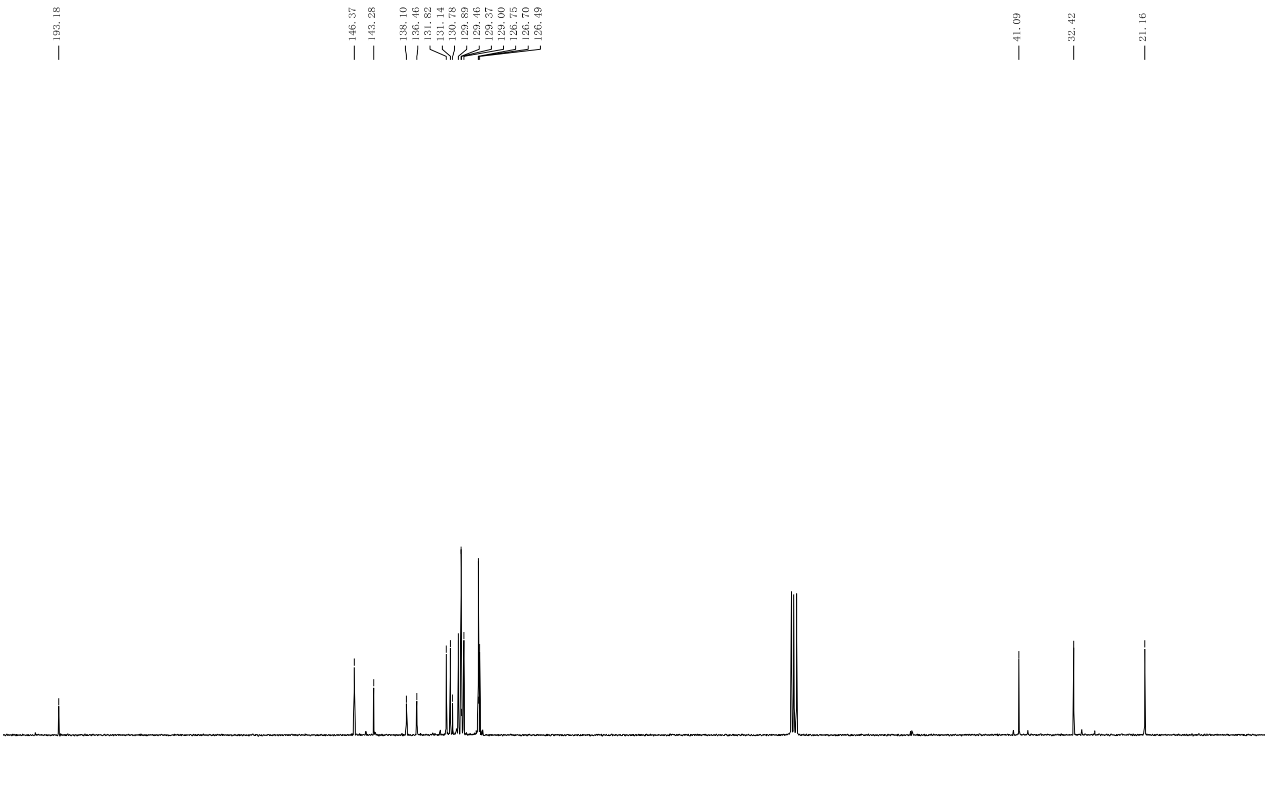
193.18
146.37 143.28
138.10 136.46 131.82 131.14 130.78 129.89 129.46 129.37 129.00 126.75 126.70 126.49
41.09
32.42
21.16
200 190 180 170 160 150 140 130 120 110 100 90 80 70 60 50 40 30 20 10
f1 (ppm)

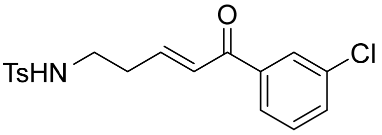

O
Cl
TsHN


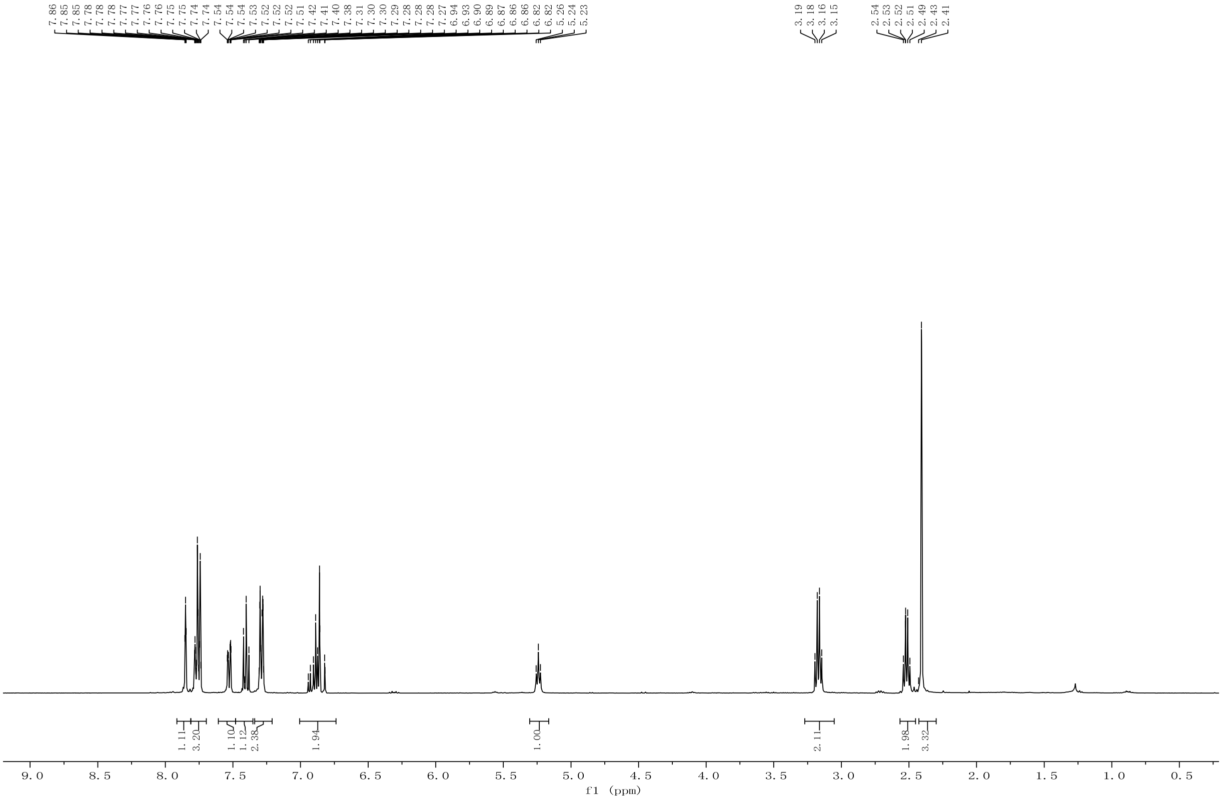

7.86 7.85 7.85 7.78 7.78 7.78 7.77 7.77 7.76 7.76 7.75 7.75 7.74 7.74 7.54 7.54 7.54 7.53 7.52 7.52 7.52 7.51 7.42 7.41 7.40 7.38 7.31 7.30 7.30 7.29 7.28 7.28 7.28 7.27 6.94 6.93 6.90 6.89 6.87 6.86 6.86 6.82 6.82 5.26 5.24 5.23
3.19 3.18 3.16 3.15
2.54 2.53 2.52 2.51 2.49 2.43 2.41
1.11 3.20 1.10 1.12 2.38 1.94 1.00 2.11 1.98 3.32
9.0 8.5 8.0 7.5 7.0 6.5 6.0 5.5 5.0 4.5 4.0 3.5 3.0 2.5 2.0 1.5 1.0 0.5
f1 (ppm)


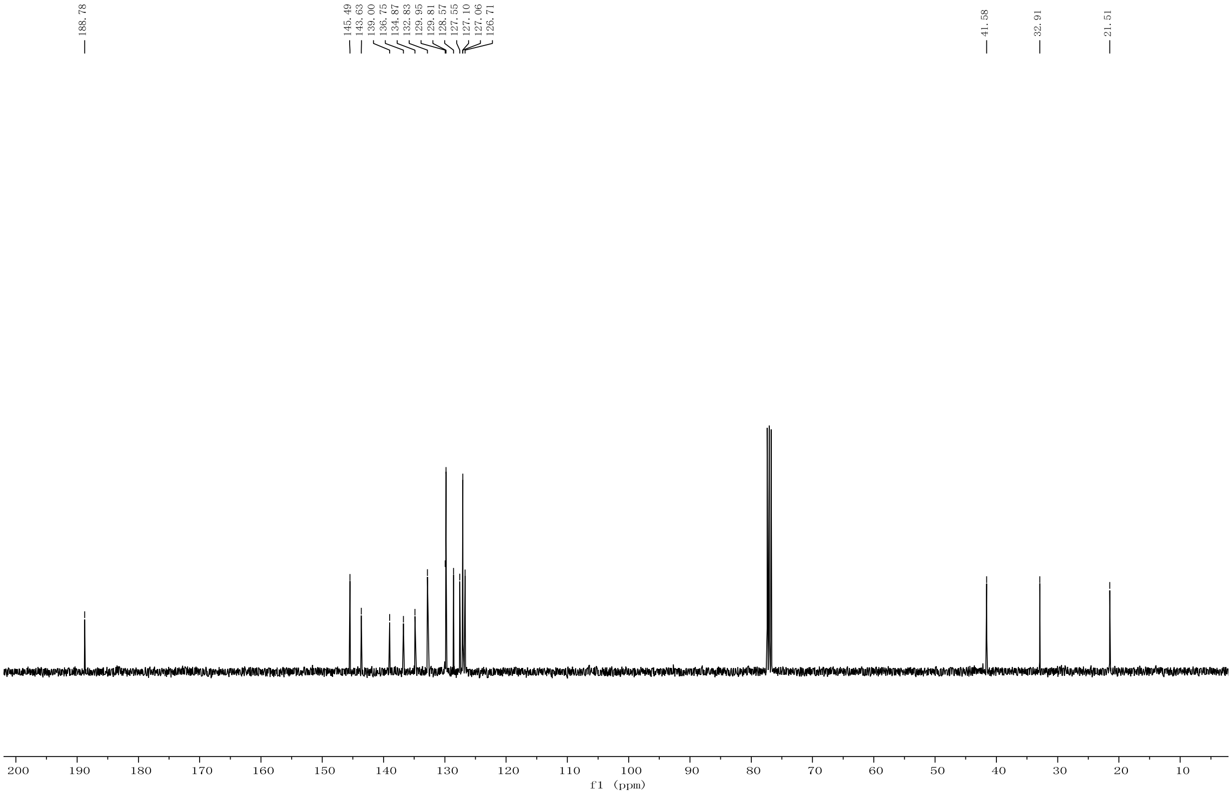

188.78
145.49 143.63 139.00 136.75 134.87 132.83 129.95 129.81 128.57 127.55 127.10 127.06 126.71
41.58
32.91
21.51
200 190 180 170 160 150 140 130 120 110 100 90 80 70 60 50 40 30 20 10
f1 (ppm)

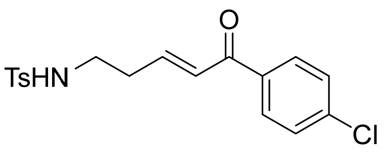

TsHN
O
Cl


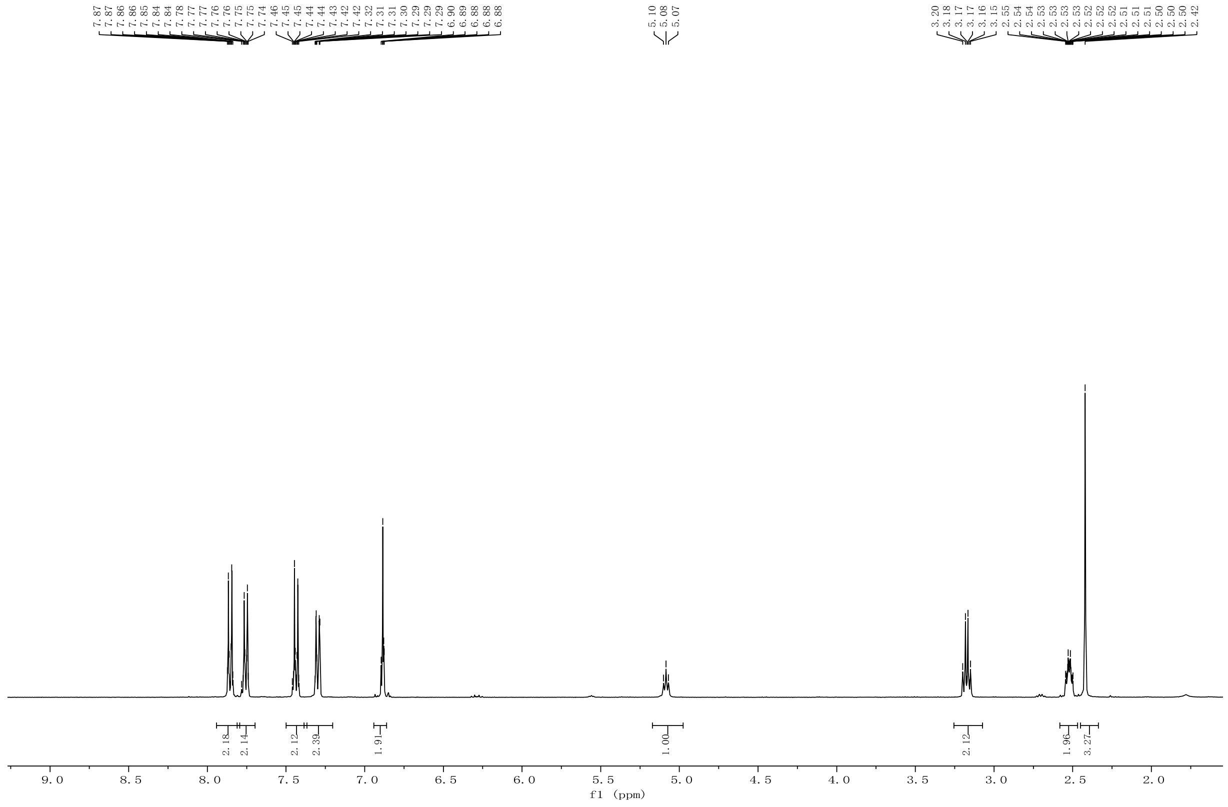

7.87 7.87 7.86 7.86 7.85 7.84 7.84 7.78 7.77 7.77 7.76 7.76 7.75 7.75 7.74 7.46 7.45 7.45 7.44 7.44 7.43 7.42 7.42 7.32 7.31 7.31 7.30 7.29 7.29 7.29 6.90 6.89 6.88 6.88 6.88
5.10 5.08 5.07
3.20 3.18 3.17 3.17 3.16 3.15 2.55 2.54 2.54 2.53 2.53 2.53 2.53 2.52 2.52 2.52 2.51 2.51 2.50 2.50 2.50 2.50 2.42
2.18 2.14 2.12 2.39 1.91 1.00 2.12 1.96 3.27
f1 (ppm)


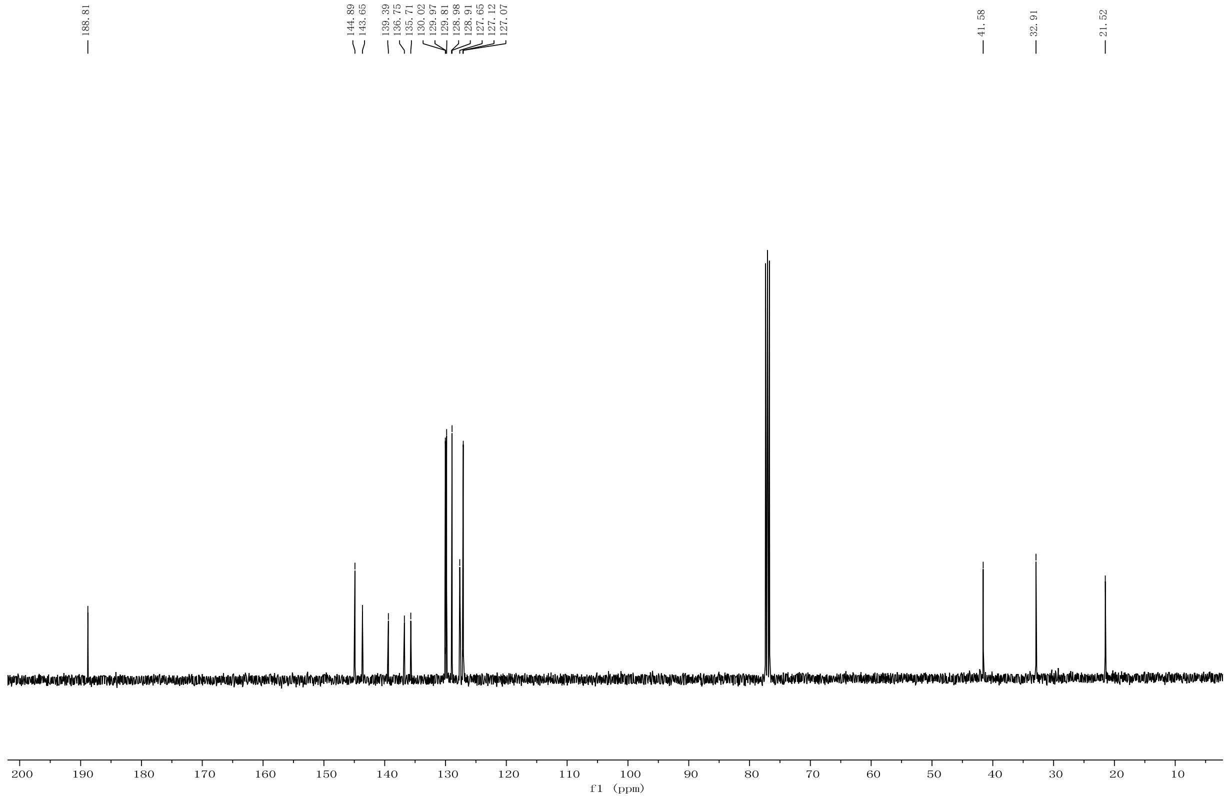

188.81
144.89 143.65 139.39 136.75 135.71 130.02 129.97 129.81 128.98 128.91 127.65 127.12 127.07
41.58 32.91 21.52
f1 (ppm)

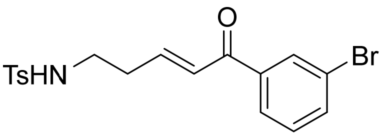

O
TsHN
Br


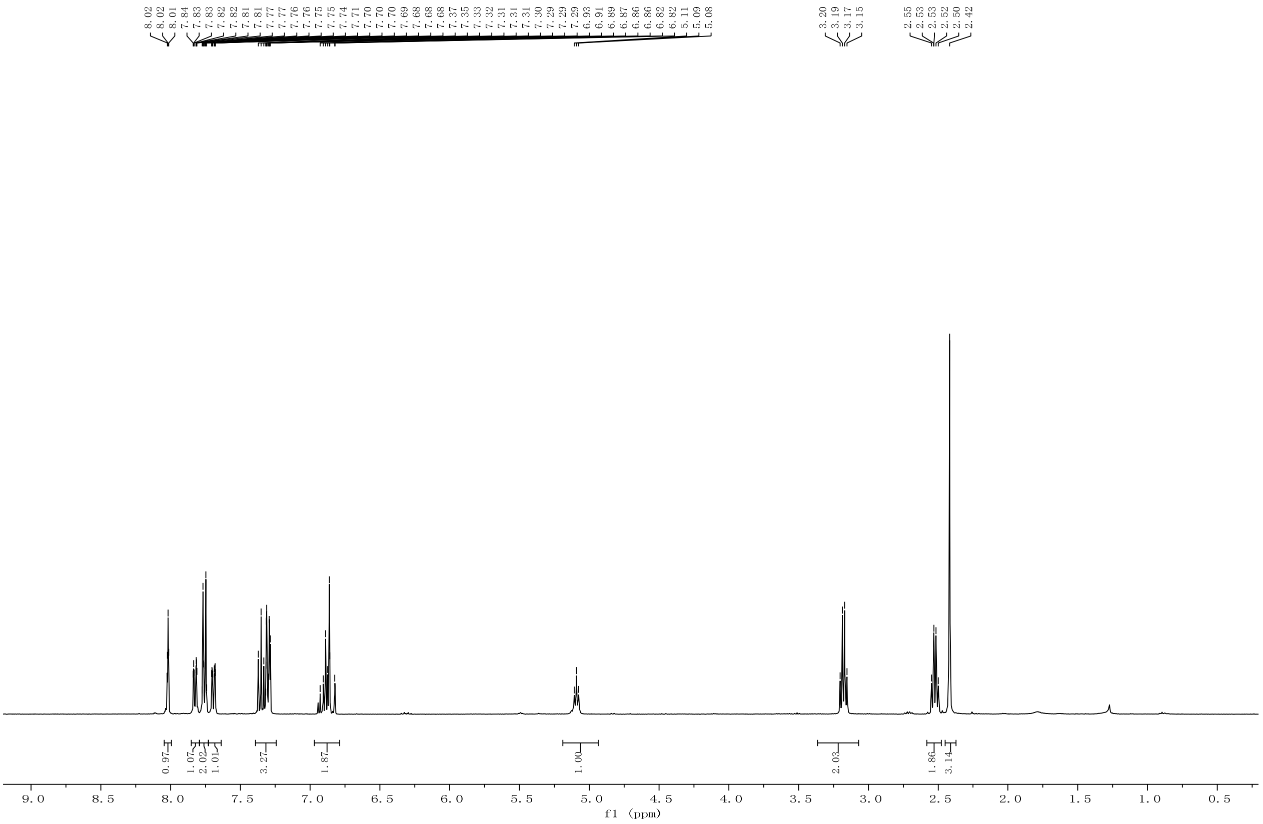

f1 (ppm)


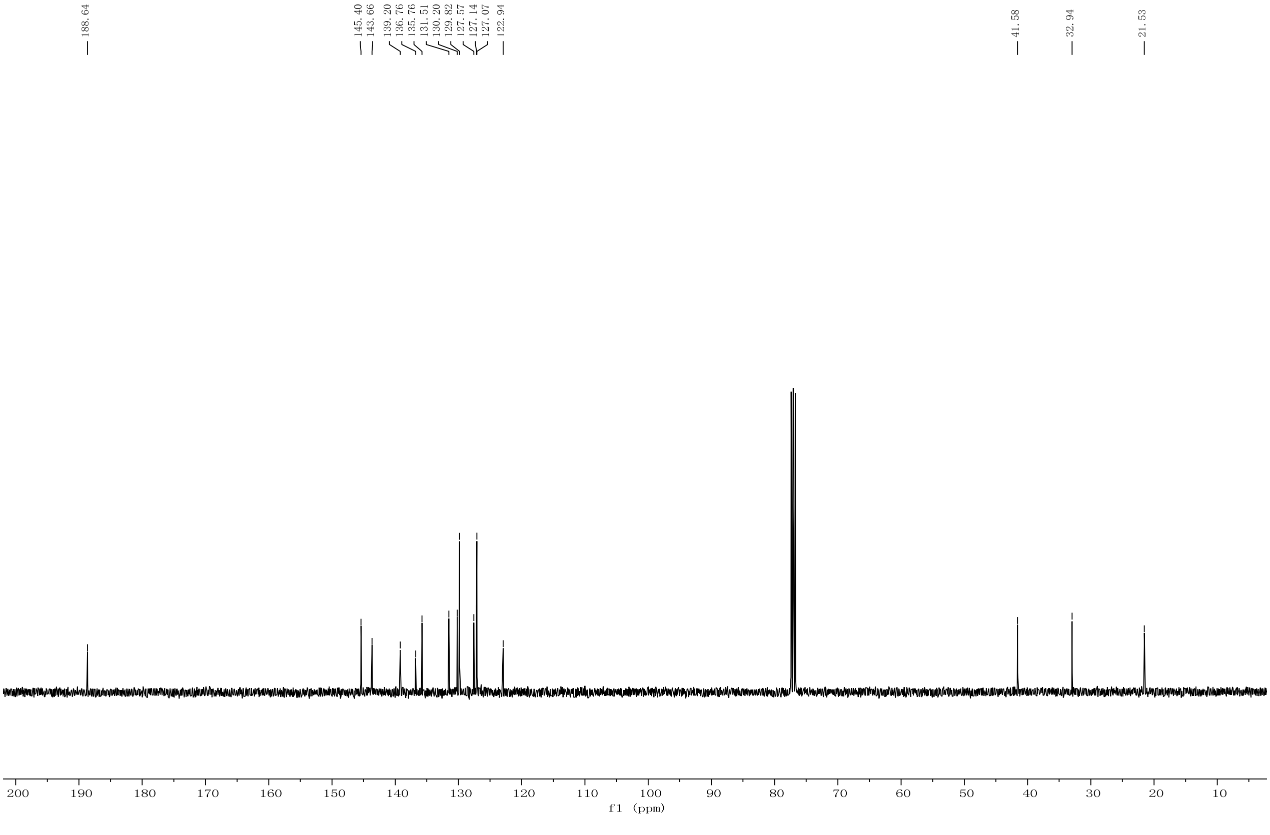

188.64
145.40
143.66
139.20
136.76
135.76
131.51
130.20
129.82
127.57
127.14
127.07
122.94
41.58
32.94
21.53
f1 (ppm)

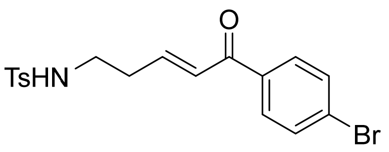

TsHN
O
Br


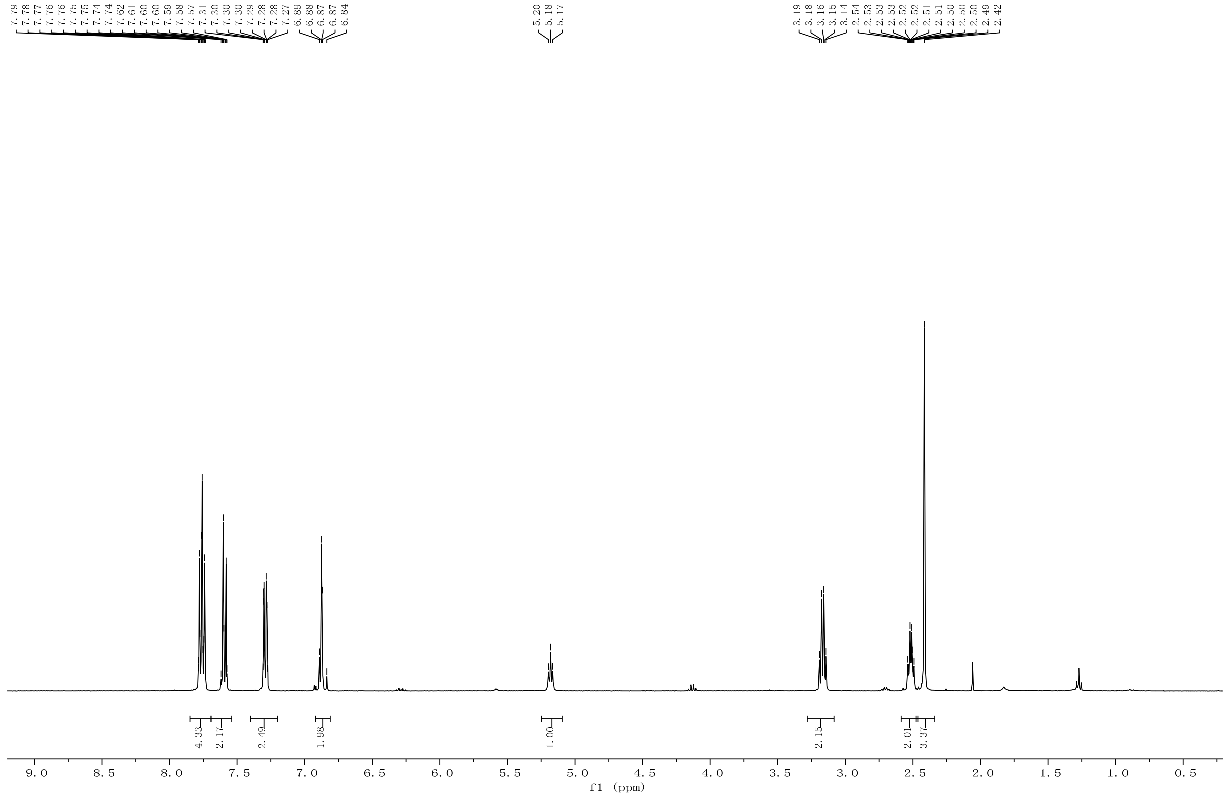

7.79 7.78 7.77 7.76 7.76 7.75 7.75 7.74 7.74 7.62 7.61 7.60 7.60 7.59 7.58 7.57 7.31 7.30 7.30 7.30 7.29 7.28 7.28 7.27 6.89 6.88 6.87 6.87 6.84
5.20 5.18 5.17
3.19 3.18 3.16 3.15 3.14 2.54 2.53 2.53 2.53 2.52 2.52 2.51 2.51 2.50 2.50 2.50 2.49 2.42
4.33 2.17 2.49 1.98 1.00 2.15 2.01 3.37
f1 (ppm)


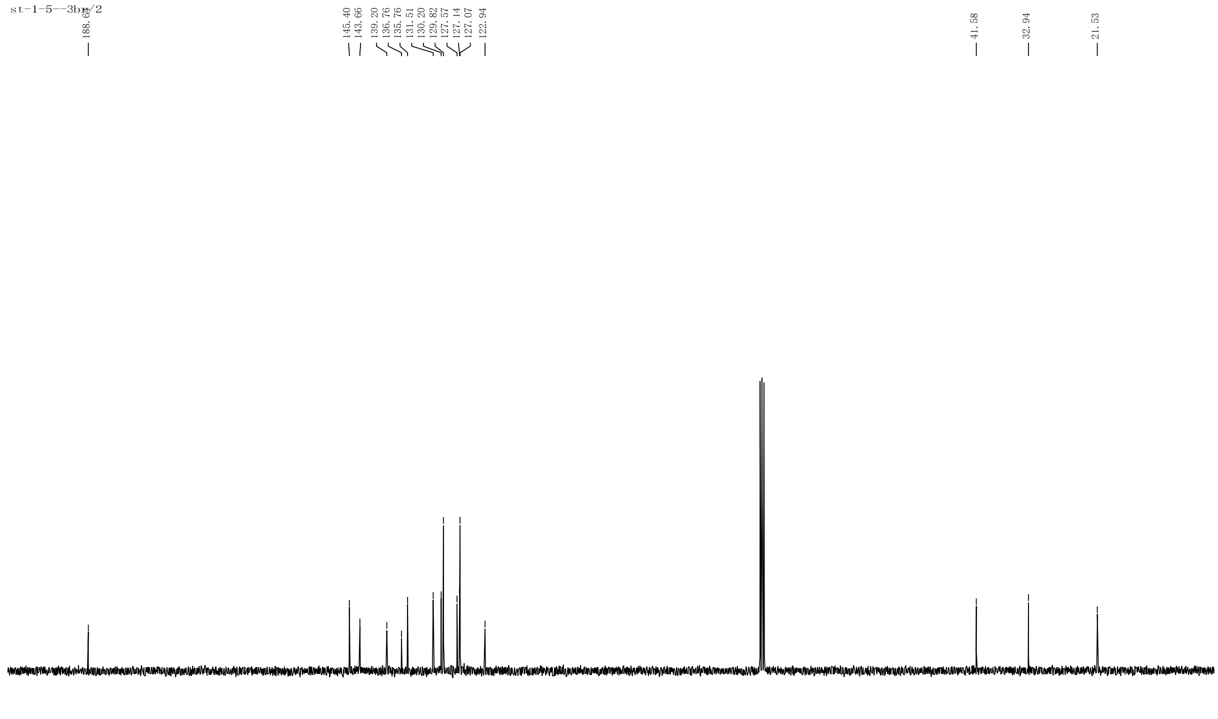

188.64
145.40 143.66 139.20 136.76 135.76 131.51 130.20 129.82 127.57 127.14 127.07 122.94
41.58
32.94
21.53
f1 (ppm)

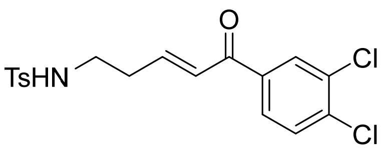
O
TsHN
Cl
Cl

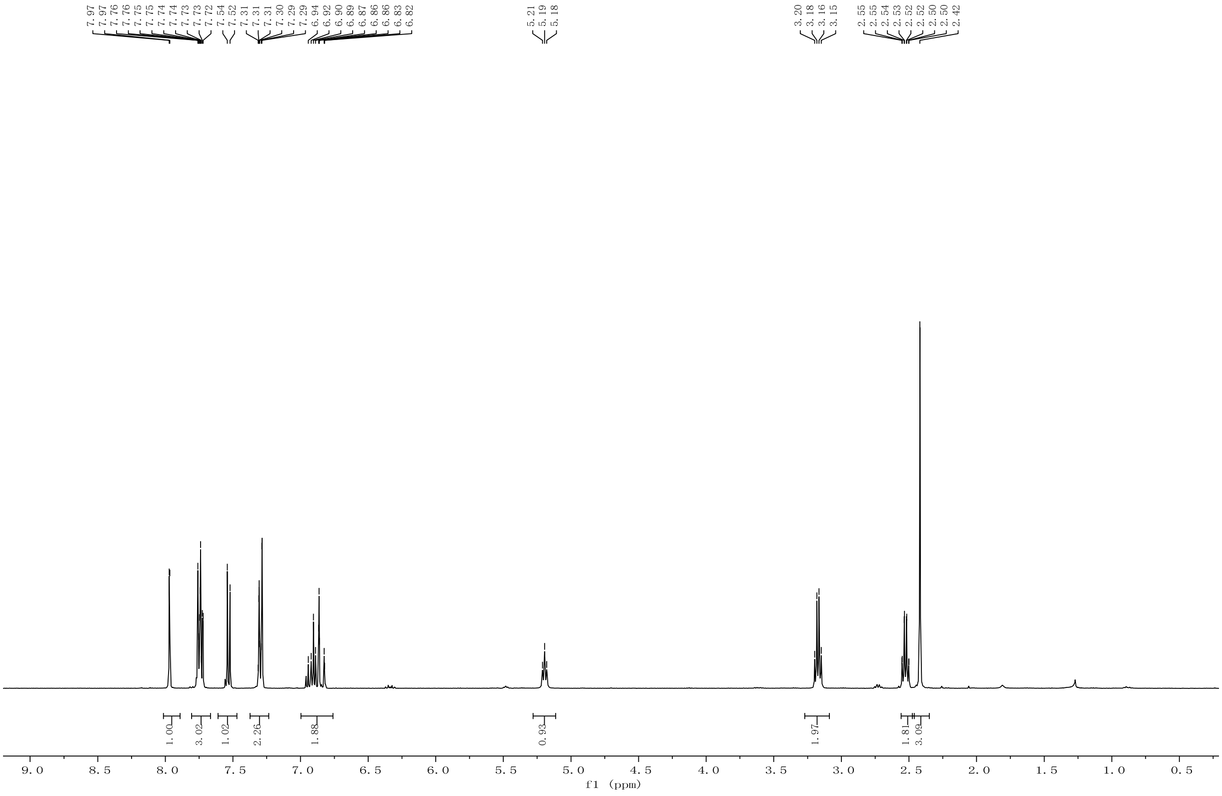
7.97 7.97 7.76 7.75 7.75 7.74 7.74 7.73 7.73 7.72 7.54 7.52 7.31 7.31 7.30 7.29 7.29 6.94 6.92 6.90 6.89 6.87 6.86 6.86 6.83 6.82
5.21 5.19 5.18
3.20 3.18 3.16 3.15
2.55 2.55 2.54 2.53 2.52 2.52 2.50 2.50 2.42
1.00 3.02 1.02 2.26 1.88 0.93 1.97 1.81 3.09
f1 (ppm)

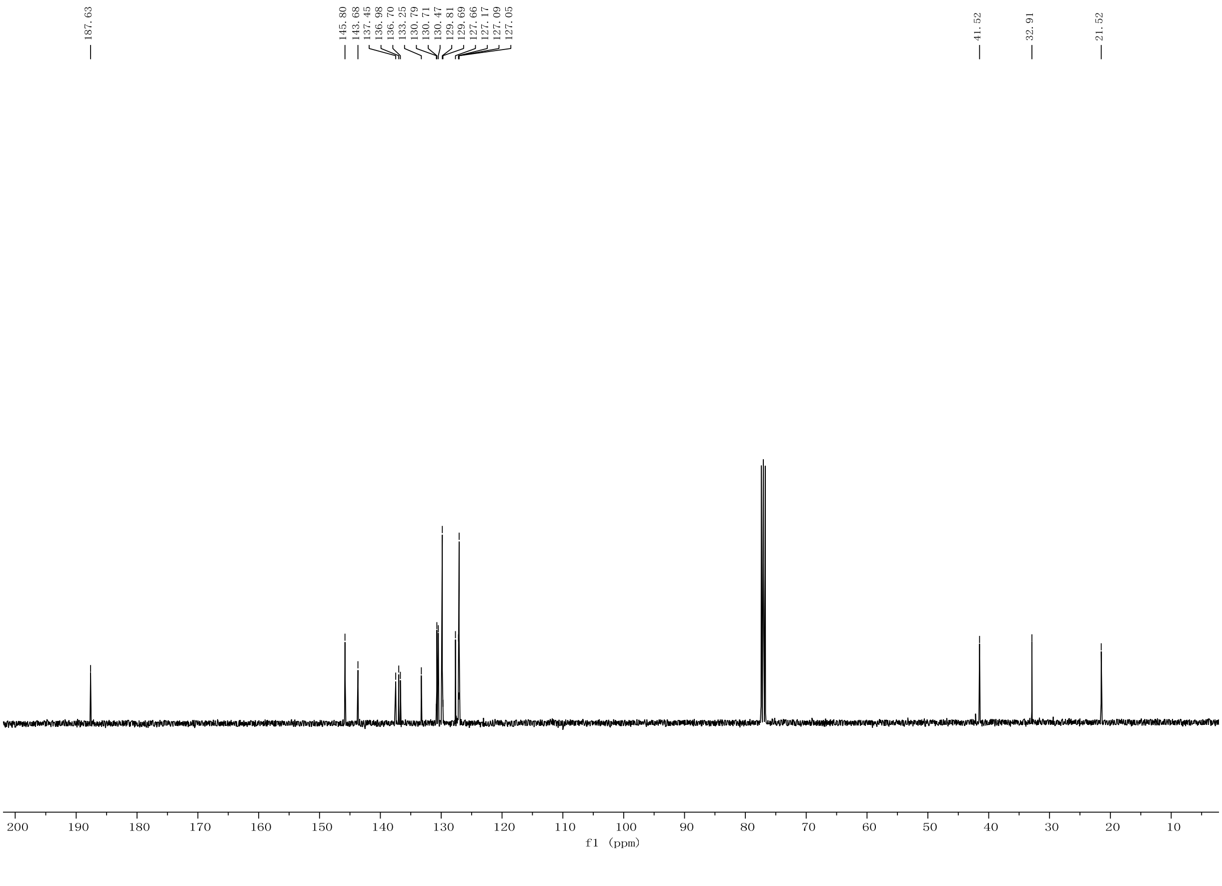
187.63
145.80 143.68 137.45 136.98 136.70 133.25 130.79 130.71 130.47 129.81 129.69 127.66 127.17 127.09 127.05
41.52
32.91
21.52
f1 (ppm)

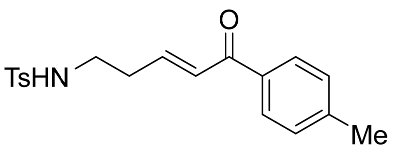
O
TsHN
Me

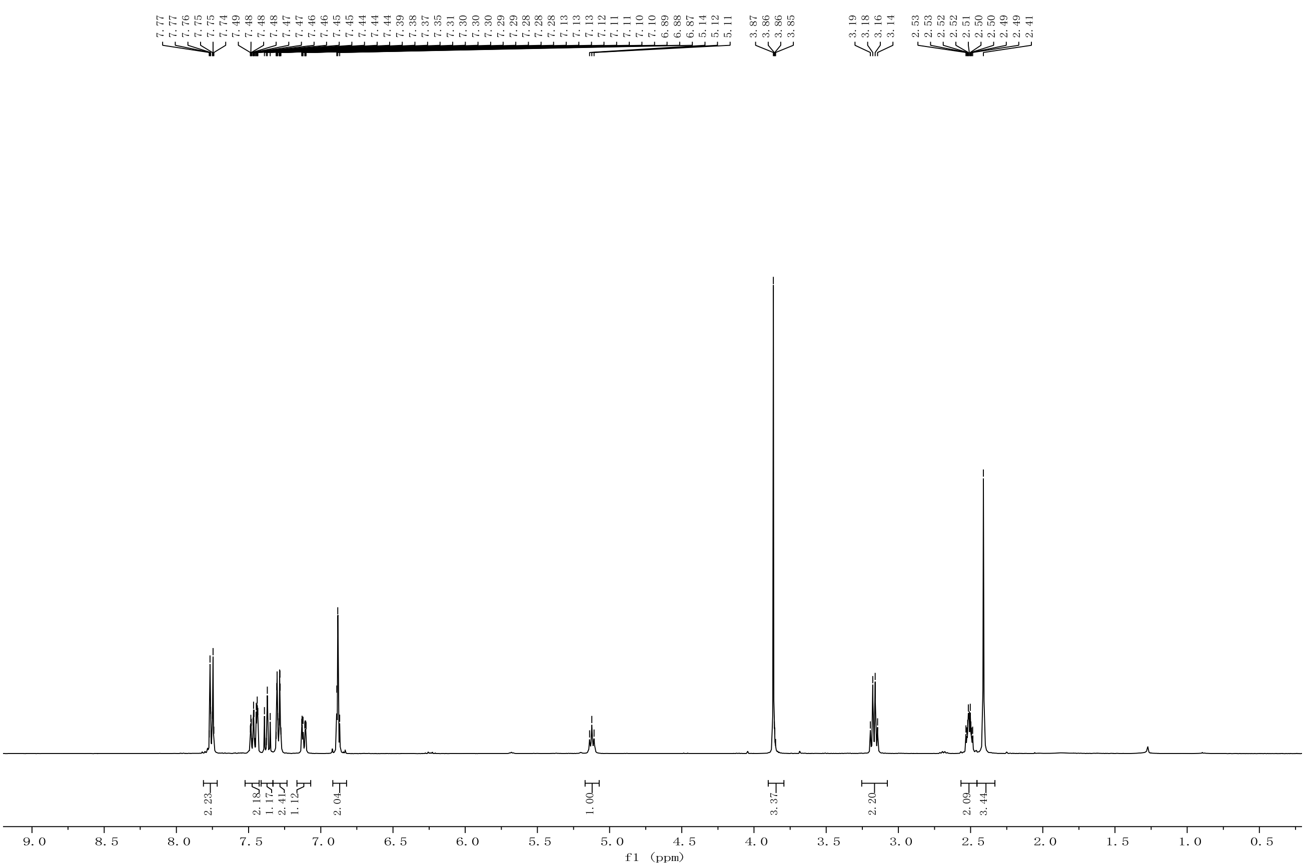
7.77 7.77 7.76 7.75 7.75 7.74 7.49 7.48 7.48 7.48 7.47 7.46 7.46 7.45 7.45 7.44 7.44 7.44 7.39 7.38 7.37 7.35 7.31 7.30 7.30 7.30 7.29 7.29 7.28 7.28 7.28 7.28 7.13 7.13 7.13 7.12 7.11 7.11 7.10 7.10 6.89 6.88 6.87 5.14 5.12 5.11
3.87 3.86 3.86 3.85
3.19 3.18 3.16 3.14
2.53 2.53 2.52 2.52 2.51 2.50 2.50 2.49 2.49 2.41
2.23 2.18 1.17 2.41 1.12 2.04 1.00 3.37 2.20 2.09 3.44
9.0 8.5 8.0 7.5 7.0 6.5 6.0 5.5 5.0 4.5 4.0 3.5 3.0 2.5 2.0 1.5 1.0 0.5
f1 (ppm)

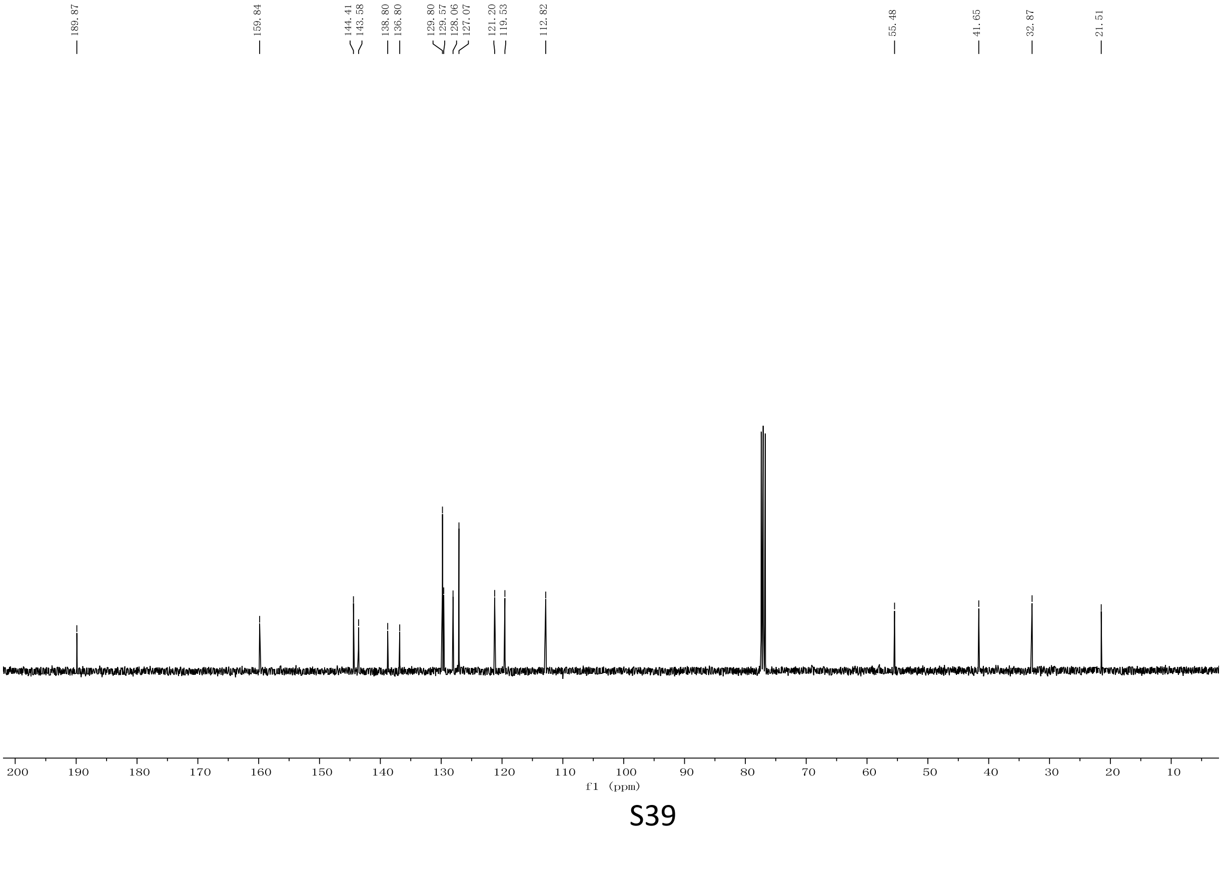
189.87
159.84
144.41 143.58
138.80 136.80
129.80 129.57 128.06 127.07
121.20 119.53
112.82
55.48
41.65
32.87
21.51
200 190 180 170 160 150 140 130 120 110 100 90 80 70 60 50 40 30 20 10
f1 (ppm)

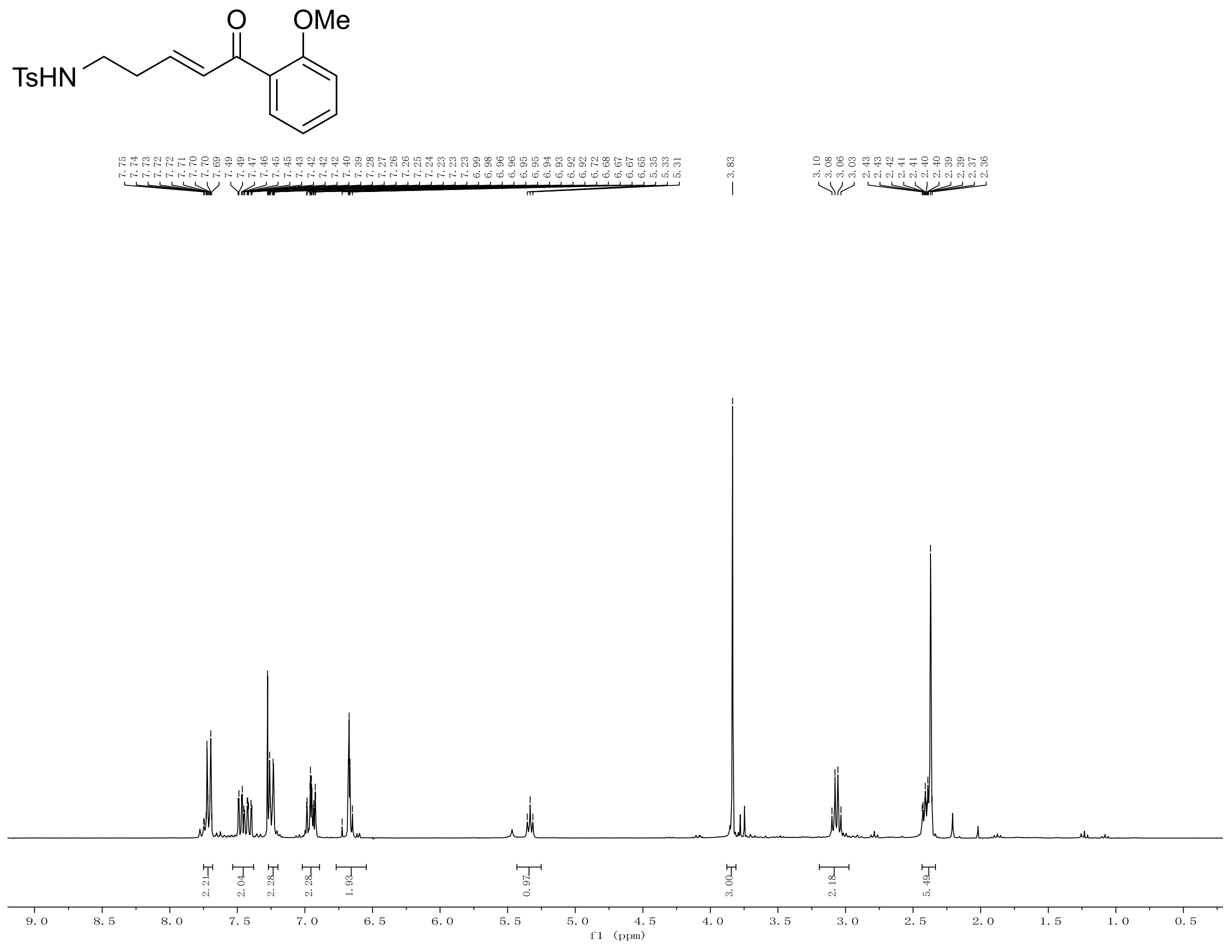

TsHN
O
OMe
7.75 7.74 7.73 7.72 7.72 7.71 7.70 7.70 7.69 7.49 7.49 7.47 7.46 7.45 7.45 7.43 7.42 7.42 7.42 7.40 7.39 7.28 7.27 7.26 7.26 7.25 7.24 7.23 7.23 7.23 6.99 6.98 6.96 6.96 6.95 6.95 6.94 6.93 6.92 6.92 6.72 6.68 6.67 6.67 6.65 6.65 5.35 5.33 5.31
3.83
3.10 3.08 3.06 3.03 2.43 2.43 2.42 2.41 2.41 2.40 2.40 2.39 2.39 2.37 2.36
2.21 2.04 2.28 2.28 1.93 0.97 3.00 2.18 5.49
9.0 8.5 8.0 7.5 7.0 6.5 6.0 5.5 5.0 4.5 4.0 3.5 3.0 2.5 2.0 1.5 1.0 0.5
f1 (ppm)


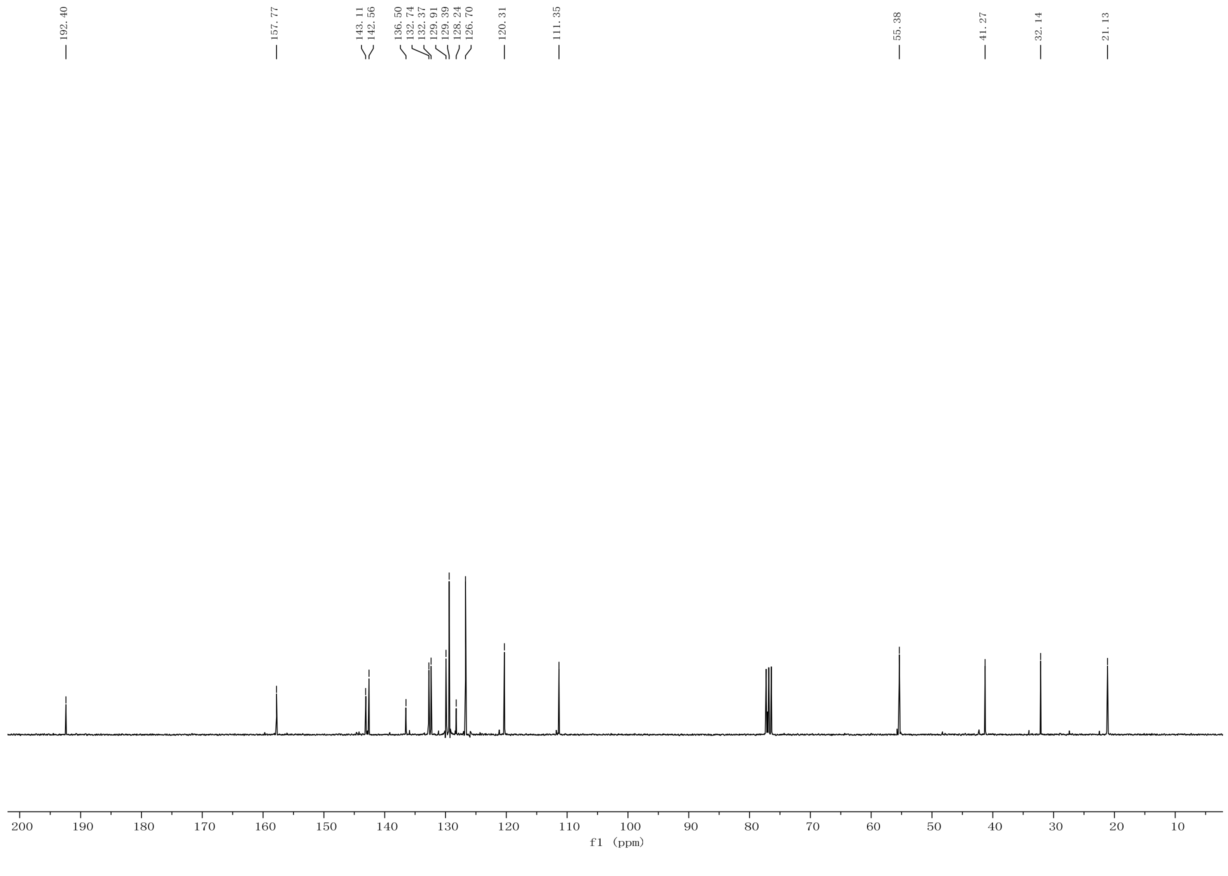

192.40
157.77
143.11 142.56
136.50 132.74 132.37 129.91 129.39 128.24 126.70
120.31
111.35
55.38
41.27
32.14
21.13
200 190 180 170 160 150 140 130 120 110 100 90 80 70 60 50 40 30 20 10
f1 (ppm)

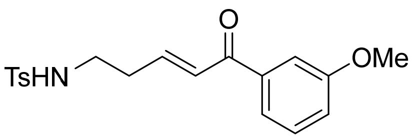
O
TsHN
OMe

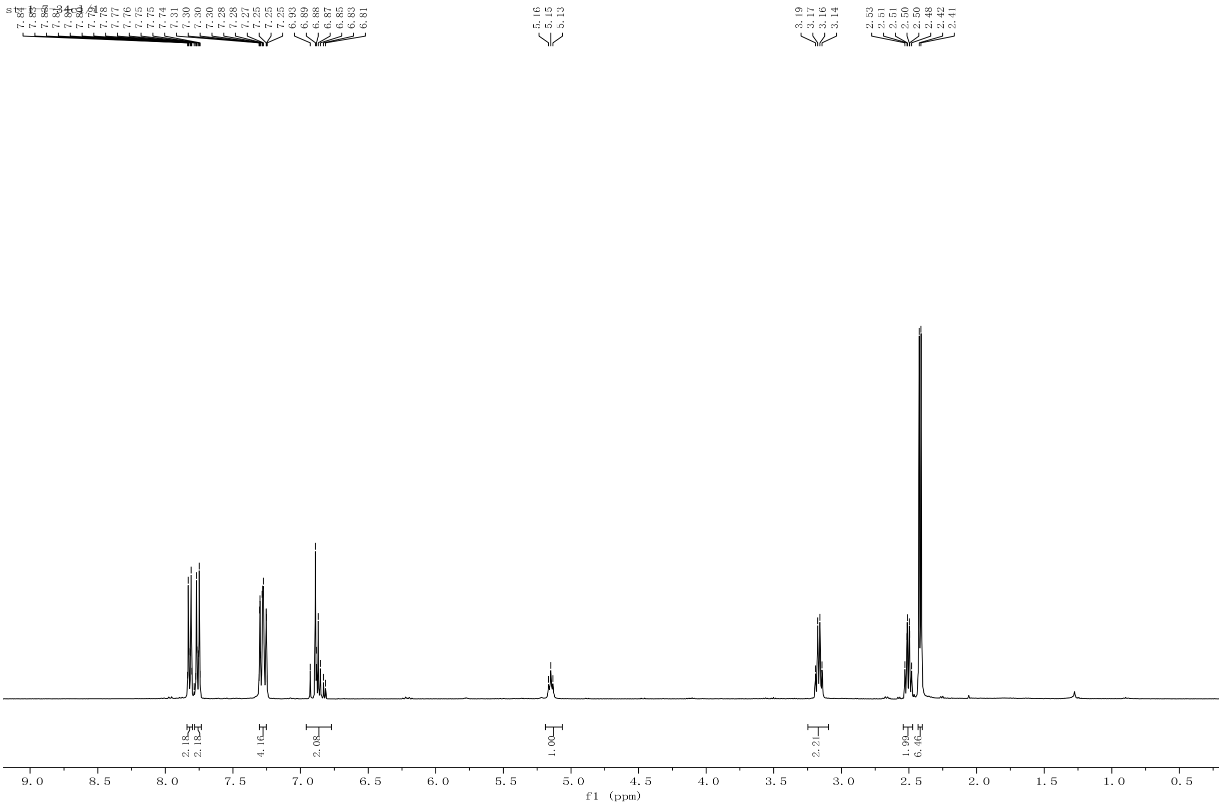
5.16
5.15
5.13
3.19
3.17
3.16
3.14
2.53
2.51
2.51
2.50
2.50
2.48
2.42
2.41
2.18
2.18
4.16
2.08
1.00
2.21
1.99
6.46
f1 (ppm)

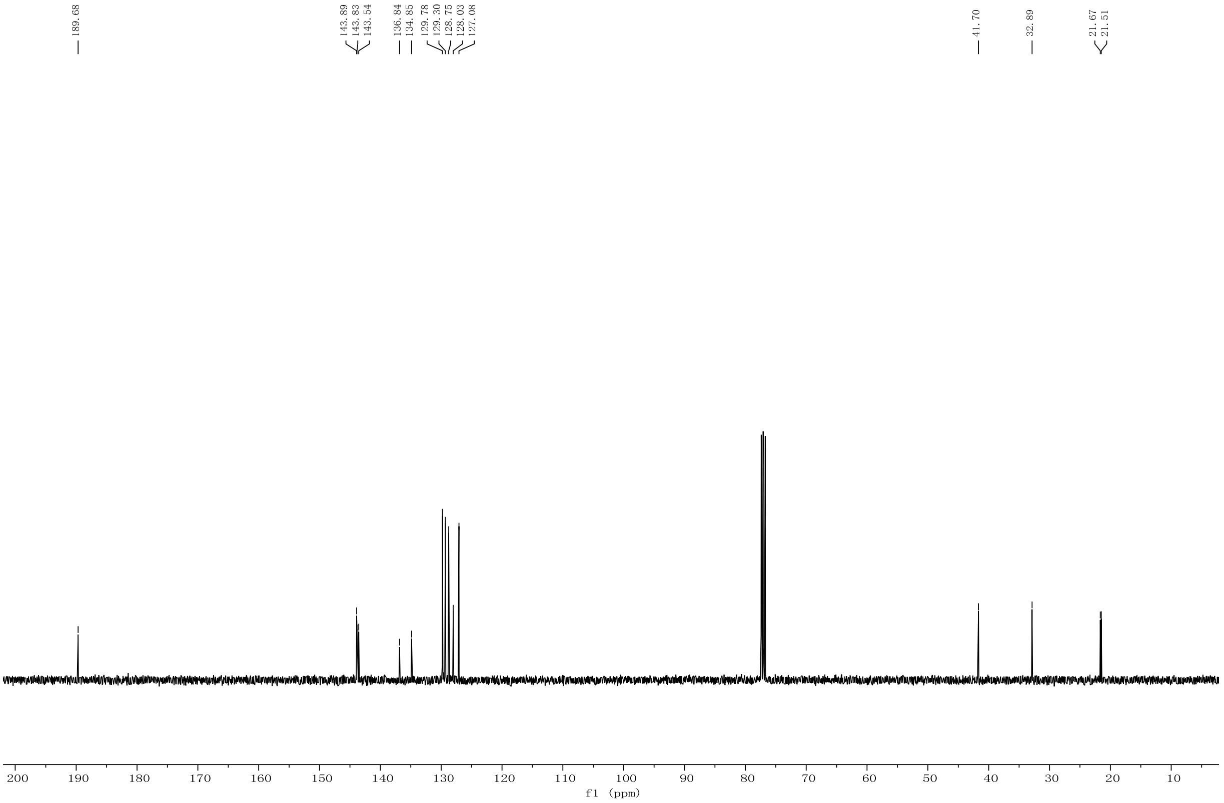
189.68
143.89
143.83
143.54
136.84
134.85
129.78
129.30
128.75
128.03
127.08
41.70
32.89
21.67
21.51
f1 (ppm)

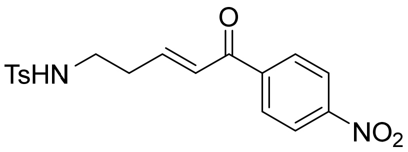
O
TsHN
NO2

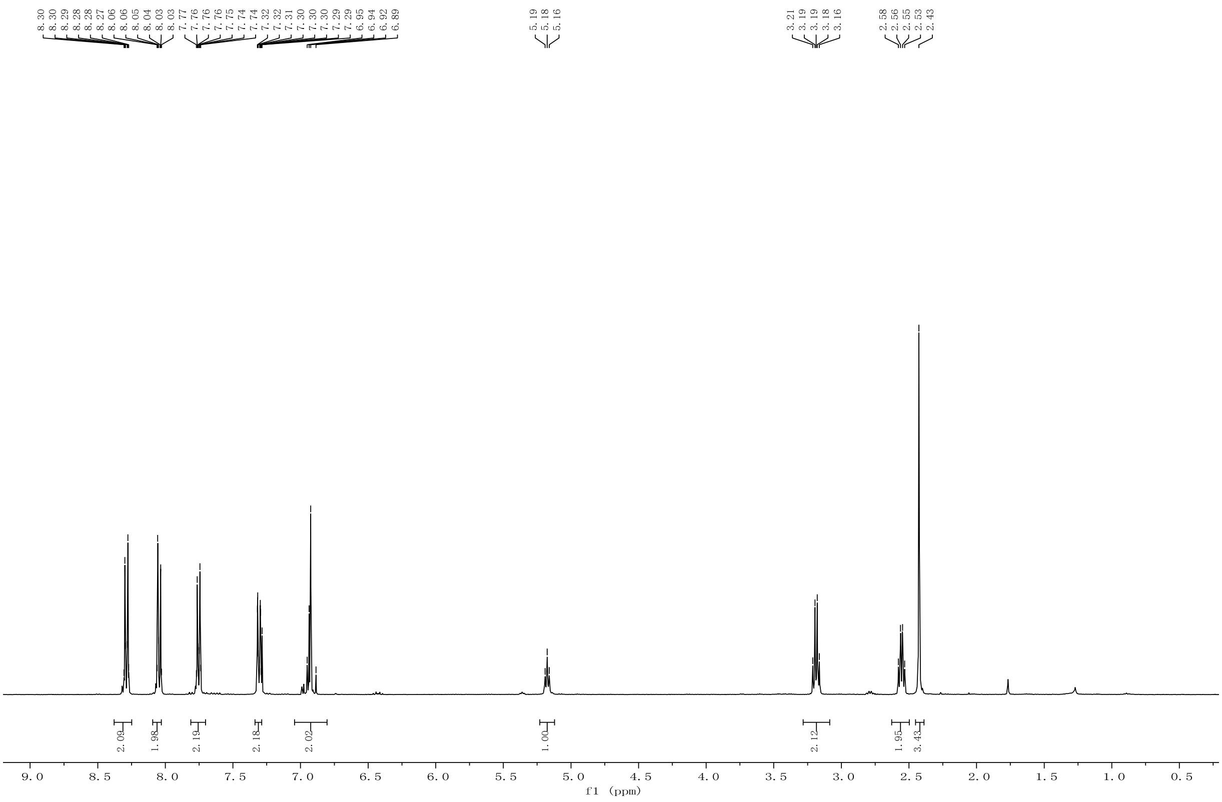

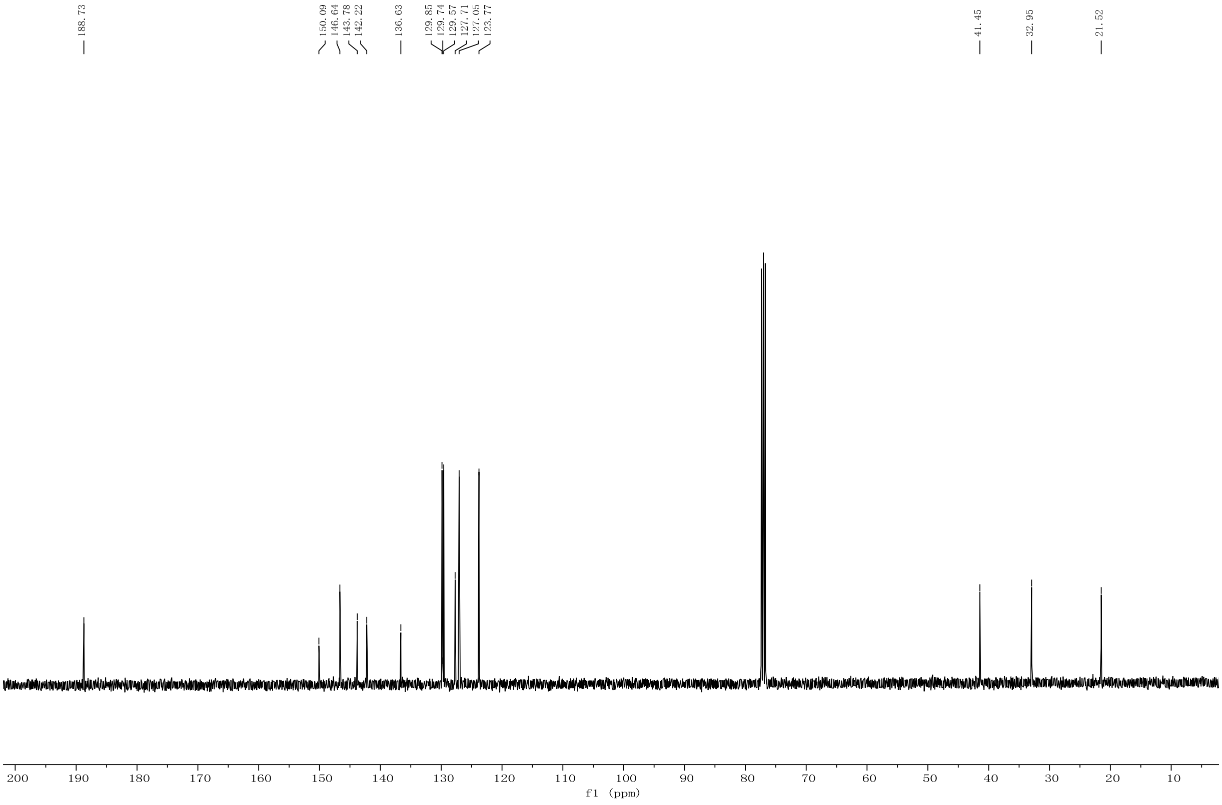

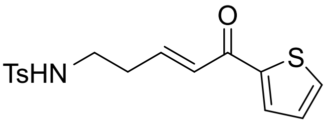

O
TsHN
S


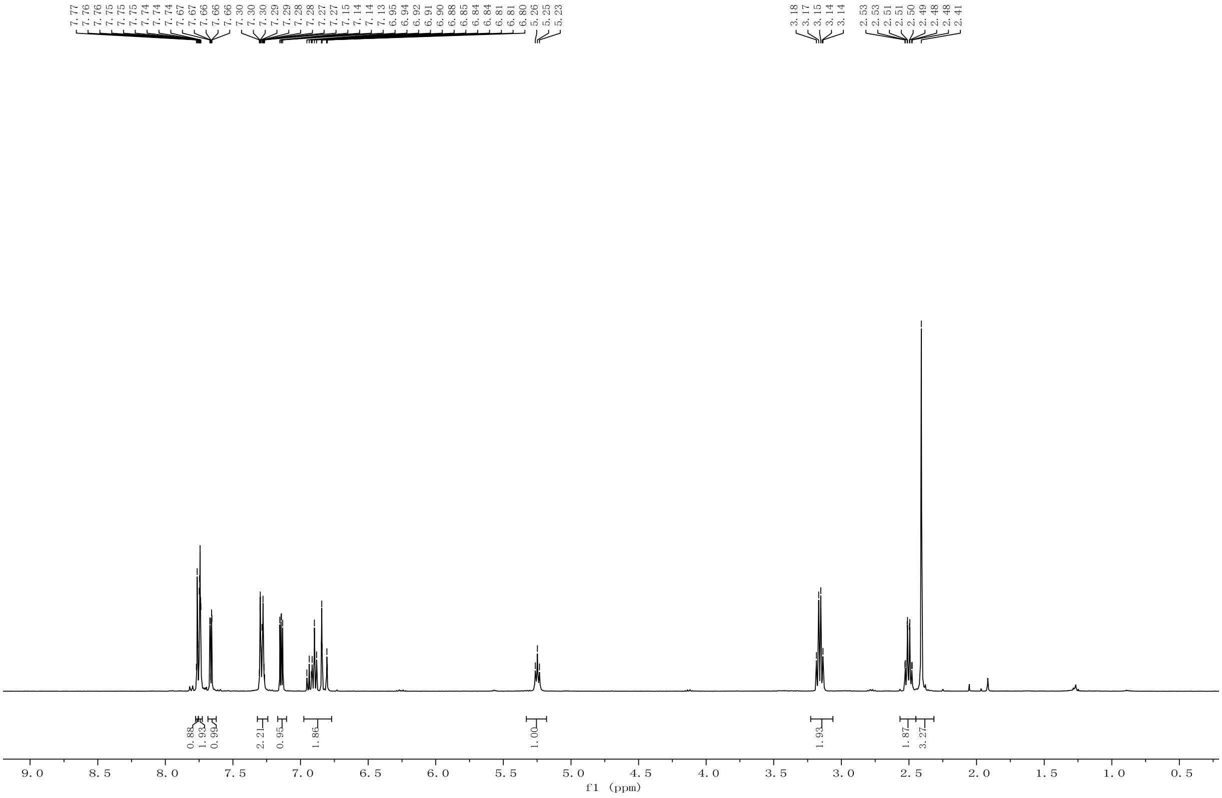

7.77 7.76 7.75 7.75 7.74 7.74 7.74 7.67 7.67 7.66 7.66 7.30 7.30 7.29 7.29 7.28 7.27 7.27 7.15 7.14 7.14 7.13 6.95 6.94 6.92 6.91 6.90 6.88 6.85 6.84 6.84 6.81 6.81 6.80 5.26 5.25 5.23
3.18 3.17 3.15 3.14 3.14
2.53 2.53 2.51 2.51 2.50 2.49 2.48 2.48 2.41
0.88 1.93 0.99 2.21 0.95 1.86 1.00 1.93 1.87 3.27
9.0 8.5 8.0 7.5 7.0 6.5 6.0 5.5 5.0 4.5 4.0 3.5 3.0 2.5 2.0 1.5 1.0 0.5
f1 (ppm)


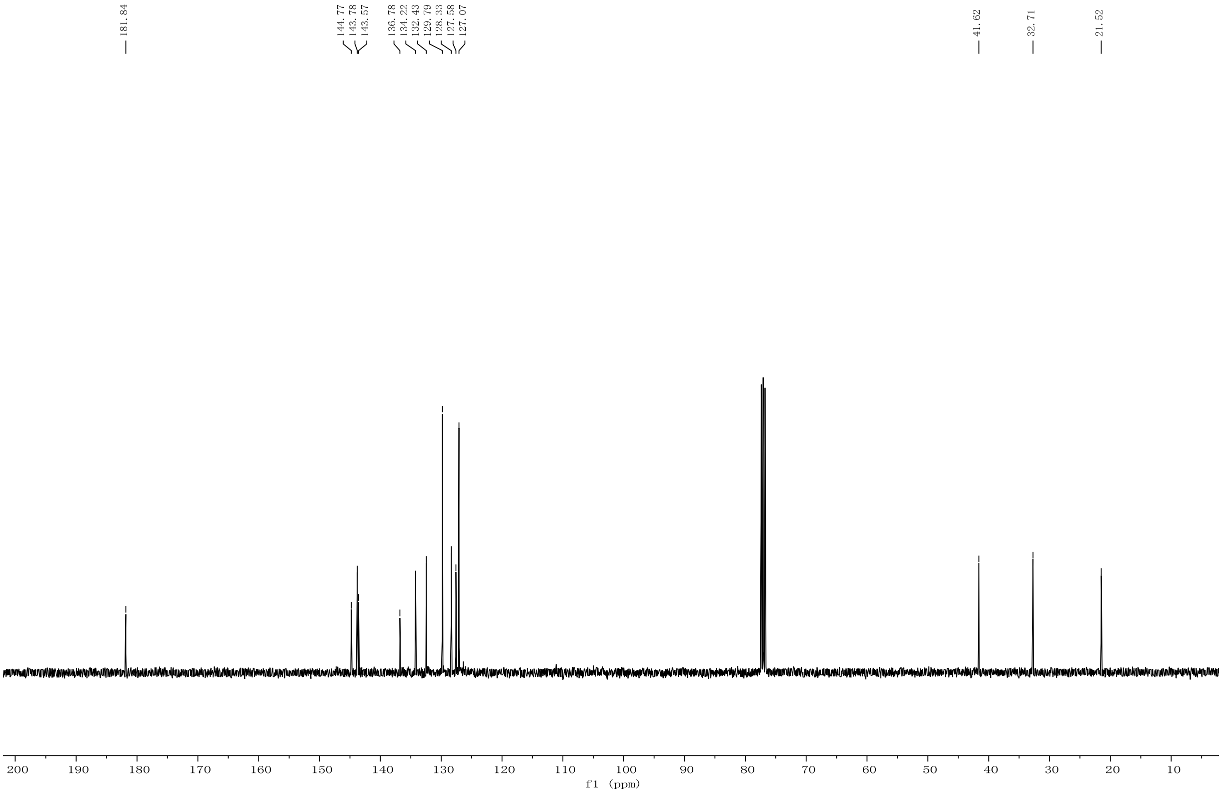

181.84
144.77 143.78 143.57
136.78 134.22 132.43 129.79 128.33 127.58 127.07
41.62
32.71
21.52
200 190 180 170 160 150 140 130 120 110 100 90 80 70 60 50 40 30 20 10
f1 (ppm)

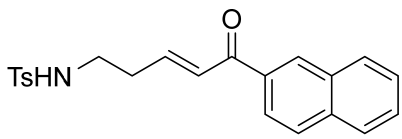
O
TsHN

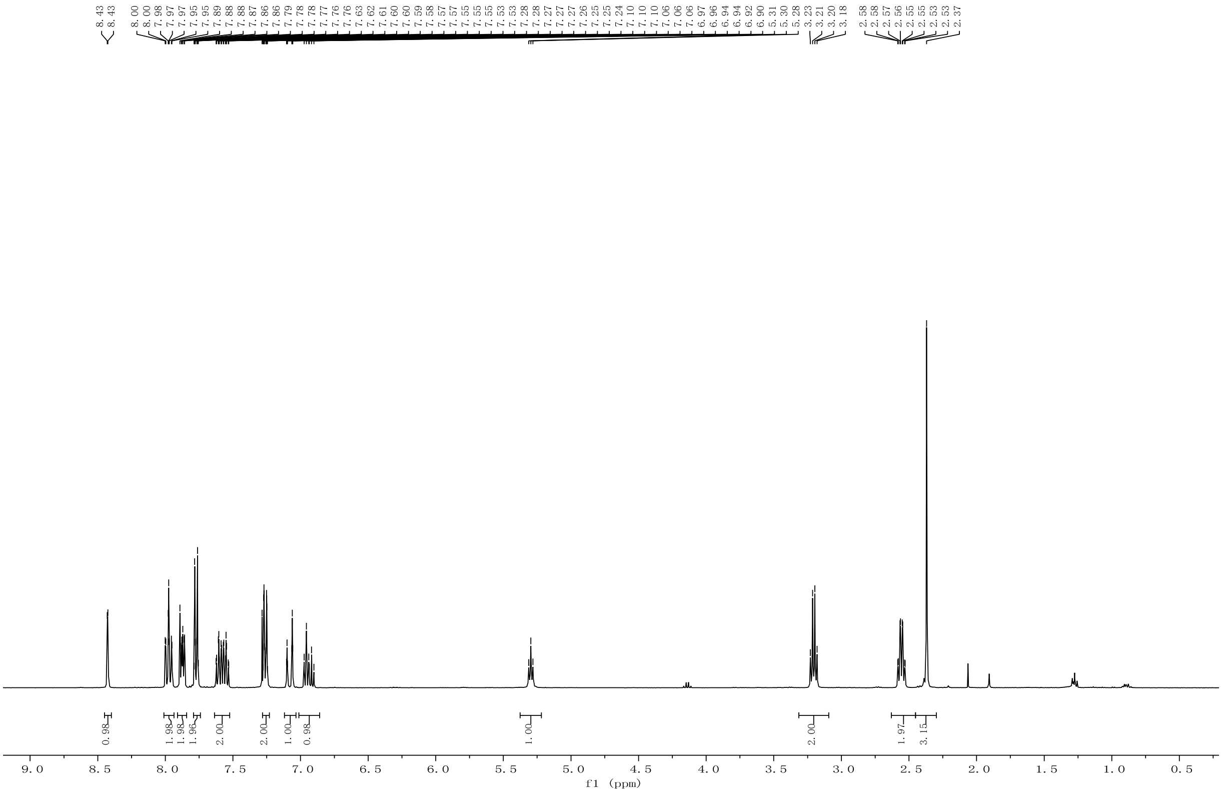
8.43
8.43
8.00
8.00
7.98
7.97
7.97
7.95
7.95
7.89
7.88
7.88
7.87
7.86
7.86
7.79
7.78
7.78
7.77
7.76
7.76
7.63
7.62
7.61
7.60
7.59
7.58
7.57
7.57
7.55
7.55
7.55
7.53
7.53
7.28
7.28
7.27
7.27
7.27
7.26
7.25
7.25
7.24
7.10
7.10
7.10
7.06
7.06
7.06
6.97
6.96
6.94
6.92
6.90
5.31
5.30
5.28
3.23
3.21
3.20
3.18
2.58
2.58
2.57
2.56
2.55
2.55
2.53
2.53
2.37
0.98
1.98
1.98
1.96
2.00
2.00
1.00
0.98
1.00
2.00
1.97
3.15
9.0
8.5
8.0
7.5
7.0
6.5
6.0
5.5
5.0
4.5
4.0
3.5
3.0
2.5
2.0
1.5
1.0
0.5
f1 (ppm)

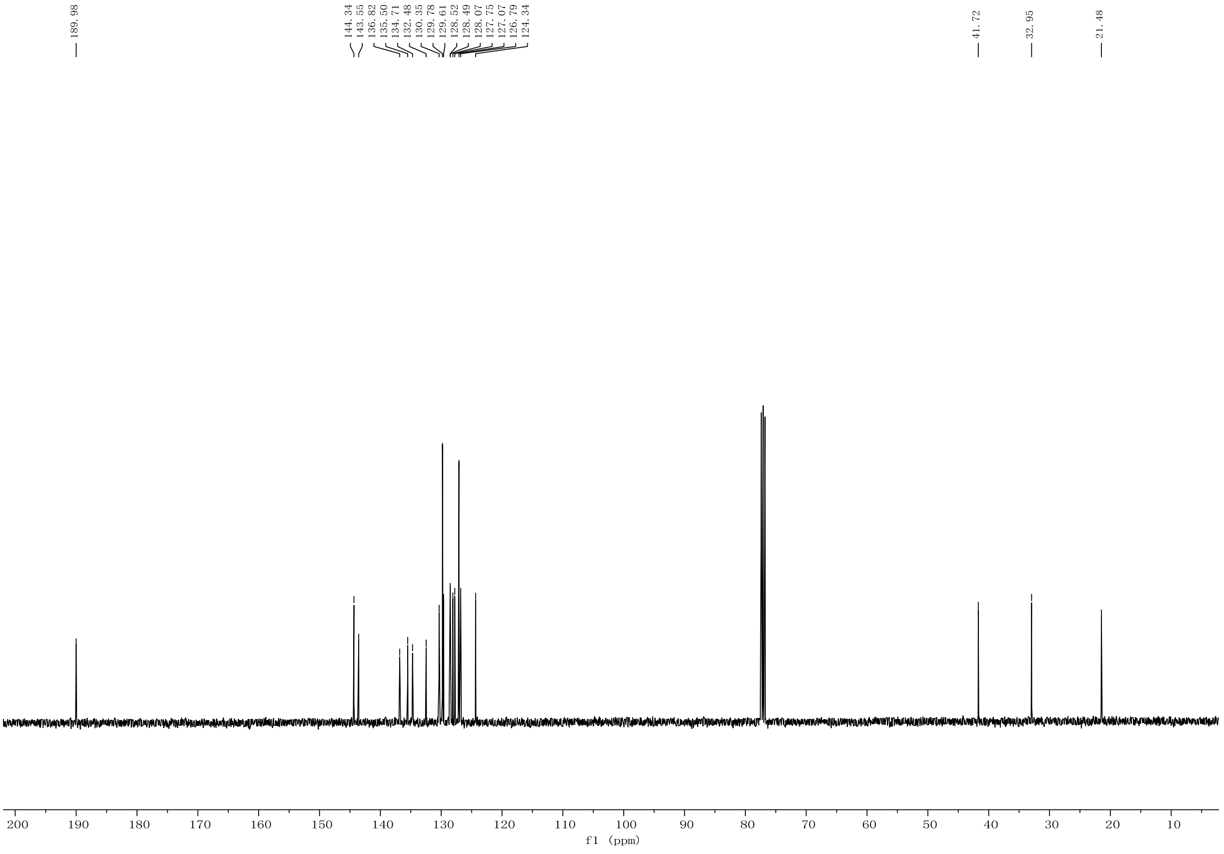
189.98
144.34
143.55
136.82
135.50
134.71
132.48
130.35
129.78
129.61
128.52
128.49
128.07
127.75
127.07
126.79
124.34
41.72
32.95
21.48
200
190
180
170
160
150
140
130
120
110
100
90
80
70
60
50
40
30
20
10
f1 (ppm)

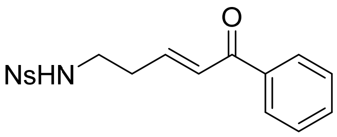
O
NsHN

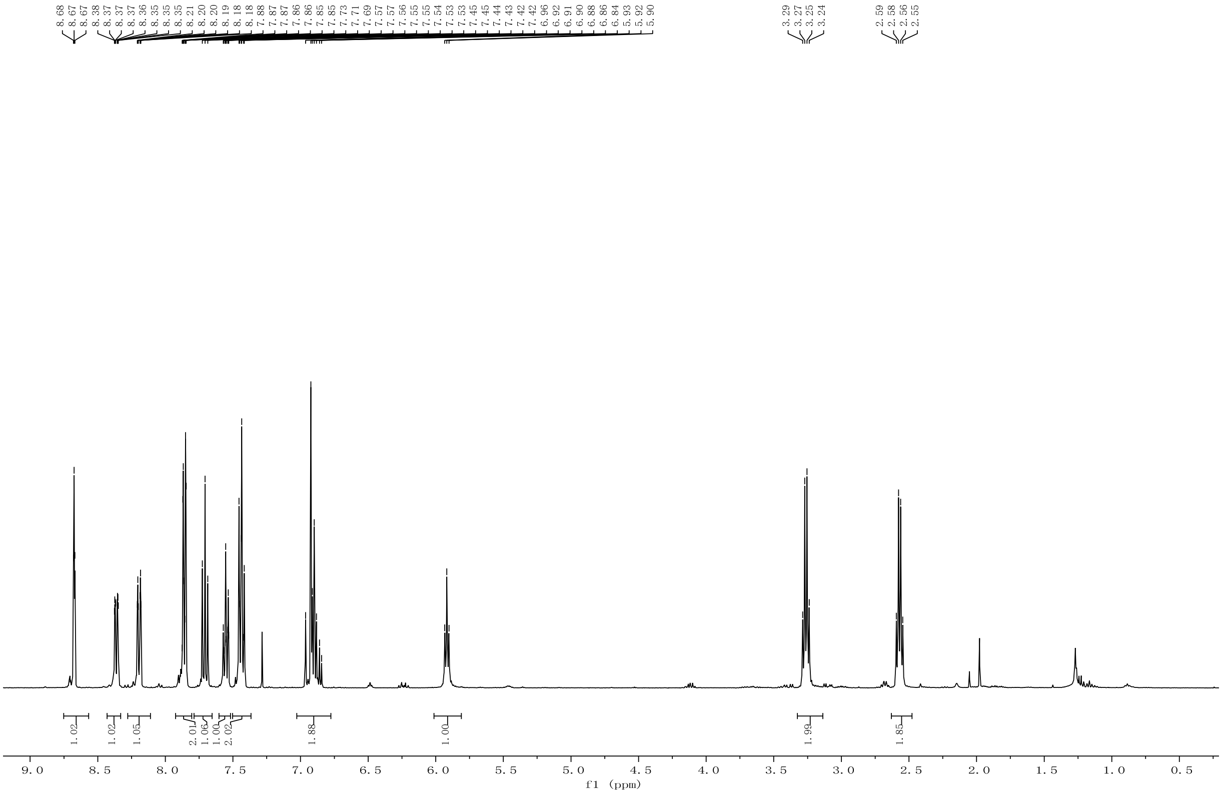
8.68 8.67 8.67 8.38 8.37 8.37 8.36 8.35 8.35 8.35 8.21 8.20 8.19 8.18 8.18 7.88 7.87 7.87 7.86 7.85 7.85 7.73 7.71 7.69 7.57 7.57 7.56 7.55 7.55 7.54 7.53 7.53 7.45 7.45 7.44 7.43 7.42 7.42 6.96 6.92 6.91 6.90 6.88 6.86 6.84 5.93 5.92 5.90
3.29 3.27 3.25 3.24
2.59 2.58 2.56 2.55
1.02 1.02 1.05 2.01 1.06 1.00 2.02 1.88 1.00 1.99 1.85
9.0 8.5 8.0 7.5 7.0 6.5 6.0 5.5 5.0 4.5 4.0 3.5 3.0 2.5 2.0 1.5 1.0 0.5
f1 (ppm)

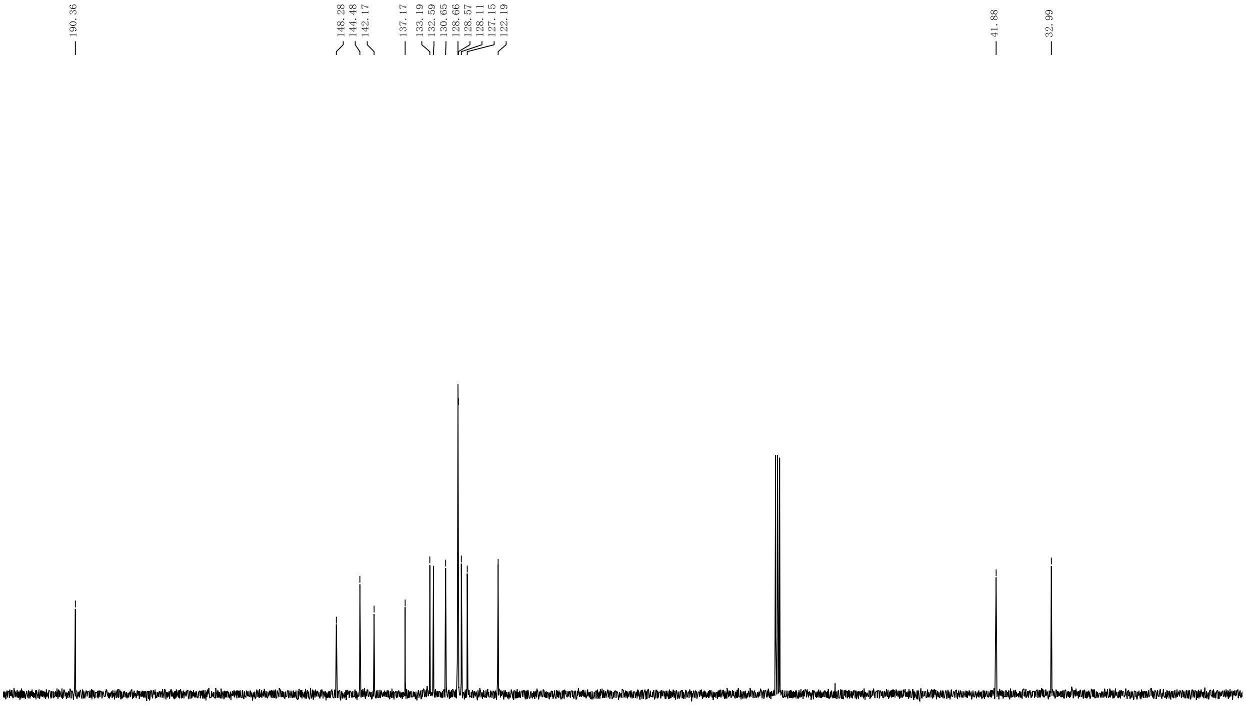
190.36
148.28 144.48 142.17
137.17 133.19 132.59 130.65 128.66 128.57 128.11 127.15 122.19
41.88
32.99
200 190 180 170 160 150 140 130 120 110 100 90 80 70 60 50 40 30 20 10
f1 (ppm)

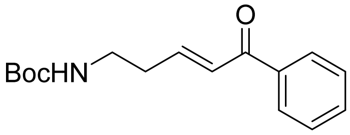
O
BocHN

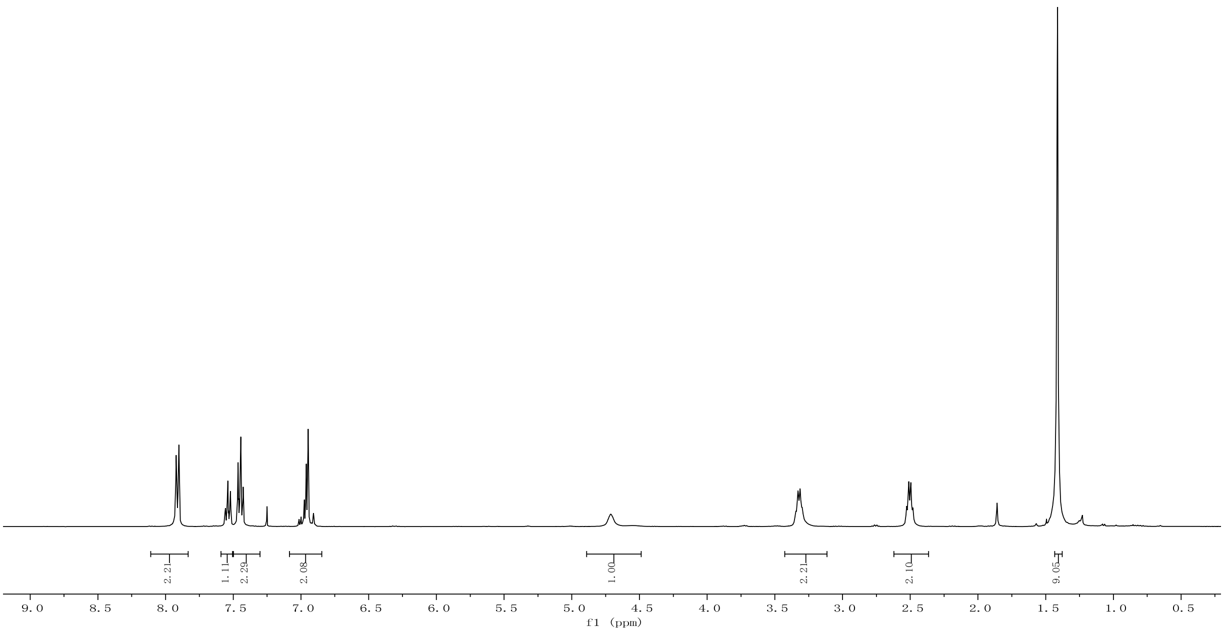
2.21
1.11
2.29
2.08
1.00
2.21
2.10
9.05
9.0 8.5 8.0 7.5 7.0 6.5 6.0 5.5 5.0 4.5 4.0 3.5 3.0 2.5 2.0 1.5 1.0 0.5
f1 (ppm)

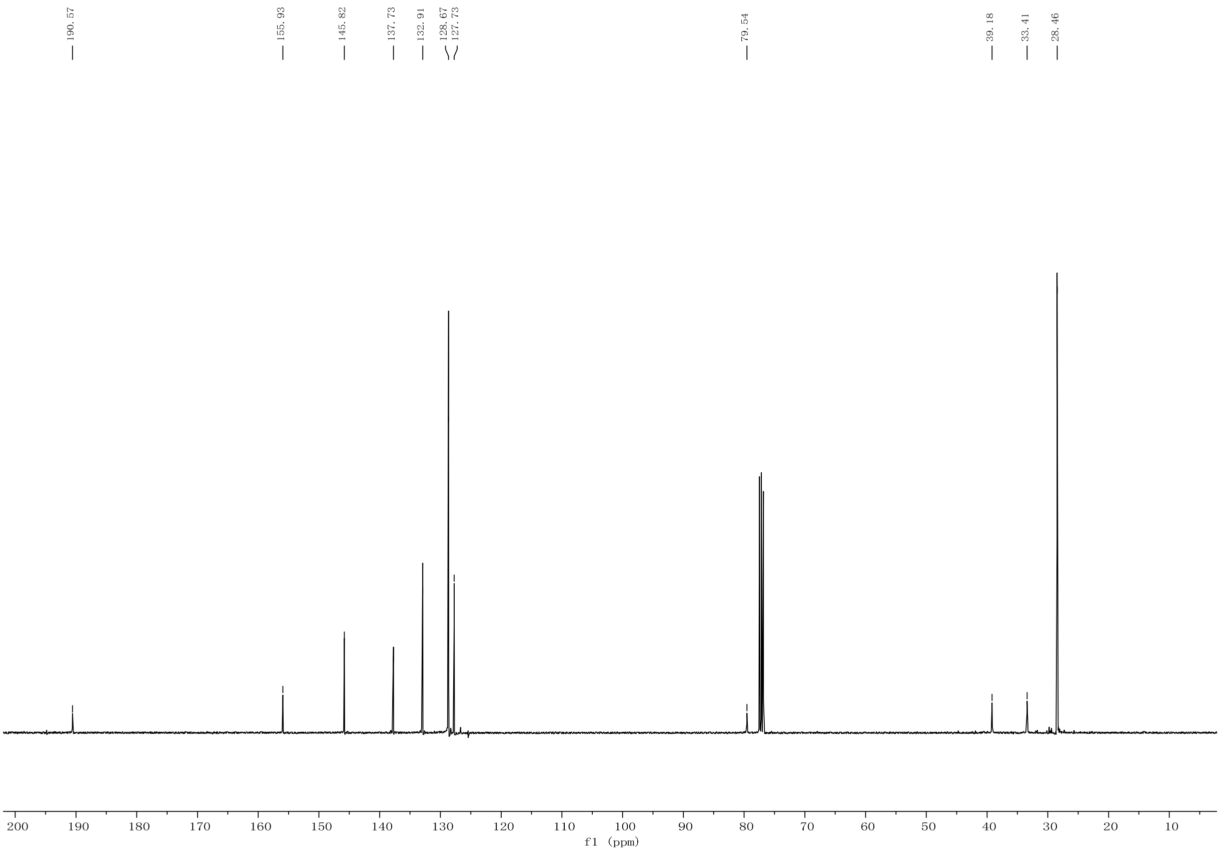
190.57
155.93
145.82
137.73
132.91
128.67
127.73
79.54
39.18
33.41
28.46
200 190 180 170 160 150 140 130 120 110 100 90 80 70 60 50 40 30 20 10
f1 (ppm)

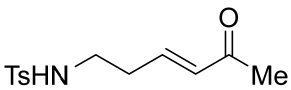
O
TsHN
Me

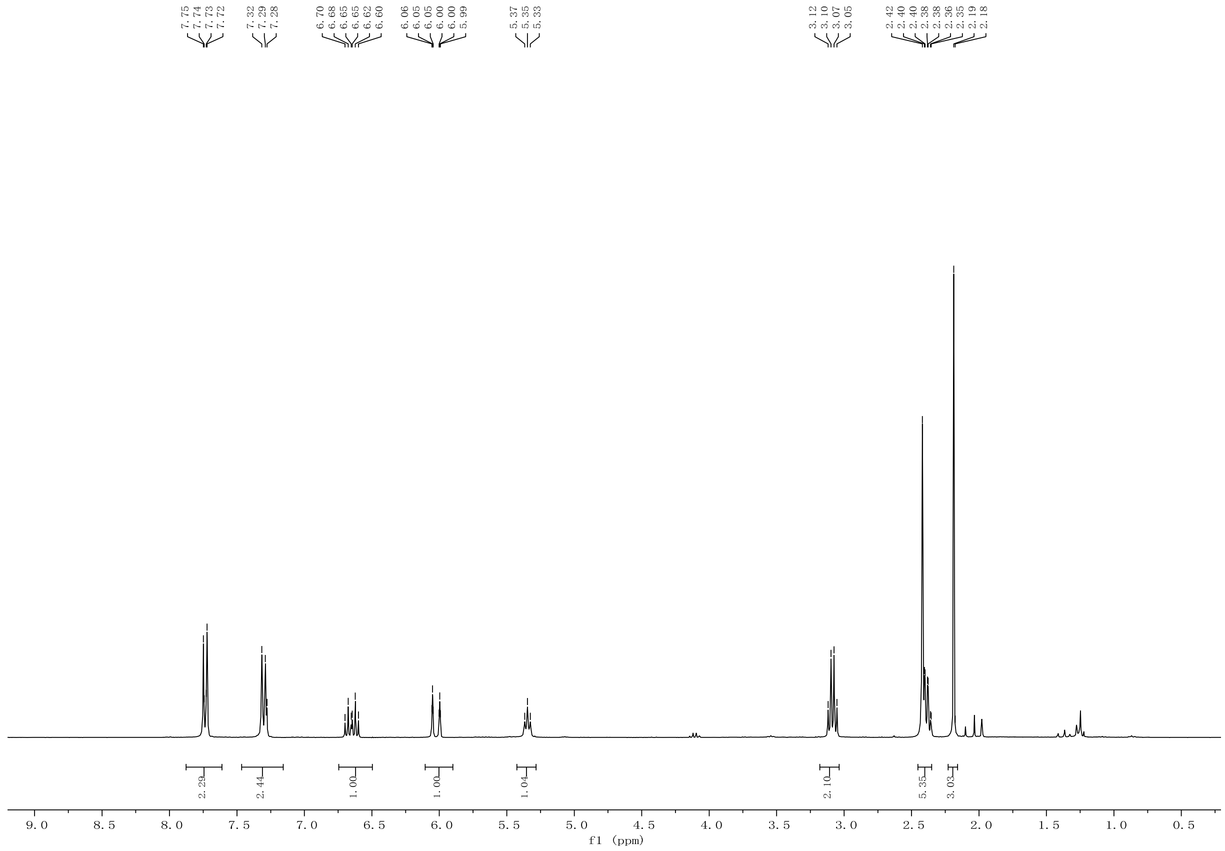
7.75
7.74
7.73
7.72
7.32
7.29
7.28
6.70
6.68
6.65
6.65
6.62
6.60
6.06
6.05
6.05
6.00
6.00
5.99
5.37
5.35
5.33
3.12
3.10
3.07
3.05
2.42
2.40
2.40
2.38
2.38
2.36
2.35
2.19
2.18
2.29
2.44
1.00
1.00
1.04
2.10
5.35
3.03
9.0
8.5
8.0
7.5
7.0
6.5
6.0
5.5
5.0
4.5
4.0
3.5
3.0
2.5
2.0
1.5
1.0
0.5
f1 (ppm)

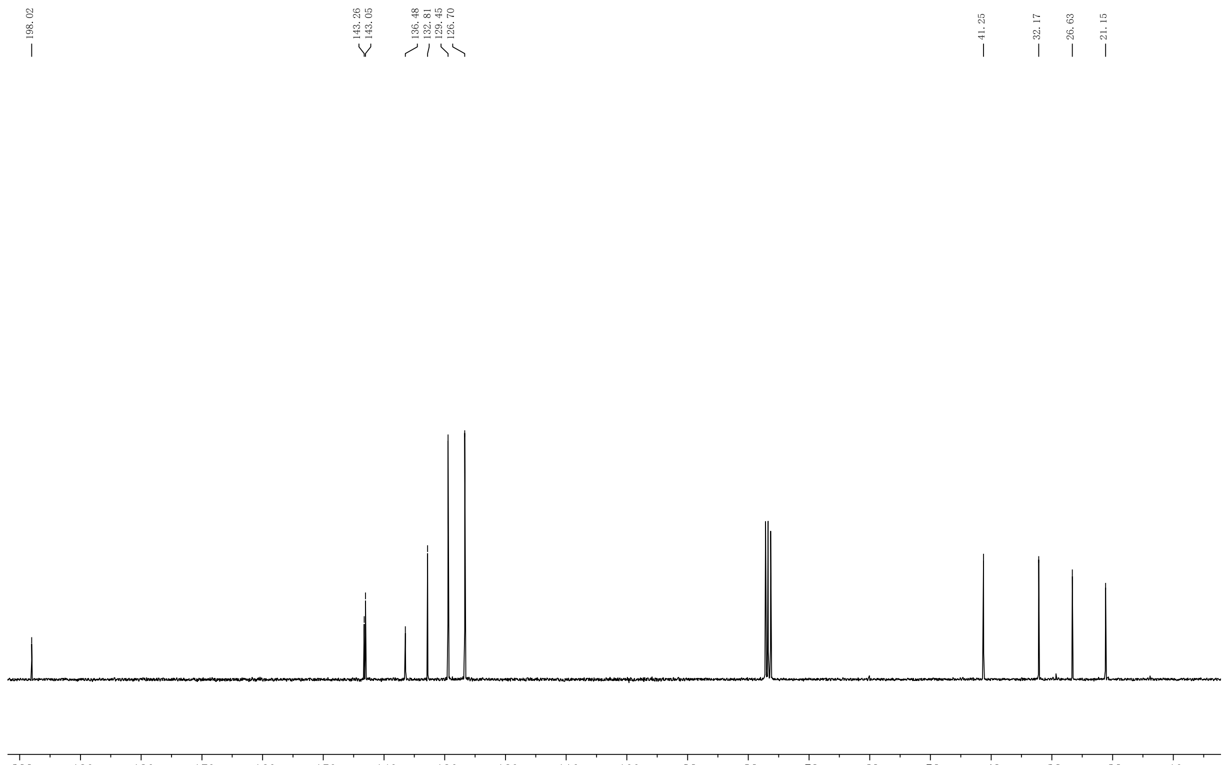
198.02
143.26
143.05
136.48
132.81
129.45
126.70
41.25
32.17
26.63
21.15
200
190
180
170
160
150
140
130
120
110
100
90
80
70
60
50
40
30
20
10
f1 (ppm)

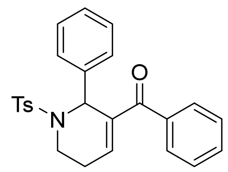

Ts
N
O


**3aa**

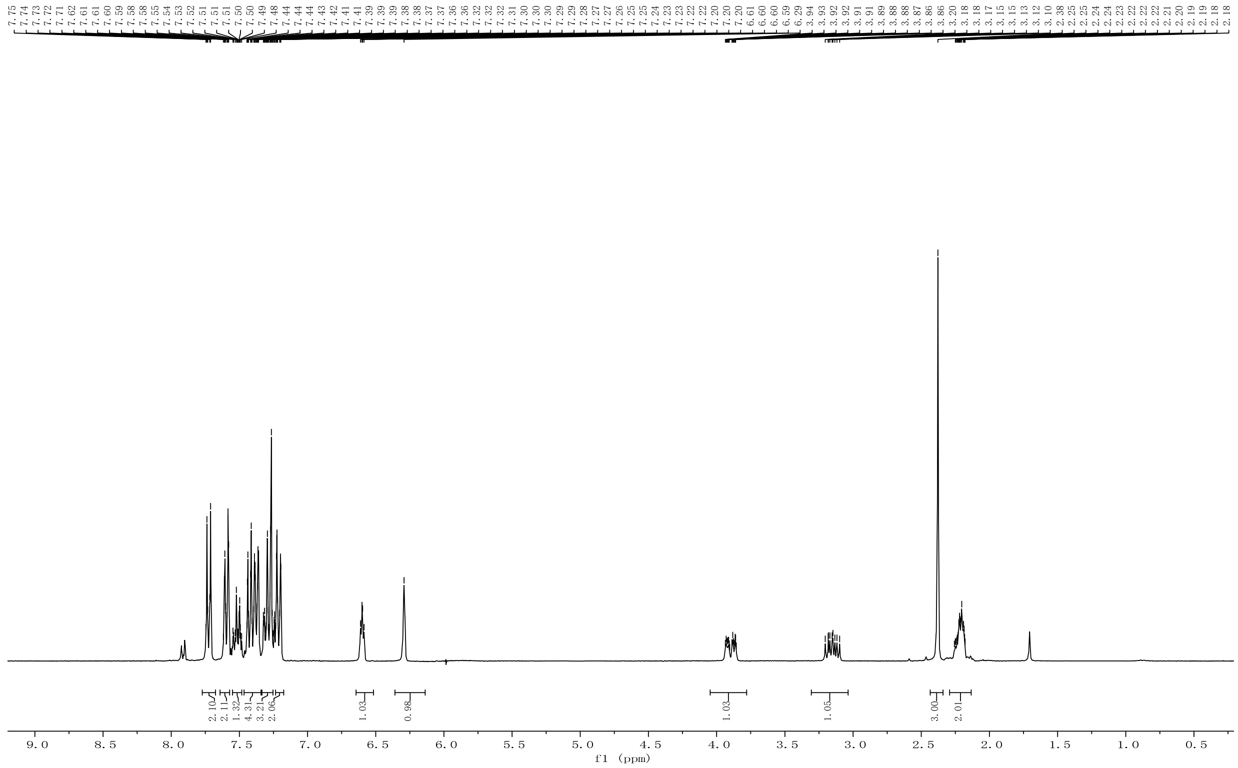

7.75 7.74 7.73 7.72 7.71 7.62 7.61 7.60 7.59 7.58 7.58 7.55 7.54 7.53 7.52 7.51 7.51 7.51 7.50 7.50 7.49 7.48 7.44 7.44 7.44 7.43 7.42 7.41 7.41 7.39 7.39 7.39 7.38 7.38 7.37 7.37 7.36 7.36 7.32 7.32 7.32 7.31 7.30 7.30 7.29 7.29 7.28 7.27 7.27 7.26 7.25 7.25 7.24 7.23 7.23 7.22 7.22 7.22 7.20 7.20 7.20 6.61 6.60 6.60 6.59 6.29 3.94 3.93 3.92 3.91 3.91 3.89 3.88 3.88 3.87 3.86 3.86 3.20 3.18 3.18 3.17 3.15 3.15 3.13 3.12 3.10 2.38 2.25 2.25 2.24 2.24 2.23 2.22 2.22 2.22 2.21 2.20 2.19 2.18 2.18
2.10 2.11 1.32 4.31 3.21 2.06 1.03 0.98 1.03 1.05 3.00 2.01
9.0 8.5 8.0 7.5 7.0 6.5 6.0 5.5 5.0 4.5 4.0 3.5 3.0 2.5 2.0 1.5 1.0 0.5
f1 (ppm)


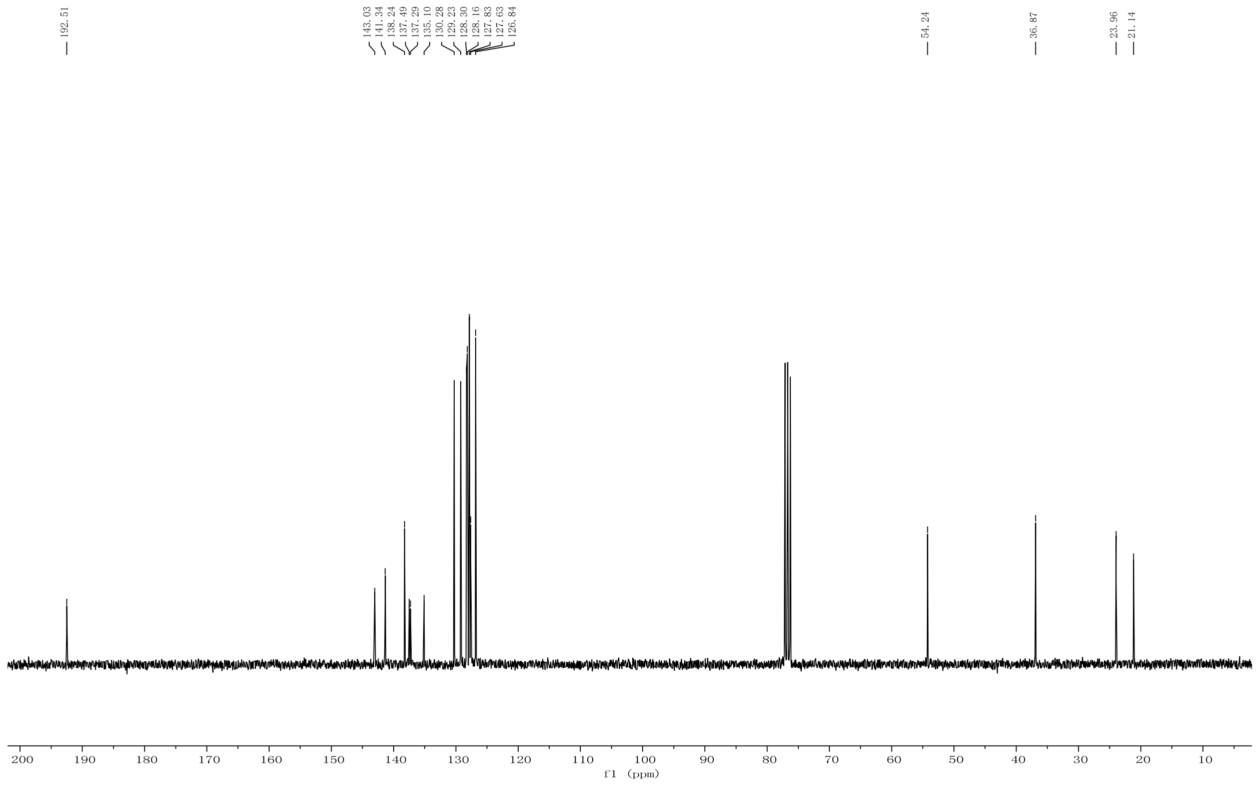

192.51
143.03 141.34 138.24 137.49 137.29 135.10 130.28 129.23 128.30 128.16 127.83 127.63 126.84
54.24
36.87
23.96 21.14
200 190 180 170 160 150 140 130 120 110 100 90 80 70 60 50 40 30 20 10
f1 (ppm)

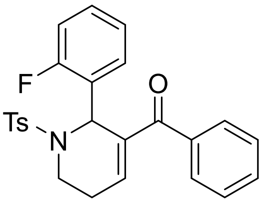

**3ab**

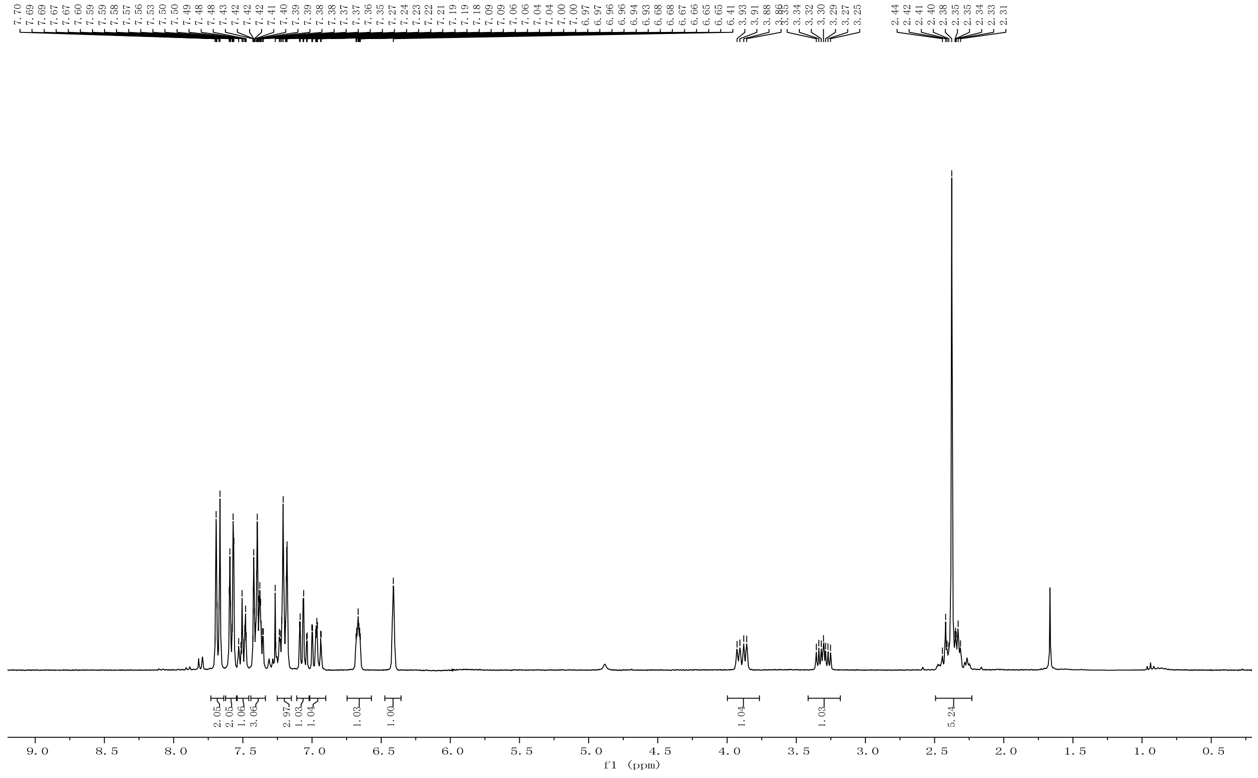

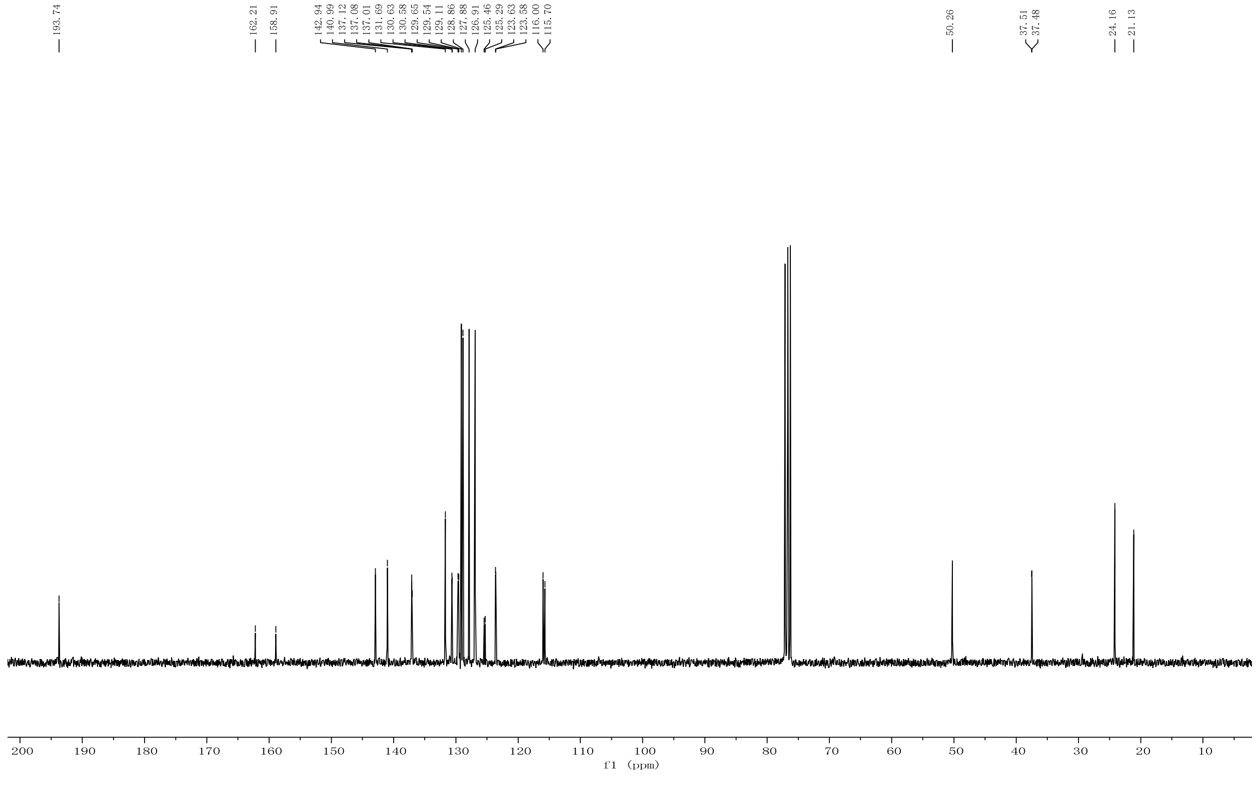

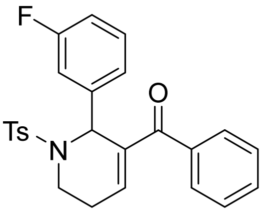
F
O
Ts
N

**3ac**

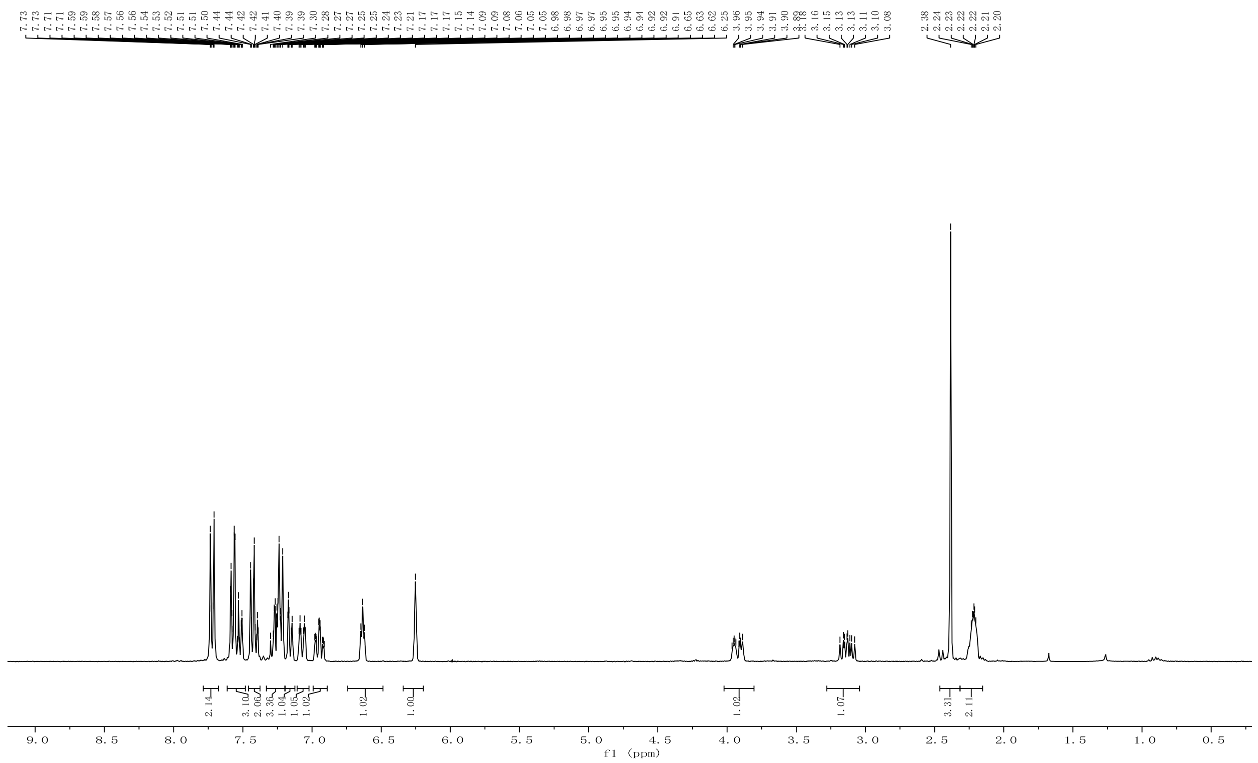
f1 (ppm)

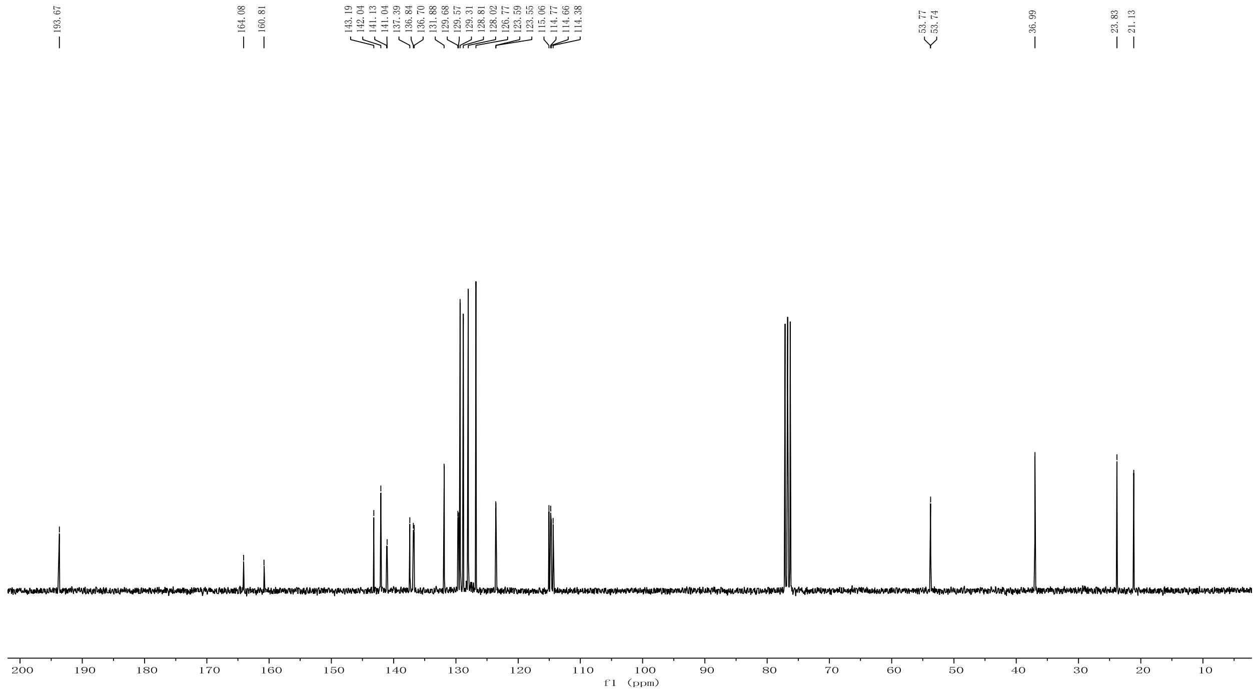
193.67
164.08
160.81
143.19
142.04
141.13
141.04
137.39
136.84
136.70
131.88
129.68
129.57
129.31
128.81
128.02
126.77
123.59
123.55
115.06
114.77
114.66
114.38
53.77
53.74
36.99
23.83
21.13
f1 (ppm)

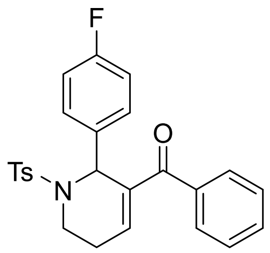
F
O
Ts
N

**3ad**

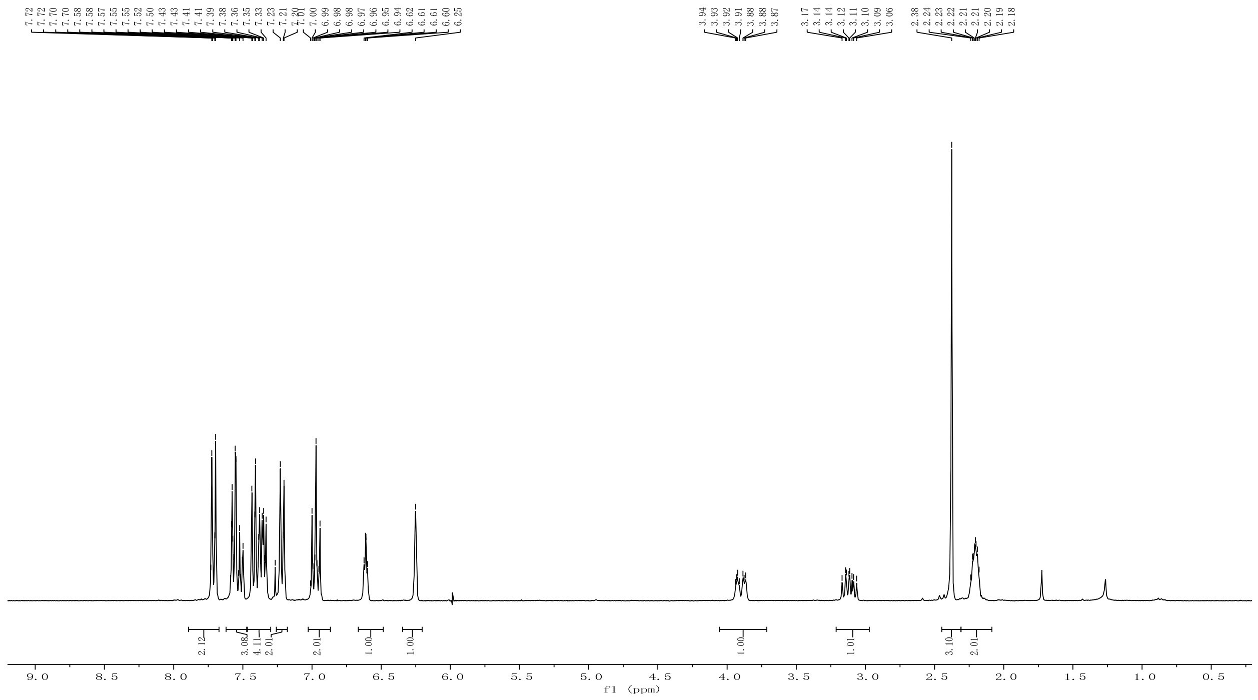
7.72 7.72 7.70 7.70 7.58 7.58 7.57 7.55 7.55 7.52 7.50 7.43 7.43 7.41 7.41 7.39 7.38 7.36 7.35 7.33 7.23 7.21 7.20 7.01 7.00 6.99 6.98 6.98 6.97 6.96 6.95 6.94 6.62 6.61 6.61 6.60 6.25
3.94 3.93 3.92 3.91 3.88 3.88 3.87
3.17 3.14 3.14 3.12 3.11 3.10 3.09 3.06
2.38 2.24 2.23 2.22 2.21 2.21 2.21 2.20 2.19 2.18
2.12 3.08 4.11 2.01 2.01 1.00 1.00 1.00 1.01 3.10 2.01
f1 (ppm)

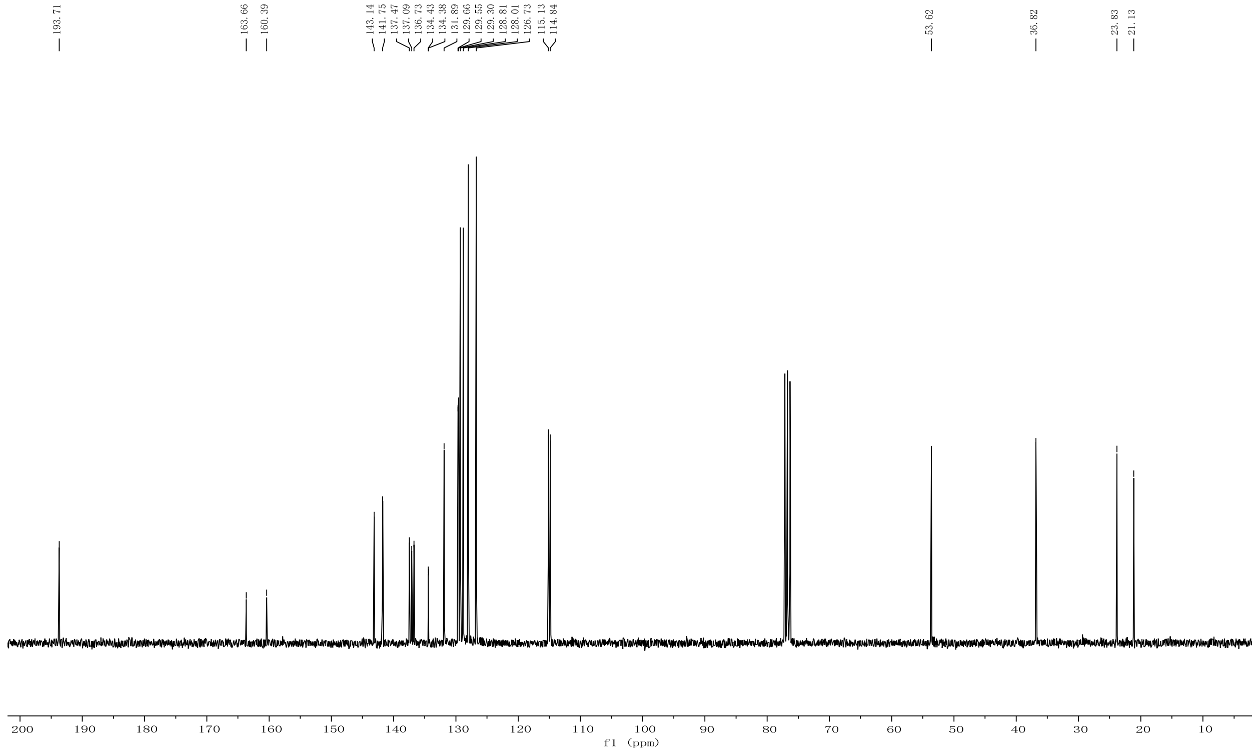
193.71
163.66 160.39
143.14 141.75 137.47 137.09 136.73 134.43 134.38 131.89 129.66 129.55 129.30 128.81 128.01 126.73 115.13 114.84
53.62
36.82
23.83 21.13
f1 (ppm)

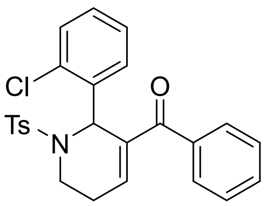
Cl
Ts
N
O

**3ae**

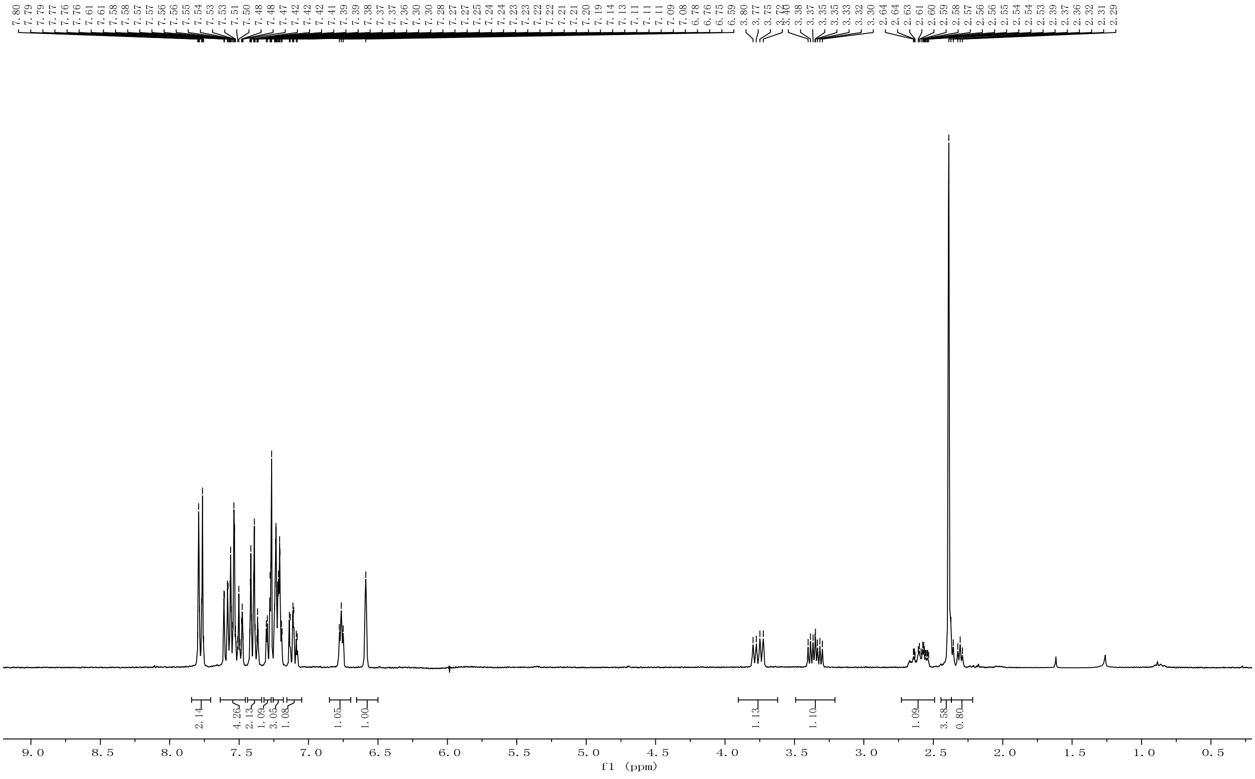
7.80 7.79 7.77 7.76 7.76 7.61 7.61 7.58 7.58 7.57 7.57 7.56 7.56 7.55 7.54 7.53 7.53 7.51 7.50 7.48 7.48 7.47 7.42 7.42 7.41 7.39 7.39 7.38 7.37 7.37 7.36 7.30 7.30 7.28 7.27 7.27 7.25 7.24 7.24 7.23 7.23 7.22 7.22 7.21 7.21 7.20 7.19 7.14 7.13 7.11 7.11 7.11 7.09 7.08 6.78 6.76 6.75 6.59 3.80 3.77 3.75 3.72 3.40 3.38 3.37 3.35 3.33 3.32 3.30 2.64 2.64 2.63 2.61 2.60 2.59 2.58 2.57 2.56 2.55 2.54 2.54 2.53 2.39 2.37 2.36 2.32 2.31 2.29
2.14 4.26 2.13 1.09 3.05 1.08 1.05 1.00 1.13 1.10 1.09 3.58 0.80
9.0 8.5 8.0 7.5 7.0 6.5 6.0 5.5 5.0 4.5 4.0 3.5 3.0 2.5 2.0 1.5 1.0 0.5
f1 (ppm)

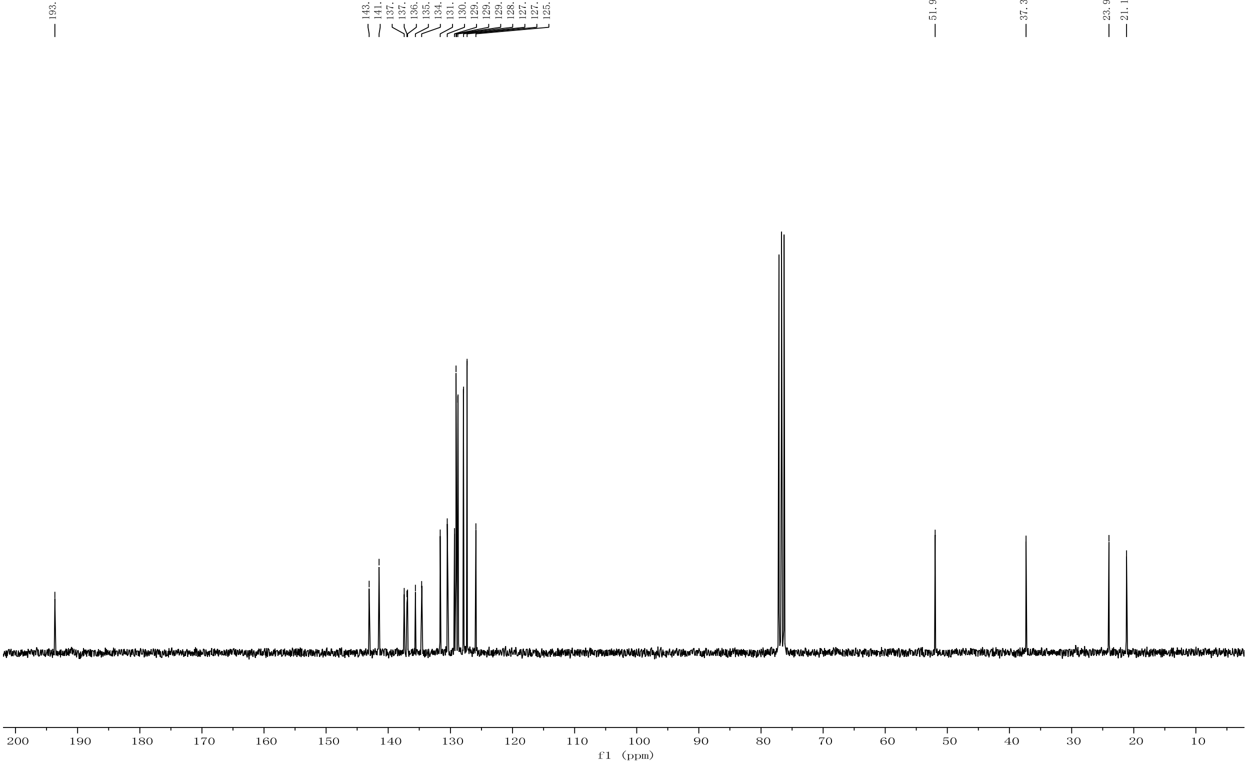
193.66
143.07 141.48 137.44 137.00 136.88 135.63 134.61 131.64 130.49 129.35 129.08 129.02 128.79 127.89 127.32 125.90
51.97
37.33
23.99 21.15
200 190 180 170 160 150 140 130 120 110 100 90 80 70 60 50 40 30 20 10
f1 (ppm)

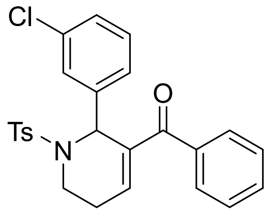
Cl
Ts
N
O

**3af**

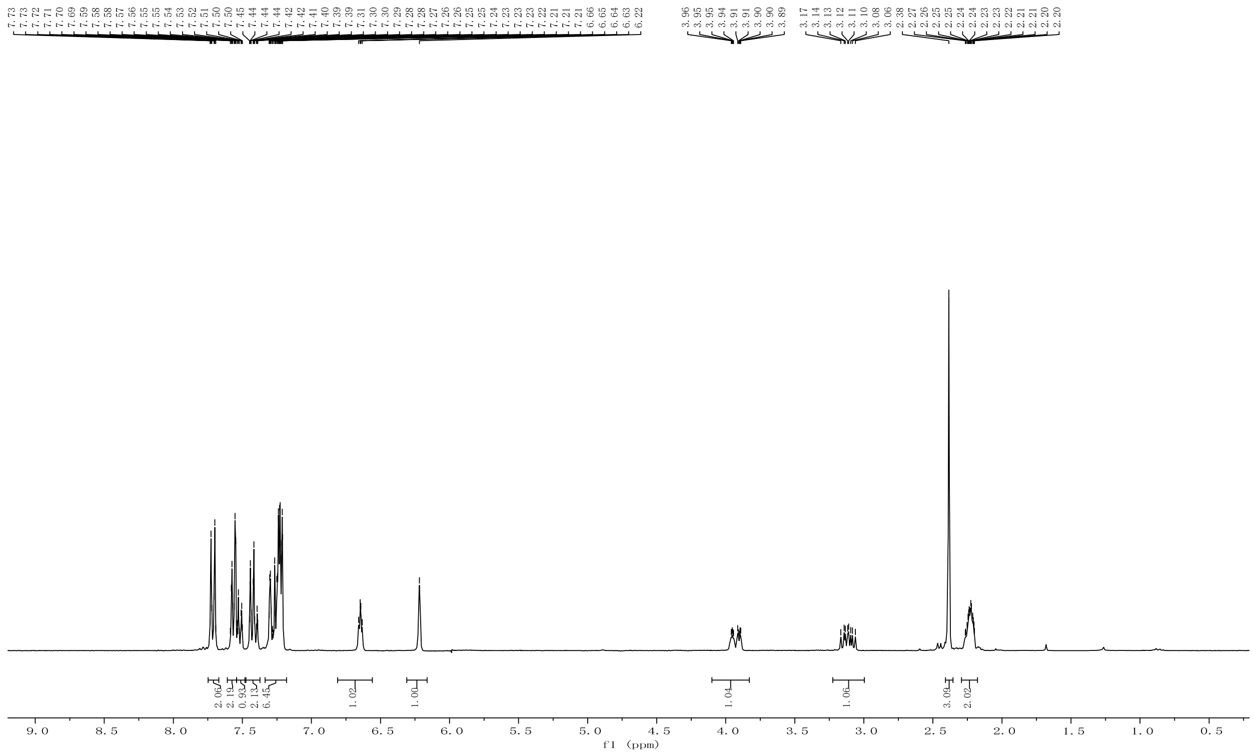
7.73 7.73 7.72 7.71 7.70 7.69 7.59 7.58 7.57 7.56 7.55 7.55 7.54 7.53 7.52 7.51 7.50 7.50 7.45 7.44 7.44 7.44 7.42 7.41 7.40 7.39 7.39 7.31 7.30 7.30 7.29 7.28 7.28 7.27 7.26 7.26 7.25 7.25 7.24 7.23 7.23 7.23 7.22 7.22 7.21 7.21 7.21 6.66 6.65 6.64 6.63 6.22
3.96 3.95 3.95 3.94 3.91 3.91 3.90 3.90 3.89
3.17 3.14 3.13 3.12 3.11 3.10 3.08 3.06
2.38 2.27 2.26 2.25 2.25 2.24 2.24 2.23 2.23 2.22 2.21 2.21 2.20 2.20
2.06 2.19 0.93 2.13 6.45 1.02 1.00 1.04 1.06 3.09 2.02
f1 (ppm)

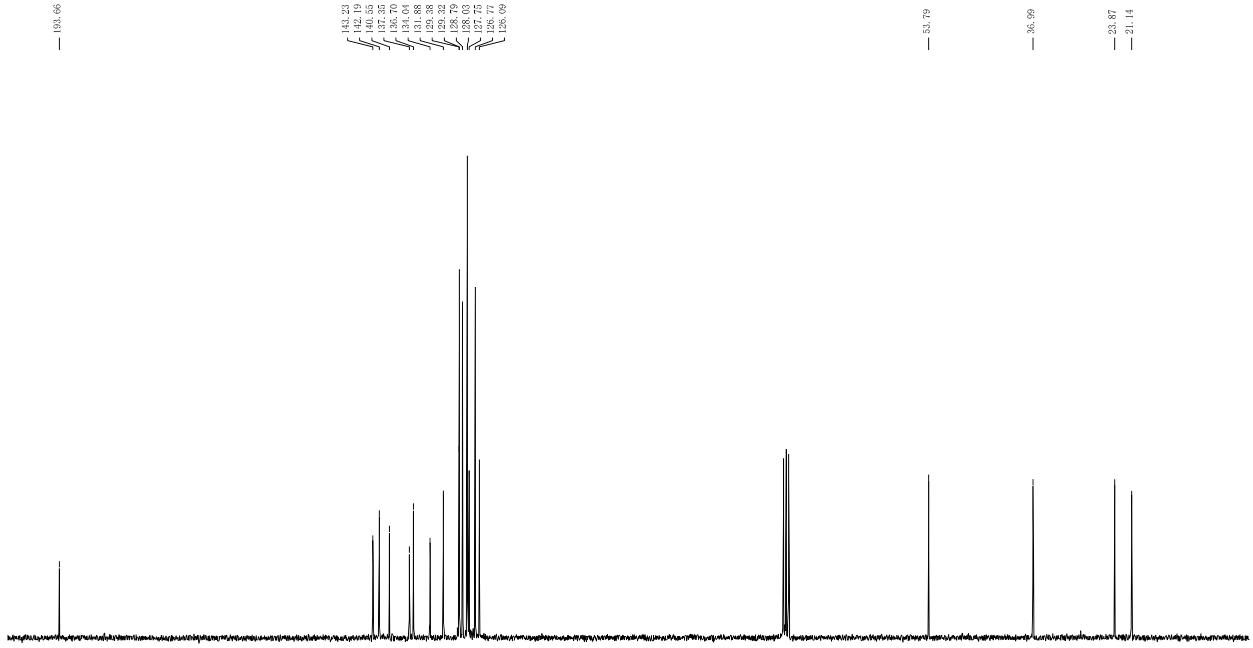
193.66
143.23 142.19 140.55 137.35 136.70 134.04 131.88 129.38 129.32 128.79 128.03 127.75 126.77 126.09
53.79
36.99
23.87 21.14
f1 (ppm)

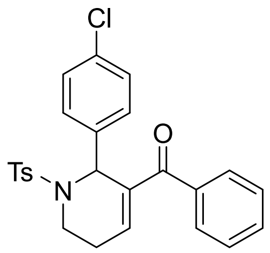

**3ag**

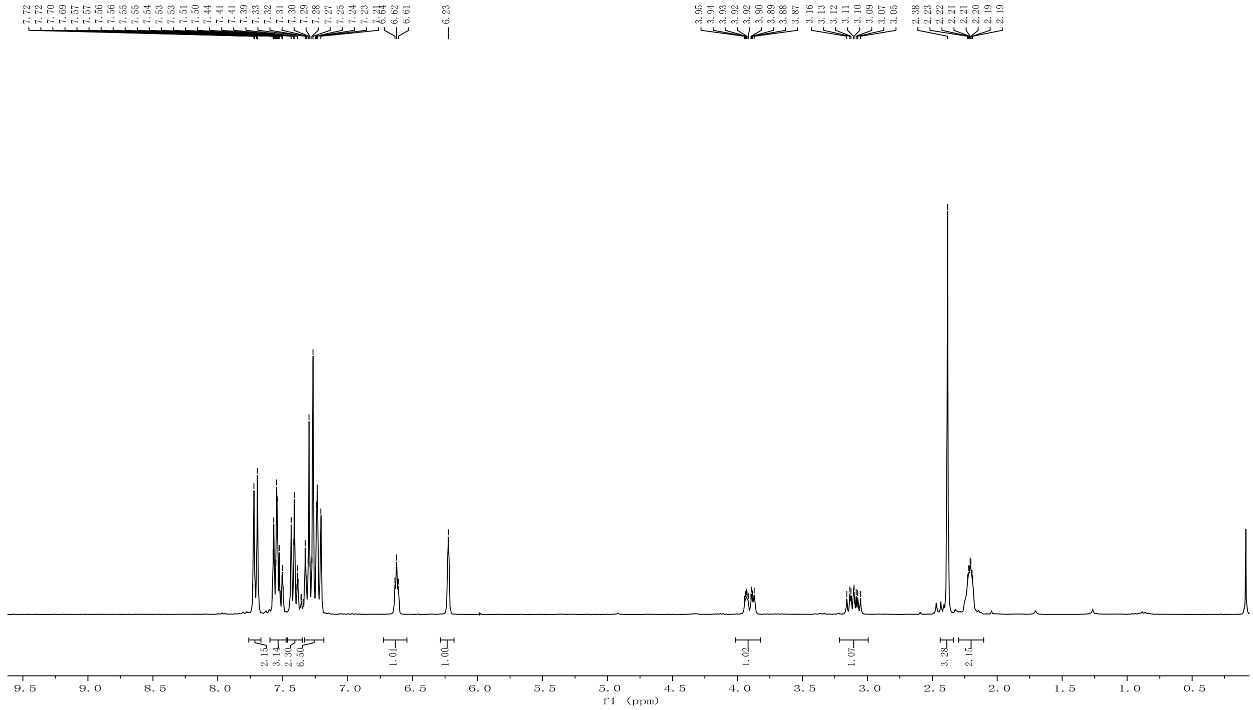

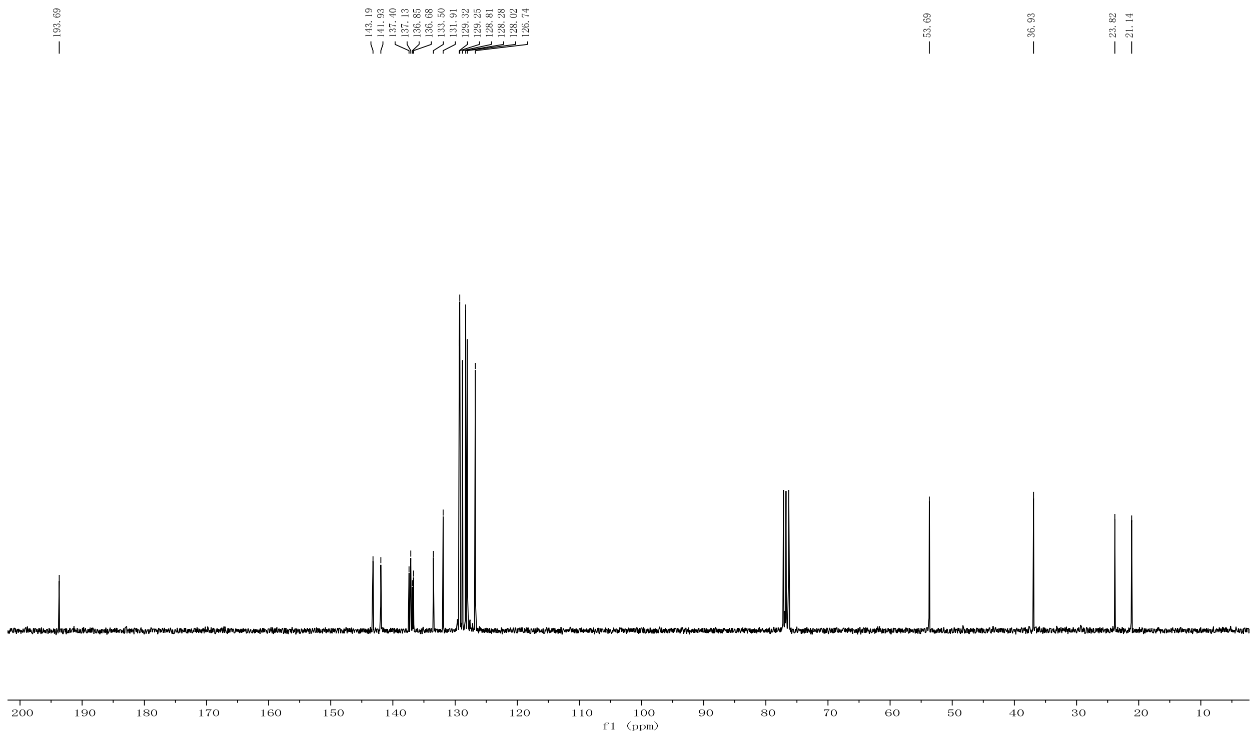

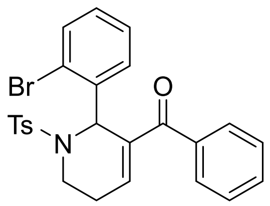

Br
Ts
N
O


**3ah**

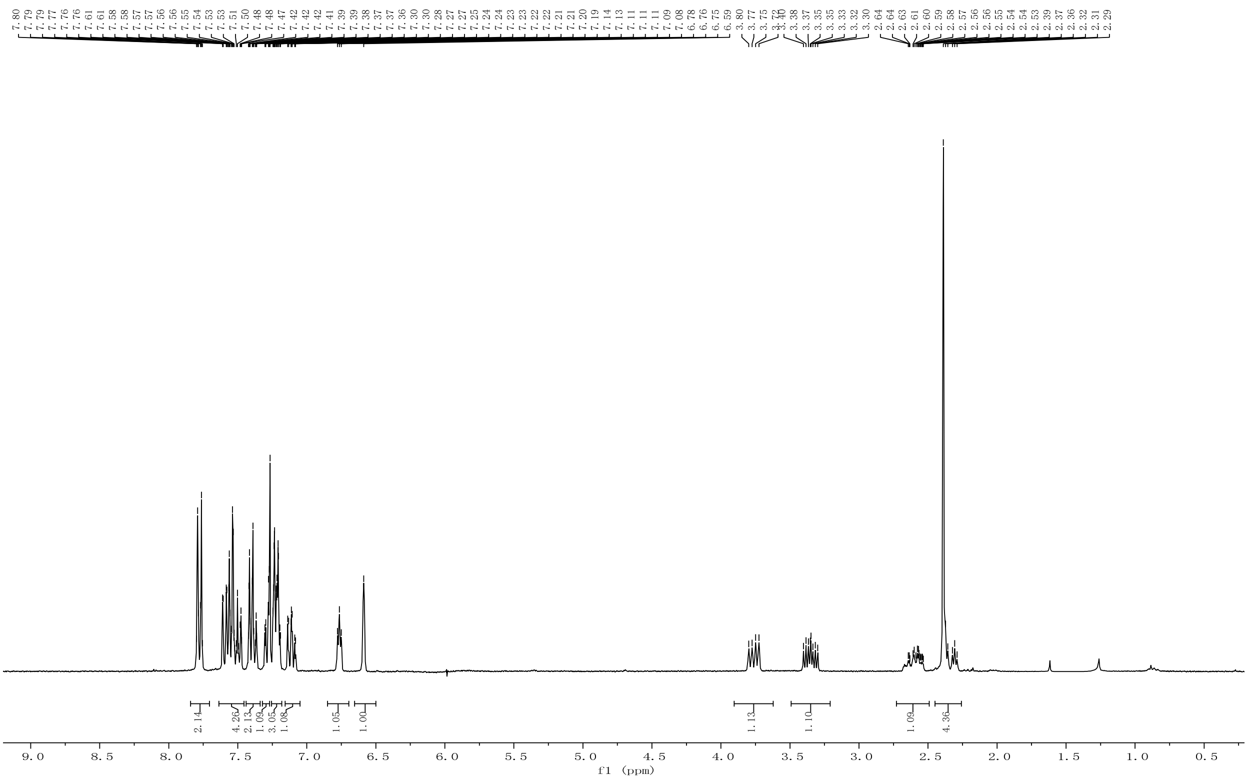

7.80 7.79 7.79 7.77 7.76 7.76 7.61 7.61 7.58 7.58 7.57 7.57 7.56 7.56 7.55 7.54 7.53 7.53 7.51 7.50 7.48 7.48 7.47 7.42 7.42 7.41 7.39 7.39 7.38 7.37 7.37 7.36 7.30 7.30 7.28 7.27 7.27 7.25 7.24 7.24 7.23 7.23 7.22 7.22 7.21 7.21 7.20 7.19 7.14 7.13 7.11 7.11 7.11 7.09 7.08 6.78 6.76 6.75 6.59 3.80 3.77 3.75 3.72 3.40 3.38 3.37 3.35 3.35 3.33 3.32 3.30 2.64 2.64 2.63 2.61 2.60 2.59 2.58 2.57 2.56 2.56 2.55 2.54 2.54 2.53 2.39 2.37 2.36 2.32 2.31 2.29
2.14 4.26 2.13 1.09 3.05 1.08 1.05 1.00 1.13 1.10 1.09 4.36
9.0 8.5 8.0 7.5 7.0 6.5 6.0 5.5 5.0 4.5 4.0 3.5 3.0 2.5 2.0 1.5 1.0 0.5
f1 (ppm)


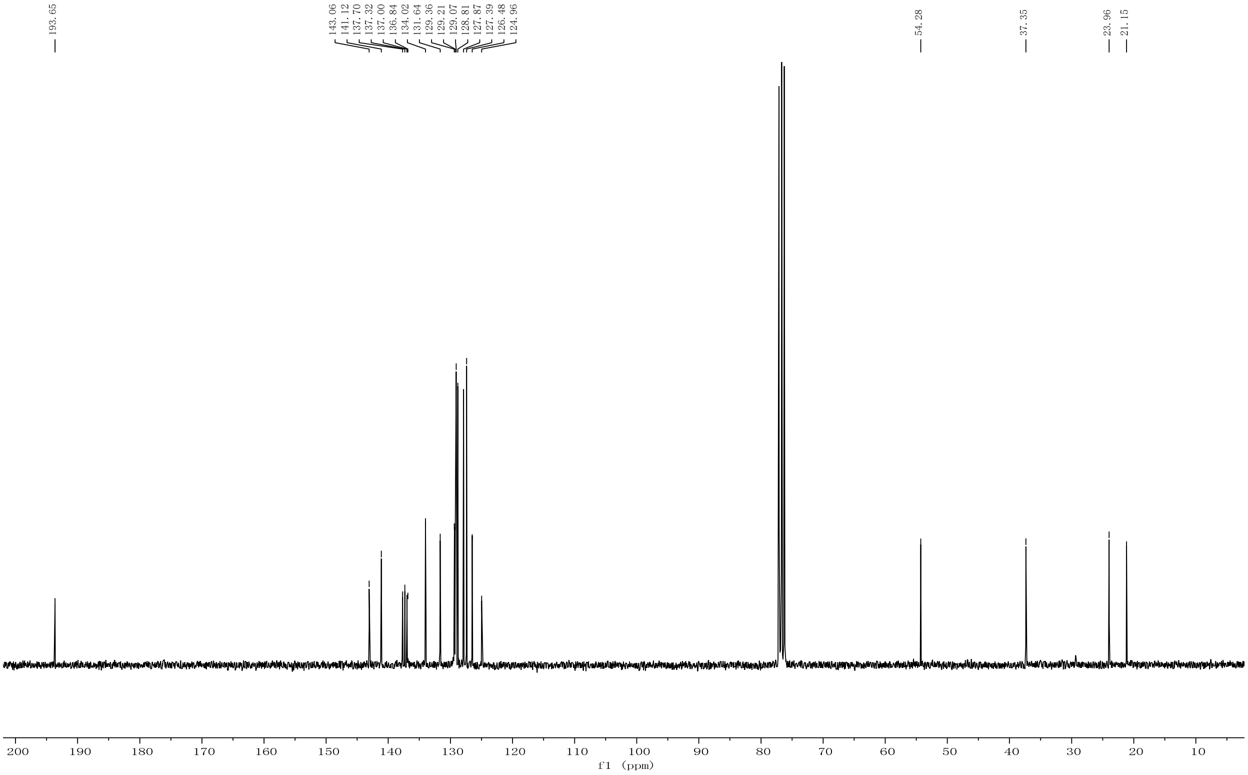

193.65
143.06 141.12 137.70 137.32 137.00 136.84 134.02 131.64 129.36 129.21 129.07 128.81 127.87 127.39 126.48 124.96
54.28
37.35
23.96 21.15
200 190 180 170 160 150 140 130 120 110 100 90 80 70 60 50 40 30 20 10
f1 (ppm)

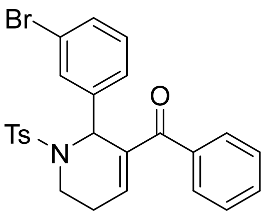
Br
O
Ts
N

**3ai**

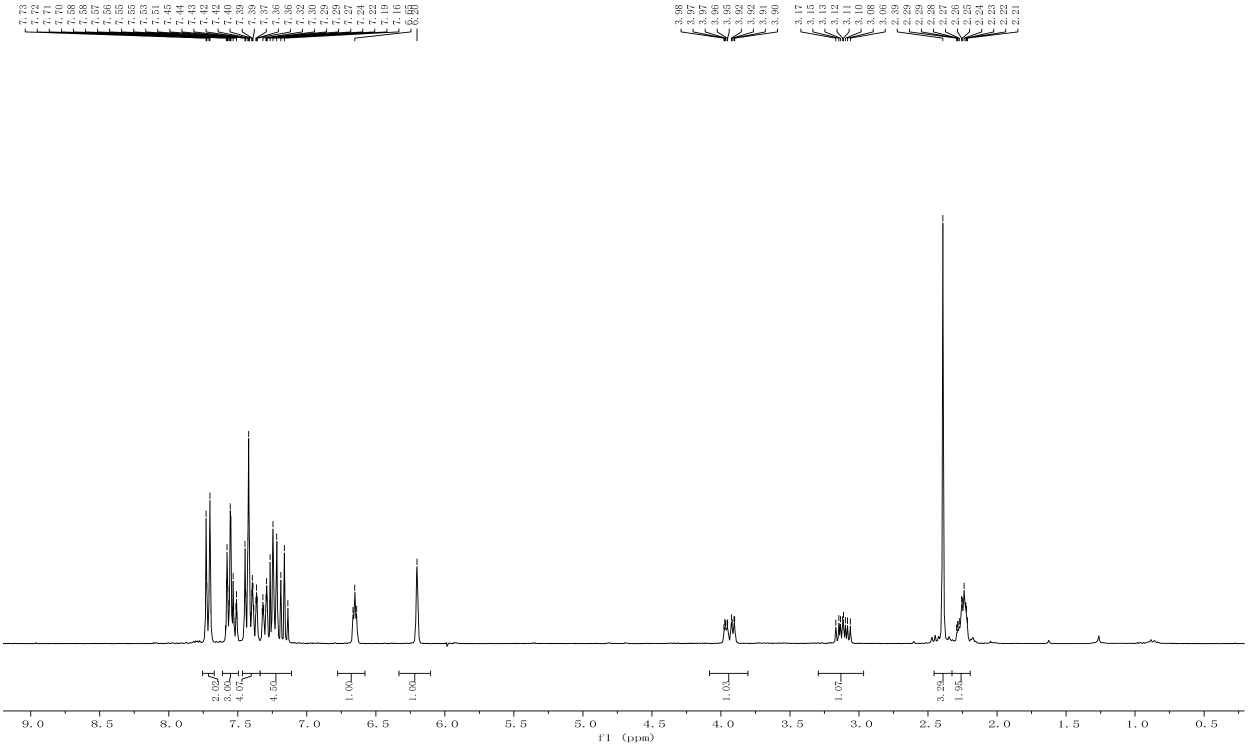
7.73 7.72 7.71 7.70 7.58 7.58 7.57 7.56 7.55 7.55 7.53 7.51 7.45 7.44 7.43 7.42 7.42 7.40 7.39 7.39 7.37 7.36 7.36 7.32 7.30 7.29 7.29 7.27 7.24 7.22 7.19 7.16 6.65 6.20
3.98 3.97 3.97 3.96 3.95 3.92 3.92 3.91 3.90
3.17 3.15 3.13 3.12 3.11 3.10 3.08 3.06
2.39 2.29 2.29 2.28 2.27 2.26 2.25 2.24 2.23 2.22 2.21
2.02 3.00 4.07 4.50 1.00 1.00 1.03 1.07 3.29 1.95
9.0 8.5 8.0 7.5 7.0 6.5 6.0 5.5 5.0 4.5 4.0 3.5 3.0 2.5 2.0 1.5 1.0 0.5
f1 (ppm)

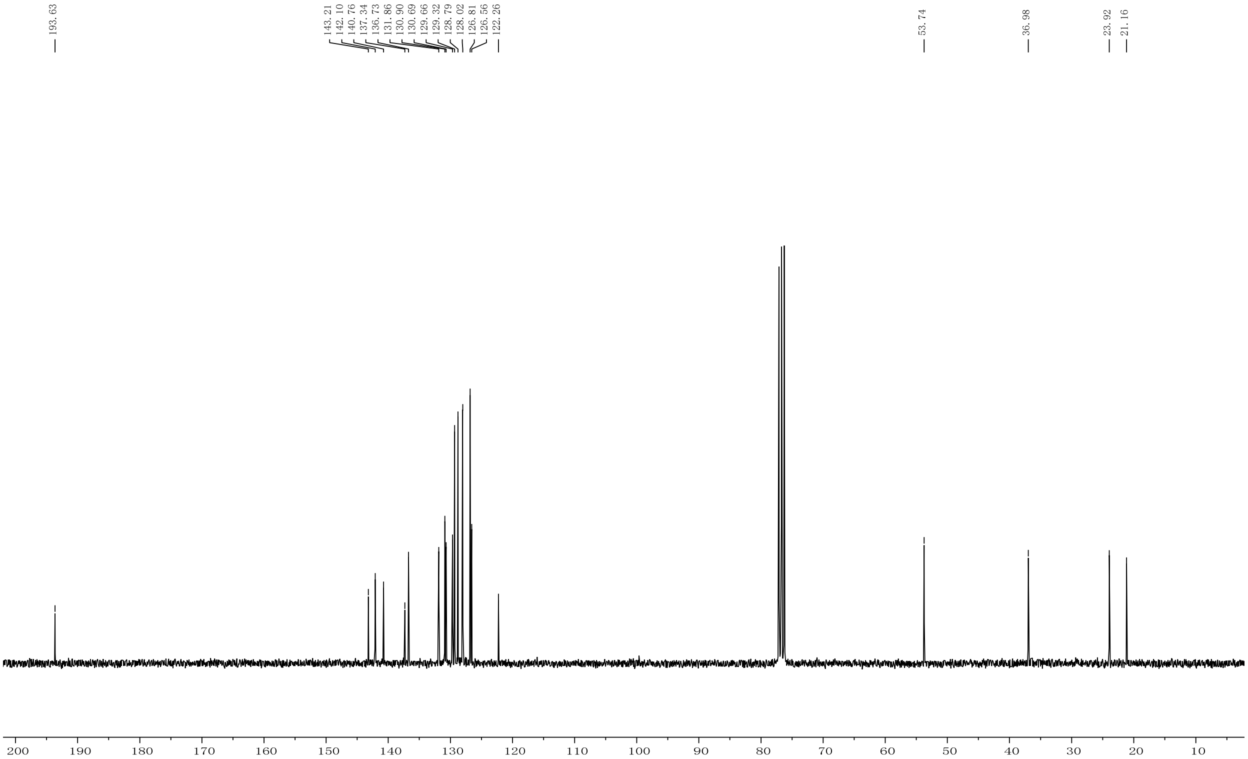
193.63
143.21 142.10 140.76 137.34 136.73 131.86 130.90 130.69 129.66 129.32 128.79 128.02 126.81 126.56 122.26
53.74
36.98
23.92 21.16
200 190 180 170 160 150 140 130 120 110 100 90 80 70 60 50 40 30 20 10
f1 (ppm)

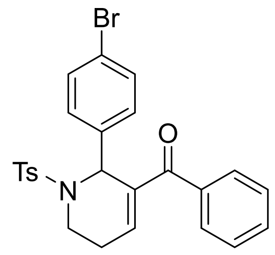
Br
O
Ts
N

**3aj**

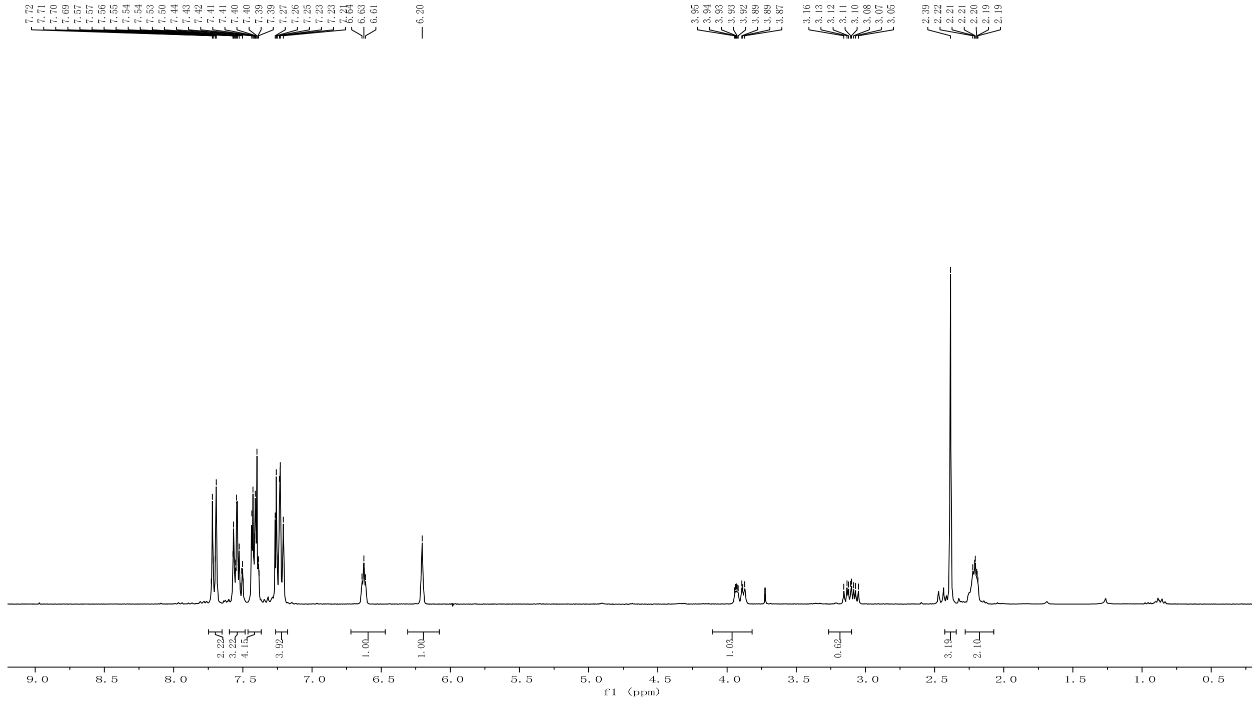
7.72 7.71 7.70 7.69 7.57 7.57 7.56 7.55 7.54 7.54 7.53 7.50 7.44 7.43 7.42 7.41 7.41 7.40 7.39 7.39 7.27 7.26 7.25 7.23 7.23 7.21 6.64 6.63 6.61
6.20
3.95 3.94 3.93 3.93 3.92 3.89 3.89 3.87
3.16 3.13 3.12 3.11 3.10 3.08 3.07 3.05
2.39 2.22 2.21 2.21 2.20 2.19 2.19
2.22 3.22 4.15 3.92 1.00 1.00 1.03 0.62 3.19 2.10
f1 (ppm)

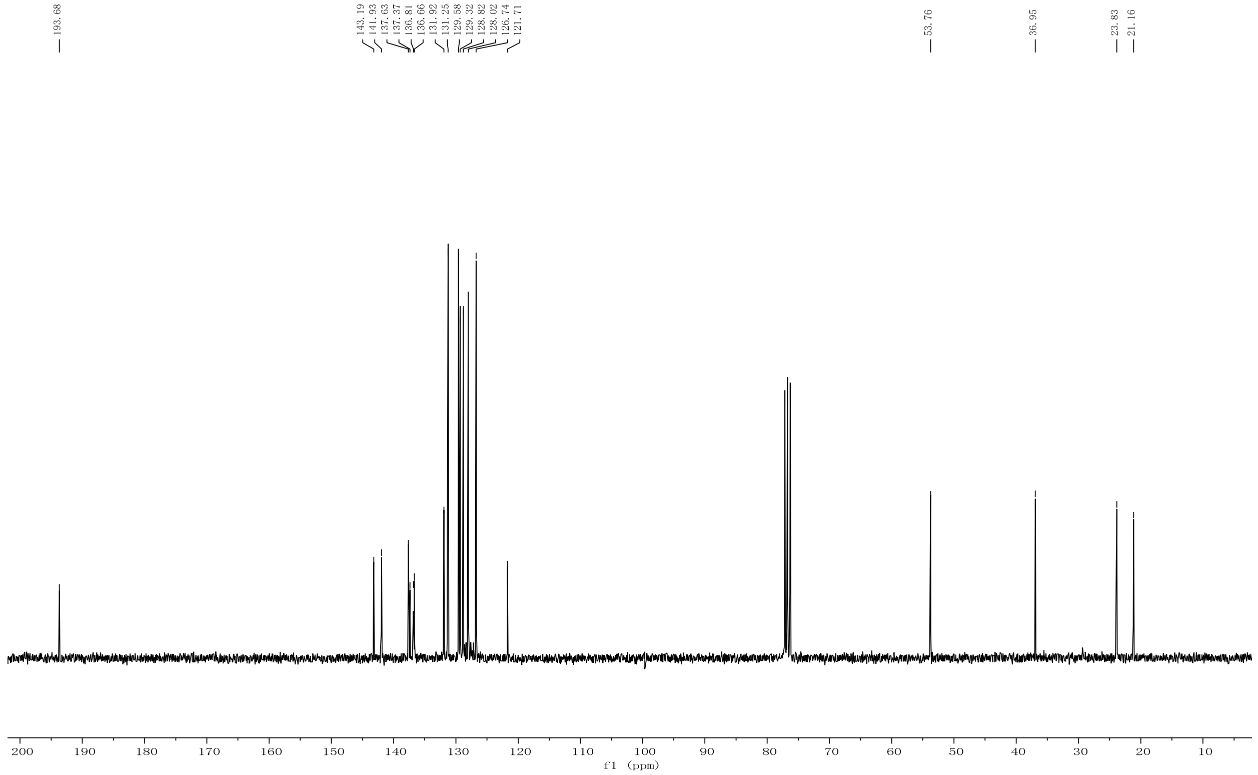
193.68
143.19 141.93 137.63 137.37 136.81 136.66 131.92 131.25 129.58 129.32 128.82 128.02 126.74 121.71
53.76
36.95
23.83 21.16
f1 (ppm)

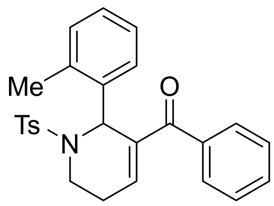
Me
O
Ts
N

**3ak**

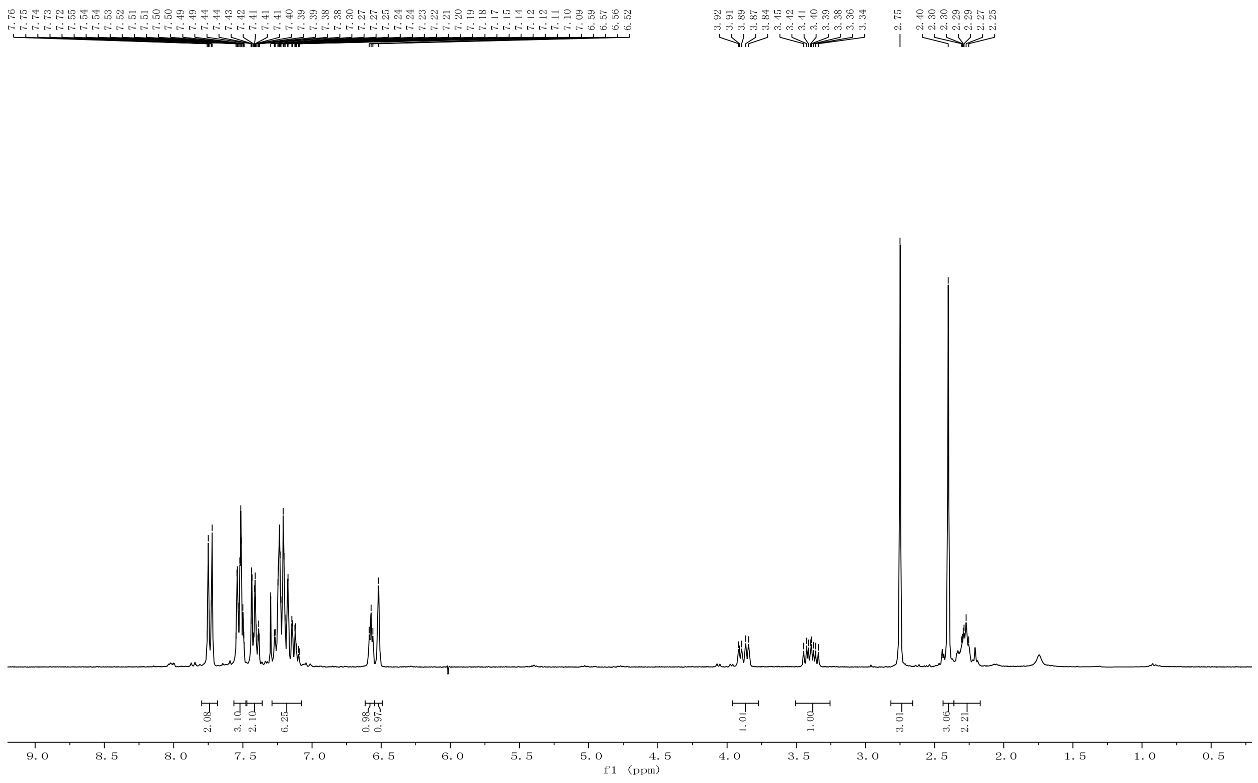
7.76 7.75 7.74 7.73 7.72 7.55 7.54 7.54 7.53 7.52 7.51 7.51 7.50 7.50 7.49 7.49 7.44 7.43 7.42 7.41 7.41 7.41 7.40 7.39 7.39 7.38 7.38 7.38 7.30 7.27 7.27 7.25 7.24 7.24 7.23 7.22 7.21 7.20 7.19 7.18 7.17 7.15 7.14 7.12 7.12 7.11 7.10 7.09 6.59 6.57 6.56 6.52
3.92 3.91 3.89 3.87 3.84 3.45 3.42 3.41 3.40 3.39 3.38 3.36 3.34
2.75
2.40 2.30 2.30 2.29 2.29 2.27 2.25
2.08
3.10
2.10
6.25
0.98
0.97
1.01
1.00
3.01
3.06
2.21
f1 (ppm)

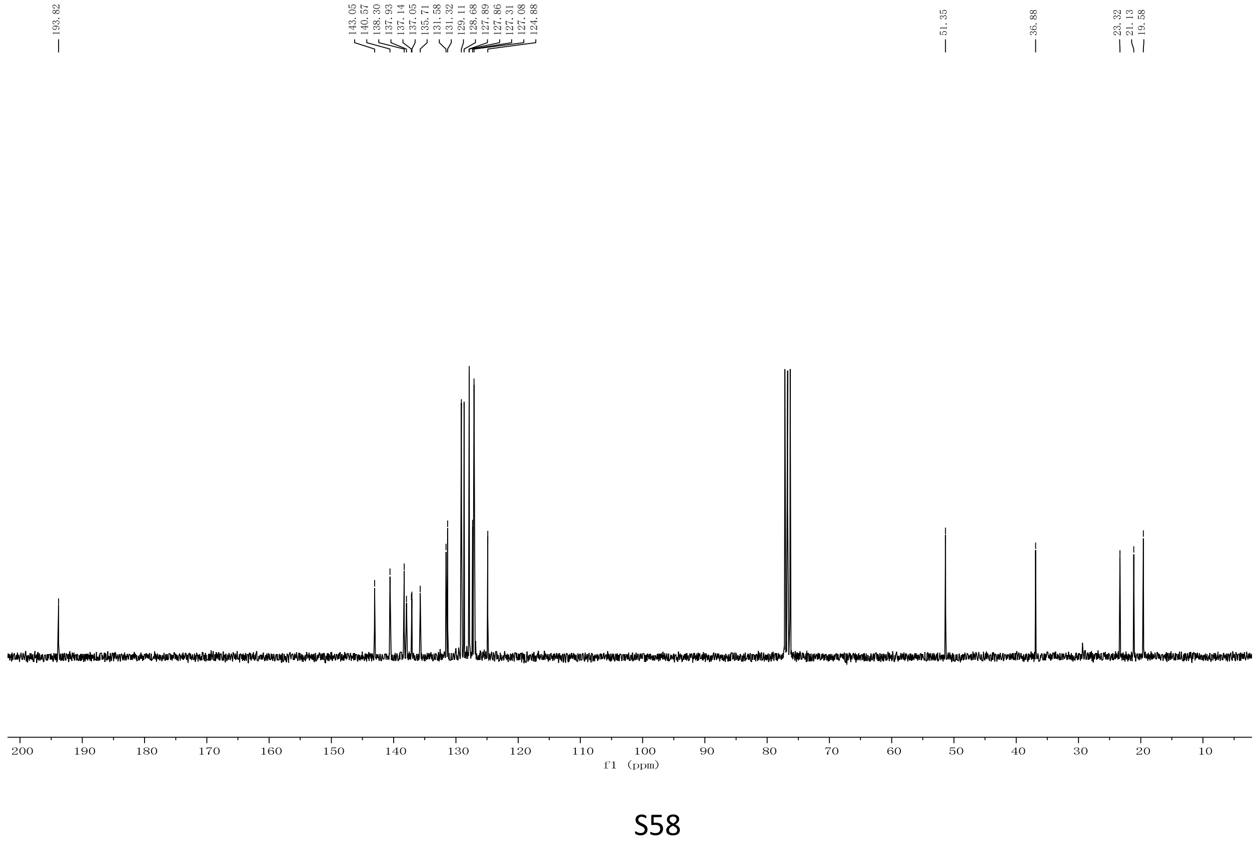
193.82
143.05 140.57 138.30 137.93 137.14 137.05 135.71 131.58 131.32 129.11 128.68 127.89 127.86 127.31 127.08 124.88
51.35
36.88
23.32 21.13 19.58
f1 (ppm)

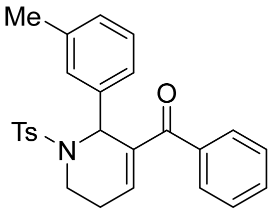
Me
O
Ts
N

**3al**

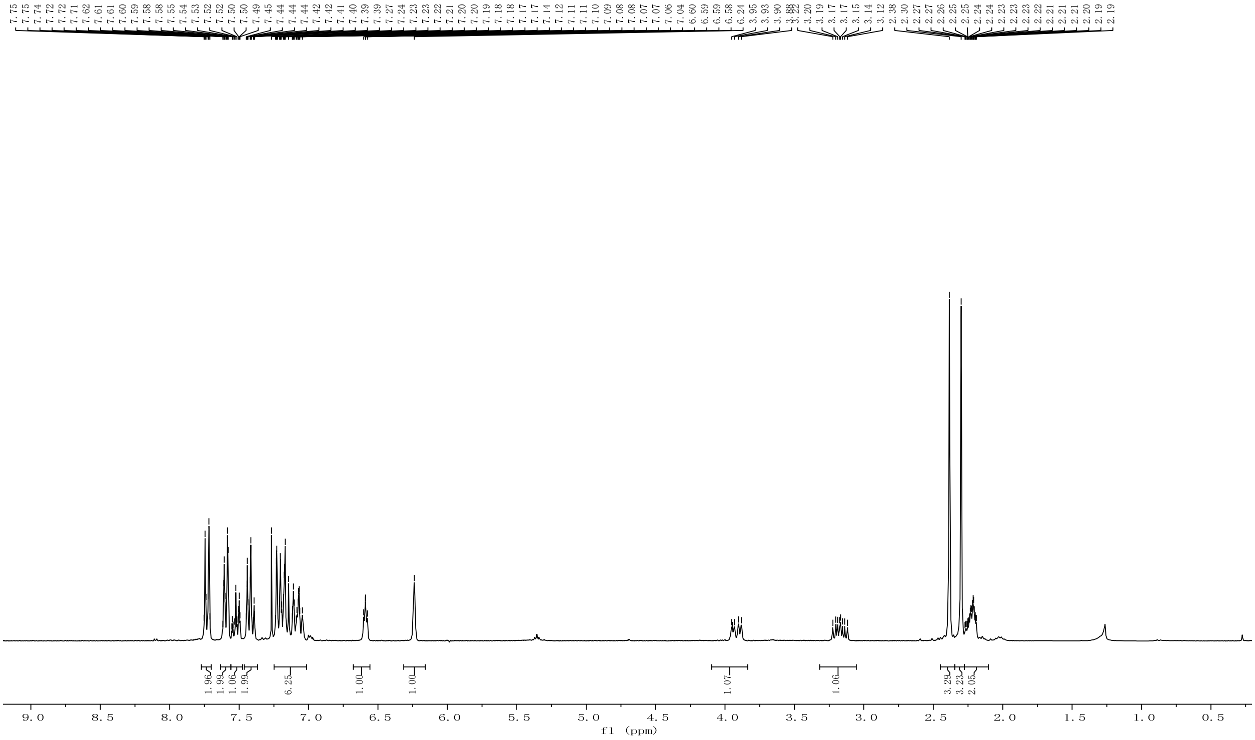
7.75 7.75 7.74 7.72 7.72 7.71 7.62 7.61 7.60 7.59 7.58 7.58 7.55 7.54 7.53 7.52 7.52 7.50 7.50 7.49 7.45 7.44 7.44 7.44 7.42 7.42 7.41 7.40 7.39 7.27 7.24 7.23 7.23 7.22 7.21 7.20 7.20 7.19 7.18 7.18 7.17 7.17 7.14 7.12 7.11 7.11 7.10 7.09 7.08 7.08 7.07 7.07 7.06 7.04 6.60 6.59 6.59 6.58 6.24 3.95 3.93 3.90
3.20 3.19 3.17 3.17 3.15 3.14 3.12 2.38 2.30 2.27 2.27 2.26 2.25 2.25 2.24 2.24 2.23 2.23 2.23 2.23 2.22 2.21 2.21 2.21 2.20 2.19 2.19
1.96 1.99 1.06 1.99 6.25 1.00 1.00 1.07 1.06 3.29 3.23 2.05
9.0 8.5 8.0 7.5 7.0 6.5 6.0 5.5 5.0 4.5 4.0 3.5 3.0 2.5 2.0 1.5 1.0 0.5
f1 (ppm)

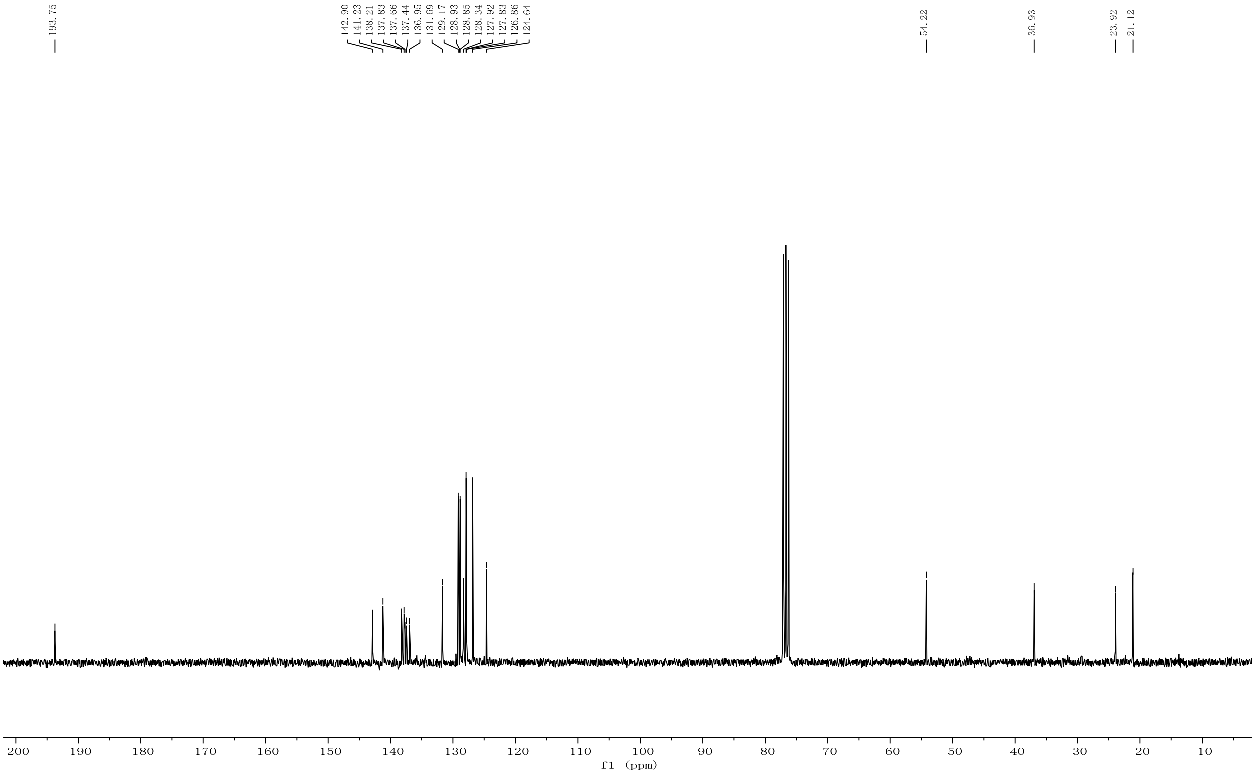
193.75
142.90 141.23 138.21 137.83 137.66 137.44 136.95 131.69 129.17 128.93 128.85 128.34 127.92 127.83 126.86 124.64
54.22
36.93
23.92
21.12
200 190 180 170 160 150 140 130 120 110 100 90 80 70 60 50 40 30 20 10
f1 (ppm)

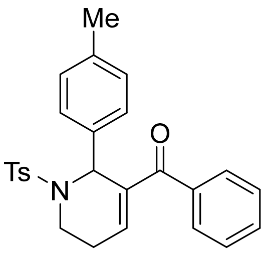

Me
O
Ts
N


**3am**

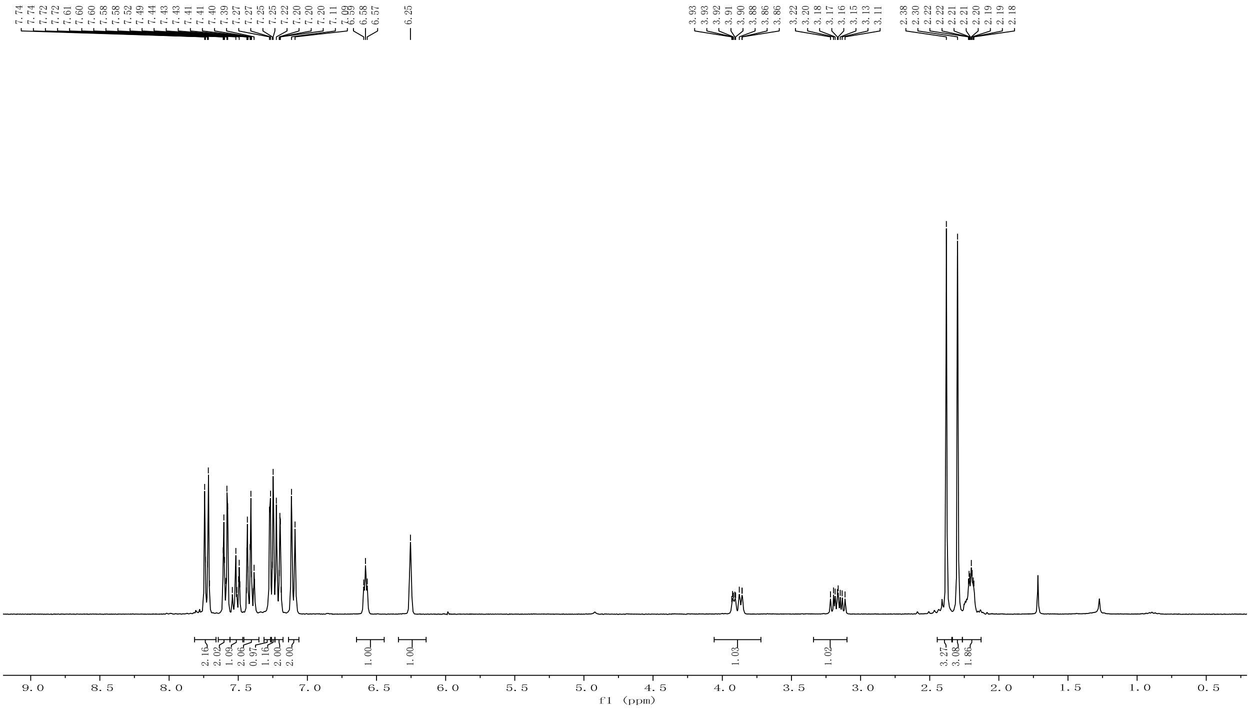

7.74 7.74 7.72 7.72 7.61 7.60 7.58 7.58 7.52 7.49 7.44 7.43 7.43 7.41 7.40 7.39 7.27 7.25 7.25 7.22 7.20 7.20 7.11 7.09 6.59 6.58 6.57 6.25
3.93 3.93 3.92 3.91 3.90 3.88 3.86 3.86 3.22 3.20 3.18 3.17 3.16 3.15 3.13 3.11 2.38 2.30 2.22 2.22 2.21 2.21 2.20 2.19 2.18
2.16 2.02 1.09 2.06 0.97 1.16 2.00 2.00 1.00 1.00 1.03 1.02 3.27 3.08 1.86
9.0 8.5 8.0 7.5 7.0 6.5 6.0 5.5 5.0 4.5 4.0 3.5 3.0 2.5 2.0 1.5 1.0 0.5
f1 (ppm)


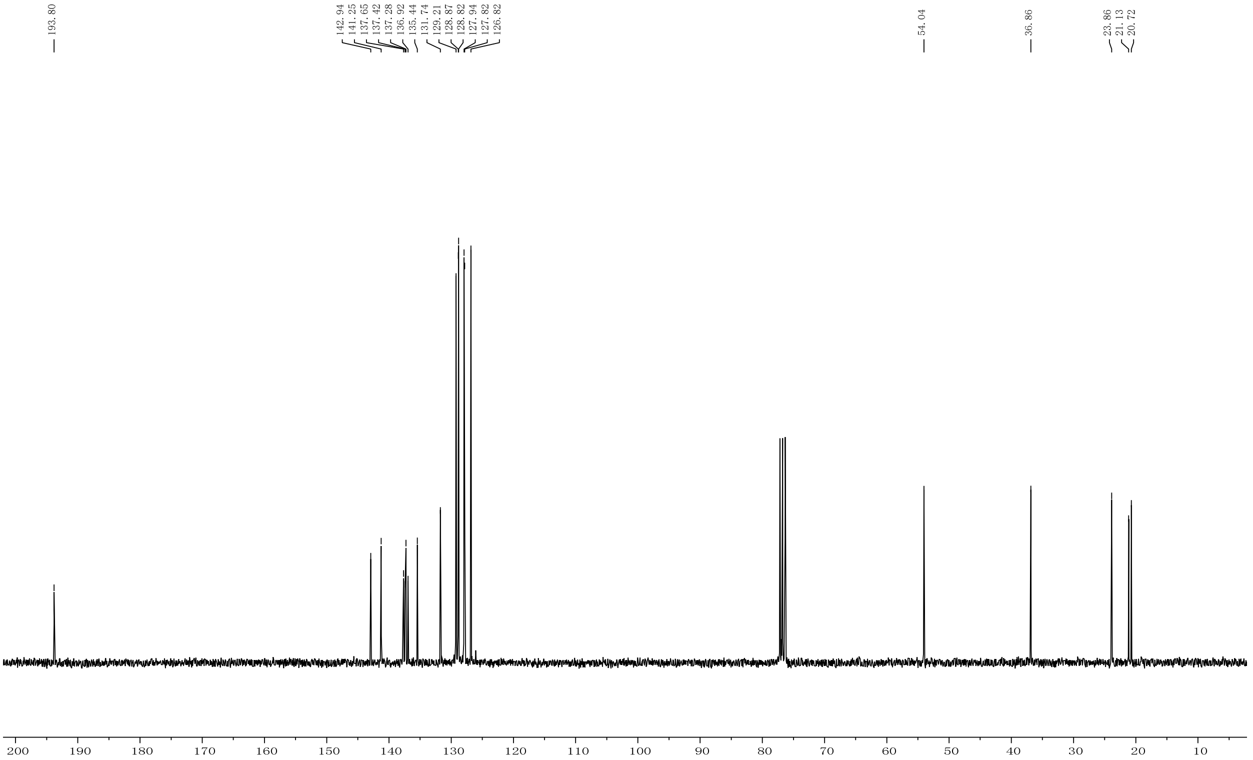

193.80
142.94 141.25 137.65 137.42 137.28 136.92 135.44 131.74 129.21 128.87 128.82 127.94 127.82 126.82
54.04
36.86
23.86 21.13 20.72
200 190 180 170 160 150 140 130 120 110 100 90 80 70 60 50 40 30 20 10
f1 (ppm)

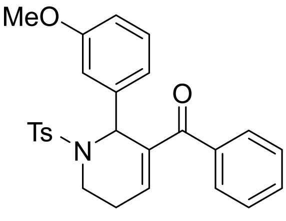
MeO
O
Ts
N

**3an**

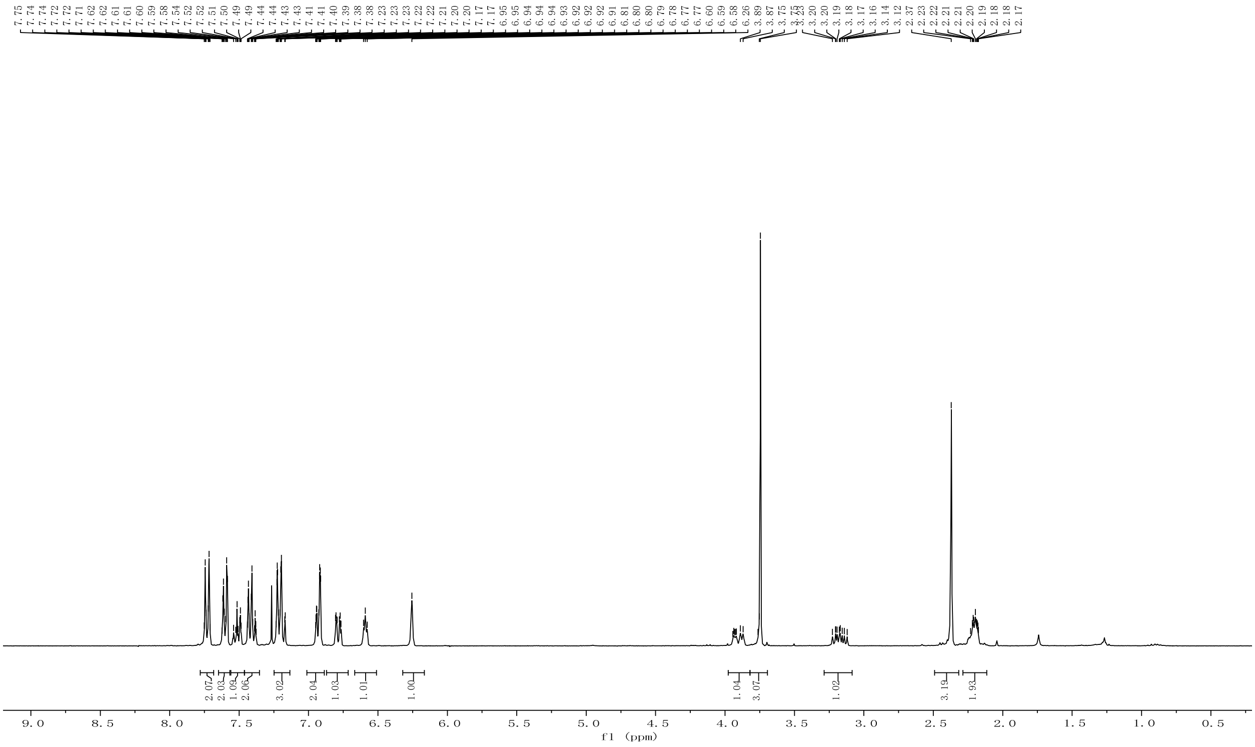
7.75 7.74 7.74 7.72 7.72 7.71 7.62 7.61 7.61 7.60 7.59 7.58 7.54 7.52 7.52 7.51 7.50 7.49 7.49 7.44 7.44 7.43 7.43 7.41 7.41 7.40 7.39 7.38 7.38 7.23 7.23 7.23 7.22 7.22 7.21 7.20 7.20 7.17 7.17 6.95 6.95 6.94 6.94 6.94 6.93 6.92 6.92 6.92 6.91 6.81 6.80 6.80 6.79 6.78 6.77 6.77 6.60 6.59 6.58 6.26 3.89 3.87 3.75 3.23 3.20 3.20 3.19 3.18 3.17 3.16 3.14 3.12 2.37 2.23 2.22 2.21 2.21 2.20 2.19 2.18 2.18 2.17
2.07 2.03 1.09 2.06 3.02 2.04 1.03 1.01 1.00 1.04 3.07 1.02 3.19 1.93
9.0 8.5 8.0 7.5 7.0 6.5 6.0 5.5 5.0 4.5 4.0 3.5 3.0 2.5 2.0 1.5 1.0 0.5
f1 (ppm)

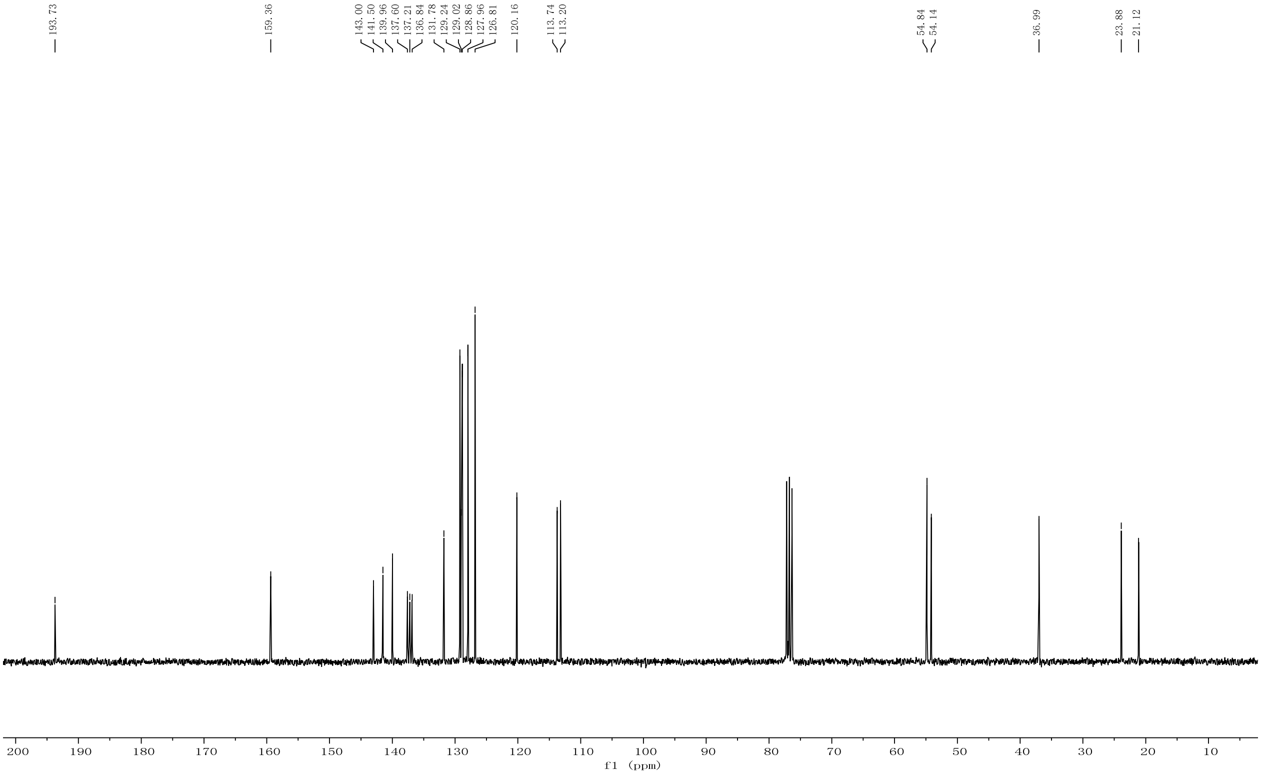
193.73
159.36
143.00 141.50 139.96 137.60 137.21 136.84 131.78 129.24 129.02 128.86 127.96 126.81
120.16
113.74 113.20
54.84 54.14
36.99
23.88 21.12
200 190 180 170 160 150 140 130 120 110 100 90 80 70 60 50 40 30 20 10
f1 (ppm)

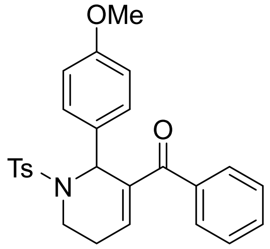

OMe
O
Ts
N


**3ao**

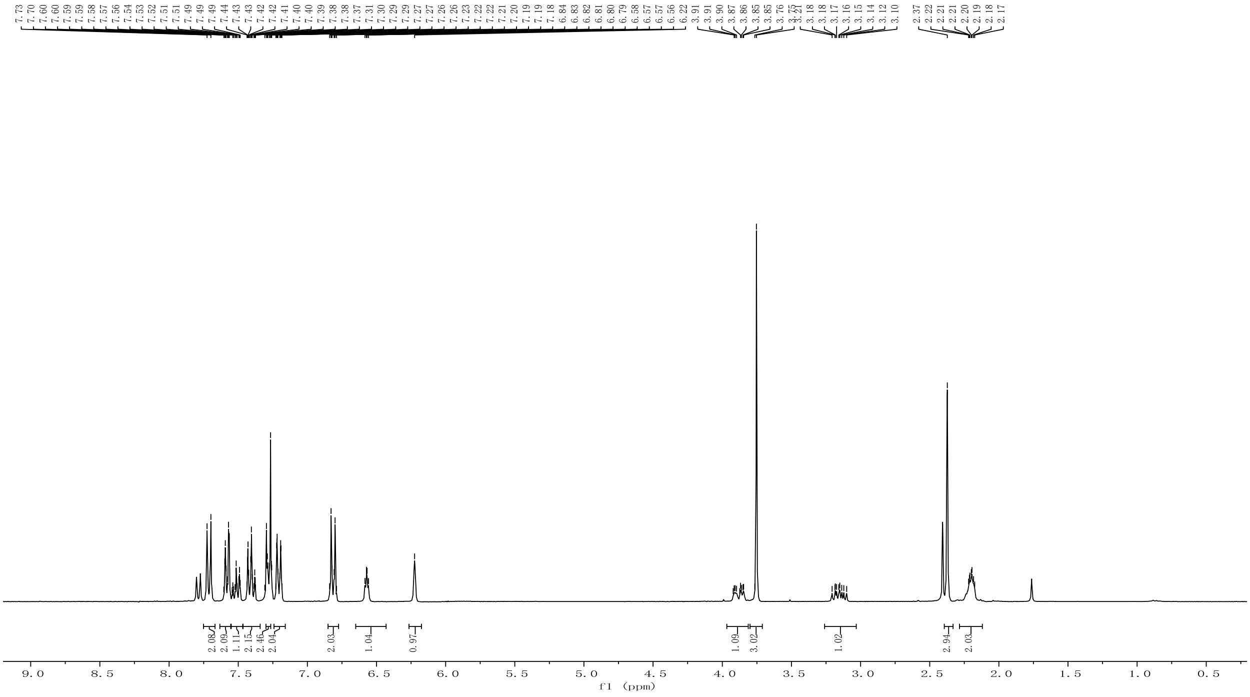

7.73 7.70 7.60 7.60 7.59 7.59 7.58 7.57 7.56 7.54 7.53 7.52 7.51 7.51 7.49 7.49 7.44 7.43 7.43 7.42 7.42 7.41 7.40 7.39 7.38 7.38 7.37 7.31 7.30 7.29 7.29 7.27 7.27 7.26 7.23 7.22 7.22 7.21 7.20 7.19 7.19 7.18 6.84 6.83 6.82 6.81 6.80 6.79 6.58 6.57 6.57 6.56 6.22 3.91 3.91 3.90 3.87 3.86 3.85 3.85 3.76 3.18 3.18 3.17 3.16 3.15 3.14 3.12 3.10 2.37 2.22 2.21 2.21 2.20 2.19 2.18 2.17
2.08 2.09 1.11 2.15 2.46 2.04 2.03 1.04 0.97 1.09 3.02 1.02 2.94 2.03
9.0 8.5 8.0 7.5 7.0 6.5 6.0 5.5 5.0 4.5 4.0 3.5 3.0 2.5 2.0 1.5 1.0 0.5
f1 (ppm)


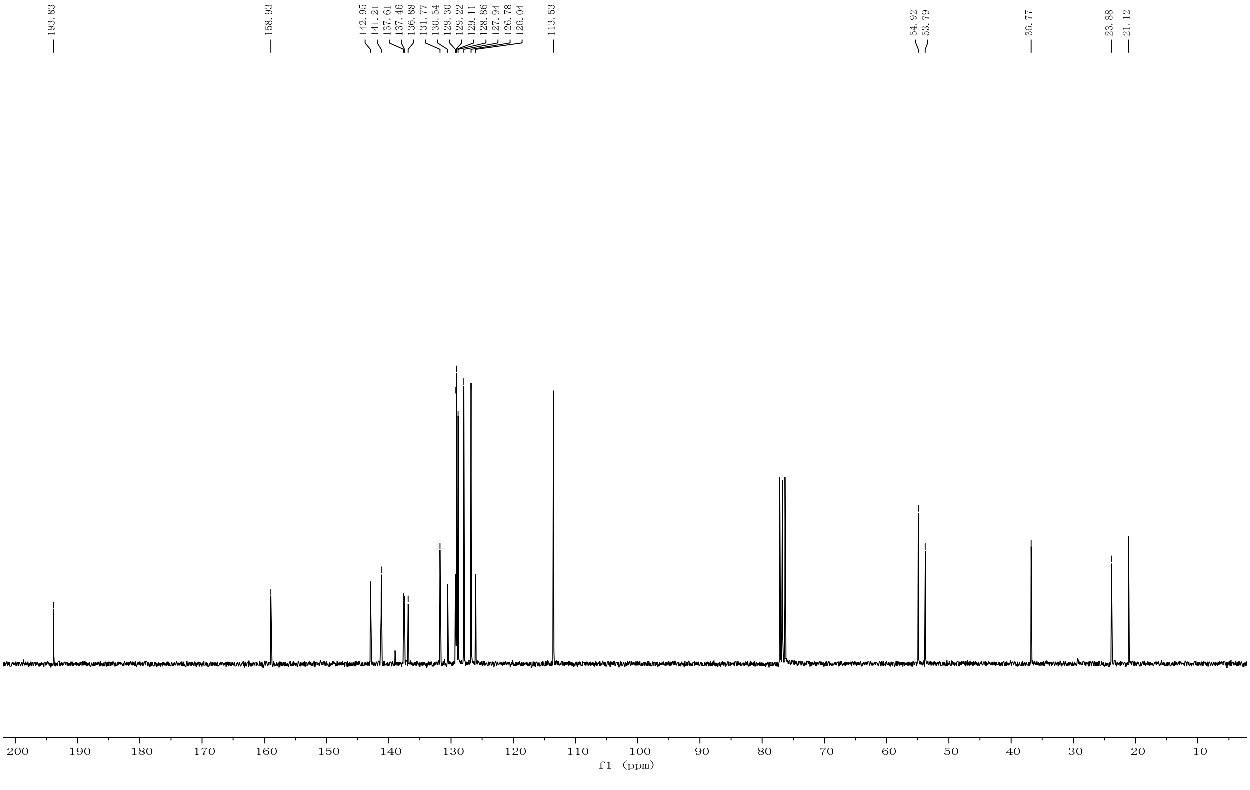

193.83
158.93
142.95 141.21 137.61 137.46 136.88 131.77 130.54 129.30 129.22 129.11 128.86 127.94 126.78 126.04
113.53
54.92 53.79
36.77
23.88 21.12
200 190 180 170 160 150 140 130 120 110 100 90 80 70 60 50 40 30 20 10
f1 (ppm)

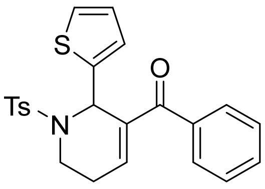

S
O
Ts
N


**3ap**

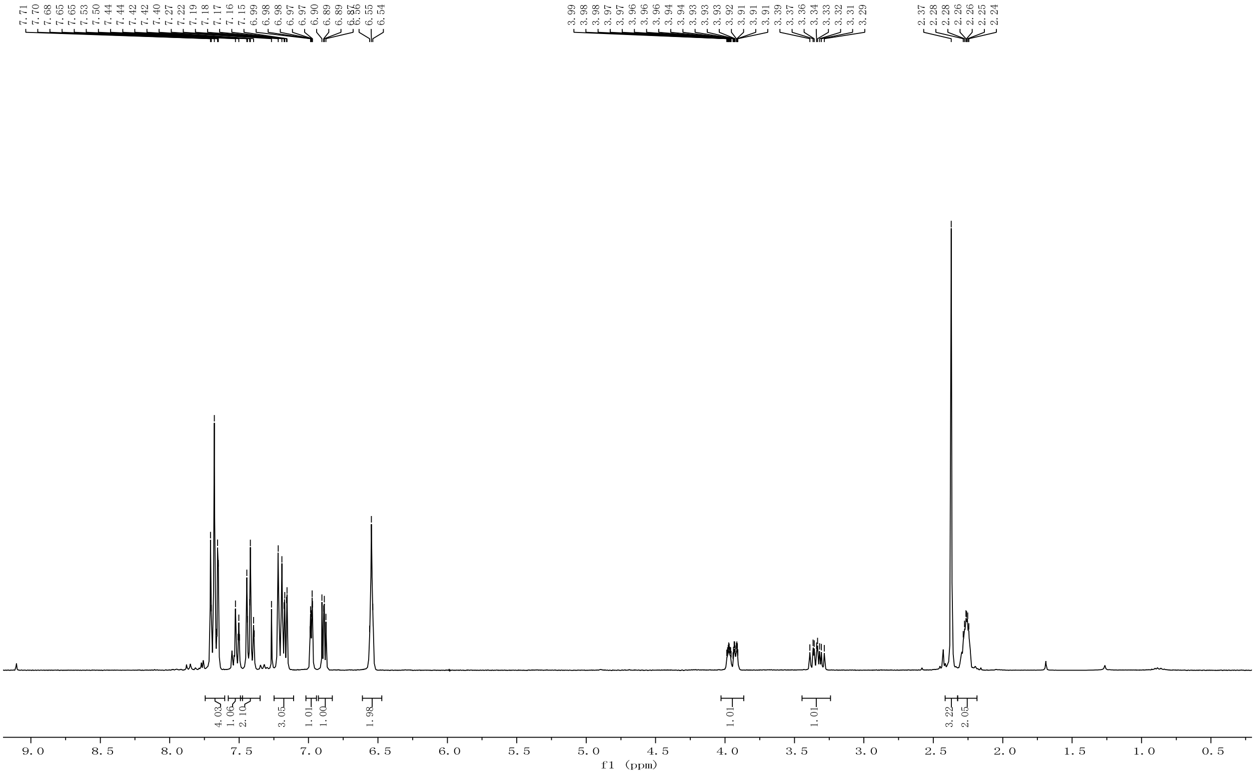

7.71 7.70 7.68 7.65 7.65 7.53 7.50 7.44 7.44 7.42 7.40 7.27 7.22 7.19 7.18 7.17 7.16 7.15 6.99 6.98 6.98 6.97 6.97 6.90 6.89 6.89 6.87 6.56 6.55 6.54
3.99 3.98 3.98 3.97 3.97 3.96 3.96 3.96 3.94 3.94 3.93 3.93 3.93 3.92 3.91 3.91 3.91 3.39 3.37 3.36 3.34 3.33 3.32 3.31 3.29
2.37 2.28 2.28 2.26 2.26 2.25 2.24
4.03 1.06 2.10 3.05 1.01 1.00 1.98 1.01 1.01 3.22 2.05
9.0 8.5 8.0 7.5 7.0 6.5 6.0 5.5 5.0 4.5 4.0 3.5 3.0 2.5 2.0 1.5 1.0 0.5
f1 (ppm)


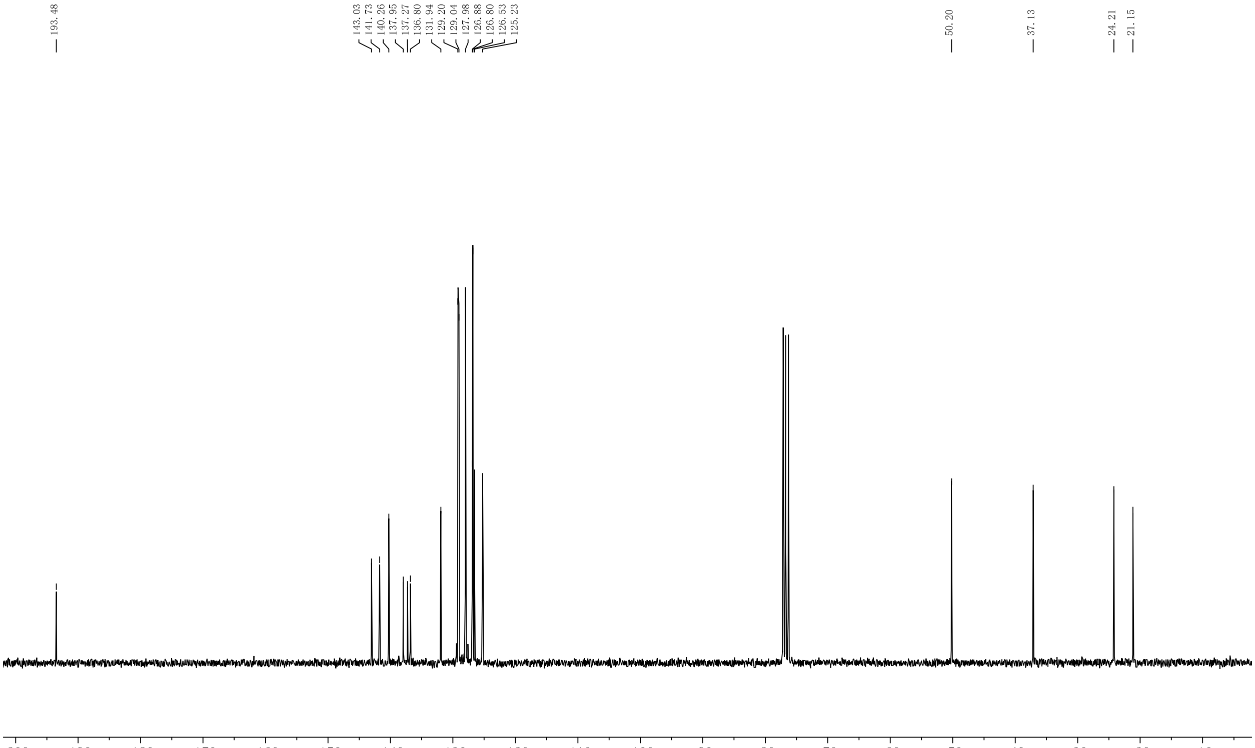

193.48
143.03 141.73 140.26 137.95 137.27 136.80 131.94 129.20 129.04 127.98 126.88 126.80 126.53 125.23
50.20
37.13
24.21 21.15
200 190 180 170 160 150 140 130 120 110 100 90 80 70 60 50 40 30 20 10
f1 (ppm)

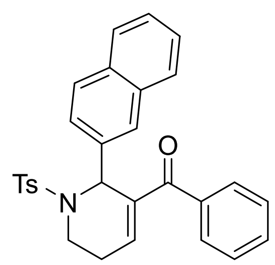
Ts
N
O

**3aq**

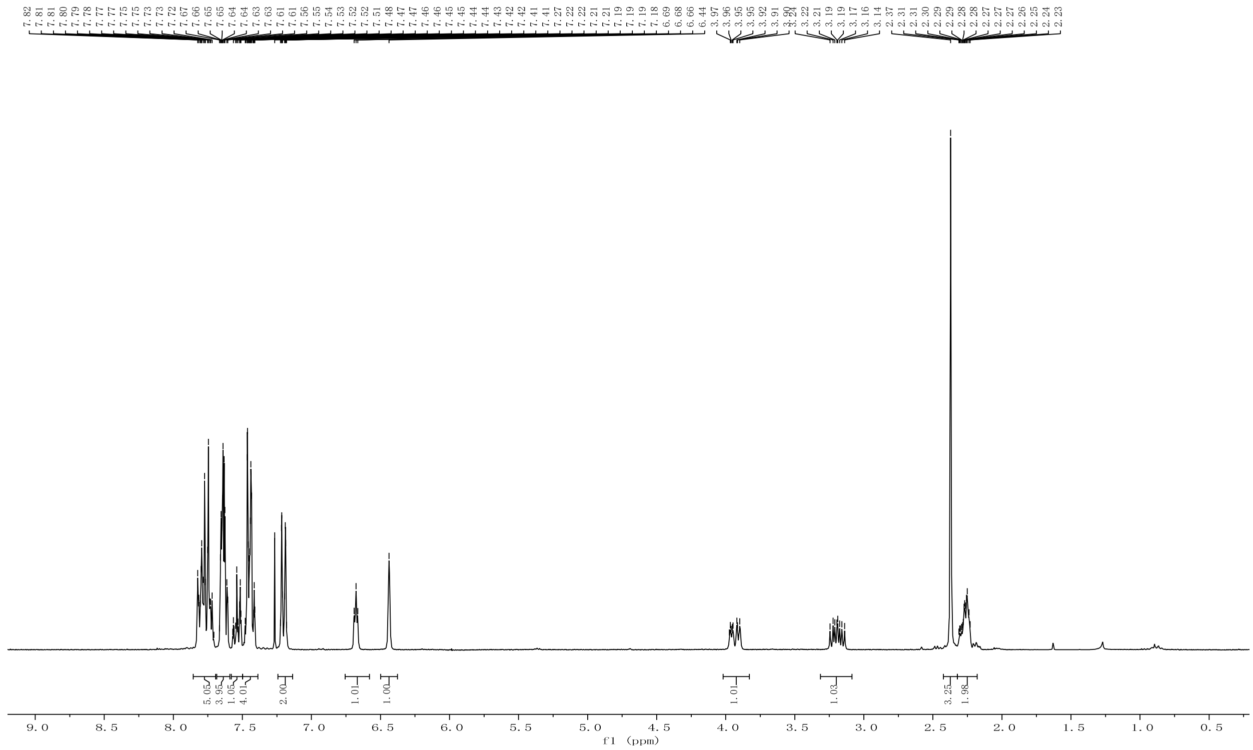
7.82 7.81 7.81 7.80 7.79 7.78 7.77 7.77 7.75 7.75 7.73 7.73 7.72 7.67 7.66 7.65 7.65 7.64 7.64 7.63 7.63 7.61 7.61 7.56 7.55 7.54 7.53 7.52 7.52 7.51 7.48 7.47 7.47 7.46 7.46 7.45 7.45 7.44 7.44 7.43 7.42 7.42 7.41 7.41 7.27 7.22 7.22 7.21 7.21 7.19 7.19 7.19 7.18 6.69 6.68 6.66 6.44
3.97 3.96 3.95 3.95 3.92 3.91
3.20 3.21 3.22 3.21 3.19 3.19 3.17 3.16 3.14
2.37 2.31 2.30 2.29 2.29 2.28 2.28 2.27 2.27 2.26 2.25 2.24 2.23
5.05 3.95 1.05 4.01 2.00 1.01 1.00 1.01 1.03 3.25 1.98
9.0 8.5 8.0 7.5 7.0 6.5 6.0 5.5 5.0 4.5 4.0 3.5 3.0 2.5 2.0 1.5 1.0 0.5
f1 (ppm)

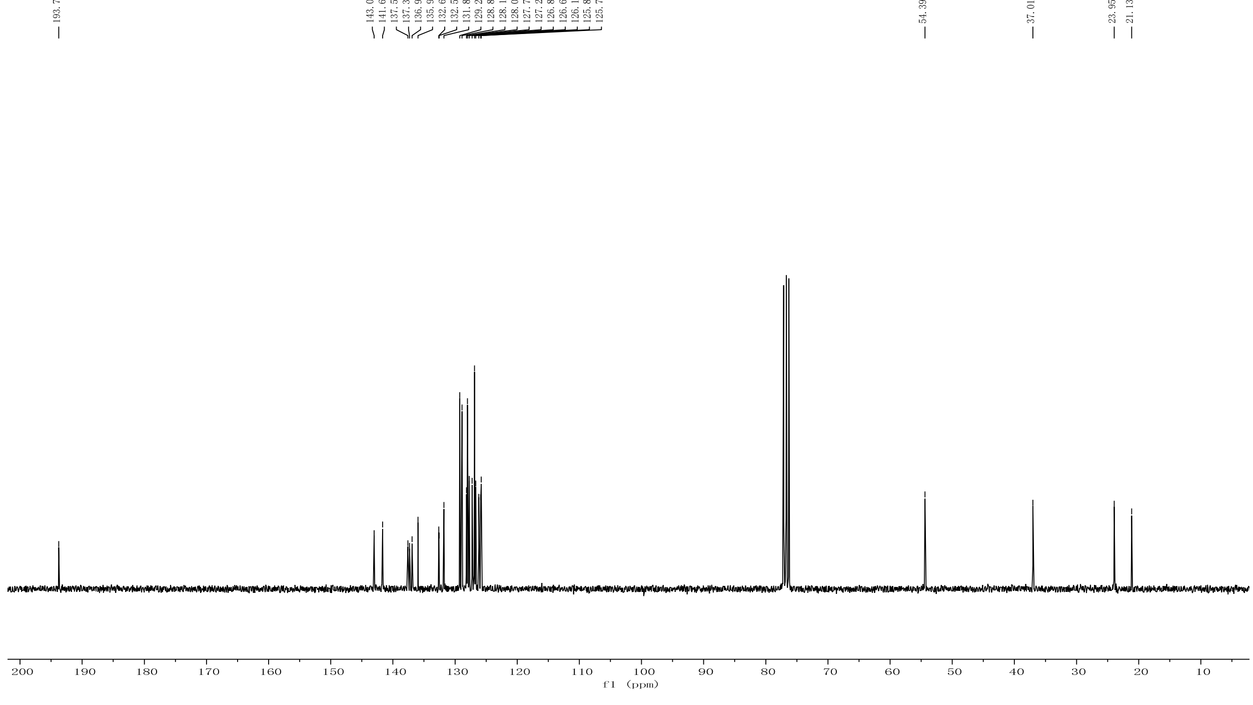
193.77
143.02 141.67 137.58 137.34 136.91 135.95 132.63 132.58 131.80 129.24 128.88 128.17 128.00 127.71 127.25 126.88 126.67 126.17 125.86 125.78
54.39
37.01
23.95 21.13
200 190 180 170 160 150 140 130 120 110 100 90 80 70 60 50 40 30 20 10
f1 (ppm)

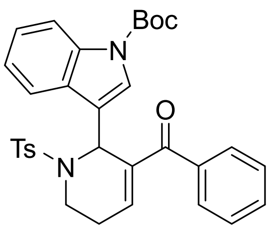
Boc
N
O
Ts
N

**3ar**

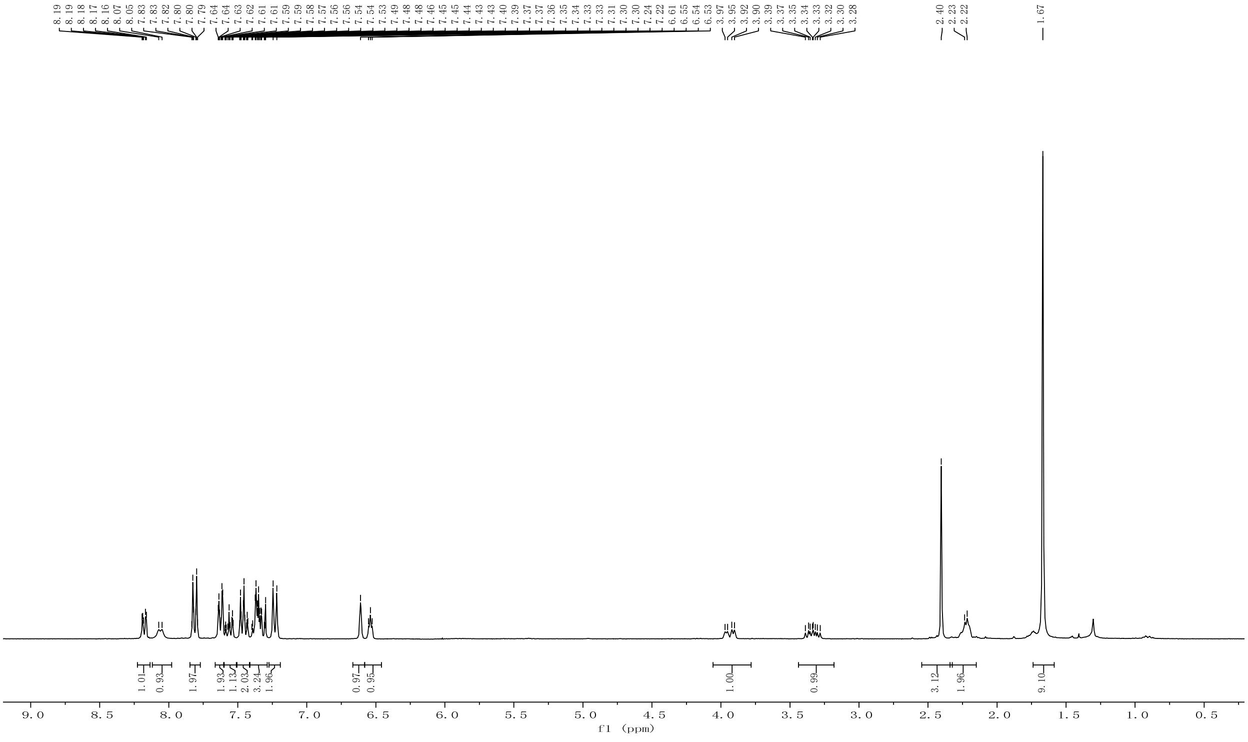
8.19
8.19
8.18
8.17
8.16
8.07
8.05
7.83
7.83
7.82
7.80
7.80
7.79
7.64
7.64
7.63
7.62
7.61
7.61
7.59
7.59
7.58
7.57
7.56
7.56
7.54
7.54
7.54
7.53
7.49
7.48
7.46
7.45
7.45
7.44
7.43
7.43
7.40
7.39
7.37
7.37
7.36
7.35
7.34
7.33
7.33
7.31
7.30
7.30
7.24
7.22
6.61
6.55
6.54
6.53
3.97
3.95
3.92
3.90
3.39
3.37
3.35
3.34
3.33
3.32
3.30
3.28
2.40
2.23
2.22
1.67
1.01
0.93
1.97
1.93
1.13
2.03
3.24
1.96
0.97
0.95
1.00
0.99
3.12
1.96
9.10
9.0
8.5
8.0
7.5
7.0
6.5
6.0
5.5
5.0
4.5
4.0
3.5
3.0
2.5
2.0
1.5
1.0
0.5
f1 (ppm)

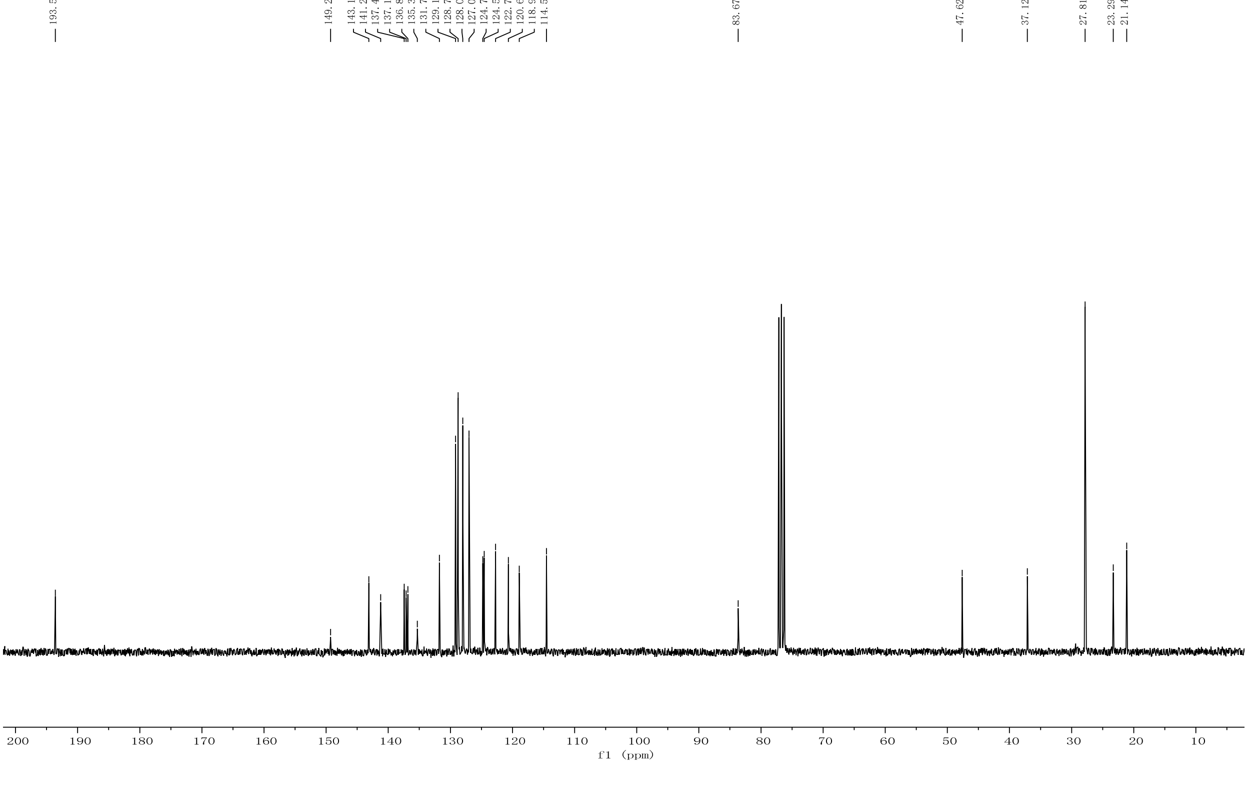
193.58
149.29
143.12
141.23
137.44
137.11
136.85
135.32
131.78
129.17
128.75
128.00
127.01
124.78
124.55
122.74
120.67
118.92
114.54
83.67
47.62
37.12
27.81
23.29
21.14
200
190
180
170
160
150
140
130
120
110
100
90
80
70
60
50
40
30
20
10
f1 (ppm)

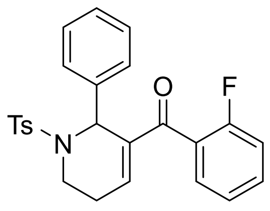

**3ba**

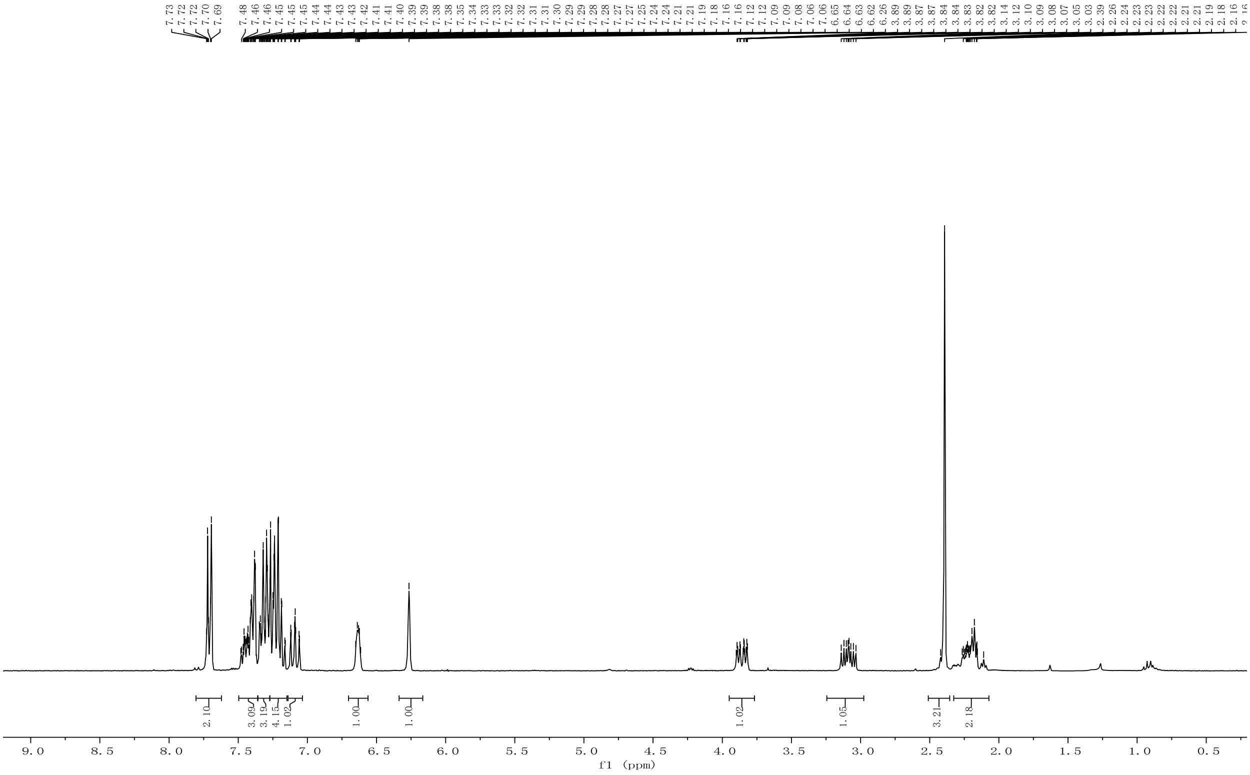

7.73
7.72
7.72
7.70
7.69
7.48
7.46
7.46
7.45
7.45
7.45
7.44
7.44
7.43
7.43
7.42
7.41
7.41
7.40
7.39
7.39
7.38
7.38
7.35
7.34
7.33
7.33
7.32
7.32
7.31
7.31
7.30
7.29
7.29
7.28
7.28
7.27
7.27
7.25
7.24
7.24
7.21
7.21
7.19
7.18
7.16
7.16
7.12
7.12
7.09
7.09
7.08
7.06
7.06
6.65
6.64
6.63
6.62
6.26
3.89
3.89
3.87
3.84
3.84
3.83
3.82
3.82
3.14
3.12
3.10
3.09
3.08
3.07
3.05
3.03
2.39
2.26
2.24
2.23
2.23
2.22
2.22
2.21
2.21
2.19
2.18
2.16
2.10
3.09
3.19
4.15
1.02
1.00
1.00
1.02
1.05
3.21
2.18
9.0 8.5 8.0 7.5 7.0 6.5 6.0 5.5 5.0 4.5 4.0 3.5 3.0 2.5 2.0 1.5 1.0 0.5
f1 (ppm)


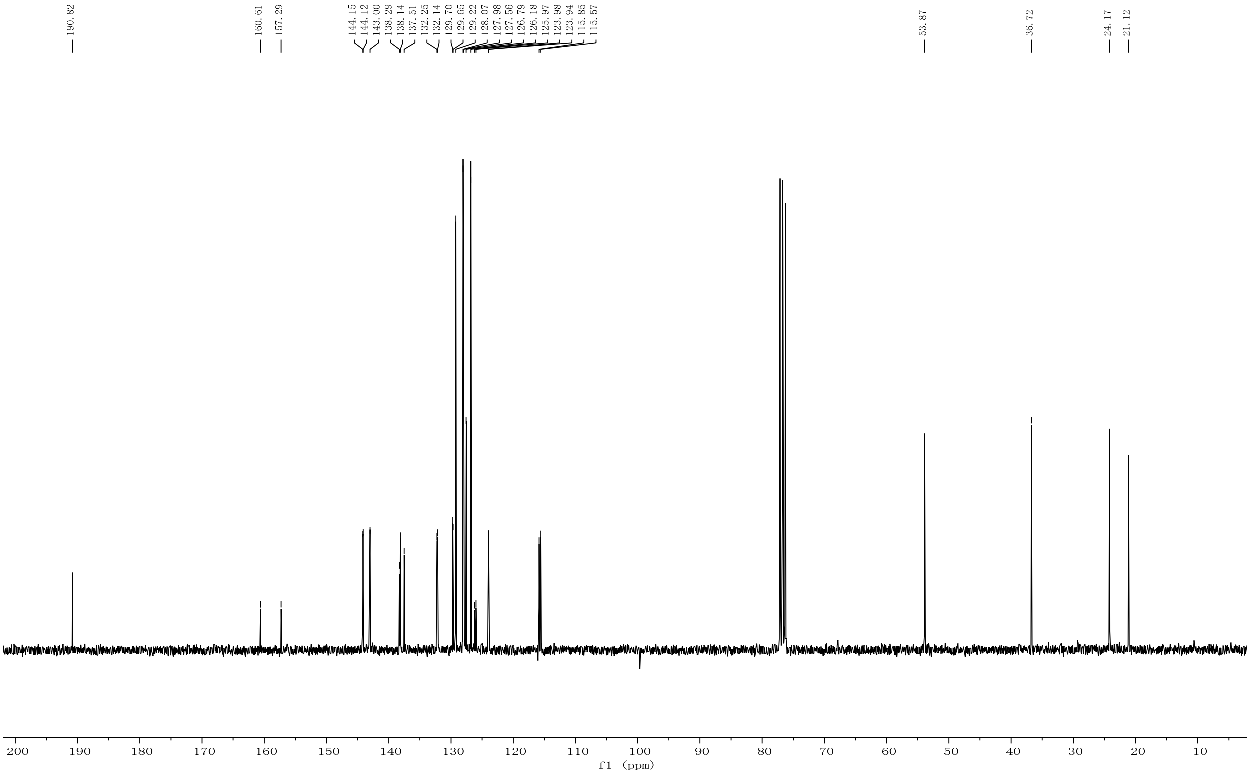

190.82
160.61
157.29
144.15
144.12
143.00
138.29
138.14
137.51
132.25
132.14
129.70
129.65
129.22
128.07
127.98
127.56
126.79
126.18
125.97
123.98
123.94
115.85
115.57
53.87
36.72
24.17
21.12
200 190 180 170 160 150 140 130 120 110 100 90 80 70 60 50 40 30 20 10
f1 (ppm)

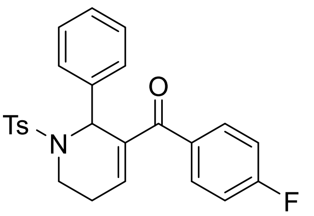

Ts
N
O
F


**3ca**

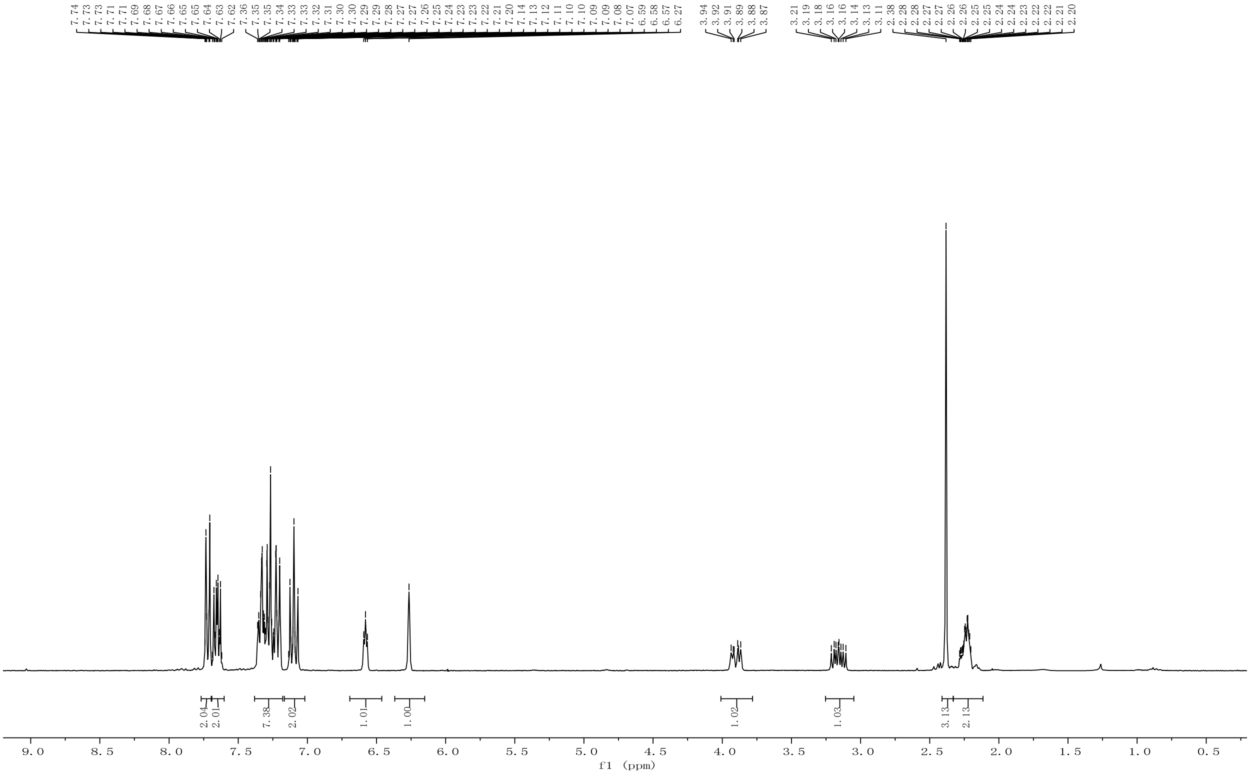

7.74 7.73 7.71 7.71 7.69 7.68 7.67 7.66 7.65 7.65 7.64 7.63 7.62 7.36 7.35 7.35 7.34 7.33 7.33 7.32 7.31 7.30 7.29 7.29 7.28 7.27 7.27 7.26 7.25 7.24 7.23 7.23 7.22 7.21 7.20 7.14 7.13 7.12 7.11 7.10 7.10 7.09 7.09 7.08 7.07 6.59 6.58 6.57 6.27
3.94 3.92 3.91 3.89 3.88 3.87
3.21 3.19 3.18 3.16 3.16 3.14 3.13 3.11
2.38 2.28 2.28 2.28 2.27 2.27 2.26 2.26 2.25 2.25 2.24 2.24 2.23 2.23 2.22 2.22 2.21 2.20
2.04 2.01 7.38 2.02 1.01 1.00 1.02 1.03 3.13 2.13
9.0 8.5 8.0 7.5 7.0 6.5 6.0 5.5 5.0 4.5 4.0 3.5 3.0 2.5 2.0 1.5 1.0 0.5
f1 (ppm)


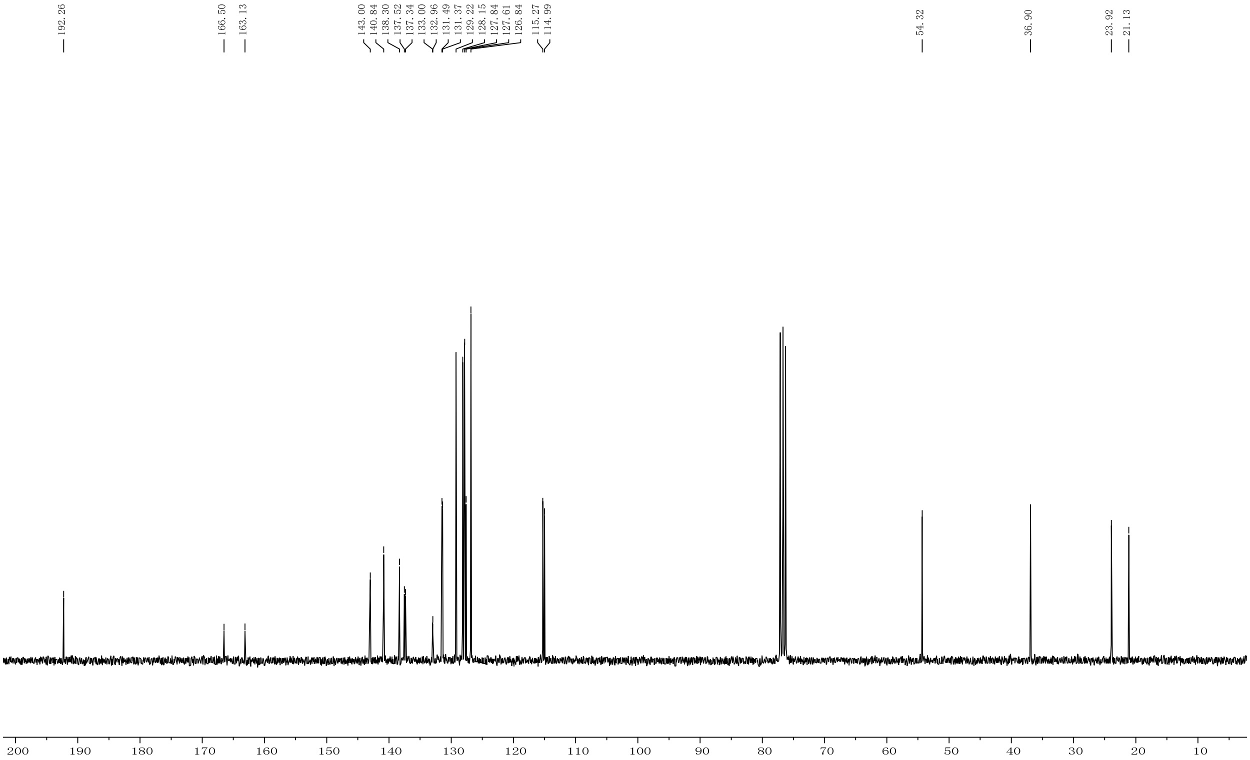

192.26
166.50 163.13
143.00 140.84 138.30 137.52 137.34 133.00 132.96 131.49 131.37 129.22 128.15 127.84 127.61 126.84 115.27 114.99
54.32
36.90
23.92 21.13
200 190 180 170 160 150 140 130 120 110 100 90 80 70 60 50 40 30 20 10
f1 (ppm)

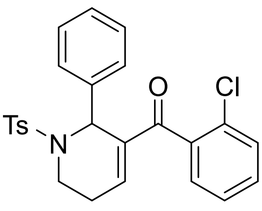


**3da**

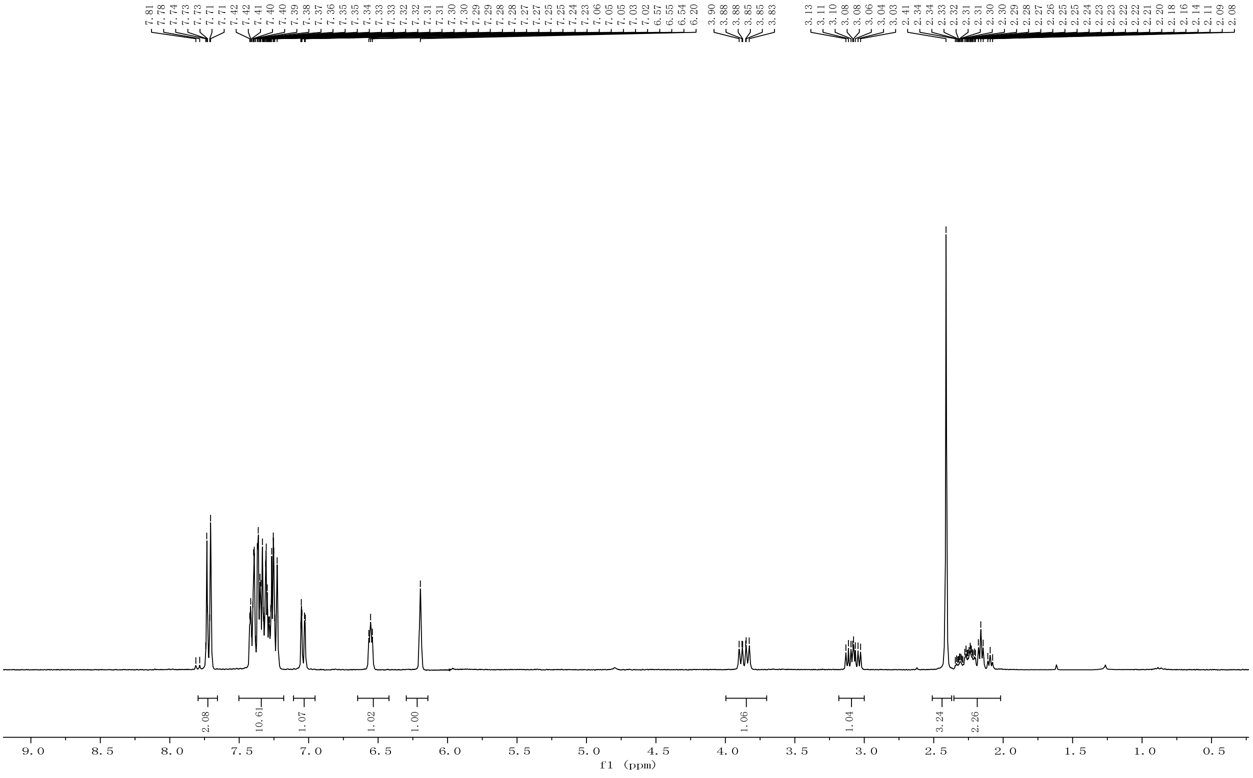

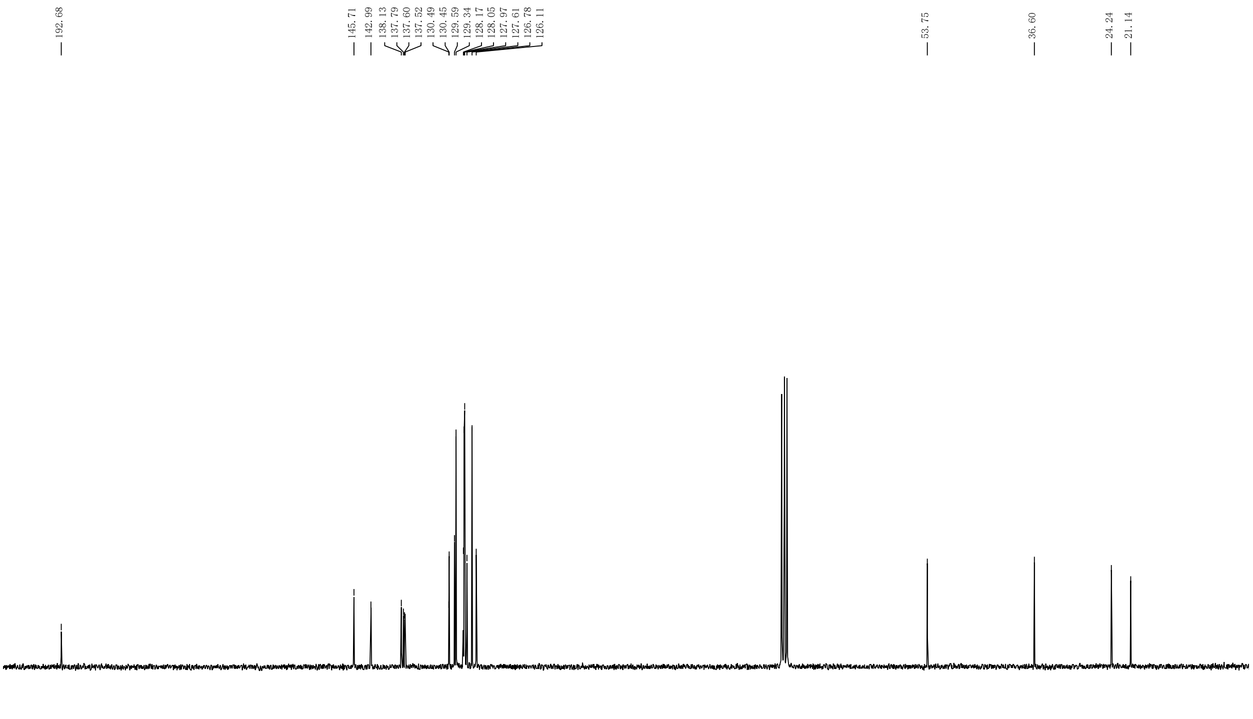

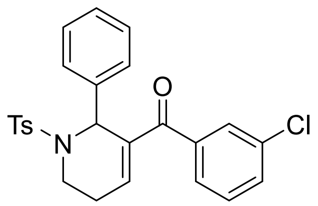
O
Ts
N
Cl

**3ea**

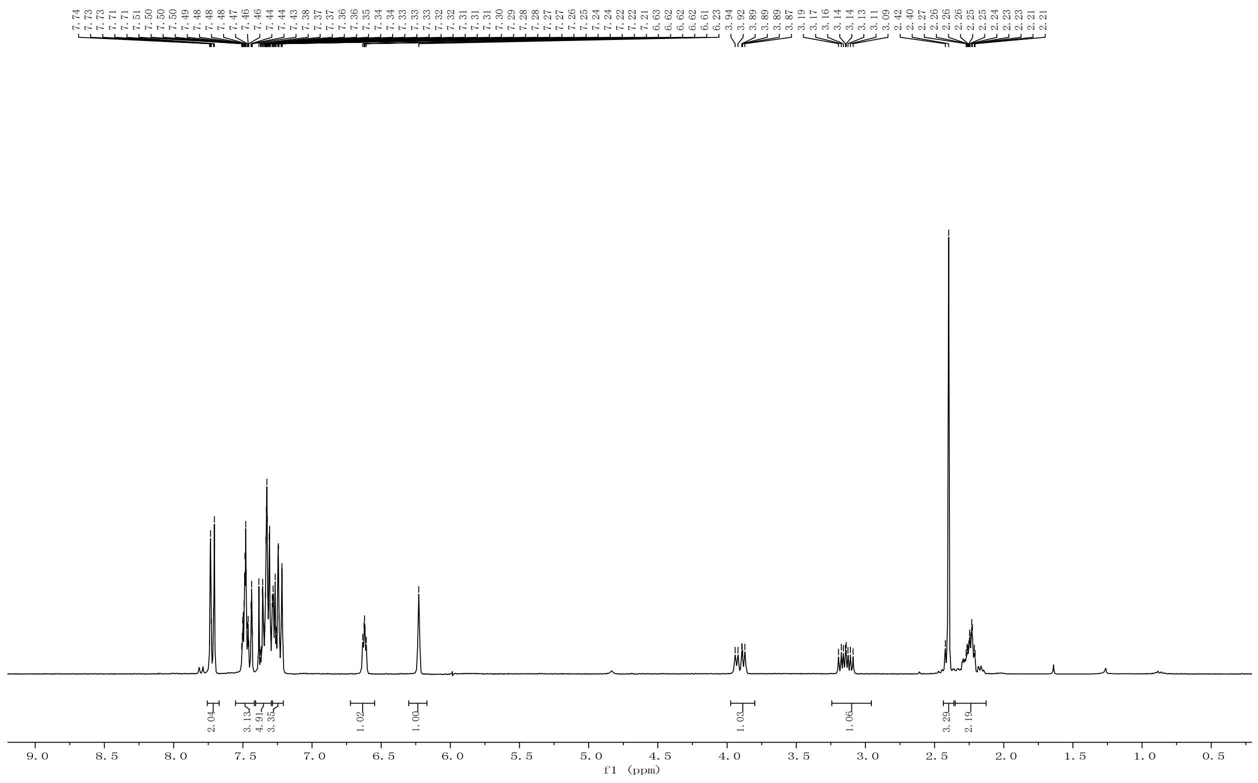
7.74 7.73 7.73 7.71 7.71 7.51 7.50 7.50 7.50 7.49 7.48 7.48 7.47 7.46 7.46 7.44 7.43 7.38 7.37 7.37 7.36 7.36 7.35 7.34 7.34 7.33 7.33 7.33 7.32 7.32 7.31 7.31 7.31 7.30 7.29 7.28 7.28 7.27 7.27 7.26 7.25 7.24 7.24 7.22 7.22 7.21 6.63 6.62 6.62 6.62 6.61 6.23 3.94 3.92 3.89 3.89 3.89 3.87 3.19 3.17 3.16 3.14 3.14 3.13 3.11 3.09 2.42 2.40 2.27 2.26 2.26 2.25 2.25 2.24 2.24 2.23 2.23 2.21 2.21
2.04
3.13
4.91
3.35
1.02
1.00
1.03
1.06
3.29
2.19
9.0 8.5 8.0 7.5 7.0 6.5 6.0 5.5 5.0 4.5 4.0 3.5 3.0 2.5 2.0 1.5 1.0 0.5
f1 (ppm)

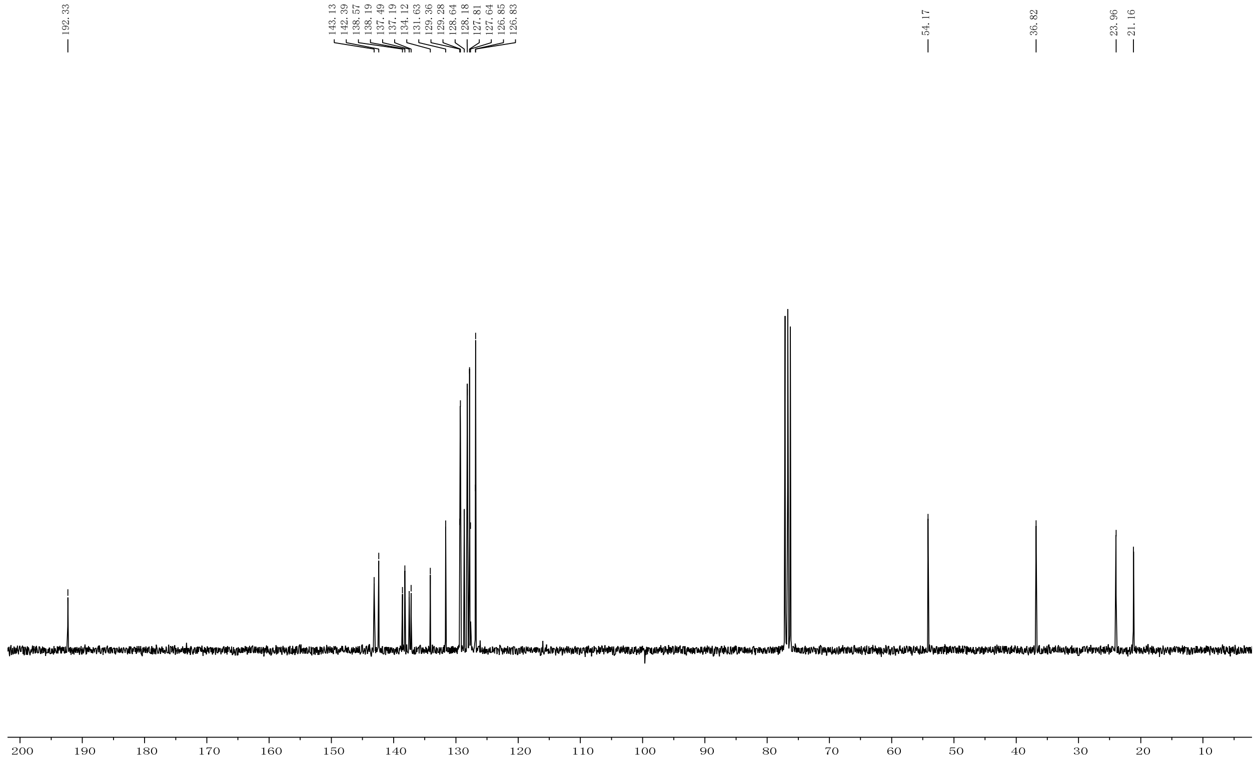
192.33
143.13 142.39 138.57 138.19 137.49 137.19 134.12 131.63 129.36 129.28 128.64 128.18 127.81 127.64 126.85 126.83
54.17
36.82
23.96
21.16
200 190 180 170 160 150 140 130 120 110 100 90 80 70 60 50 40 30 20 10
f1 (ppm)

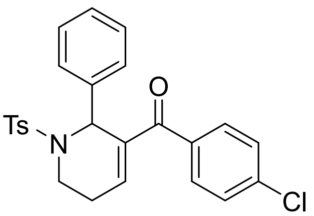
Ts
N
O
Cl

**3fa**

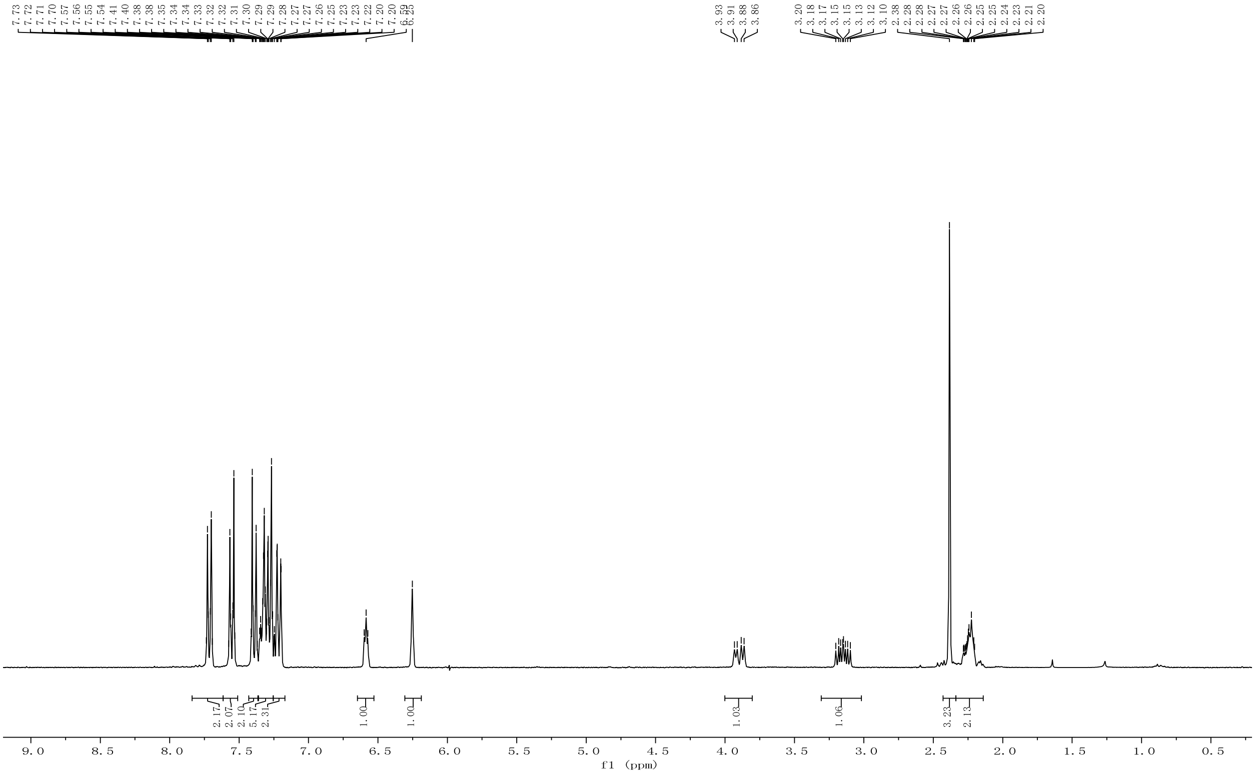
7.73 7.72 7.71 7.70 7.57 7.56 7.55 7.54 7.41 7.40 7.38 7.38 7.35 7.34 7.34 7.33 7.32 7.32 7.31 7.30 7.29 7.29 7.28 7.27 7.27 7.26 7.25 7.23 7.23 7.22 7.20 6.59 6.25
3.93 3.91 3.88 3.86
3.20 3.18 3.17 3.15 3.15 3.13 3.12 3.10
2.38 2.28 2.28 2.27 2.27 2.26 2.26 2.25 2.25 2.24 2.23 2.21 2.20
2.17 2.07 2.10 5.17 2.31
1.00
1.00
1.03
1.06
3.23 2.13
9.0 8.5 8.0 7.5 7.0 6.5 6.0 5.5 5.0 4.5 4.0 3.5 3.0 2.5 2.0 1.5 1.0 0.5
f1 (ppm)

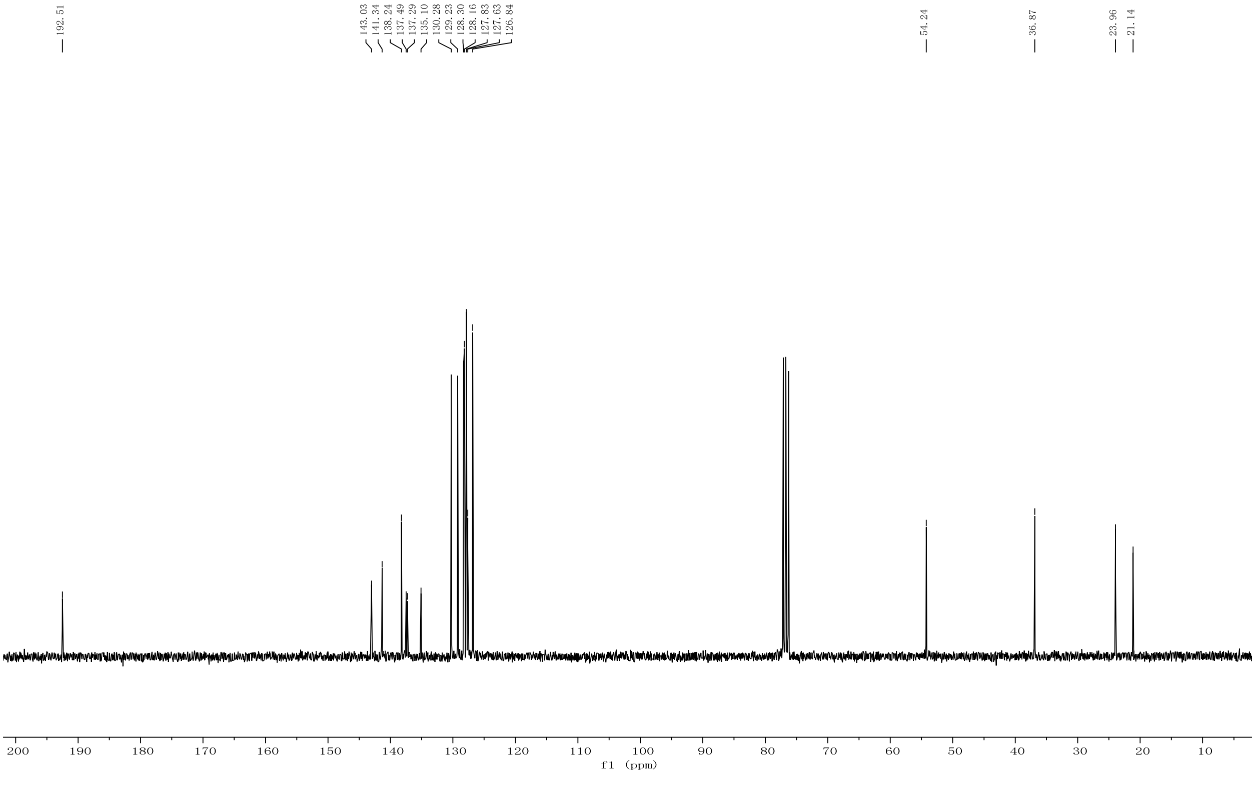
192.51
143.03 141.34 138.24 137.49 137.29 135.10 130.28 129.23 128.30 128.16 127.83 127.63 126.84
54.24
36.87
23.96
21.14
200 190 180 170 160 150 140 130 120 110 100 90 80 70 60 50 40 30 20 10
f1 (ppm)

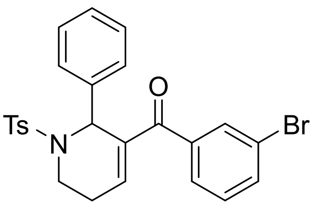

Ts
N
O
Br


**3ga**

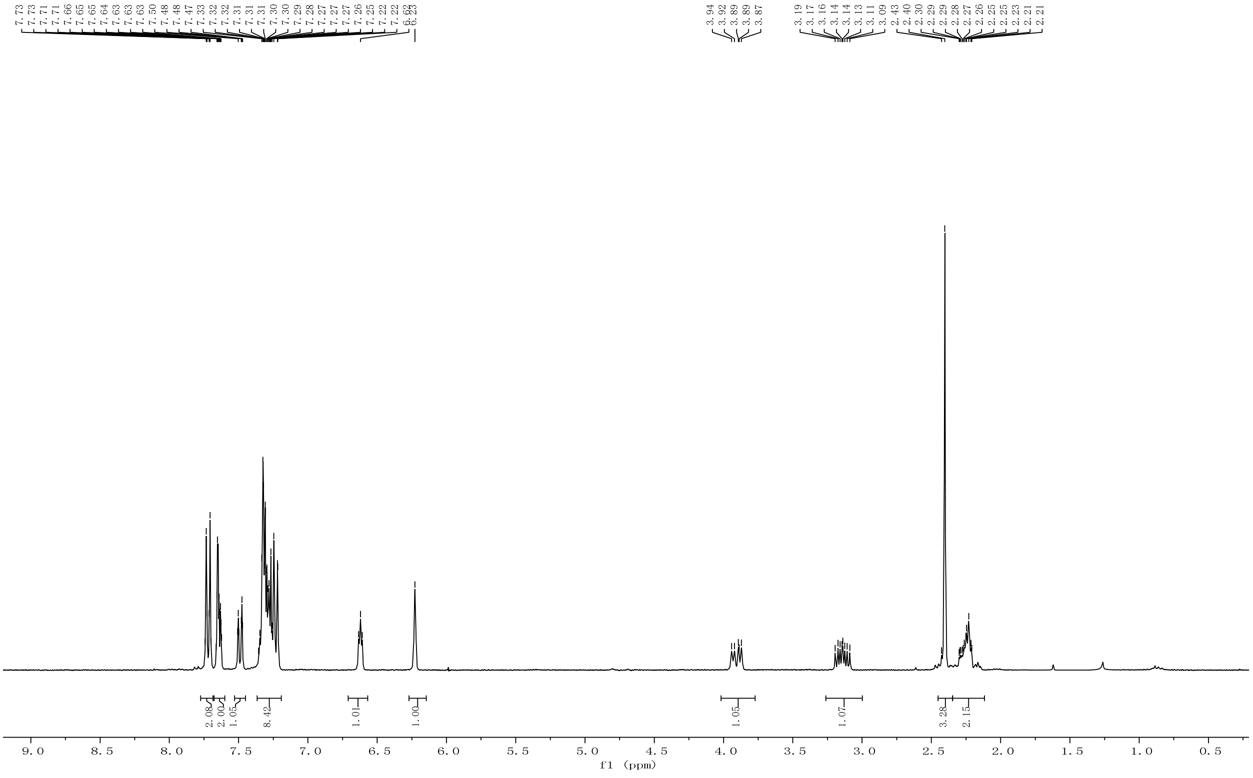

7.73 7.73 7.71 7.71 7.66 7.65 7.65 7.64 7.63 7.63 7.63 7.50 7.48 7.48 7.47 7.33 7.32 7.32 7.31 7.31 7.31 7.30 7.29 7.28 7.27 7.27 7.27 7.26 7.25 7.22 7.22 6.23
3.94 3.92 3.89 3.89 3.87
3.19 3.17 3.16 3.14 3.14 3.13 3.11 3.09
2.43 2.40 2.30 2.29 2.29 2.28 2.27 2.26 2.25 2.25 2.23 2.21 2.21
2.08 2.00 1.05 8.42 1.01 1.00 1.05 1.07 3.28 2.15
9.0 8.5 8.0 7.5 7.0 6.5 6.0 5.5 5.0 4.5 4.0 3.5 3.0 2.5 2.0 1.5 1.0 0.5
f1 (ppm)


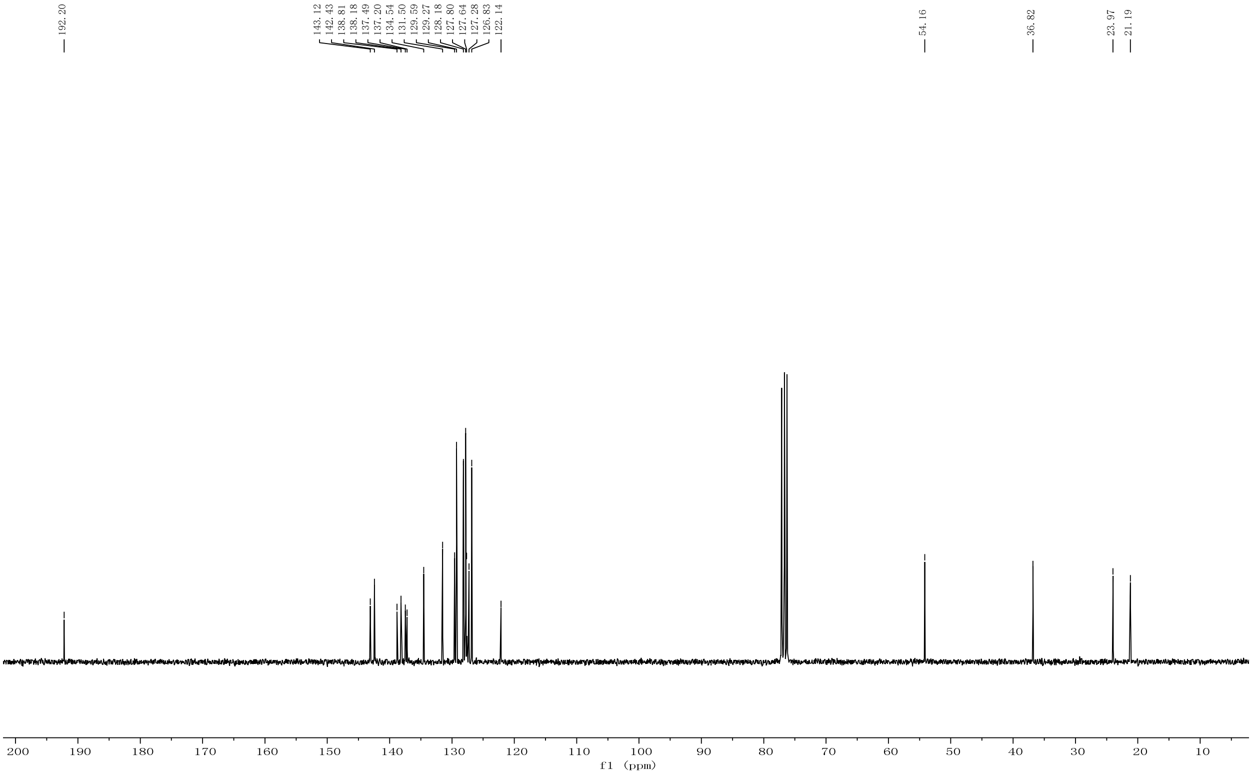

192.20
143.12 142.43 138.81 138.18 137.49 137.20 134.54 131.50 129.59 129.27 128.18 127.80 127.64 127.28 126.83 122.14
54.16
36.82
23.97 21.19
200 190 180 170 160 150 140 130 120 110 100 90 80 70 60 50 40 30 20 10
f1 (ppm)

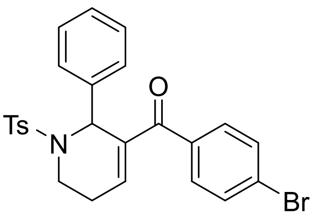

Ts
N
O
Br


**3ha**

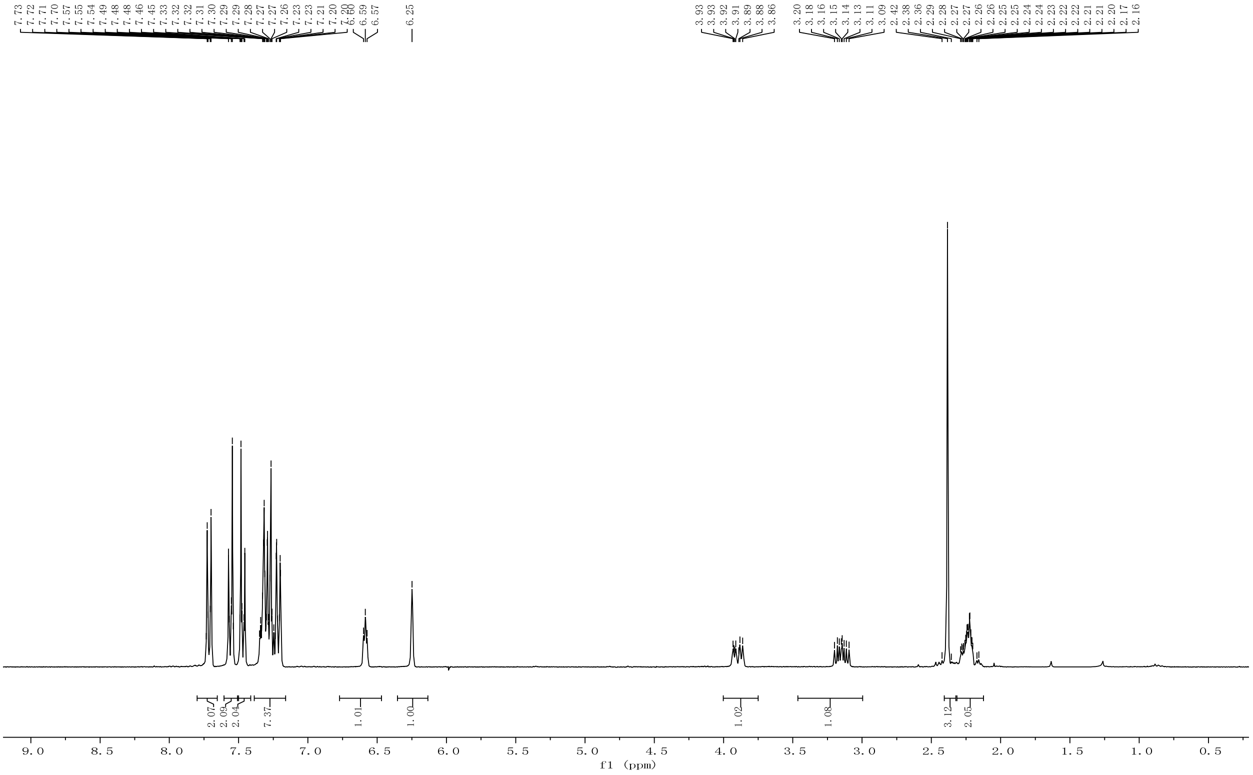

7.73 7.72 7.71 7.70 7.57 7.55 7.54 7.49 7.48 7.48 7.46 7.45 7.33 7.32 7.32 7.31 7.30 7.29 7.29 7.28 7.27 7.27 7.26 7.23 7.23 7.21 7.20 7.20 6.60 6.59 6.57
6.25
3.93 3.93 3.92 3.91 3.89 3.88 3.86
3.20 3.18 3.16 3.15 3.14 3.13 3.11 3.09
2.42 2.38 2.36 2.29 2.28 2.27 2.27 2.26 2.26 2.25 2.25 2.24 2.24 2.23 2.23 2.22 2.22 2.21 2.21 2.20 2.17 2.16
2.07 2.09 2.04 7.37 1.01 1.00 1.02 1.08 3.12 2.05
f1 (ppm)


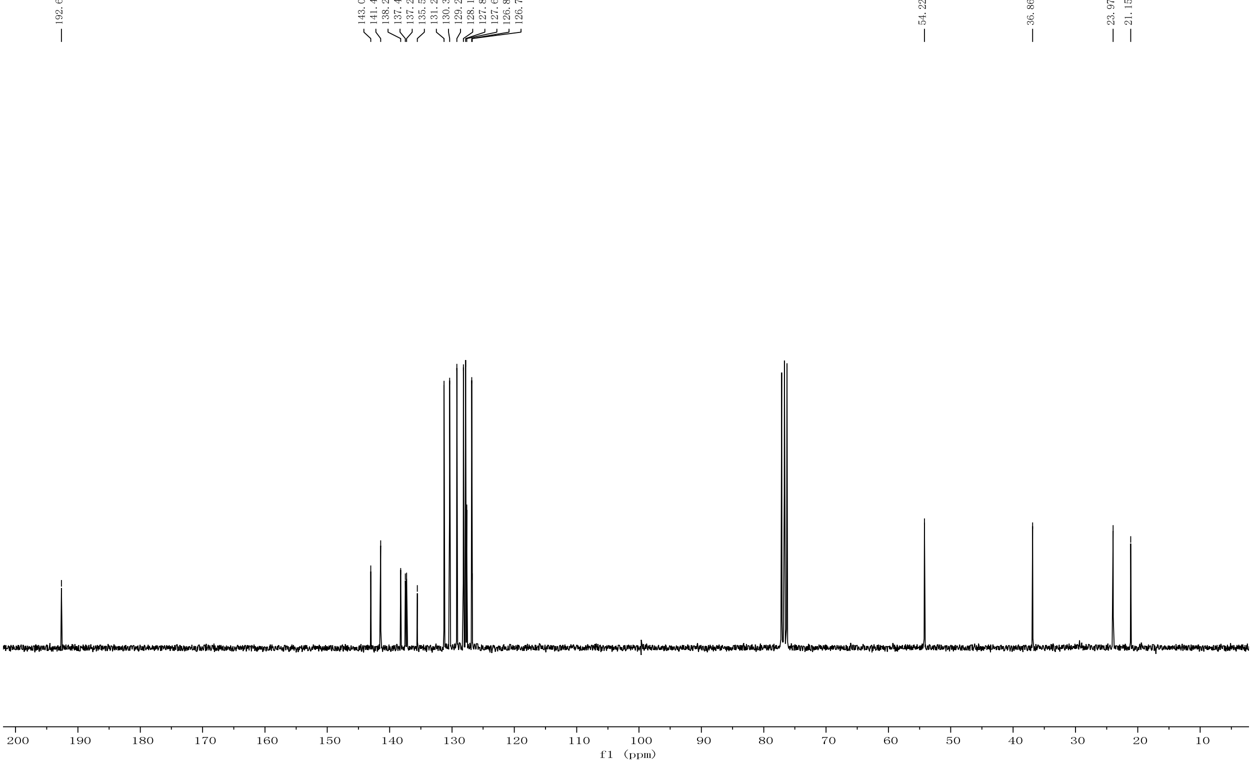

192.65
143.03 141.45 138.23 137.49 137.29 135.56 131.28 130.38 129.23 128.16 127.82 127.63 126.84 126.75
54.22
36.86
23.97 21.15
f1 (ppm)

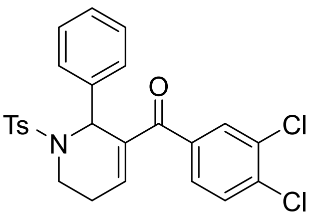
O
Ts
N
Cl
Cl

**3ia**

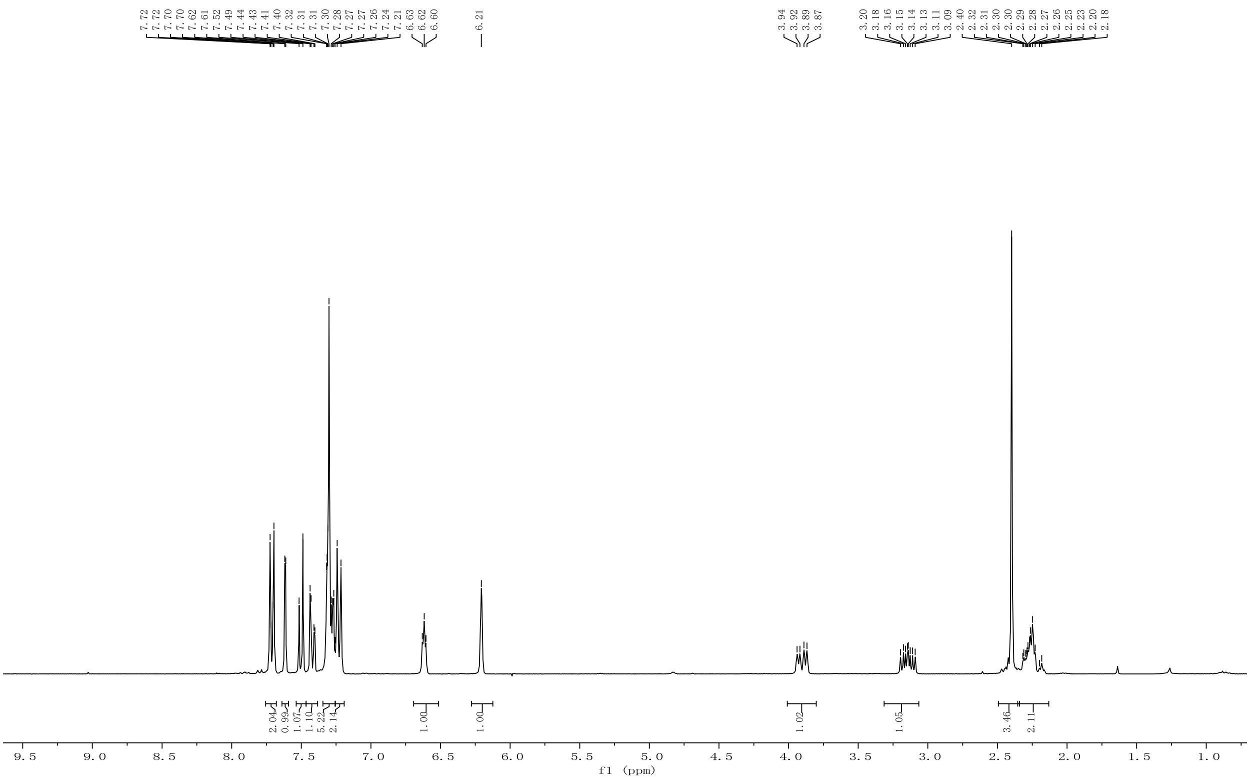
7.72 7.72 7.70 7.70 7.62 7.61 7.52 7.49 7.44 7.43 7.41 7.40 7.32 7.31 7.31 7.30 7.28 7.27 7.27 7.26 7.24 7.21 6.63 6.62 6.60
6.21
3.94 3.92 3.89 3.87
3.20 3.18 3.16 3.15 3.14 3.13 3.11 3.09 2.40 2.32 2.31 2.30 2.30 2.29 2.28 2.27 2.26 2.25 2.23 2.20 2.18
2.04 0.99 1.07 1.10 5.22 2.14
1.00
1.00
1.02
1.05
3.46 2.11
9.5 9.0 8.5 8.0 7.5 7.0 6.5 6.0 5.5 5.0 4.5 4.0 3.5 3.0 2.5 2.0 1.5 1.0
f1 (ppm)

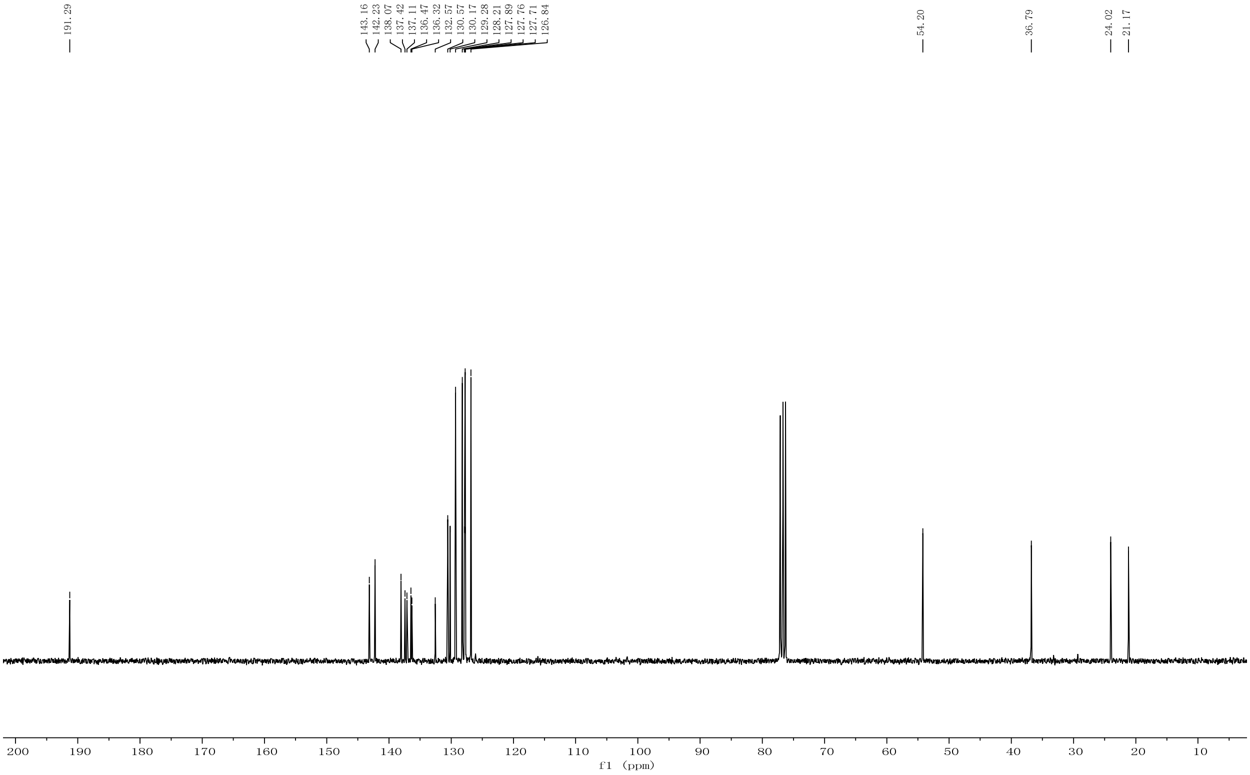
191.29
143.16 142.23 138.07 137.42 137.11 136.47 136.32 132.57 130.57 130.17 129.28 128.21 127.89 127.76 127.71 126.84
54.20
36.79
24.02 21.17
200 190 180 170 160 150 140 130 120 110 100 90 80 70 60 50 40 30 20 10
f1 (ppm)

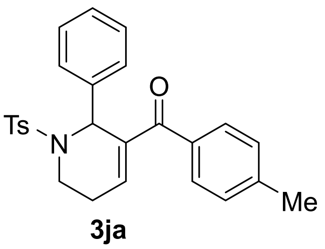


**3ja**

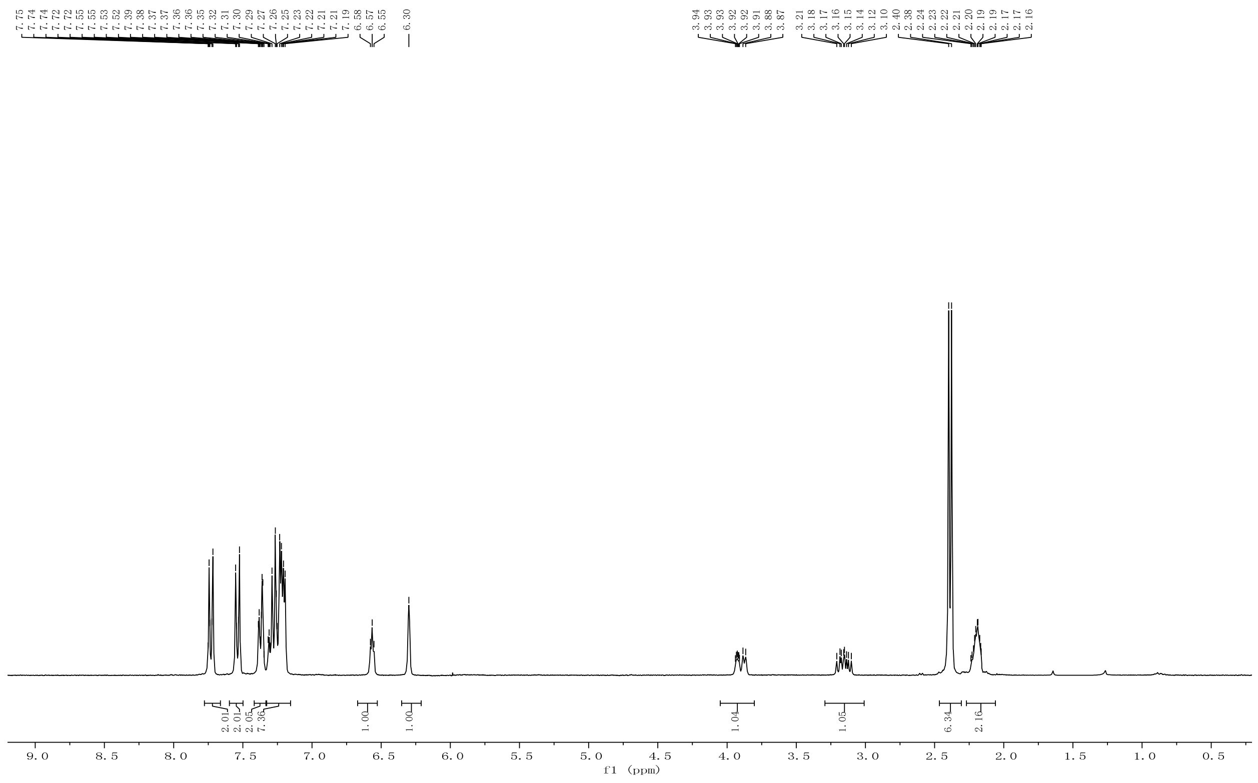

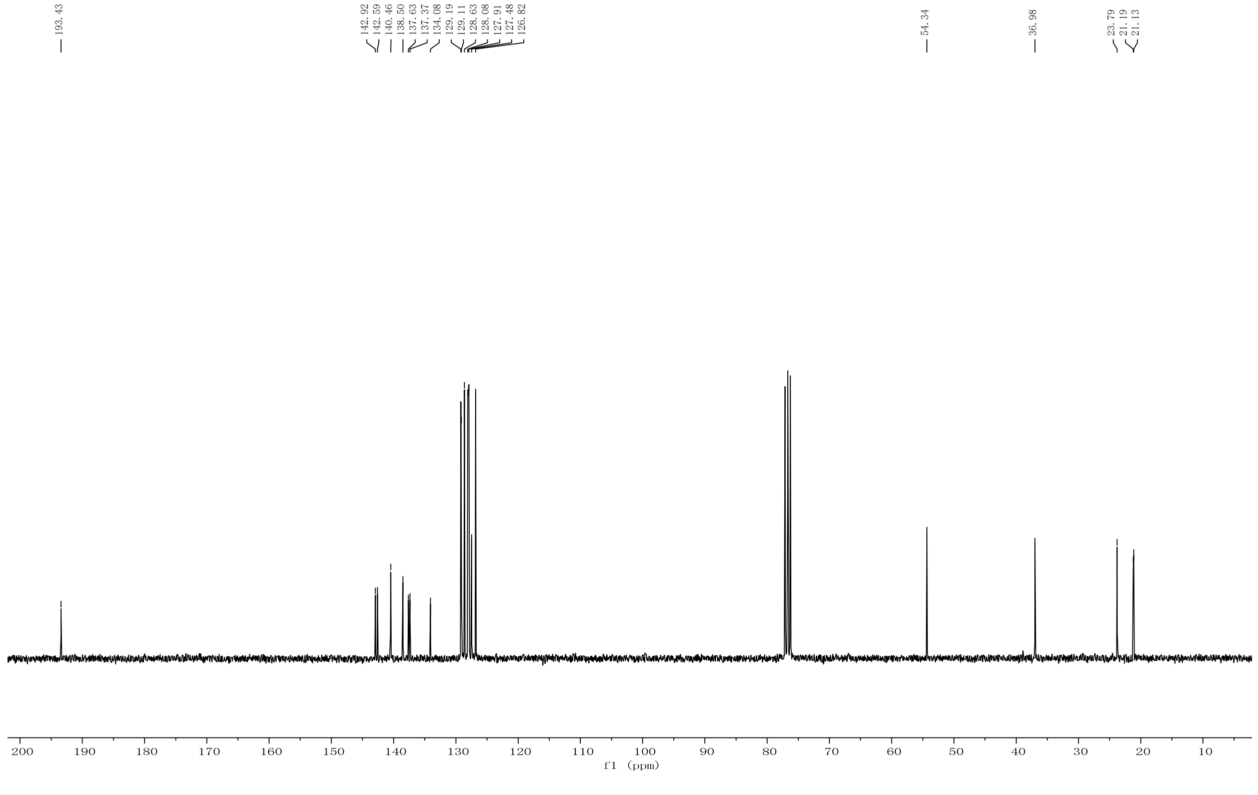

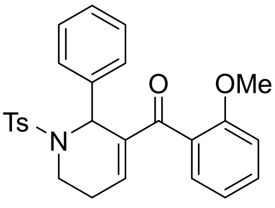
O
OMe
Ts
N

**3ka**

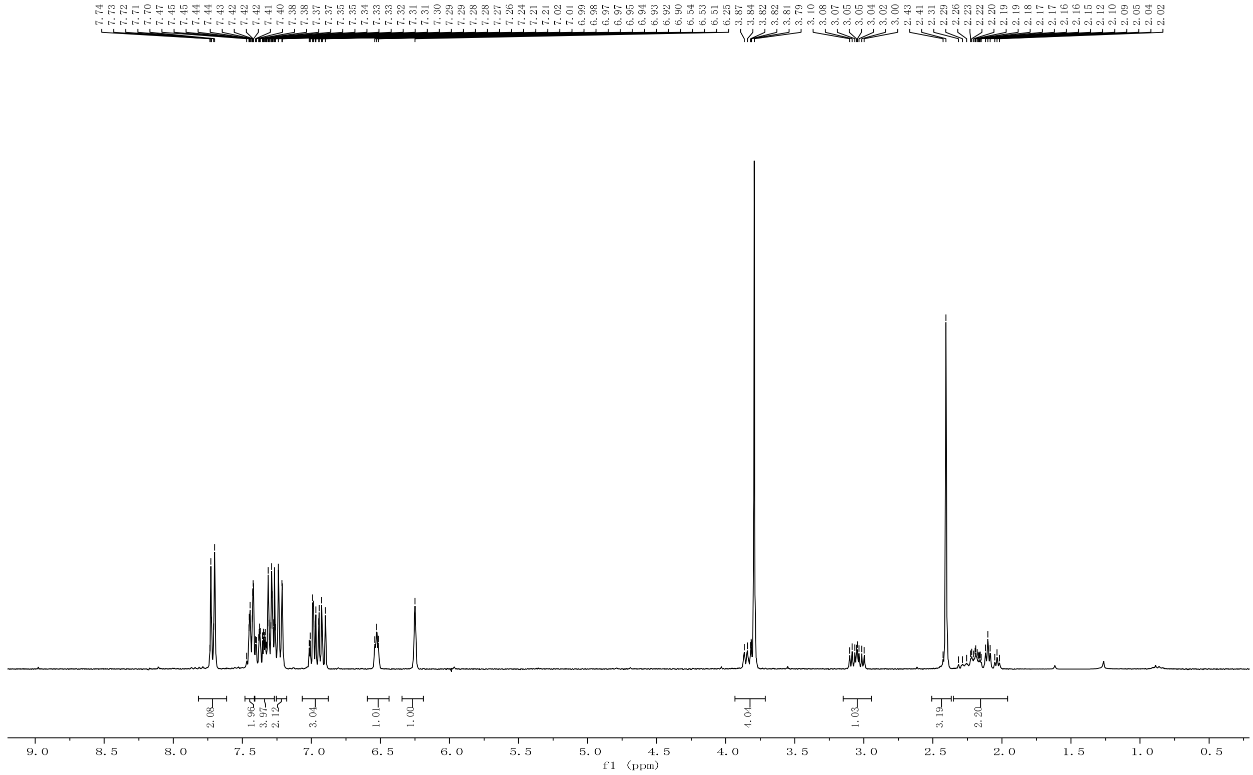
7.74 7.73 7.72 7.71 7.70 7.47 7.45 7.45 7.44 7.44 7.43 7.42 7.42 7.41 7.40 7.38 7.38 7.37 7.37 7.35 7.35 7.34 7.33 7.33 7.32 7.31 7.31 7.30 7.29 7.29 7.28 7.28 7.27 7.26 7.24 7.21 7.21 7.02 7.01 7.01 6.99 6.98 6.97 6.97 6.95 6.94 6.93 6.92 6.90 6.54 6.53 6.51 6.25 3.87 3.84 3.82 3.82 3.81 3.79 3.10 3.08 3.07 3.05 3.05 3.04 3.02 3.00 2.43 2.41 2.31 2.29 2.26 2.23 2.22 2.20 2.19 2.19 2.18 2.17 2.16 2.16 2.15 2.12 2.10 2.09 2.05 2.04 2.02
2.08
1.96
3.97
2.12
3.04
1.01
1.00
4.04
1.03
3.19
2.20
9.0 8.5 8.0 7.5 7.0 6.5 6.0 5.5 5.0 4.5 4.0 3.5 3.0 2.5 2.0 1.5 1.0 0.5
f1 (ppm)

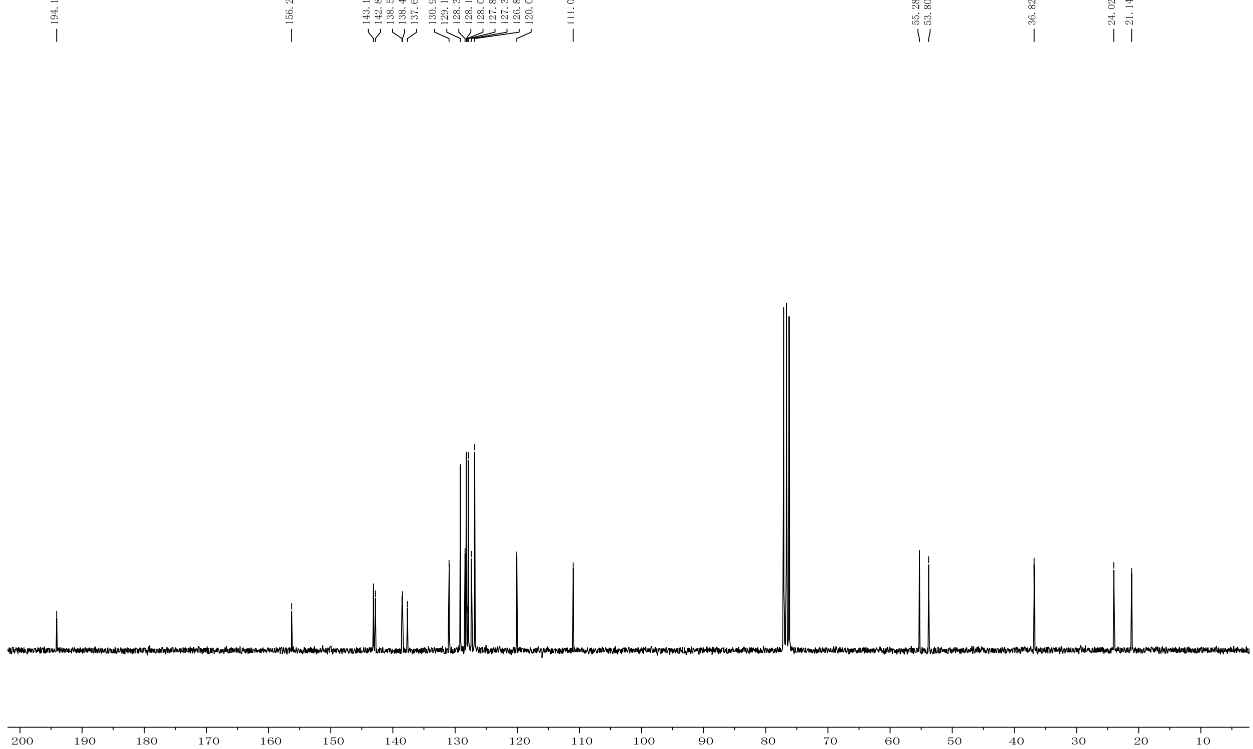
194.12
156.28
143.13 142.82 138.55 138.45 137.66
130.95 129.14 128.39 128.18 128.05 127.86 127.38 126.85 120.07
111.01
55.28 53.80
36.82
24.02 21.14
200 190 180 170 160 150 140 130 120 110 100 90 80 70 60 50 40 30 20 10
f1 (ppm)

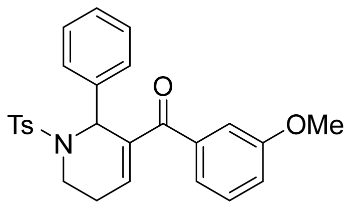
Ts
N
O
OMe

**3la**

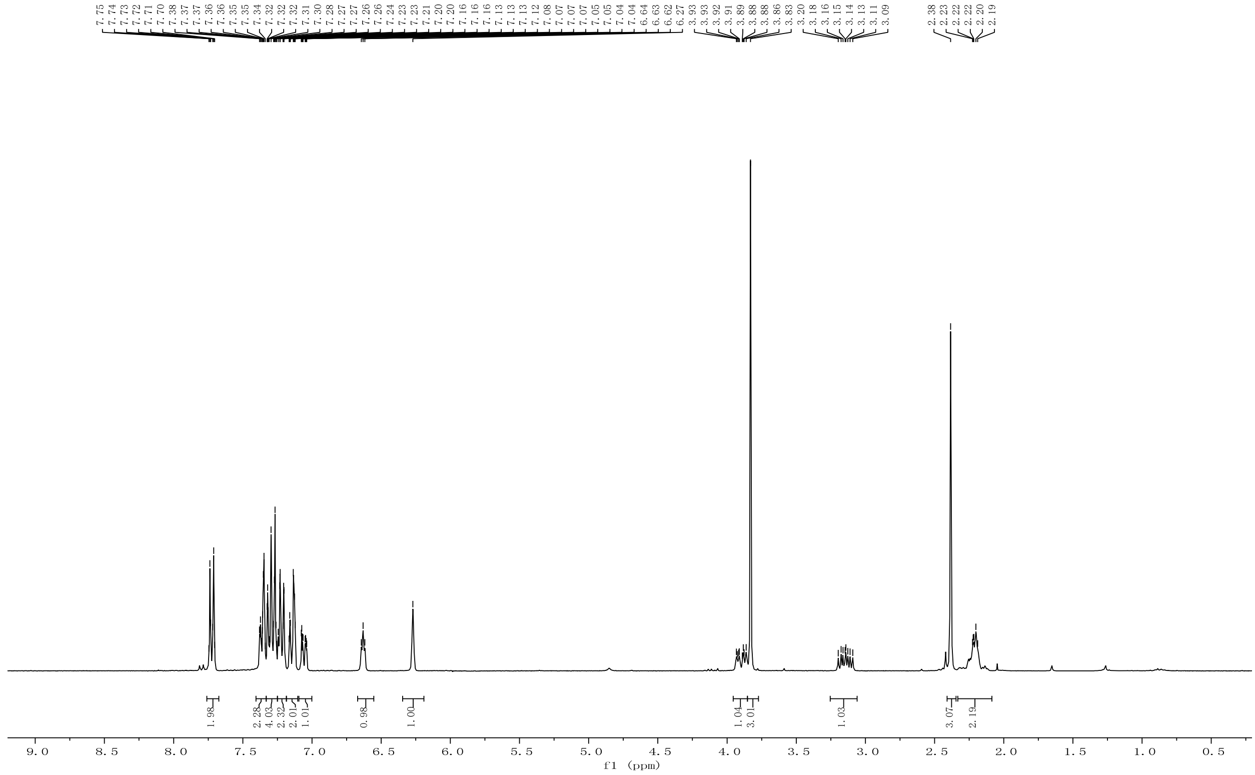
7.75 7.74 7.73 7.72 7.71 7.70 7.38 7.37 7.37 7.36 7.36 7.35 7.35 7.34 7.32 7.32 7.32 7.32 7.31 7.30 7.28 7.27 7.27 7.26 7.26 7.24 7.23 7.23 7.21 7.20 7.20 7.16 7.16 7.13 7.13 7.13 7.12 7.08 7.07 7.07 7.07 7.05 7.05 7.04 7.04 6.64 6.63 6.62 6.27 3.93 3.93 3.92 3.91 3.89 3.88 3.88 3.86 3.83 3.20 3.18 3.16 3.15 3.14 3.13 3.11 3.09 2.38 2.23 2.22 2.22 2.20 2.19
1.98 2.28 4.03 2.32 2.01 1.01 0.98 1.00 1.04 3.01 1.03 3.07 2.19
9.0 8.5 8.0 7.5 7.0 6.5 6.0 5.5 5.0 4.5 4.0 3.5 3.0 2.5 2.0 1.5 1.0 0.5
f1 (ppm)

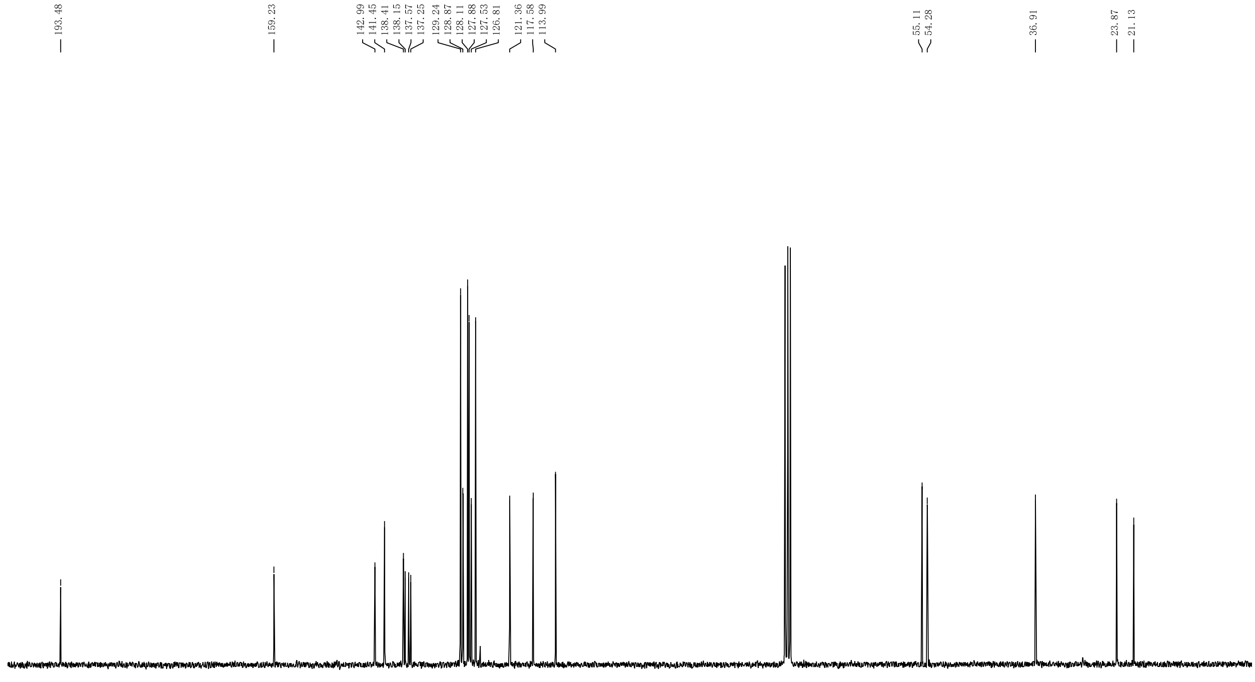
193.48
159.23
142.99 141.45 138.41 138.15 137.57 137.25 129.24 128.87 128.11 127.88 127.53 126.81 121.36 117.58 113.99
55.11 54.28
36.91
23.87 21.13
200 190 180 170 160 150 140 130 120 110 100 90 80 70 60 50 40 30 20 10
f1 (ppm)

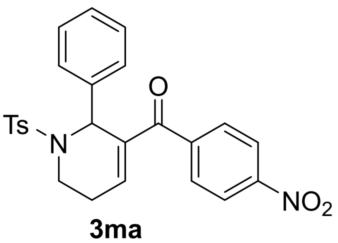
Ts
N
O
NO2

**3ma**

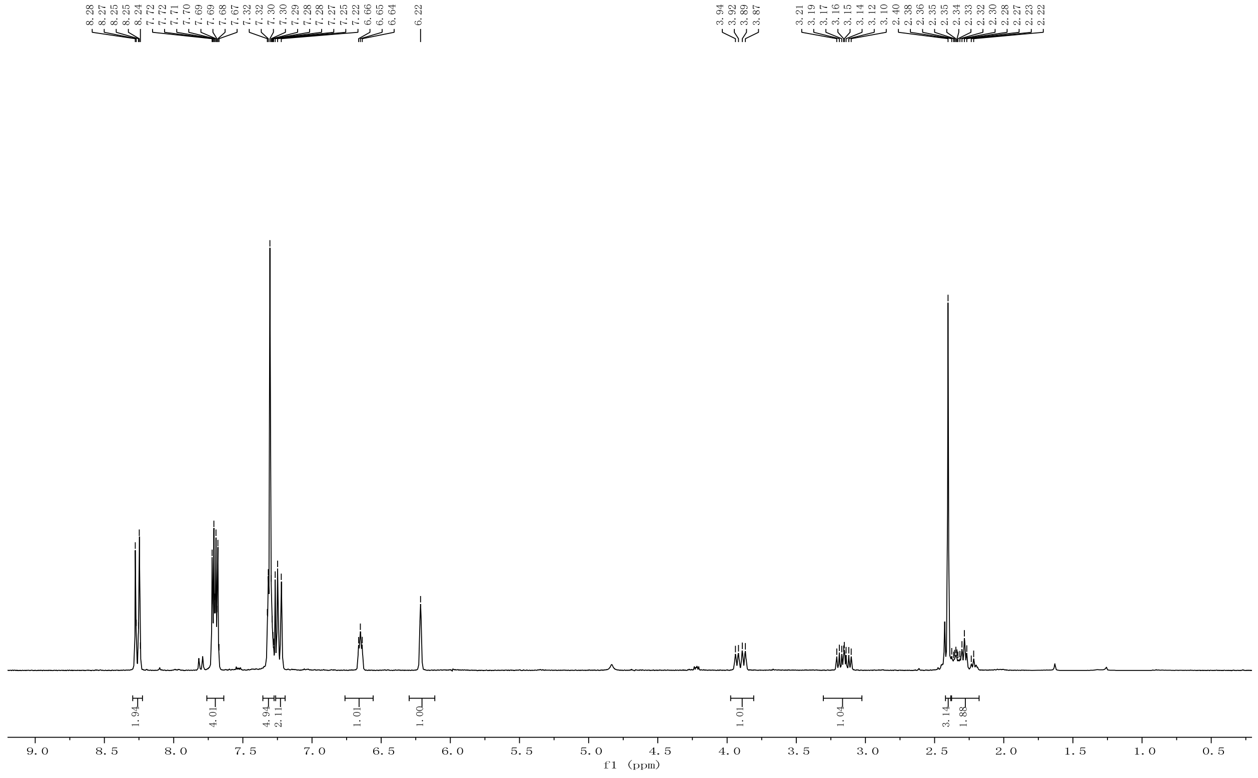
8.28
8.27
8.25
8.25
8.24
7.72
7.72
7.71
7.70
7.69
7.69
7.68
7.67
7.32
7.32
7.30
7.30
7.29
7.28
7.28
7.27
7.25
7.22
6.66
6.65
6.64
6.22
3.94
3.92
3.89
3.87
3.21
3.19
3.17
3.16
3.15
3.14
3.12
3.10
2.40
2.38
2.36
2.35
2.34
2.33
2.32
2.30
2.28
2.27
2.23
2.22
1.94
4.01
4.94
2.11
1.01
1.00
1.01
1.04
3.14
1.88
9.0
8.5
8.0
7.5
7.0
6.5
6.0
5.5
5.0
4.5
4.0
3.5
3.0
2.5
2.0
1.5
1.0
0.5
f1 (ppm)

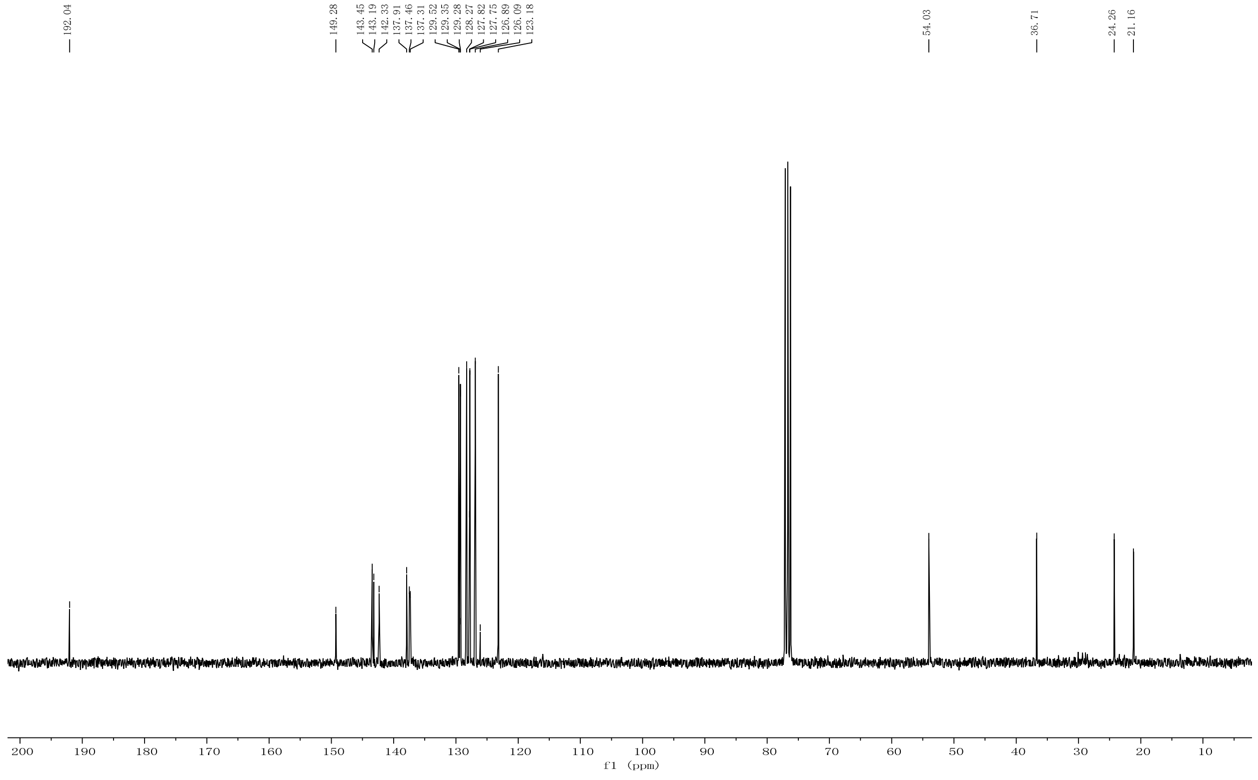
192.04
149.28
143.45
143.19
142.33
137.91
137.46
137.31
129.52
129.35
129.28
128.27
127.82
127.75
126.89
126.09
123.18
54.03
36.71
24.26
21.16
200
190
180
170
160
150
140
130
120
110
100
90
80
70
60
50
40
30
20
10
f1 (ppm)

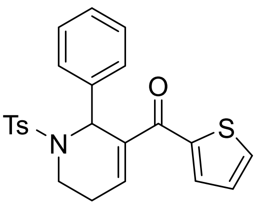
O
S
Ts
N

**3na**

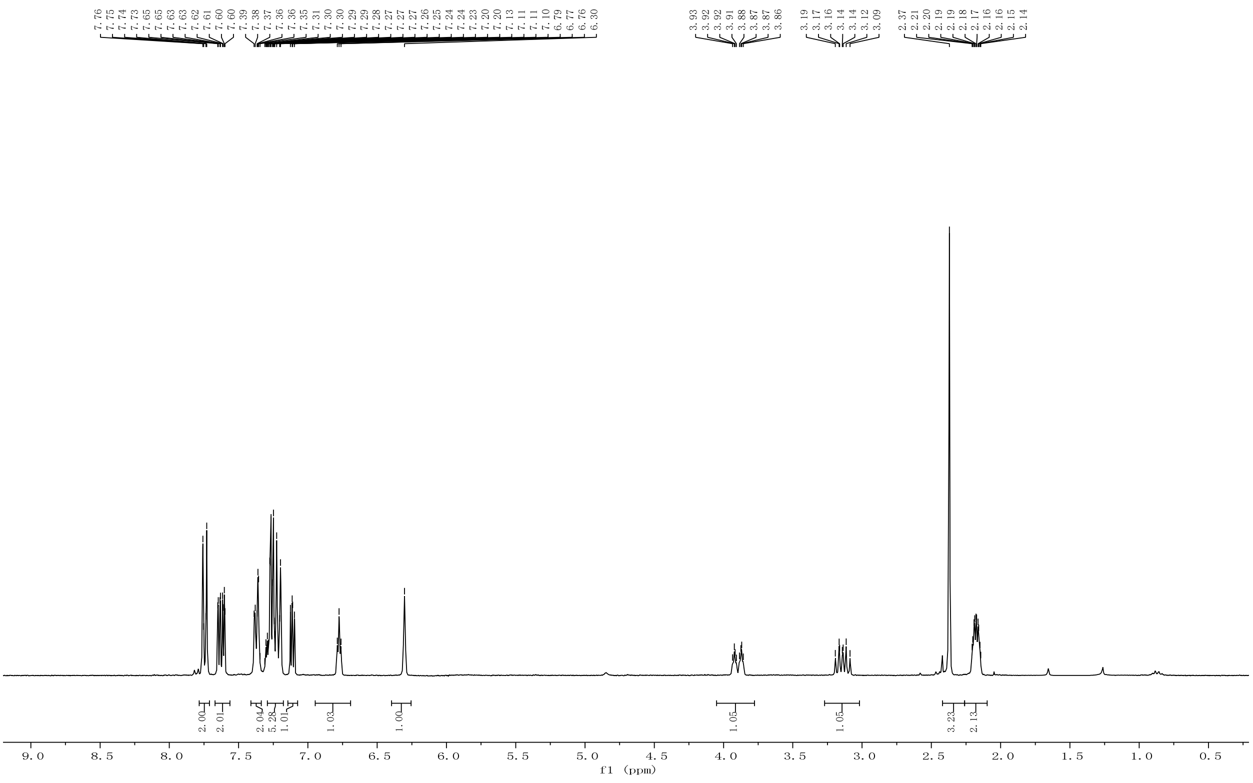
7.76 7.75 7.74 7.73 7.65 7.65 7.63 7.63 7.62 7.61 7.60 7.60 7.39 7.38 7.37 7.36 7.36 7.35 7.31 7.30 7.30 7.29 7.29 7.28 7.27 7.27 7.27 7.26 7.25 7.24 7.24 7.23 7.20 7.20 7.13 7.11 7.11 7.10 6.79 6.77 6.76 6.30
3.93 3.92 3.92 3.91 3.88 3.87 3.87 3.86
3.19 3.17 3.16 3.14 3.14 3.12 3.09
2.37 2.21 2.20 2.19 2.19 2.18 2.17 2.16 2.15 2.14
2.00 2.01 2.04 5.28 1.01 1.03 1.00 1.05 1.05 3.23 2.13
9.0 8.5 8.0 7.5 7.0 6.5 6.0 5.5 5.0 4.5 4.0 3.5 3.0 2.5 2.0 1.5 1.0 0.5
f1 (ppm)

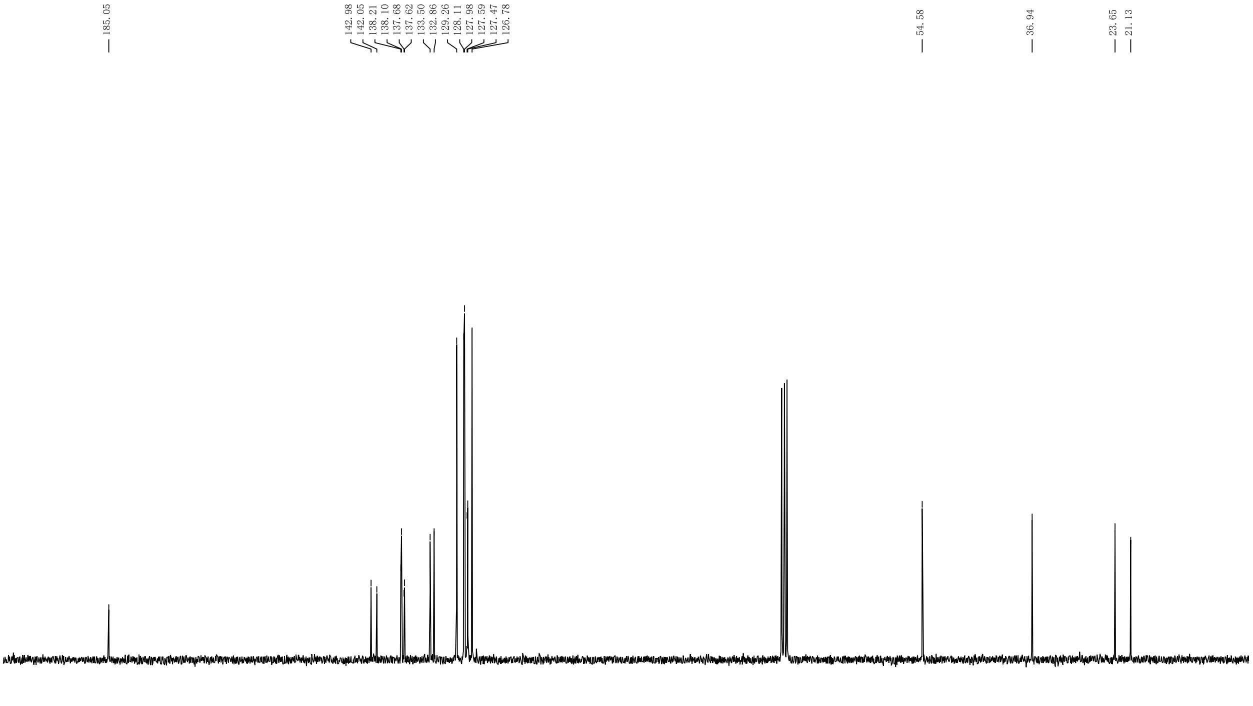
185.05
142.98 142.05 138.21 138.10 137.68 137.62 133.50 132.86 129.26 128.11 127.98 127.59 127.47 126.78
54.58
36.94
23.65 21.13
200 190 180 170 160 150 140 130 120 110 100 90 80 70 60 50 40 30 20 10
f1 (ppm)

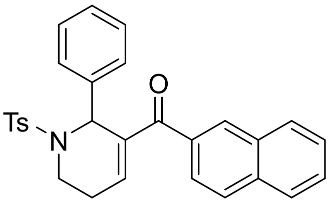
Ts
N
O

**3oa**

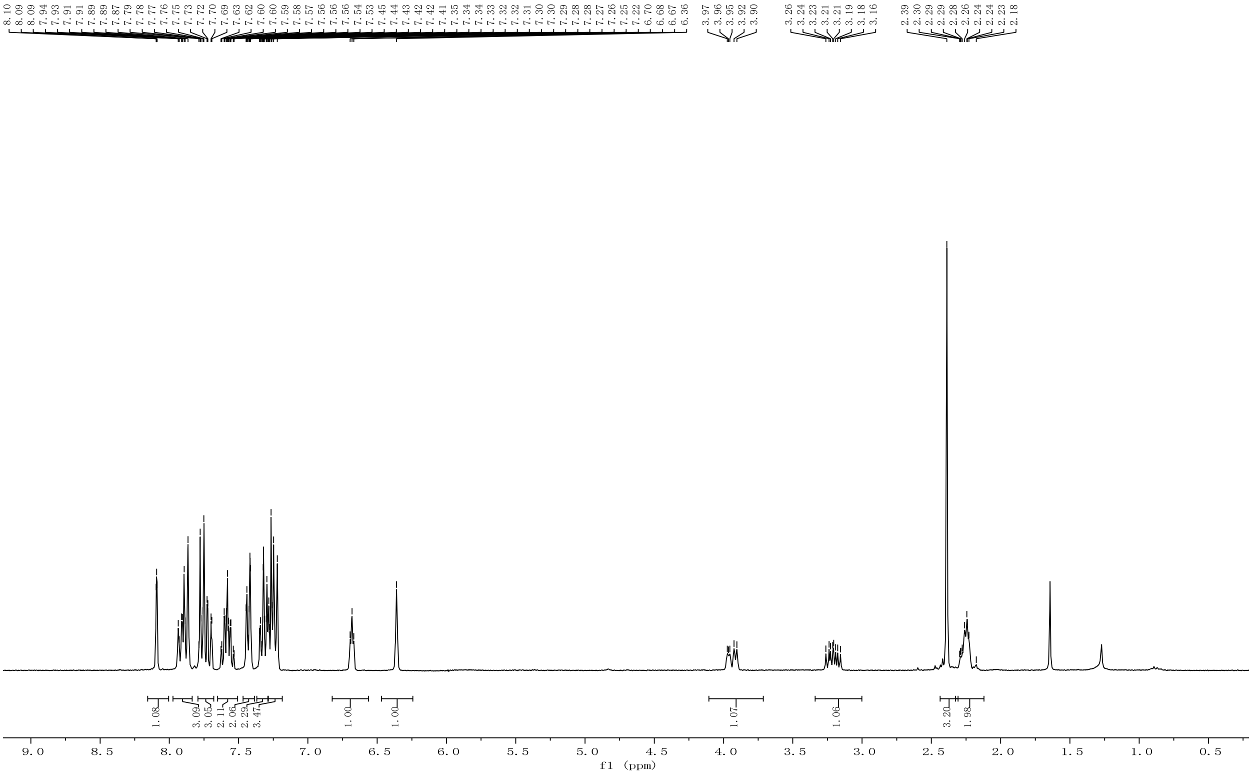
8.10 8.09 8.09 7.94 7.93 7.91 7.91 7.89 7.89 7.87 7.79 7.78 7.77 7.76 7.75 7.73 7.72 7.70 7.69 7.63 7.62 7.60 7.60 7.59 7.58 7.57 7.56 7.56 7.56 7.54 7.53 7.45 7.44 7.43 7.42 7.42 7.41 7.35 7.34 7.34 7.33 7.32 7.32 7.31 7.30 7.30 7.29 7.28 7.28 7.27 7.26 7.25 7.22 6.70 6.68 6.67 6.36
3.97 3.96 3.95 3.92 3.90
3.26 3.24 3.23 3.21 3.21 3.19 3.18 3.16
2.39 2.30 2.29 2.29 2.28 2.26 2.24 2.24 2.23 2.18
1.08 3.09 3.05 2.11 2.06 2.29 3.47 1.00 1.00 1.07 1.06 3.20 1.98
9.0 8.5 8.0 7.5 7.0 6.5 6.0 5.5 5.0 4.5 4.0 3.5 3.0 2.5 2.0 1.5 1.0 0.5
f1 (ppm)

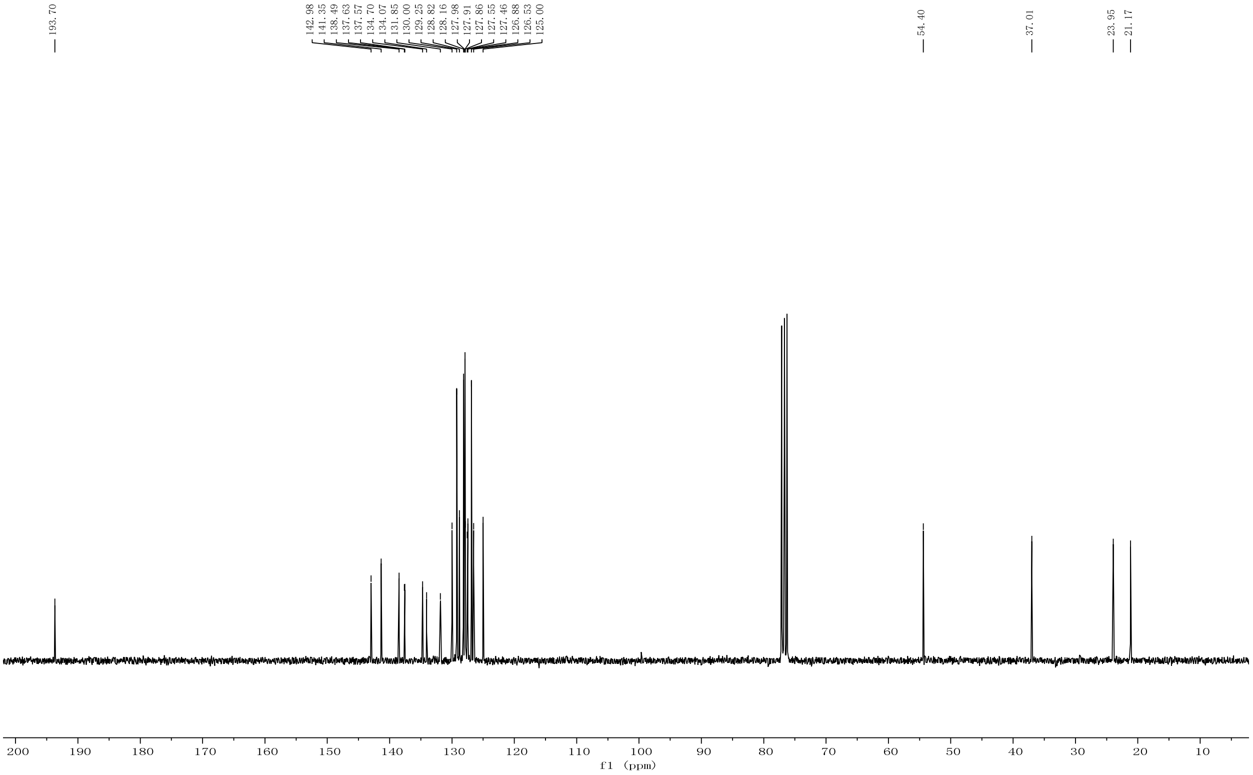
193.70
142.98 141.35 138.49 137.63 137.57 134.70 134.07 131.85 130.00 129.25 128.82 128.16 127.98 127.91 127.86 127.55 127.46 126.88 126.53 125.00
54.40
37.01
23.95
21.17
200 190 180 170 160 150 140 130 120 110 100 90 80 70 60 50 40 30 20 10
f1 (ppm)

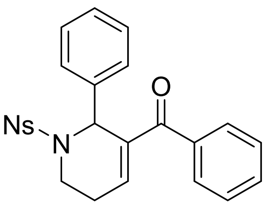
O
Ns
N

**3pa**

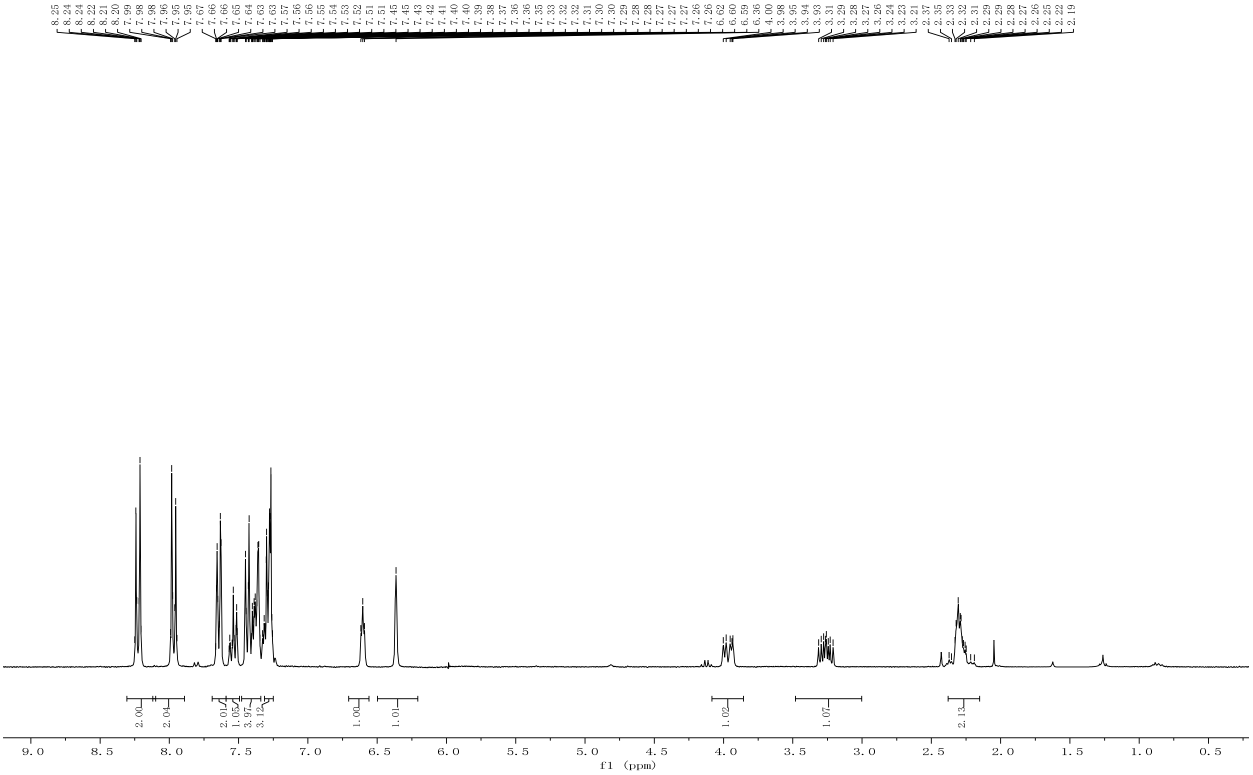
8.25 8.24 8.24 8.22 8.21 8.20 7.99 7.98 7.98 7.96 7.95 7.95 7.67 7.66 7.66 7.65 7.64 7.63 7.63 7.57 7.57 7.56 7.56 7.55 7.54 7.53 7.52 7.51 7.51 7.45 7.45 7.43 7.42 7.41 7.40 7.40 7.39 7.38 7.37 7.36 7.36 7.35 7.33 7.32 7.32 7.31 7.30 7.29 7.28 7.28 7.27 7.27 7.27 7.26 7.26 6.62 6.60 6.59 6.36 4.00 3.98 3.95 3.94 3.93 3.31 3.29 3.28 3.27 3.26 3.24 3.23 3.21 2.37 2.35 2.33 2.32 2.31 2.29 2.29 2.28 2.27 2.26 2.25 2.22 2.19
2.00
2.04
2.01
1.05
3.97
3.12
1.00
1.01
1.02
1.07
2.13
9.0 8.5 8.0 7.5 7.0 6.5 6.0 5.5 5.0 4.5 4.0 3.5 3.0 2.5 2.0 1.5 1.0 0.5
f1 (ppm)

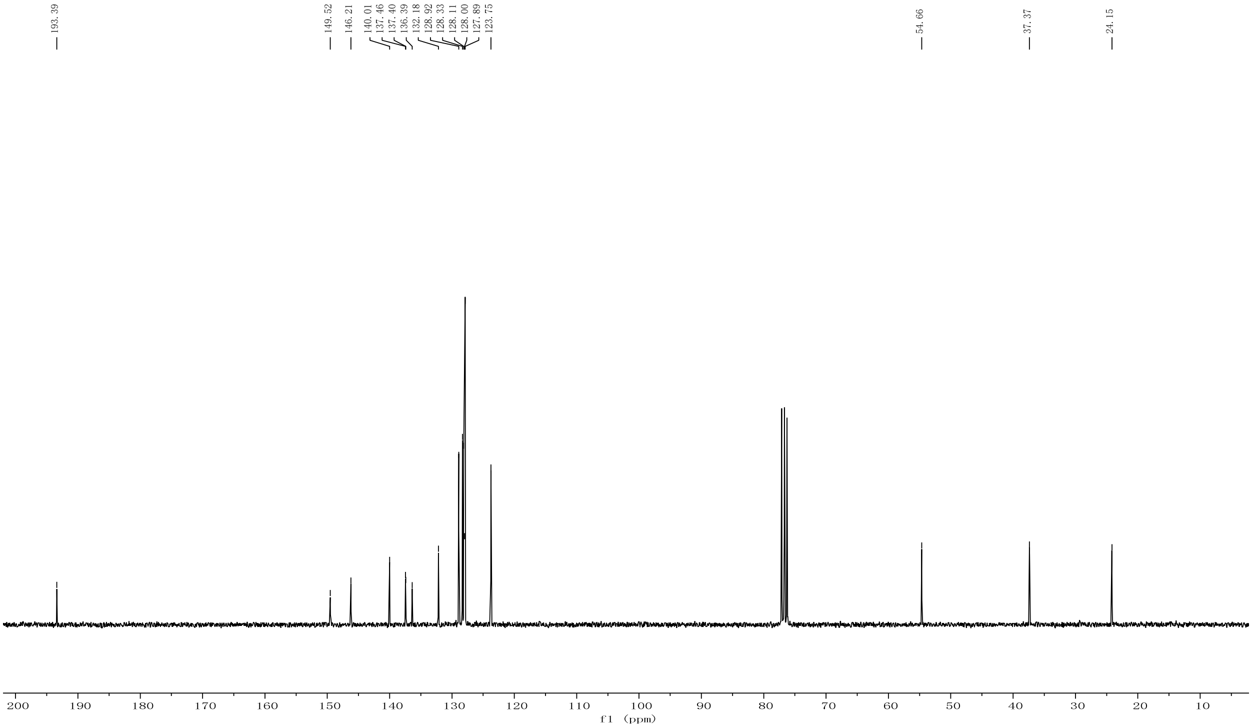
193.39
149.52
146.21
140.01 137.46 137.40 136.39 132.18 128.92 128.33 128.11 128.00 127.89 123.75
54.66
37.37
24.15
200 190 180 170 160 150 140 130 120 110 100 90 80 70 60 50 40 30 20 10
f1 (ppm)

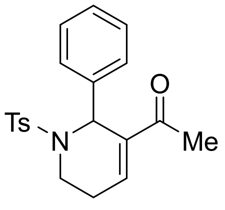
Ts
N
O
Me

**3sa**

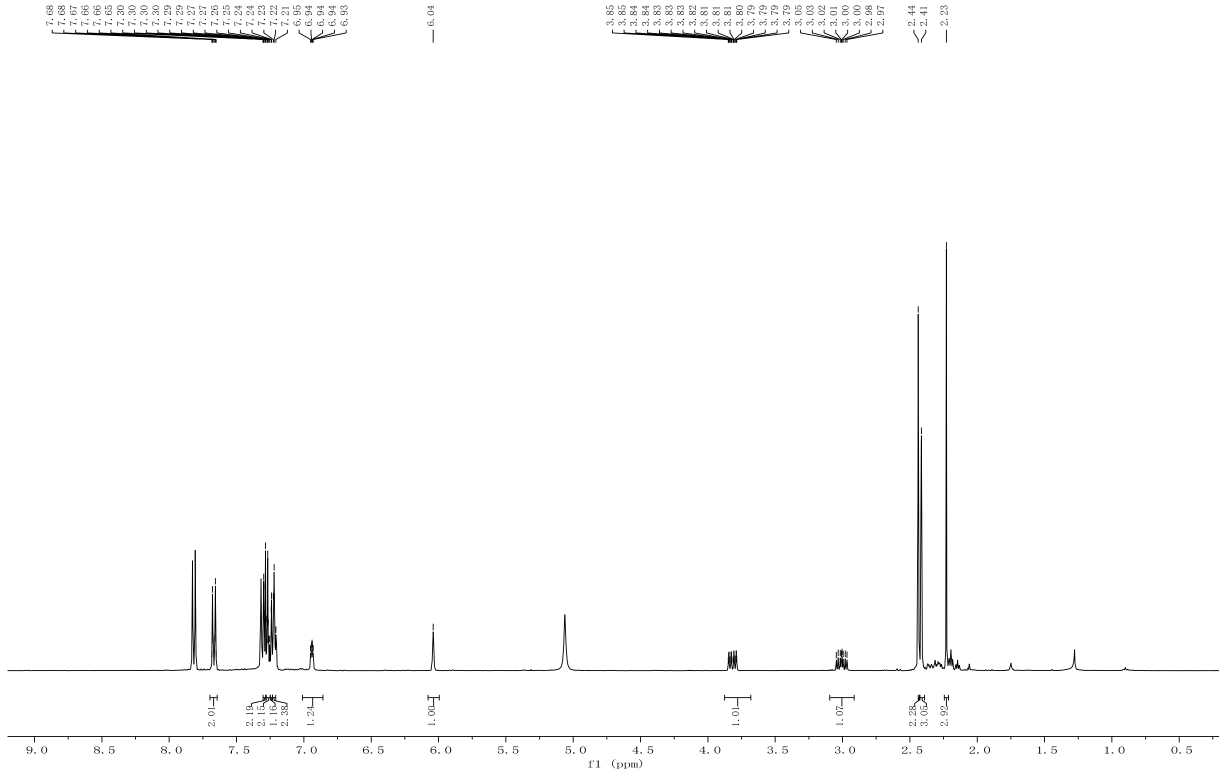
7.68 7.68 7.67 7.66 7.66 7.65 7.30 7.30 7.30 7.30 7.29 7.29 7.27 7.27 7.26 7.25 7.24 7.24 7.23 7.22 7.21 6.95 6.94 6.94 6.94 6.93
6.04
3.85 3.85 3.84 3.83 3.83 3.82 3.81 3.81 3.81 3.80 3.79 3.79 3.79 3.79 3.05 3.03 3.02 3.01 3.00 3.00 2.98 2.97
2.44 2.41
2.23
2.01 2.19 2.15 1.16 2.38 1.24 1.00 1.01 1.07 2.28 3.05 2.92
9.0 8.5 8.0 7.5 7.0 6.5 6.0 5.5 5.0 4.5 4.0 3.5 3.0 2.5 2.0 1.5 1.0 0.5
f1 (ppm)

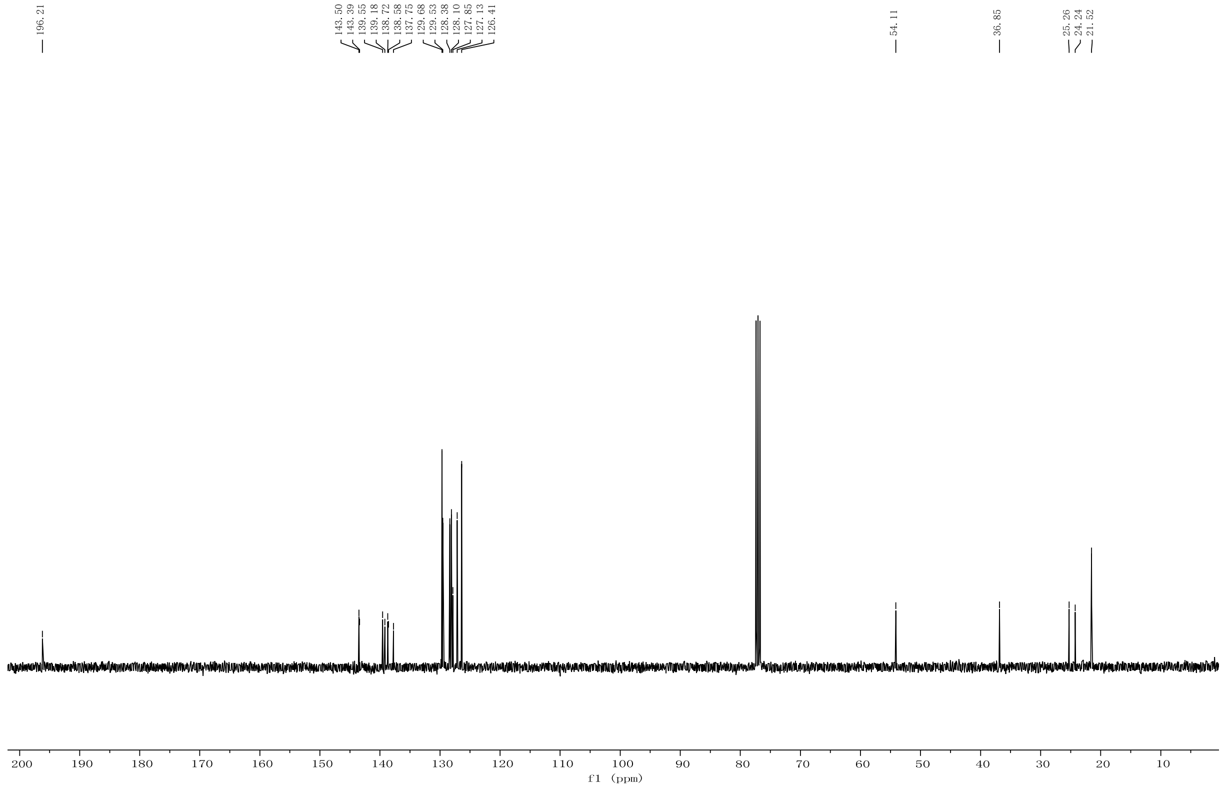
196.21
143.50 143.39 139.55 139.18 138.72 138.58 137.75 129.68 129.53 128.38 128.10 127.85 127.13 126.41
54.11
36.85
25.26 24.24 21.52
200 190 180 170 160 150 140 130 120 110 100 90 80 70 60 50 40 30 20 10
f1 (ppm)

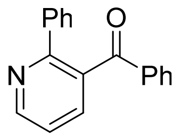

Ph
O
N
Ph


**4**

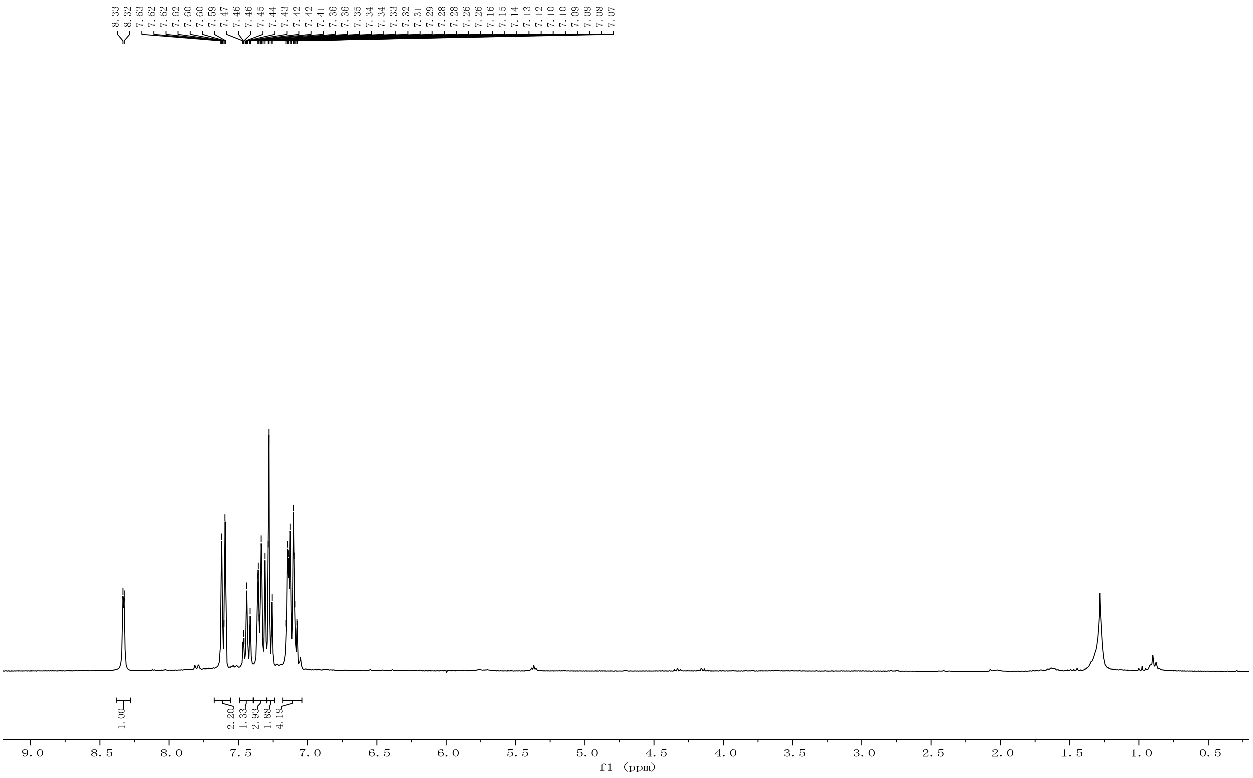

8.33
8.32
7.63
7.62
7.62
7.62
7.60
7.60
7.59
7.47
7.46
7.46
7.45
7.44
7.43
7.42
7.41
7.36
7.36
7.35
7.34
7.34
7.33
7.32
7.31
7.29
7.28
7.28
7.26
7.26
7.16
7.15
7.14
7.13
7.12
7.10
7.10
7.09
7.08
7.07
1.00
2.20
1.33
2.93
1.88
4.19
9.0
8.5
8.0
7.5
7.0
6.5
6.0
5.5
5.0
4.5
4.0
3.5
3.0
2.5
2.0
1.5
1.0
0.5
f1 (ppm)


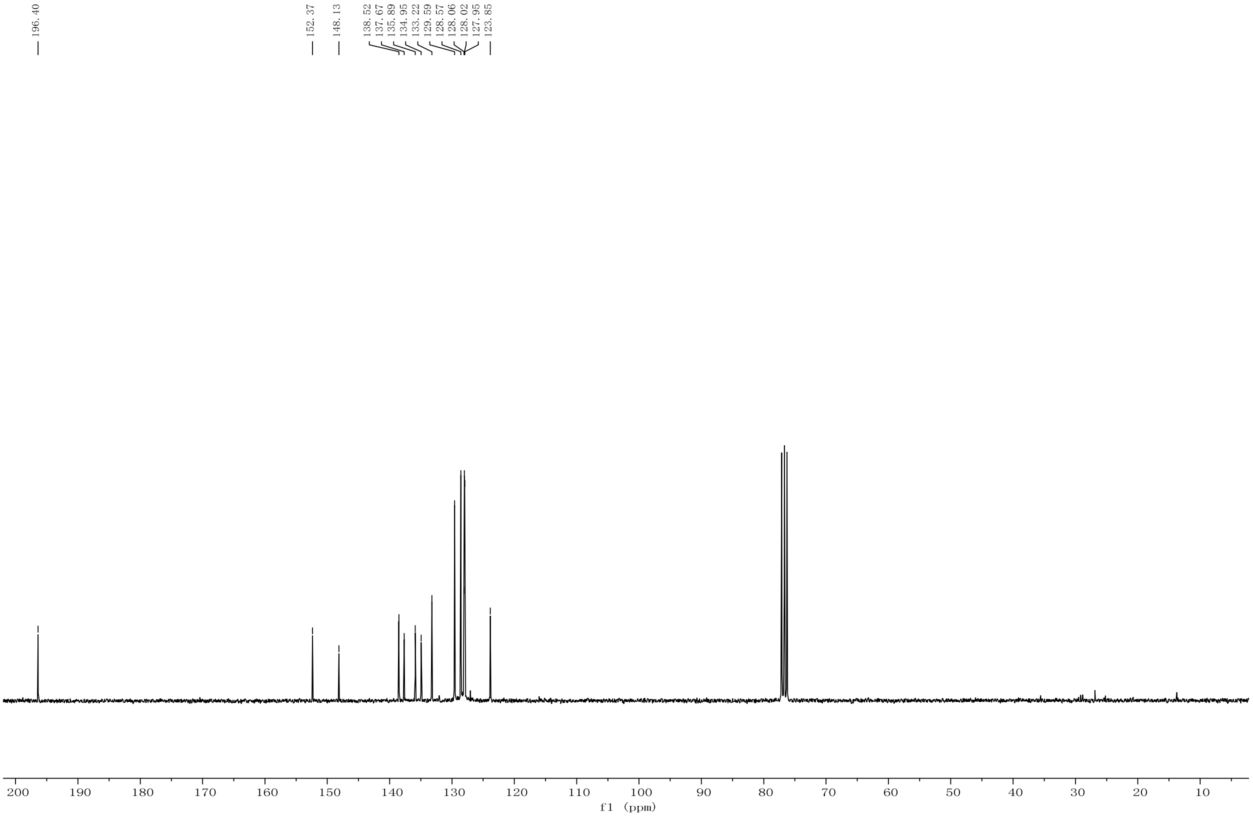

196.40
152.37
148.13
138.52
137.67
135.89
134.95
133.22
129.59
128.57
128.06
128.02
127.95
123.85
200
190
180
170
160
150
140
130
120
110
100
90
80
70
60
50
40
30
20
10
f1 (ppm)

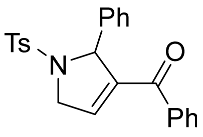


**6**

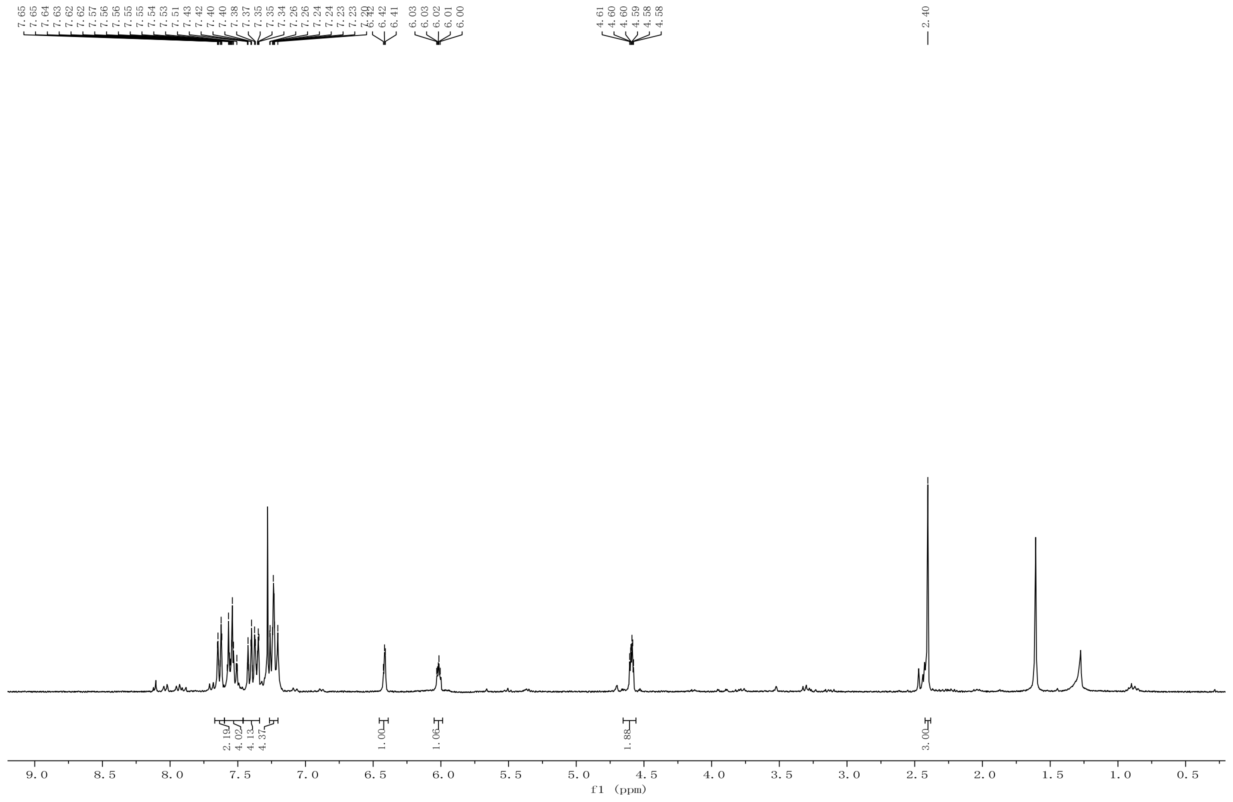

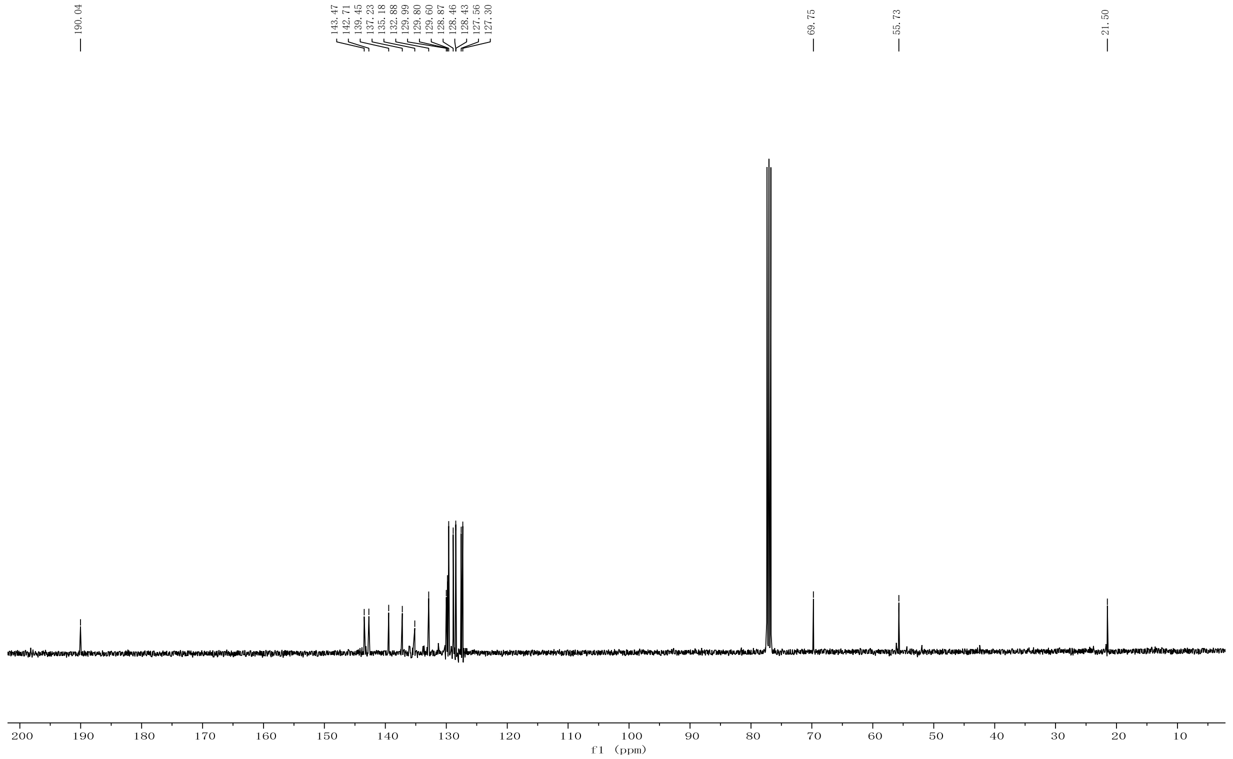

## HPLC chromatogram of racemic 3aa

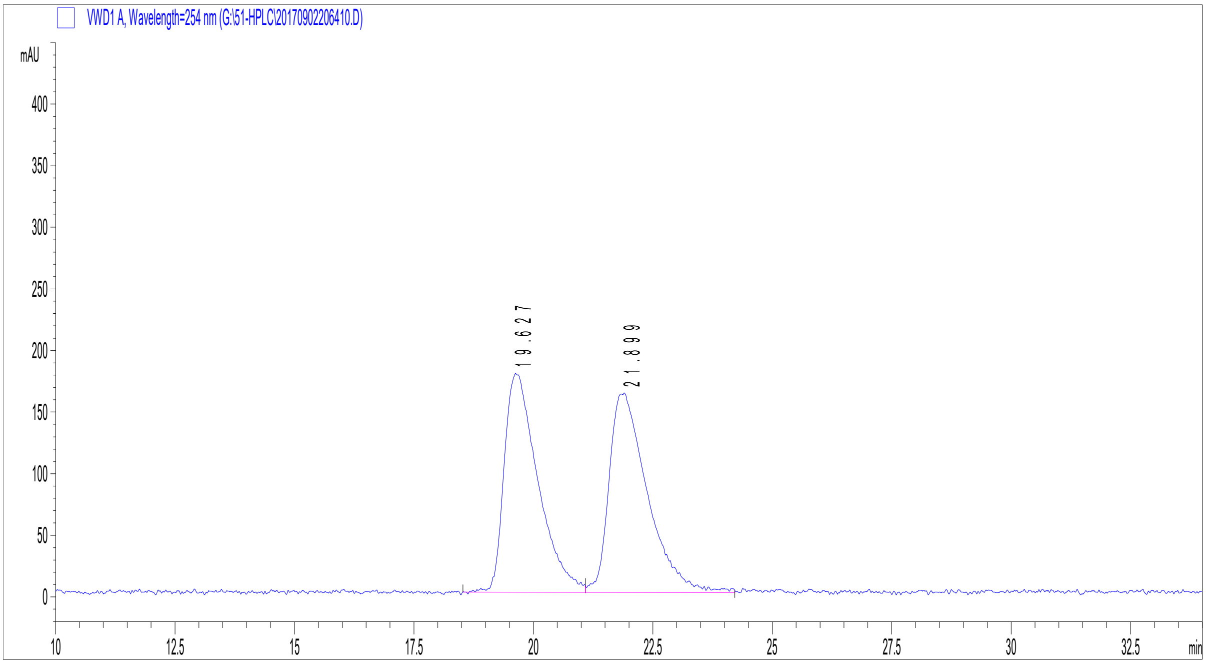


| Peak # | RetTime [min] | Type | Width [min] | Area mAU *s | Height [mAU] | Area % |
|---|---|---|---|---|---|---|
| 1 | 19.627 | MF | 0.8182 | 8717.53906 | 177.56789 | 49.0440 |
| 2 | 21.899 | FM | 0.9324 | 9057.38965 | 161.89520 | 50.9560 |

## HPLC chromatogram of chiral 3aa

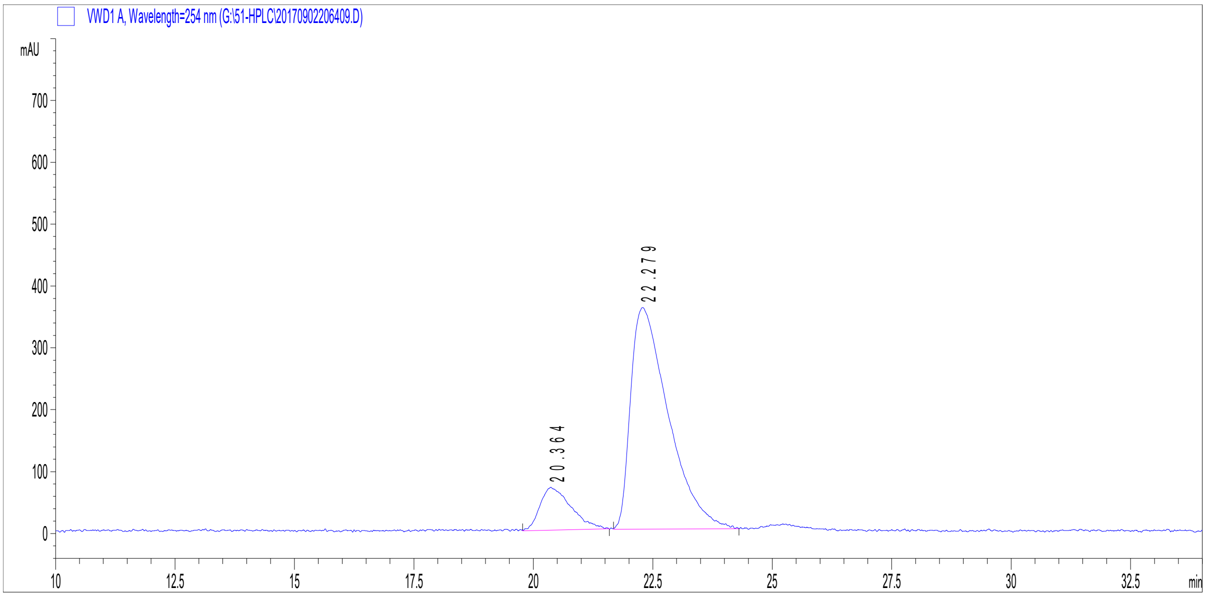


| Peak # | RetTime [min] | Type | Width [min] | Area mAU *s | Height [mAU] | Area % |
|---|---|---|---|---|---|---|
| 1 | 20.364 | MM | 0.7506 | 3121.38257 | 69.31057 | 13.6582 |
| 2 | 22.279 | MM | 0.9181 | 1.97321e4 | 358.21002 | 86.3418 |

## X-Ray Crystallography Data

Crystallographic data for **3aq** has been deposited with the Cambridge Crystallographic Data Centre as deposition number CCDC 1575011. These data can be obtained free of charge via www.ccdc.cam.ac.uk/data_request/cif, or by emailing data_request@ccdc.cam.ac.uk, or by contacting The Cambridge Crystallographic Data Centre, 12, Union Road, Cambridge CB2 1EZ, UK; fax: +44 1223 336033.

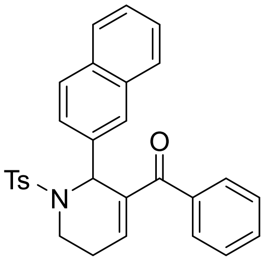


**3aq**

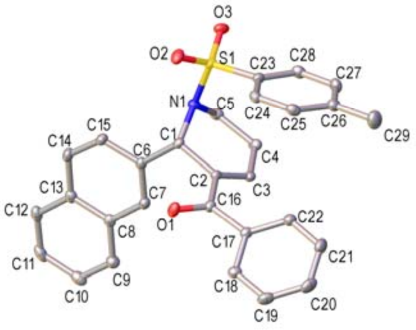


**Table S1**. Crystal data and structure refinement for **3aq**.

Identification code: **3aq**

Empirical formula, $C_{29}H_{25}NO_3S$

Formula weight, 467.56

Temperature, 173.1500 K

Wavelength, 0.71073 Å

Crystal system, Monoclinic

Space group, P 1 21 1

| | | |
|---|---|---|
| Unit cell dimensions | a = 9.769(3) Å | a= 90°. |
| | b = 13.123(3) Å | b= 114.345(3)°. |
| | c = 10.039(3) Å | g = 90°. |
| Volume | 1172.6(5) Å3 | |
| Z | 2 | |
| Density (calculated) | 1.324 Mg/m3 | |
| Absorption coefficient | 0.170 mm-1 | |
| F(000) | 492 | |
| Crystal size | 0.406 x 0.315 x 0.166 mm3 | |
| Theta range for data collection | 2.899 to 27.482°. | |
| Index ranges | -12<=h<=12, -17<=k<=16, -10<=l<=13 | |
| Reflections collected | 7830 | |
| Independent reflections | 4984 [R(int) = 0.0263] | |
| Completeness to theta = 26.000° | 99.3 % | |

| | |
|---|---|
| Absorption correction | Semi-empirical from equivalents |
| Max. and min. transmission | 1.00000 and 0.88525 |
| Refinement method | Full-matrix least-squares on F2 |
| Data / restraints / parameters | 4984 / 1 / 308 |
| Goodness-of-fit on F2 | 1.096 |
| Final R indices [I>2sigma(I)] | R1 = 0.0413, wR2 = 0.0895 |
| R indices (all data) | R1 = 0.0433, wR2 = 0.0909 |
| Absolute structure parameter | -0.05(4) |
| Extinction coefficient | n/a |
| Largest diff. peak and hole | 0.190 and -0.220 e.Å-3 |

**Table S2**. Atomic coordinates (x 104) and equivalent isotropic displacement parameters (Å$^2$ x 103) for **3aq**. U(eq) is defined as one third of the trace of the orthogonalized Uij tensor.

| | x | y | z | U(eq) |
|---|---|---|---|---|
| S1 | 6054(1) | 2631(1) | 6739(1) | 26(1) |
| O1 | 8272(2) | 4804(2) | 4139(2) | 35(1) |
| O2 | 7061(3) | 1983(2) | 6426(2) | 35(1) |
| O3 | 4939(3) | 2204(2) | 7158(2) | 35(1) |
| N1 | 5133(3) | 3283(2) | 5230(2) | 23(1) |
| C1 | 6019(3) | 3768(2) | 4515(3) | 22(1) |
| C2 | 6447(3) | 4850(2) | 5076(3) | 21(1) |
| C3 | 5577(3) | 5392(2) | 5548(3) | 24(1) |
| C4 | 4213(3) | 4975(2) | 5681(3) | 28(1) |
| C5 | 3811(3) | 3889(2) | 5095(3) | 28(1) |
| C6 | 5118(3) | 3722(2) | 2863(3) | 24(1) |
| C7 | 4580(3) | 4580(2) | 2037(3) | 25(1) |
| C8 | 3714(3) | 4525(2) | 503(3) | 26(1) |
| C9 | 3193(3) | 5412(3) | -356(3) | 31(1) |
| C10 | 2334(3) | 5337(3) | -1837(3) | 35(1) |
| C11 | 1965(3) | 4381(3) | -2509(3) | 37(1) |
| C12 | 2473(4) | 3507(3) | -1709(3) | 35(1) |
| C13 | 3372(3) | 3552(2) | -177(3) | 28(1) |
| C14 | 3964(3) | 2670(3) | 704(3) | 33(1) |
| C15 | 4814(3) | 2757(2) | 2163(3) | 30(1) |
| C16 | 7786(3) | 5264(2) | 4921(3) | 24(1) |
| C17 | 8570(3) | 6213(2) | 5672(3) | 24(1) |

| | | | | |
|---|---|---|---|---|
| C18 | 9327(3) | 6761(2) | 4995(3) | 28(1) |
| C19 | 10144(3) | 7619(3) | 5650(4) | 38(1) |
| C20 | 10224(4) | 7937(2) | 6987(4) | 42(1) |
| C21 | 9501(4) | 7402(2) | 7676(4) | 40(1) |
| C22 | 8667(3) | 6536(2) | 7032(3) | 31(1) |
| C23 | 7172(3) | 3477(2) | 8138(3) | 25(1) |
| C24 | 8635(3) | 3684(2) | 8317(3) | 28(1) |
| C25 | 9476(3) | 4383(2) | 9368(3) | 30(1) |
| C26 | 8890(3) | 4877(2) | 10237(3) | 31(1) |
| C27 | 7436(4) | 4641(2) | 10052(3) | 33(1) |
| C28 | 6575(3) | 3945(2) | 9014(3) | 30(1) |
| C29 | 9826(5) | 5639(3) | 11377(4) | 52(1) |

**Table S3**. Bond lengths [Å] and angles [°] for **3aq**.

| | |
|---|---|
| S1-O2 | 1.430(2) |
| S1-O3 | 1.434(2) |
| S1-N1 | 1.647(2) |
| S1-C23 | 1.770(3) |
| O1-C16 | 1.230(3) |
| N1-C1 | 1.477(3) |
| N1-C5 | 1.475(4) |
| C1-H1 | 0.9800 |
| C1-C2 | 1.521(4) |
| C1-C6 | 1.525(4) |
| C2-C3 | 1.336(4) |
| C2-C16 | 1.483(4) |
| C3-H3 | 0.9300 |
| C3-C4 | 1.496(4) |
| C4-H4A | 0.9700 |
| C4-H4B | 0.9700 |
| C4-C5 | 1.530(4) |
| C5-H5A | 0.9700 |
| C5-H5B | 0.9700 |
| C6-C7 | 1.367(4) |
| C6-C15 | 1.419(4) |
| C7-H7 | 0.9300 |

| | |
|---|---|
| C7-C8 | 1.421(4) |
| C8-C9 | 1.413(4) |
| C8-C13 | 1.420(4) |
| C9-H9 | 0.9300 |
| C9-C10 | 1.377(4) |
| C10-H10 | 0.9300 |
| C10-C11 | 1.400(5) |
| C11-H11 | 0.9300 |
| C11-C12 | 1.370(5) |
| C12-H12 | 0.9300 |
| C12-C13 | 1.424(4) |
| C13-C14 | 1.426(5) |
| C14-H14 | 0.9300 |
| C14-C15 | 1.358(4) |
| C15-H15 | 0.9300 |
| C16-C17 | 1.493(4) |
| C17-C18 | 1.393(4) |
| C17-C22 | 1.395(4) |
| C18-H18 | 0.9300 |
| C18-C19 | 1.380(4) |
| C19-H19 | 0.9300 |
| C19-C20 | 1.376(5) |
| C20-H20 | 0.9300 |
| C20-C21 | 1.369(5) |
| C21-H21 | 0.9300 |
| C21-C22 | 1.392(4) |
| C22-H22 | 0.9300 |
| C23-C24 | 1.391(4) |
| C23-C28 | 1.384(4) |
| C24-H24 | 0.9300 |
| C24-C25 | 1.384(4) |
| C25-H25 | 0.9300 |
| C25-C26 | 1.386(4) |
| C26-C27 | 1.390(4) |
| C26-C29 | 1.511(4) |
| C27-H27 | 0.9300 |
| C27-C28 | 1.380(4) |
| C28-H28 | 0.9300 |

| | |
|---|---|
| C29-H29A | 0.9600 |
| C29-H29B | 0.9600 |
| C29-H29C | 0.9600 |

| | |
|---|---|
| O2-S1-O3 | 120.49(14) |
| O2-S1-N1 | 105.75(13) |
| O2-S1-C23 | 106.97(14) |
| O3-S1-N1 | 106.25(13) |
| O3-S1-C23 | 107.86(14) |
| N1-S1-C23 | 109.18(13) |
| C1-N1-S1 | 117.64(18) |
| C5-N1-S1 | 119.4(2) |
| C5-N1-C1 | 112.9(2) |
| N1-C1-H1 | 108.3 |
| N1-C1-C2 | 110.4(2) |
| N1-C1-C6 | 108.7(2) |
| C2-C1-H1 | 108.3 |
| C2-C1-C6 | 112.8(2) |
| C6-C1-H1 | 108.3 |
| C3-C2-C1 | 120.6(2) |
| C3-C2-C16 | 124.1(2) |
| C16-C2-C1 | 115.1(2) |
| C2-C3-H3 | 118.1 |
| C2-C3-C4 | 123.9(2) |
| C4-C3-H3 | 118.1 |
| C3-C4-H4A | 108.8 |
| C3-C4-H4B | 108.8 |
| C3-C4-C5 | 113.7(2) |
| H4A-C4-H4B | 107.7 |
| C5-C4-H4A | 108.8 |
| C5-C4-H4B | 108.8 |
| N1-C5-C4 | 113.0(2) |
| N1-C5-H5A | 109.0 |
| N1-C5-H5B | 109.0 |
| C4-C5-H5A | 109.0 |
| C4-C5-H5B | 109.0 |
| H5A-C5-H5B | 107.8 |
| C7-C6-C1 | 122.1(2) |

| | |
|---|---|
| C7-C6-C15 | 119.0(2) |
| C15-C6-C1 | 118.8(2) |
| C6-C7-H7 | 119.2 |
| C6-C7-C8 | 121.6(3) |
| C8-C7-H7 | 119.2 |
| C9-C8-C7 | 121.5(3) |
| C9-C8-C13 | 119.6(3) |
| C13-C8-C7 | 118.9(3) |
| C8-C9-H9 | 119.8 |
| C10-C9-C8 | 120.3(3) |
| C10-C9-H9 | 119.8 |
| C9-C10-H10 | 119.8 |
| C9-C10-C11 | 120.4(3) |
| C11-C10-H10 | 119.8 |
| C10-C11-H11 | 119.7 |
| C12-C11-C10 | 120.6(3) |
| C12-C11-H11 | 119.7 |
| C11-C12-H12 | 119.6 |
| C11-C12-C13 | 120.8(3) |
| C13-C12-H12 | 119.6 |
| C8-C13-C12 | 118.3(3) |
| C8-C13-C14 | 118.4(2) |
| C12-C13-C14 | 123.3(3) |
| C13-C14-H14 | 119.6 |
| C15-C14-C13 | 120.8(3) |
| C15-C14-H14 | 119.6 |
| C6-C15-H15 | 119.4 |
| C14-C15-C6 | 121.2(3) |
| C14-C15-H15 | 119.4 |
| O1-C16-C2 | 118.4(2) |
| O1-C16-C17 | 118.7(3) |
| C2-C16-C17 | 122.9(2) |
| C18-C17-C16 | 116.7(3) |
| C18-C17-C22 | 119.0(3) |
| C22-C17-C16 | 124.2(3) |
| C17-C18-H18 | 119.7 |
| C19-C18-C17 | 120.5(3) |
| C19-C18-H18 | 119.7 |

| | |
|---|---|
| C18-C19-H19 | 120.0 |
| C20-C19-C18 | 120.1(3) |
| C20-C19-H19 | 120.0 |
| C19-C20-H20 | 119.9 |
| C21-C20-C19 | 120.2(3) |
| C21-C20-H20 | 119.9 |
| C20-C21-H21 | 119.7 |
| C20-C21-C22 | 120.6(3) |
| C22-C21-H21 | 119.7 |
| C17-C22-H22 | 120.2 |
| C21-C22-C17 | 119.6(3) |
| C21-C22-H22 | 120.2 |
| C24-C23-S1 | 119.4(2) |
| C28-C23-S1 | 119.9(2) |
| C28-C23-C24 | 120.7(3) |
| C23-C24-H24 | 120.5 |
| C25-C24-C23 | 118.9(3) |
| C25-C24-H24 | 120.5 |
| C24-C25-H25 | 119.3 |
| C24-C25-C26 | 121.4(3) |
| C26-C25-H25 | 119.3 |
| C25-C26-C27 | 118.4(3) |
| C25-C26-C29 | 120.6(3) |
| C27-C26-C29 | 121.0(3) |
| C26-C27-H27 | 119.3 |
| C28-C27-C26 | 121.3(3) |
| C28-C27-H27 | 119.3 |
| C23-C28-H28 | 120.4 |
| C27-C28-C23 | 119.3(3) |
| C27-C28-H28 | 120.4 |
| C26-C29-H29A | 109.5 |
| C26-C29-H29B | 109.5 |
| C26-C29-H29C | 109.5 |
| H29A-C29-H29B | 109.5 |
| H29A-C29-H29C | 109.5 |
| H29B-C29-H29C | 109.5 |

Symmetry transformations used to generate equivalent atoms:

**Table S4**. Anisotropic displacement parameters ($Å^2$ x $10^3$) for **3aq**. The anisotropic displacement factor exponent takes the form: $-2\pi^2[ h^2 a^{*2}U^{11} + ... + 2 h k a^* b^* U^{12}]$

| | U11 | U22 | U33 | U23 | U13 | U12 |
|---|---|---|---|---|---|---|
| S1 | | 30(1) | | 19(1) | 25(1) | 2(1) |
| O1 | | 36(1) | | 30(1) | 48(1) | -12(1) |
| O2 | | 40(1) | | 25(1) | 34(1) | -1(1) |
| O3 | | 39(1) | | 29(1) | 32(1) | 4(1) |
| N1 | | 22(1) | | 22(1) | 22(1) | 3(1) |
| C1 | | 21(1) | | 18(1) | 25(1) | 0(1) |
| C2 | | 22(1) | | 19(1) | 21(1) | 1(1) |
| C3 | | 27(1) | | 20(1) | 22(1) | 2(1) |
| C4 | | 30(1) | | 25(1) | 36(2) | 3(1) |
| C5 | | 23(1) | | 30(2) | 30(1) | 4(1) |
| C6 | | 24(1) | | 24(1) | 25(1) | -4(1) |
| C7 | | 26(1) | | 24(1) | 27(1) | -4(1) |
| C8 | | 21(1) | | 32(2) | 26(1) | -3(1) |
| C9 | | 30(2) | | 33(2) | 30(2) | 1(1) |
| C10 | | 27(2) | | 50(2) | 29(2) | 9(1) |
| C11 | | 26(2) | | 64(2) | 23(1) | -3(2) |
| C12 | | 31(2) | | 45(2) | 30(2) | -13(1) |
| C13 | | 23(1) | | 34(2) | 28(1) | -4(1) |
| C14 | | 41(2) | | 27(1) | 33(2) | -10(1) |
| C15 | | 37(2) | | 21(2) | 33(2) | -2(1) |
| C16 | | 22(1) | | 23(1) | 25(1) | -2(1) |
| C17 | | 21(1) | | 22(1) | 26(1) | 1(1) |
| C18 | | 28(1) | | 26(2) | 31(2) | 4(1) |
| C19 | | 32(2) | | 29(1) | 50(2) | 5(2) |
| C20 | | 38(2) | | 28(2) | 48(2) | -6(1) |
| C21 | | 47(2) | | 34(2) | 32(2) | -10(1) |
| C22 | | 35(2) | | 27(1) | 28(2) | 2(1) |
| C23 | | 27(1) | | 22(1) | 22(1) | 2(1) |
| C24 | | 29(2) | | 32(1) | 24(1) | 2(1) |
| C25 | | 25(1) | | 37(2) | 28(2) | 4(1) |
| C26 | | 34(2) | | 32(2) | 26(1) | 0(1) |
| C27 | | 34(2) | | 38(2) | 30(2) | -4(1) |
| C28 | | 24(1) | | 37(2) | 30(2) | 3(1) |
| C29 | | 51(2) | | 57(2) | 45(2) | -18(2) |

**Table S5**. Hydrogen coordinates (x $10^4$) and isotropic displacement parameters (Å$^2$ x $10^3$) for **3aq**.

| | x | y | z | U(eq) |
|---|---|---|---|---|
| H1 | 6944 | 3374 | 4762 | 26 |
| H3 | 5834 | 6068 | 5810 | 29 |
| H4A | 3367 | 5418 | 5154 | 34 |
| H4B | 4380 | 4984 | 6703 | 34 |
| H5A | 3306 | 3549 | 5624 | 33 |
| H5B | 3116 | 3920 | 4073 | 33 |
| H7 | 4785 | 5214 | 2491 | 30 |
| H9 | 3432 | 6051 | 82 | 38 |
| H10 | 1998 | 5925 | -2394 | 42 |
| H11 | 1370 | 4338 | -3507 | 44 |
| H12 | 2228 | 2878 | -2172 | 42 |
| H14 | 3766 | 2028 | 275 | 40 |
| H15 | 5203 | 2172 | 2714 | 36 |
| H18 | 9281 | 6548 | 4094 | 34 |
| H19 | 10640 | 7983 | 5187 | 45 |
| H20 | 10772 | 8517 | 7423 | 51 |
| H21 | 9567 | 7619 | 8582 | 48 |
| H22 | 8178 | 6176 | 7505 | 37 |
| H24 | 9040 | 3359 | 7740 | 33 |
| H25 | 10455 | 4524 | 9495 | 37 |
| H27 | 7035 | 4958 | 10640 | 39 |
| H28 | 5605 | 3793 | 8905 | 36 |
| H29A | 10801 | 5693 | 11366 | 78 |
| H29B | 9930 | 5416 | 12325 | 78 |
| H29C | 9341 | 6292 | 11164 | 78 |

**Table S6**. Torsion angles [°] for **3aq**.

| | |
|---|---|
| S1-N1-C1-C2 | -91.4(2) |
| S1-N1-C1-C6 | 144.45(19) |
| S1-N1-C5-C4 | 88.7(3) |
| S1-C23-C24-C25 | 177.0(2) |
| S1-C23-C28-C27 | -176.8(2) |
| O1-C16-C17-C18 | 27.7(4) |
| O1-C16-C17-C22 | -148.0(3) |
| O2-S1-N1-C1 | -48.0(2) |
| O2-S1-N1-C5 | 169.0(2) |
| O2-S1-C23-C24 | 24.1(3) |
| O2-S1-C23-C28 | -157.7(2) |
| O3-S1-N1-C1 | -177.19(19) |
| O3-S1-N1-C5 | 39.8(2) |
| O3-S1-C23-C24 | 155.1(2) |
| O3-S1-C23-C28 | -26.7(3) |
| N1-S1-C23-C24 | -89.9(2) |
| N1-S1-C23-C28 | 88.4(2) |
| N1-C1-C2-C3 | -28.7(3) |
| N1-C1-C2-C16 | 156.8(2) |
| N1-C1-C6-C7 | 113.7(3) |
| N1-C1-C6-C15 | -65.5(3) |
| C1-N1-C5-C4 | -55.9(3) |
| C1-C2-C3-C4 | 5.0(4) |
| C1-C2-C16-O1 | 13.0(4) |
| C1-C2-C16-C17 | -166.9(2) |
| C1-C6-C7-C8 | -178.6(2) |
| C1-C6-C15-C14 | 177.3(3) |

| | |
|---|---|
| C2-C1-C6-C7 | -9.1(4) |
| C2-C1-C6-C15 | 171.7(2) |
| C2-C3-C4-C5 | -5.2(4) |
| C2-C16-C17-C18 | -152.4(3) |
| C2-C16-C17-C22 | 31.8(4) |
| C3-C2-C16-O1 | -161.4(3) |
| C3-C2-C16-C17 | 18.7(4) |
| C3-C4-C5-N1 | 29.9(3) |
| C5-N1-C1-C2 | 53.9(3) |
| C5-N1-C1-C6 | -70.3(3) |
| C6-C1-C2-C3 | 93.1(3) |
| C6-C1-C2-C16 | -81.4(3) |
| C6-C7-C8-C9 | -178.2(3) |
| C6-C7-C8-C13 | 1.6(4) |
| C7-C6-C15-C14 | -2.0(4) |
| C7-C8-C9-C10 | -178.9(3) |
| C7-C8-C13-C12 | 178.5(3) |
| C7-C8-C13-C14 | -2.4(4) |
| C8-C9-C10-C11 | 0.1(5) |
| C8-C13-C14-C15 | 1.1(4) |
| C9-C8-C13-C12 | -1.7(4) |
| C9-C8-C13-C14 | 177.4(3) |
| C9-C10-C11-C12 | -1.1(5) |
| C10-C11-C12-C13 | 0.7(5) |
| C11-C12-C13-C8 | 0.7(5) |
| C11-C12-C13-C14 | -178.3(3) |
| C12-C13-C14-C15 | -179.9(3) |
| C13-C8-C9-C10 | 1.3(4) |
| C13-C14-C15-C6 | 1.1(5) |

| | |
|---|---|
| C15-C6-C7-C8 | 0.6(4) |
| C16-C2-C3-C4 | 179.0(3) |
| C16-C17-C18-C19 | -176.8(3) |
| C16-C17-C22-C21 | 176.2(3) |
| C17-C18-C19-C20 | 0.4(5) |
| C18-C17-C22-C21 | 0.6(4) |
| C18-C19-C20-C21 | 0.3(5) |
| C19-C20-C21-C22 | -0.5(5) |
| C20-C21-C22-C17 | 0.0(5) |
| C22-C17-C18-C19 | -0.8(4) |
| C23-S1-N1-C1 | 66.7(2) |
| C23-S1-N1-C5 | -76.2(2) |
| C23-C24-C25-C26 | -0.2(4) |
| C24-C23-C28-C27 | 1.4(4) |
| C24-C25-C26-C27 | 1.3(5) |
| C24-C25-C26-C29 | -179.9(3) |
| C25-C26-C27-C28 | -1.1(5) |
| C26-C27-C28-C23 | -0.2(5) |
| C28-C23-C24-C25 | -1.2(4) |
| C29-C26-C27-C28 | -179.9(3) |

Symmetry transformations used to generate equivalent atoms:

Table S7. Hydrogen bonds for **3aq** [Å and °].

| D-H...A | d(D-H) | d(H...A) | d(D...A) | <(DHA) |
|---|---|---|---|---|
